# Tropical Land‐Use Change Disrupts Zeta‐Diversity Across Taxa

**DOI:** 10.1111/gcb.70245

**Published:** 2025-05-14

**Authors:** Edicson Parra‐Sanchez, Guillaume Latombe, Simon C. Mills, Jacob B. Socolar, Felicity A. Edwards, Diego Martinez‐Revelo, Oscar A. Perez‐Escobar, Robert W. Davies, Christopher G. Bousfield, Gianluca R. Cerullo, Jose Manuel Ochoa‐Quintero, Torbjørn Haugaasen, Jos Barlow, Robert P. Freckleton, David P. Edwards

**Affiliations:** ^1^ Ecology and Evolutionary Biology, School of Biosciences University of Sheffield Sheffield UK; ^2^ Department of Plant Sciences and Centre for Global Wood Security University of Cambridge Cambridge UK; ^3^ Conservation Research Institute University of Cambridge Cambridge UK; ^4^ Institute of Ecology and Evolution The University of Edinburgh Edinburgh UK; ^5^ Faculty of Environmental Sciences and Natural Resource Management Norwegian University of Life Sciences Ås Norway; ^6^ Grupo de Investigación en Ecología de Agroecosistemas y Hábitats Naturales (GEAHNA), Departamento de Biología, Facultad de Ciencias Naturales y Exactas Universidad del Valle Cali Colombia; ^7^ Royal Botanic Gardens, Kew London UK; ^8^ Department of Forest Ecosystems and Society Oregon State University Corvallis Oregon USA; ^9^ Instituto de Investigación de Recursos Biológicos Alexander von Humboldt Bogotá Colombia; ^10^ Lancaster Environment Centre Lancaster University Lancaster UK

**Keywords:** community assembly, deforestation, habitat loss, montane ecosystems, rarity, tropical Andes

## Abstract

Land‐use change causes community turnover via local extinction and colonisation of species, driving biotic homogenization or heterogenization at larger spatial scales. Quantification of these processes has focused on beta‐diversity metrics, which upweight rarity and overlook the role of widespread species. A key knowledge gap is understanding the impact of land‐use change on both rare and widespread species—zeta‐diversity—allowing the detection of statistical patterns and drivers based on community turnover across space. We sampled bird, dung beetle, and orchid communities in 341 plots across natural (Andean forests and paramo) and transformed habitats (pasturelands) spanning ~270 km north‐to‐south in the Colombian Andes. We detected major losses in species richness following land‐use conversion, which disrupts zeta‐diversity across elevation in two ways. First, biodiversity patterns are rewired such that bird and dung beetle communities become structured by dispersal ability, overriding the effects of natural biogeographical drivers (i.e., elevation) and landscape conditions (i.e., canopy cover). Second, land‐use change causes biotic homogenization across bird communities, with pasture retaining twice as many widespread species than natural habitats, and a four‐fold reduction in widespread dung beetle species pointing to subtractive heterogenization. Orchid communities show high community turnover in both natural and transformed habitat. Our results show that the effect of local deforestation has a doubly devastating impact simplifying communities and reducing widespread species. Transforming natural habitats into anthropogenic landscapes may substantially raise extinction risk for communities composed of both widespread and rare species, especially in orchids as the most sensitive taxon.

## Graphical abstract

We studied birds, dung beetles, and orchids across natural and human‐transformed habitats in the Colombian Andes to understand how land‐use change affects biodiversity. We found that deforestation simplifies ecological communities and reduces both rare and widespread species, especially orchids. These changes may increase extinction risk and undermine long‐term ecosystem resilience.

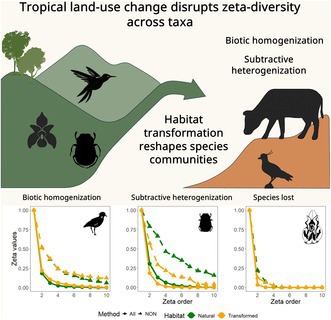

## RESUMEN

El cambio en el uso del suelo provoca recambio en las comunidades biológicas mediante la extinción local y la colonización de especies, lo que impulsa la homogeneización o heterogeneización biótica a escalas espaciales mayores. La cuantificación de estos procesos se ha centrado en métricas de beta‐diversidad, las cuales dan mayor peso a la rareza y reduciendo el papel de las especies ampliamente distribuidas. Una brecha clave en el conocimiento es comprender el impacto del cambio en el uso del suelo tanto en especies raras como en especies comunes—diversidad zeta—lo que permite detectar patrones estadísticos y sus efectos en el recambio de comunidades espacialmente. Muestreamos comunidades de aves, escarabajos estercoleros y orquídeas en 341 parcelas distribuidas en hábitats naturales (bosques andinos y páramo) y transformados (pastizales), a lo largo de ~270 km de norte a sur en los Andes colombianos. Detectamos pérdidas significativas en la riqueza de especies tras la conversión del uso del suelo, lo que altera la diversidad zeta a lo largo del gradiente altitudinal de dos maneras. Primero, los patrones de biodiversidad se reconfiguran, de modo que las comunidades de aves y escarabajos pasan a estar estructuradas principalmente por su capacidad de dispersión, superando los efectos de fuerzas biogeográficas naturales (e.g., la elevación) y las condiciones del paisaje (e.g., la cobertura de habitat). Segundo, el cambio en el uso del suelo genera homogeneización biótica en las comunidades de aves, donde los pastizales retienen el doble de especies ampliamente distribuidas en comparación con los hábitats naturales, mientras que los escarabajos estercoleros presentan una reducción de especies de habitats naturales, lo que indica una heterogeneización sustractiva. Las comunidades de orquídeas muestran un alto recambio comunitario tanto en hábitats naturales como transformados. Nuestros resultados demuestran que la deforestación local tiene un impacto doblemente devastador, al simplificar las comunidades y reducir la presencia de especies ampliamente distribuidas. La transformación de hábitats naturales en paisajes antropogénicos puede aumentar sustancialmente el riesgo de extinción para comunidades compuestas por especies tanto raras como comunes, especialmente en el caso de las orquídeas, como el taxón más sensible.

## 1 Introduction

Human activities have massively reshaped Earth's natural habitats. Between 2001 and 2023, a total of 488 Mha (12%) of tree cover was lost globally (Global Forest Watch 2024). Consequently, most species are now impacted by transformed habitats (Haddad et al. 2015), with many of these species threatened with global extinction, including 43.7% of vascular plants (Nic Lughadha et al. 2020), 13% of birds (IUCN 2020), 40% of entomofauna (Sánchez‐Bayo and Wyckhuys 2019), and an estimated 75% of undiscovered plant species (Brown et al. 2023). Understanding how biodiversity is assembled in natural habitats and then re‐structured after habitat conversion across local‐to‐regional spatial scales is thus an urgent ecological question with broad implications for conservation.

This unprecedented habitat transformation disrupts and reshape communities across different spatial scales. At local scales, the species richness (alpha diversity) of vertebrate, invertebrate, and plant communities have, on average, declined by 76% following land‐use change, particularly when the transformed habitat is structurally very different to the natural habitat it replaces (Newbold et al. 2015). At landscape‐scales, land‐use change reshapes structural and functional connectivity (via habitat loss and fragmentation). As connectivity loss disrupts recolonization events across natural and transformed habitats, restricted‐ranged species are replaced by those with larger ranges, with the average geographic range size of species 30%–50% higher in disturbed than in primary habitats (Newbold et al. 2018). However, the detectability of these local‐scale and larger‐scale impacts depends on the metric in use.

Metrics like pairwise beta diversity are commonly used to evaluate how community composition changes with habitat transformation from local to gamma (regional) spatial scales (Socolar et al. 2016). The dominant beta diversity pattern is that communities are more similar between them across space following anthropogenization of natural habitats (Socolar et al. 2016; Kramer et al. 2023; Montràs‐Janer et al. 2024). For example, bird, plant, and butterfly communities are spatially more similar after human intervention of habitats, as beta diversity showed a decreased between 3% and 53% in community composition (Montràs‐Janer et al. 2024). Other taxa such as orchids can reach 100% turnover of orchid species post‐deforestation (Parra‐Sanchez et al. 2023a). This points towards the loss of geographically restricted‐range species and increases in species with larger ranges that can cope with conditions in transformed habitats over larger spatial scales (e.g., Wallacea and Central America; Karp et al. 2012; Mitchell et al. 2022).

Understanding how widespread and geographically restricted species respond to habitat transformation can improve conservation actions. Species with widespread geographical distribution, often dominate ecological processes (e.g., 227 trees species hyper‐dominate the Amazon basin tree diversity; ter Steege et al. 2013), and play a crucial role in maintaining ecosystem structure and global functional diversity (Lennon et al. 2004; Affleck and McGeoch 2024). In contrast, rare species, despite their limited distribution, contribute critically to ecological functions (Mouillot et al. 2013), whilst serving as indicators of environmental change (Enquist et al. 2019) because they are more sensitive to the novel conditions in transformed habitats (McKinney and Lockwood 1999; Tabarelli et al. 2012; Newbold et al. 2018). In contrast, widespread species are typically better adapted to disturbance in part due to resource use in transformed habitats (Tabarelli et al. 2012; Newbold et al. 2018). For example, orchid bees appear to be insensitive to high transformation of natural habitats because of their strong dispersal capacity (Crall et al. 2020; Nunes et al. 2022), whereas bird species from natural grassland ecosystems (e.g., eastern meadowlark *Sturnella magna* , Andean lapwing *Vanellus resplendens* ) can invade anthropogenic grasslands (Gilroy et al. 2014a). However, widespread species have experienced declines in populations and contraction of geographical ranges (van Klink et al. 2024). By considering the dynamics of both common and rare species, we can gain a more comprehensive understanding of how biodiversity responds to land‐use change across local and regional scales.

Disentangling the spatial distribution of rare to widespread species is essential to adequately understand the impact of habitat transformation on community turnover. Zeta (ζ) diversity—the number of species shared by any number of sites or species assemblages (Hui and McGeoch 2014)—fills this knowledge gap by comparing multiple sites simultaneously (the number of sites is termed the order of zeta diversity), rather than contrasting and averaging pairs of sites to generate pairwise dissimilarity metrics of beta‐diversity (Hui and McGeoch 2014; Latombe et al. 2017). As the number of assemblages being combined increases, rare species are progressively excluded and only widespread species remain, allowing consideration of the continuum from very rare to the most widespread species. This distinction is especially useful in detecting biotic homogenization as widespread ‘winner’ species benefit from transformed habitats and rare ‘loser’ species decline (McKinney and Lockwood 1999; Newbold et al. 2018) leading to communities that are more compositionally similar across space. By contrast, the opposite (an increase in dissimilarity among communities via the loss of widespread species) drives subtractive biotic heterogenization (McKinney and Lockwood 1999; Tabarelli et al. 2012). By contrast, pairwise beta‐diversity comparisons, which correspond to zeta diversity of order 2, are biased towards the contribution of rare species to turnover (Jost 2007), and can overestimate the impact of habitat transformation on rare species in community turnover, while overlooking its effect along the spectrum from rare to widespread species (Hui and McGeoch 2014).

To understand how local‐scale conversion affects larger‐scale patterns of community structure, we disentangle the contribution of rare and widespread species to community turnover. We do so for three biological groups spanning vertebrates (birds), invertebrates (dung beetles), and plants (orchids) sampled simultaneously across a wide elevational gradient (1163–3415 masl) in the global hotspot of Colombia and chosen as indicators of human‐driven disturbances (Gilroy et al. 2014b; Parra‐Sanchez et al. 2024). Colombia is the world's epicentre of birds (1966 species; Ángela Echeverry‐Galvis et al. 2022) and orchid diversity (~4300 spp.; POWO 2023) diversity, and the second most diverse country for dung beetles (366 spp.; Schoolmeesters 2022). We used zeta‐diversity to unravel the drivers that trigger community‐level patterns across space that shape community assembly of rare to widespread species by asking: (1) what biophysical variables drive community turnover across natural and transformed habitats, while accounting for the contribution of rare to widespread species to turnover? and (2) how are communities structured across natural and transformed habitats? We hypothesize that the drivers of community turnover will shift between natural and transformed habitats, requiring habitat‐specific conservation strategies. Additionally, we expect transformed habitats to favour widespread species, consistent with patterns of biotic homogenization (McKinney and Lockwood 1999; Kramer et al. 2023; Montràs‐Janer et al. 2024). Understanding community turnover is essential for informing conservation efforts, including the creation of protected area networks that safeguard ecologically diverse communities and reduce the risk of species extinction (Socolar et al. 2016).

## 2 Methods

### 2.1 Study Area

The research was carried out under the conceptual framework of the ‘Provisioning of ecosystem services And cultuRAl values in the MOntane tropics’ project, which focused on how best to incorporate and optimise the combination of biodiversity, ecosystem services, and cultural values within natural resource management in Colombia. The study was conducted in Cundinamarca, Boyacá, Meta, and Santander, encompassing the eastern and western slopes of the eastern Andes cordillera, in Colombia. Natural habitats included remnants of Andean Forest, paramo forests (high‐elevation forest > 3200 masl), and paramo shrublands and grasslands. Human‐transformed habitats were areas that have undergone high local deforestation, where the natural habitat has completely been transformed mainly for cattle production, comprising Andean and paramo pastures utilized mainly for grazing. Habitats have experienced historical anthropogenic disturbances due to their accessibility via roads or footpaths, as confirmed by information provided by local field assistants and landowners. All forests were part of Colombia's protected areas network known as the Sistema Nacional de areas Protegidas (Ministerio de Ambiente y Desarrollo Sostenible 2023).

### 2.2 Sampling Design

**FIGURE 1:**
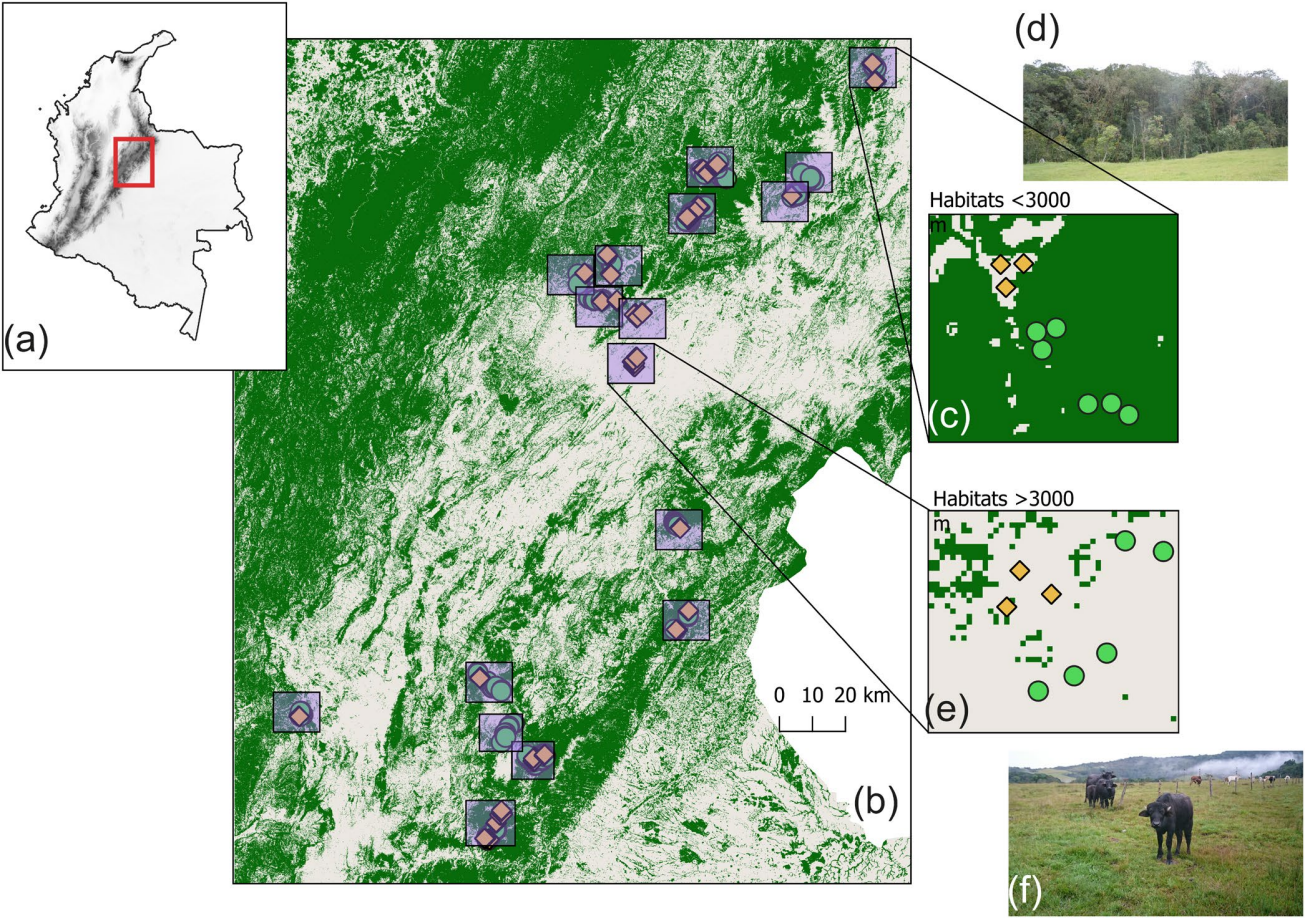
Distribution of sampling plots in the study area, the eastern cordillera of the Colombian Andes. (a) study area within Colombia (red box), and elevation (digital elevation model AlosPalsar; Tadono et al. 2014). (b) map shows forest cover (green) and the absence of forest cover (grey), and sampling plots in natural (green dots) and transformed habitats (yellow rhomboids) within 18 landscapes (pink box). (c) landscape window of 3 × 3 km < 3000 masl. (d) photo depicting a natural habitat at 2100 masl in Gambita, Santander. (e) landscape window of 3 × 3 km > 3000 m. (f) photo depicting a typical paramo pastures at 3400 m in Belen, Boyacá. Map lines delineate study areas and do not necessarily depict accepted national boundaries.

We designed our sampling to represent two types of habitats depending on land transformation to capture the effects of land‐use in community turnover. Across the study area, we sampled 18 landscapes composed of natural and transformed habitats (Figure 1). Each landscape was defined as an area of 9 km^2^ where natural habitats were paired with transformed habitats. This approach allows us to have both habitats at approximately the same elevation and reduce the effects of topography and other landscape‐scale and climate variables unaccounted for in the models. All natural habitat points were placed at least 50 m from the forest edge or roads keeping a minimum of 172 m distance apart (range = 172.3–2759 m). We set up transformed habitat plots at least 60 m away from the forest edge and at a minimum of 193 m apart between them (range = 193–4225 m; *n* = 114 plots in 22 pasturelands). Transformed habitats correspond to Andean pastures characterised by having sparse trees and grasses mainly of the Poaceae family, and Paramo pastures were dominated by grasses and lacked frailejones species (Asteraceae: *Espeletiinae* subtribe), which are a cluster of highly endemic, slow‐growing species with high sensitivity to disturbance and narrowly restricted geographical distribution (Cortés et al. 2018).

We sampled 341 plots across a wide elevational range of 1163–3763 m (4°C–28°C and 879–3817 mm per year), spanning ~270 km in distance. Specifically, 206 plots were in natural habitats (148 forest, 48 paramo, and 10 paramo forest), and 135 plots in transformed habitats (90 pastures next to Andean forests and 45 plots in transformed Paramos, *n* = 341). We sampled birds, dung beetles, and orchids from January 2019 to March 2020, with sampling conducted simultaneously across taxa in time and space. Due to the nature of the cross‐species sampling we used points for animal taxa and plots for orchids, and these terms will be used throughout the text. We sampled between 2 and 18 sampling points randomly placed within forests and kept a minimum of 200 m distance apart from each other (with only two cases where points were placed around 170 due to topography). Larger habitats had more plots to ensure representative cover. All sampling plots were placed at least 30 m from the forest edge to reduce the impact from cattle (Parra Sánchez et al. 2016). The sampling area of each sampling point differed between groups (orchids = 10 × 30 m plots; dung beetles = ~ 100 m radius; birds = 100 m radius; see survey explanation below).

### 2.3 Birds Survey

Bird communities were sampled using repeat‐visit point counts at each sampling point (Mills et al. 2022). This methodology allows for detections within an estimated 100‐m radius (Gilroy et al. 2014b). Points were visited on four consecutive days for counts of 10‐min duration (sampling times between 05:00 and 12:00 pm). Visiting time to each point was randomised to ensure that each point was visited both early and late in the morning during sampling days. Counts were carried out by one person at a time, avoiding heavy rain, dense mist, or high winds, because these conditions reduce visibility, introduce noise to recordings, and alter species behaviour. Each survey was recorded using a recording device to record vocalizations. Species identification was done in the field and subsequent confirmation using recordings against online reference material (www.xeno‐canto.org, and recordings deposited in the ‘Coleccion de Sonidos Ambientales’ from Instituto Alexander von Humboldt, Colombia). We omitted species that exhibit high mobility or transient behaviour making them unreliable indicators of local environmental conditions (e.g., non‐breeding trans‐continental migrants, large raptors and swifts). Non‐breeding transcontinental migrants may only be present temporarily, large raptors often have large home ranges that extend beyond the surveyed areas, and swifts spend most of their time in flight, making detection inconsistent.

### 2.4 Dung Beetle Survey

Baited pitfall traps were used to sample dung beetles (Coleoptera: Scarabaeidae: Scarabaeinae). We placed one trap at each sampling point and insects were collected every 24‐h across four sampling days. Traps were set with fresh human faeces as bait and rebaited after 2 days. This type of bait attracts a wide range of dung‐feeding species to give the best representative sample of the community (Larsen and Forsyth 2005; Mora‐Aguilar et al. 2023). Species determinations were carried out by Diego Martínez and F. Edwards at the Instituto Alexander von Humboldt (IvAH), Colombia. All specimens were deposited in the biological collections at IvAH.

### 2.5 Orchid Survey

Orchids were sampled in a 10 × 30 m plot at each sampling point, recording both terrestrial and epiphytic orchids within the plots, from the ground up to a height of 2 m (Parra‐Sanchez et al. 2023b). All standing tree trunks, fallen tree trunks and branches, vines, lianas, leaves on standing trees or herbaceous plants, palm trees, tree ferns and cycads were sampled. Canopy orchid species could have been sampled from fallen branches, but because we used a standardized sampling approach across all sites, every plot had an equal chance of capturing canopy orchids. Only mature individuals with developed floral or reproductive structures, large clumps of stems, and prominent root systems were recorded. Plant individuals without flowers were translocated to nearby nurseries and subsequently visited for identification. Identification to the species or morphospecies level was carried out using specialized literature and consultation with local experts at the Herbarium VALLE in Palmira, Colombia.

### 2.6 Habitat and Landscape Predictors

We selected predictors that represent different determinants of community composition (Myers et al. 2000; Rahbek et al. 2019; Parra‐Sanchez et al. 2023a). First, we used tree density and canopy cover to capture forest structure. Second, we used the proportion of forest cover in 1 km radius (hereafter forest cover), number of forest fragment per area (hereafter fragmentation), and disturbance to assess the effect of landscape composition and configuration on community turnover. Third, we used elevation and precipitation as natural biogeographic drivers of species richness. Finally, we used geographical distance between sampling points as a proxy linked to dispersal (Loke and Chisholm 2023). Geographical distance was calculated as spherical distance in the modelling process (Latombe et al. 2017).

At a local scale, we measured tree density (trees per plot) and percentage of canopy cover as predictors of forest structure on trees > 10 cm DBH (diameter at breast height). Predictors were quantified at each 10 × 30 m plot. Canopy cover was measured as the percentage of canopy cover in the sampling plot from the 30‐m resolution global change forest map (hereafter referred to as the GCF map; Hansen et al. 2013).

At the landscape‐scale, we quantified three predictors linked to different ecological processes: (1) percent of forest cover as a metric of habitat available in the landscape (Fahrig 2013); (2) the number of forest patches present in the local landscape as a proxy for fragmentation (Fahrig 2013) and (3) disturbance, calculated as the total of forest pixels that have undergone any change over a span of 18 years (2005–2022) using data from Global Forest Watch (2024). Percent of forest cover and number of forest patches were measured from GCF by 2010 (Hansen et al. 2013), which was converted into a binary forest/non‐forest cover map (using the 50% threshold; non‐forest <= 50%, and > 50% labelled as forest). Due to the spatial resolution of the GCF, analyses were constrained to forest fragments > 9 ha. Disturbance was defined as a single metric of forest disturbance that included both deforestation and degradation (forest degradation areas defined by Vancutsem et al. 2021). Disturbance included deforested land (permanent conversion from moist forest cover to another land cover), degraded forest (where disturbances were observed over a short period), forest regrowth (post‐deforestation recovery), conversion to plantations, conversion to water, and afforestation (regrowth of areas initially classified as non‐forest cover). These predictors were obtained at a 1000 m resolution, which also matches the climatic predictors (as the finest scale available), as we did not quantify the scale‐of‐effects (Jackson and Fahrig 2015).

Finally, elevation (m.a.s.l.) and precipitation (mm per year) were included as environmental predictors involved in driving biogeographical patterns. Mean annual precipitation was extracted from CHELSA (1 km resolution MAP; climatologies at high resolution for the Earth's land surface areas; Karger et al. 2017). Elevation was measured at each sampling point with a GPS garminC60 and validated with the 12.5‐m ALosPalsar´s Radiometric terrain model using data from ASF DAAC (2015). Multicollinearity among climatic and landscape predictors was weak using the variance inflation factor (VIF range = 1.15–4.09, Table S1).

### 2.7 Data Analysis

#### 2.7.1 Land‐Use Change Alters Drivers and Patterns of Community Structure

##### 2.7.1.1 Alpha Diversity

Generalised linear mixed models (GLMMs) were fitted for each taxonomical group to assess how species richness varied across land‐uses. Observed species richness at plot level were used as a response variable. Land‐use was used as a predictor and treated as a dichotomous factorial variable (natural and transformed) and landscapes as random effects. We used a Poisson error distribution, confidence Intervals (CIs at 95%) and fit by maximum likelihood. Models were assessed for normality of residuals, overdispersion, homogeneity of variance, and outliers using the DHARMa package (Kolmogorov–Smirnov test, nonparametric dispersion, quantile, and outlier tests; Hartig 2018). Results confirm that no assumptions were violated (see details in Supporting Information—S1).

##### 2.7.1.2 Zeta‐Diversity

We ran analyses across both natural (*n* = 206) and transformed (*n* = 135) habitats to unravel how rare and widespread species are structuring Andean communities (birds, dung beetles, and orchids). All analyses were based on occurrence data at the spatial scale of the sampling points.

Zeta‐diversity quantifies the variation in species composition of multiple communities in geographical space (or time), to capture the contribution of rare and widespread species to biotic heterogeneity (Hui and McGeoch 2014). Zeta‐diversity of order two (ζ_2_) calculates the average number of species shared by any two sites (hereafter beta diversity), ζ_3_ is the average number of species shared by any three sites, and so forth. As the zeta order increases, rare species are excluded from the analyses. Using a range of zeta orders therefore allows capturing the full spectrum of rare, intermediate, and widespread species as they contribute differently to compositional turnover. The effect of environmental heterogeneity and spatial distance on species turnover was assessed using Multi‐Site Generalized Dissimilarity Modelling for zeta orders ζ_2‐7_ (MS‐GDM), therefore examining how drivers of turnover vary with species commonness (Latombe et al. 2017).

We assessed the role of elevation, precipitation, spatial distance, and landscape‐scale structure (fragmentation, habitat loss, and land‐use change) in explaining species turnover for different orders of zeta using MS‐GDM (Latombe et al. 2017). MS‐GDM is computed separately for different orders of zeta, capturing the effect of the different drivers on the change in community composition for rare to widespread species (ζ_2‐7_, respectively; Latombe et al. 2017). We used the richness‐independent transformation method of Simpson‐normalization computed as the ratio of the ζ_i_ value for *i* specific sites divided by the minimum richness across the *i* sites, to account for differences in species richness between taxonomic group (i.e., alpha diversity) and provide output that was comparable among datasets (McGeoch et al. 2019).

An MS‐GDM is a generalised linear model (Binomial family [link = ‘log’]) with a constraint on the signs of the coefficient, applied to a monotonic transformation of the predictors. It generates a non‐linear relationship between each explanatory variable and zeta‐diversity, modelled as an I‐spline. I‐splines are interpreted based on the two following properties: (i) The relative amplitude of a spline (along the y‐axis) gives an indication of the effect of a particular explanatory variable on zeta‐diversity relative to other variables (Latombe et al. 2017); and (ii) the shape of the I‐splines curves provide insights on the range of values over which a variable has an important effect, with steeper slopes meaning greater importance. We also included spatial distance calculated from the geographical coordinates using the orthodromic distance method. For distance, a spline simply represents the distance‐decay of similarity (Latombe et al. 2017; McGeoch et al. 2019).

As the number of combinations of *i* sites across all sites can become extremely high, we ran 50 MS‐GDM for each order of zeta and each taxon, using a random sample of 1000 site combinations for each MS‐GDM. Below we present the median of the 50 splines for each predictor, whereas all splines can be found in supplementary material. The variability between the 50 replicates provides an estimate of confidence for each predictor. We show the outcome plotting the I‐splines. The *x*‐axis represents the rescaled value of all predictors (zero‐to‐one). The *y*‐axis represents the relative importance of each model in capturing the influence of different variable sets on community turnover at each zeta order independently. Given the nature of the data, we use different *y*‐axis scales for the dung beetle community to improve interpretability. The absolute values of the I‐splines are less informative than their relative amplitudes and the model's explanatory power. These amplitudes depend on the distribution of zeta values, which varies across taxa and environments. A fixed *y*‐axis scale would obscure differences in some cases, so a custom scale is preferable to improve interpretability.

The variance explained for each model (i.e., for each replicate) was calculated as Pearson r^2^ between the observed and the predicted zeta values. Results are presented as the full range of variance of zeta‐values and the mean and standard deviation at each zeta order. The most informative predictor was selected based upon the maximum value of the spline.

#### 2.7.2 Land‐Use Change has Diverse, Pervasive Effects on Montane Community Structures

We further explore the zeta‐diversity decline as the trend across sampling points of rare‐to‐widespread species in each taxon and habitat type. As the zeta order increases, the zeta values (ζi) decrease, indicating the rate of change in species turnover from rare to widespread species across space (McGeoch et al. 2019). As before, we rescaled zeta values using the equivalent of Simpson's dissimilarity index in the analyses to assess the effects of predictors on species turnover caused by species replacement and not richness difference (Jost 2007).

Zeta decline was tested using two spatial sampling schemes to capture the spatial structure of species distribution at the community level (McGeoch et al. 2019). First, the ‘all combinations’ (ALL) spatial arrangement considers combinations of points independently from their geographical position. Points that are far from each other are therefore less likely to share species than closer sites if isolation by distance structures the ecological communities. By contrast, the ‘nearest neighbours non‐directional’ (NON) sampling scheme combines points based on a nearest‐neighbour approach based on their spatial coordinates in the computation of the zeta values. The comparison between zeta‐diversity declines computed with these two schemes therefore allows to unpack how communities are structured in space, and if the spatial structure differs for rare to widespread species.

**FIGURE 2:**
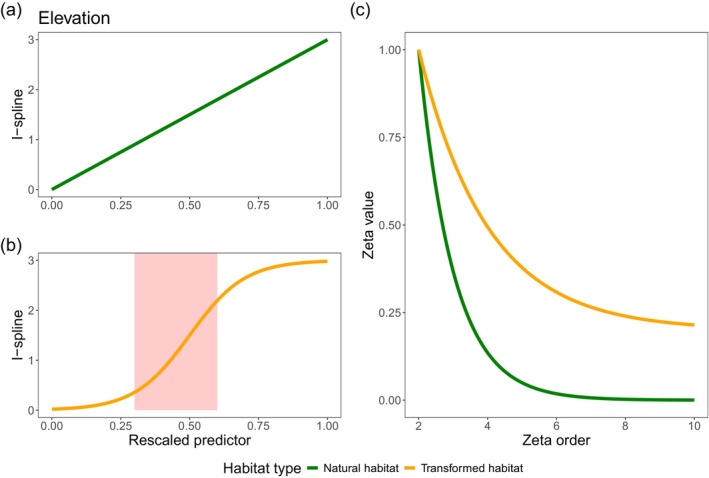
Hypotheses testing on community turnover across taxa. (a) Steady slopes mean consistent community turnover in relation with elevation across natural habitats. (b) inflexion points (red box) depict rapid species turnover across transformed habitats at mid‐elevations and low turnover at low and high elevations. This contrasting pattern shows the effect of land‐use change on community turnover across an elevational gradient regardless of the taxonomic group. (c) Proportionally, we expect more widespread species in transformed than in natural habitats as a signal of biotic homogenization due to land‐use change (green = natural habitat, orange = transformed habitat).

We first hypothesized that major biophysical drivers of community turnover in natural habitats (e.g., elevation, precipitation, high forest cover and low fragmentation; Fahrig 2017; Watling et al. 2020; Arroyo‐Rodríguez et al. 2020), would be replaced by local disturbance and high fragmentation in transformed habitats. For instance, communities in natural habitats would have a steady turnover along the elevational gradient, but this pattern would be shifted in transformed habitats where it is expected to have the highest turnover at mid‐elevations (Figure 2). Our second hypothesis is that across taxa we expect more widespread species in transformed than in natural habitats, which reflect biotic homogenization (Kramer et al. 2023; Montràs‐Janer et al. 2024).

##### 2.7.2.1 Software

All analyses were performed in R studio (R Core Team 2021). Data manipulation was done with tidyverse (Wickham et al. 2019), landscape metric calculations with *landscapemetrics* (Hesselbarth et al. 2019), zeta analyses with *zetadiv* 1.1.1. (Latombe et al. 2017), and plotting using ggplot2 (Wickham 2016).

## 3 Results

We recorded 537 bird species across 95.6% of sampled points (*n* = 326), 123 dung beetle species in 60.3% of sampling points (*n* = 205), and 332 orchid species in 52.6% of sampling plots (*n* = 179). In natural habitats, we found geographical range expansions of two bird species (Socolar et al. 2022), records of 12 dung beetles endemic to Colombia, and 11 orchid species new to science (e.g., Parra‐Sanchez et al. 2023b), as well as 15 species of global conservation concern (one bird species critically endangered, five bird and two dung beetle species endangered, and seven bird and dung beetle species vulnerable; IUCN 2020).

### 3.1 Land‐Use Change Alters Drivers and Patterns of Community Turnover

**FIGURE 3:**
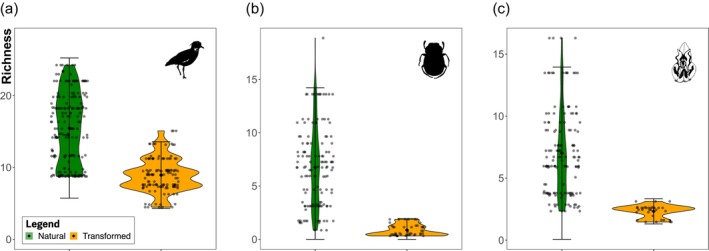
Species richness per taxonomic group across two land‐uses in the eastern Andes, Colombia. Violin plots represent the effects of land‐use on species richness per plot across bird (a), dung beetle (b), and orchid communities (c). Violin plots show the median (solid line), 25% and 75% quantiles (boxes); whiskers extend to the minimum and maximum within 1.5 times the interquartile range. The shaded area shows the kernel density of the variability range in natural (green) and transformed habitats (dark orange), whilst the dots represent the predicted values based on generalized linear models (GLMM).

The conversion of natural habitats to pastures disrupted the spatial structure of communities. Communities in natural habitats had 71.7%, 86.5%, and 166.6% higher species richness than communities in transformed habitats for birds, dung beetles, and orchids, respectively (Figure 3). These effects were statistically significant across taxa (all *p*‐values < 0.001; Birds, beta = −0.60, CI [−0.77, −0.43]; Dung beetles, beta = −2.04, CI [−2.74, −1.41]; and orchids, beta = −0.99, CI [−1.45, −0.52]; Table S2). The explanatory power across models was robust (conditional *R* ^2^ = 0.62–0.79; marginal *R* ^2^ = 0.22–0.43).

Land‐use change modified community composition in two ways. First, in natural habitats, elevation was the prevalent force shaping community turnover, but habitat transformation rewired this pattern (ζ_2‐7_; average splines over 50 replicates; Figure 4; Figures S1–S12; Appendix S1 MS‐GDM model output). Second, transforming natural habitats into pastures changed the scale at which biophysical factors influence community structure.

**FIGURE 4:**
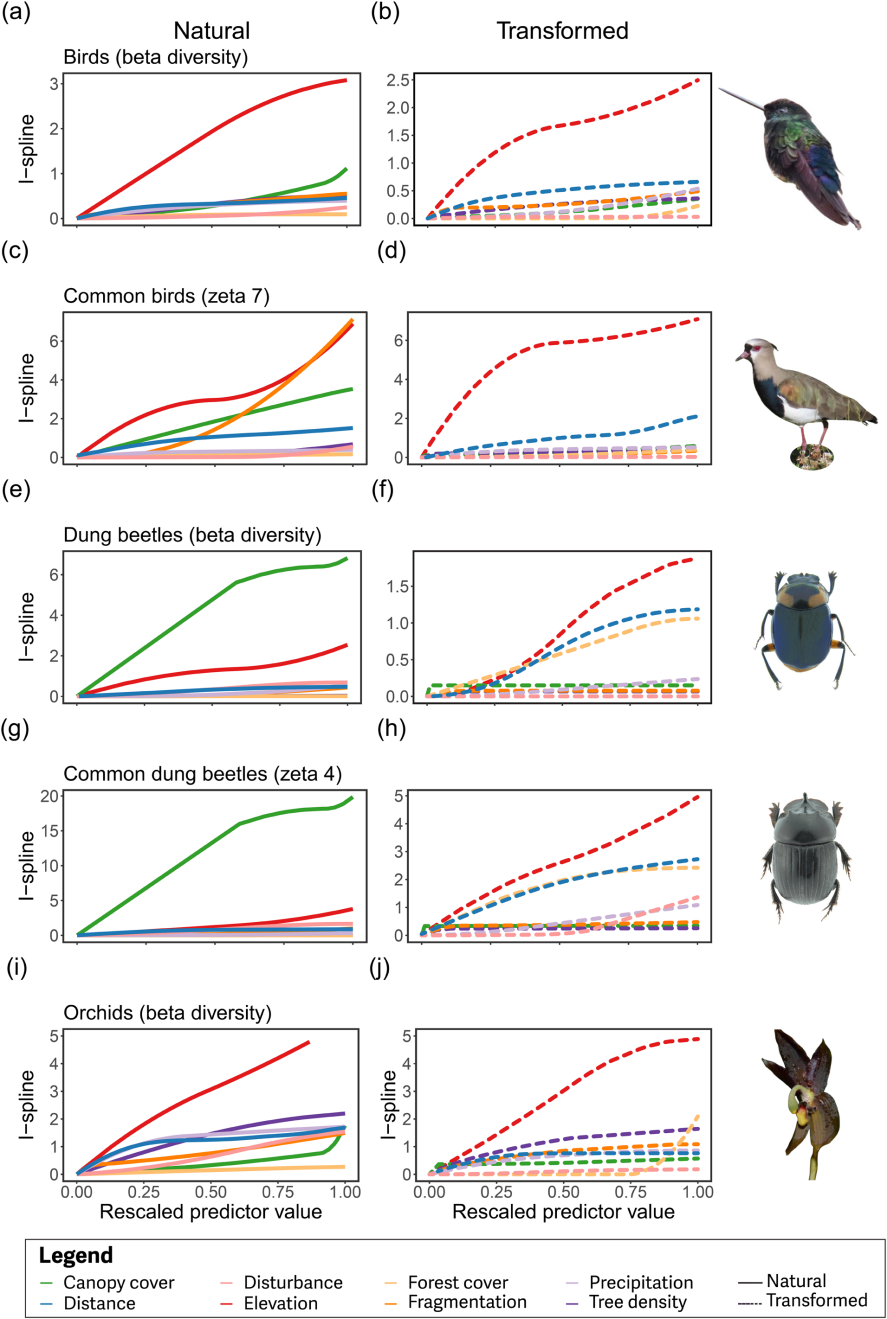
Relative importance (amplitude of the mean I‐splines) and contribution to turnover (slope of the mean I‐splines) of geographic distance and biophysical drivers of compositional turnover of birds (a–d), dung beetles (e‐h), and orchid communities (i–l). Plots depict each taxon across natural (a, c, e, g, and i), and transformed habitats (b, d, f, h, j). Results are shown for rare species as ζ_2_ and widespread species as ζ_7_ in bird and ζ_4_ in dung beetle communities. Lines depict the mean I‐splines derived from the MS‐GDM models based on 50 repetitions with a sampling size of 1000 combinations. The *x*‐axis shows the rescaled value from zero to one within the range of each predictor. The *y*‐axis depicts the relative importance of each model that accommodates the influence of each set of variables on community turnover at each zeta order independently. Thus, we present custom y‐axis scales to enhance interpretability due to the nature of the data. Photos depict examples of rare species for zeta order 2 ( *Coeligena prunellei* , *Canthon arcabuquensis*, and *Maxillaria rhomboglossa*), and common species for zeta order > 4 ( *Vanellus resplendens* and *Ontherus diabolicus*). Photographs by Marcela Avellaneda (birds), Diego Martinez‐Revelo (dung beetles), and Edicson Parra‐Sanchez (orchid).

For bird communities, we found that ζ_2_ in natural habitats (beta‐diversity) shifted from a consistent turnover of species along the whole elevation gradient to a rapid loss of species at low elevations in pasturelands (1100–1600 m; Pearson's *R* ^2^ = 0.45–0.57 in *ζ*_2_; Figure 4a,b). For widespread bird species (ζ_> 4_), turnover was high at lower (< 1500 m) and high elevation (> 2900 m), but low at intermediate elevations, as shown by the steep slope of the splines at low and high values and the plateau at intermediate values in natural habitats (*R* ^2^ = 0.19–0.52 in *ζ*_7_; Figure 4b). This pattern changed in transformed habitats, where the highest turnover happens at low elevations (< 1600 m) and mostly disappeared at high elevations (< 3000 m), meaning low turnover of widespread species (steady curves; Figure 4d). In addition, the variance explained by MS‐GDM decreased as the zeta order increased in transformed habitats, in contrast to natural habitats, consistent with the failure to identify environmental drivers of species turnover for widespread species (Supporting Information S1; Figure S13).

In natural habitats, ζ_2_ of dung beetles (beta diversity, Figure 3) was steady at mid‐elevations (1500–2500 m), then peaked at high‐elevations (> 2500 m), but in transformed habitats turnover was low at low elevations (1100–1600 m), then switch to a rapid turnover at mid‐elevation (~1600 m; Pearson's *R* ^2^ = 26–0.34 in *ζ*_2_). For high orders (ζ_4_), widespread dung beetle species across natural habitats increased at the same elevation (> 2500 m), but turnover remains steady in transformed habitats (Pearson's *R* ^2^ = 0.20–0.22 in *ζ*_5_ showing low steady slopes ζ_> 3_). Orchid community had low turnover at high elevation in transformed habitats (1150– ~1700 m), whereas in natural habitats, elevation had a consistent effect on turnover across the whole gradient (steady slope across the entire gradient at ζ_2_; Figure 4c,d; Pearson's *R* ^2^ = 0.05; Figure S13). MS‐GDM models showed good deviance explained across taxa (Figure S13).

Second, in natural habitats, local‐scale canopy cover was the second most important determinant of bird and dung beetle communities (but first in widespread dung beetles ζ_4_), causing a sharp rise in turnover between 10% and 50% cover, but minimal impact on community turnover above 50% canopy cover (Figure 4a,c,e,g). By contrast, in transformed habitats, geographical distance and landscape‐scale forest cover emerged as strong forces of community turnover after elevation (high distance ~10 km, and mid‐to‐high forest cover 70%–100% at 1 km resolution).

### 3.2 Land‐Use Change Has Diverse Effects on Montane Community Structures

**FIGURE 5:**
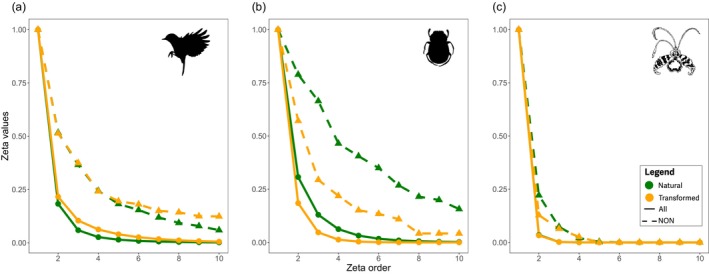
Proportion of species shared by multiple sites using Simpson‐normalization zeta declines across birds (a), dung beetles (b), and orchid (c) communities across all sampling units irrespective of their geographical position (i.e., ALL, continuous line) and the nearest neighbour (i.e., NON, dashed line) across natural (green) and transform habitats (orange). As the zeta order increases, the zeta values (ζi) decrease, indicating the rate of change in species turnover from rare to widespread species. In axis‐*y*, zeta diversity represents the total number of species (after Simpson‐normalization) shared across sampling units in axis‐*x*. Zeta‐diversity decline indicates changes in community composition.

We found patterns of biotic homogenization on birds and subtractive biotic heterogenization of dung beetles driven by changing patterns of widespread species post‐deforestation, whilst we found a rapid decline in zeta towards zero in orchid communities demonstrates strongly localised species in both habitats (Table S3). Zeta‐decline models show that neighbouring sites (sampling of nearest‐neighbours ‐NON) were more likely to share species compared to random sites, suggesting that the structure of ecological communities is driven by isolation by distance (sampling across all‐combinations ‐ALL; Figure 5, Table S3).

Our models revealed a larger set of widespread bird species in transformed habitats showing signs of additive homogenization (natural ~10% vs. transformed~20% of the communities, by ζ_10_ in NON sampling, Figure 5a), with concurrent loss of smaller‐ranged species from natural habitats (subtractive homogenization). In contrast, widespread dung beetles dominate natural habitats, but habitat transformation increases dung beetle community turnover towards a lower proportion of widespread species revealing subtractive heterogenization (natural ~20% vs. transformed ~5% NON, Figure 5b). This suggests that closer communities in natural habitats are more similar to each other (isolation by distance) in dung beetle communities than across bird and orchid communities. However, land‐use change disrupted the effects of this spatial structure by reducing the prevalence of widespread dung beetles in natural habitats. Finally, orchid communities were mostly composed of rare species without spatial structure (ζ_2_, Figure 5c) and changes in composition were due to the loss of species richness (Figures 2c and 5).

## 4 Discussion

Our study across bird, dung beetle, and orchid communities reveals taxon‐dependent patterns that converge into two general conclusions. First, local deforestation reduces species pools, leading to lower species richness in transformed habitats, and reshapes communities, overriding the effects of natural biogeographical forces (i.e., elevation) and landscape conditions (i.e., high canopy cover). This confirms our first hypothesis that deforestation disrupts biophysical drivers of community structure. Second, we detected distinct community‐level responses to local deforestation, ranging from biotic homogenization to subtractive biotic heterogenization. Our findings partially reject our second hypothesis that land‐use causes communities to shift towards biotic homogenization. We unravel co‐occurring processes obscured when using only beta‐diversity.

We provide evidence that Andean natural habitats have an irreplaceable role in maintaining rich and diverse communities. We further show that orchids have the highest sensitivity to deforestation and the highest proportion of rare species compared to birds and dung beetles in this global biodiversity hotspot.

### 4.1 Land‐Use Change Alters Drivers and Patterns of Community Turnover

Once natural habitats are converted to pasturelands, entire communities are reshaped as many species are unable to tolerate conditions in the transformed habitat. Our results align with the idea that land‐use change can impact the effects of biogeographical forces such as elevation that govern community composition and structure (Peters et al. 2019). However, the novelty of our study lies in detecting specific elevations where land‐use change exerts profound impacts on ecological patterns across the rare‐to‐widespread species spectrum.

Specifically, at low elevations in pasturelands (1100— ~1600 m), the rapid decline in bird species underscores the difficulty many species face in coping with transformed habitats. In the Andes, the highest bird diversity occurs at lower elevations, where land‐use transformation intensity is high (Clark et al. 2012). Conversely, at mid‐to‐high elevations (~2500–3700 m), the low turnover of widespread bird species signals a group of dominant species can persist in transformed environments. This stands in stark contrast to natural habitats, where widespread bird species show high turnover in the opposite direction at higher elevations (~2500–3700 m). Our findings align with the idea that high‐elevation bird communities might be preadapted or filtered for species that can survive when levels of forest cover in the landscape are naturally low (Betts et al. 2019; Mills et al. 2022). High‐elevation landscapes in natural habitats present high turnover of widespread species, which might indicate a degree of species specialization to conditions such as sparse tree distribution, with encroached growth, and big canopy gaps (Bader et al. 2007; Betts et al. 2019). Our results contrast with patterns in the Australian avifauna where the highest shift from rare to widespread species occurs at low elevations. Instead, in the Andes, elevation influenced community turnover along the whole gradient (McGeoch et al. 2019). These differences may stem from several biogeographic factors such as the far larger elevational range in our study area (0‐ ~ 500 m Australia vs. 1100–3700 m this study) and the richer endemism of Andean bird communities (Myers et al. 2000).

We found that natural habitats along the whole elevation gradient retain more dung beetles and more diverse communities, which are largely lost post‐deforestation. This might be explained by the high dependency of dung beetles to good habitat conditions such as low sun‐exposure and high dung availability in natural habitats (Davis et al. 2001; Horgan 2005; Nichols et al. 2009). For instance, canopy cover is high in natural habitats which influences dung beetle community assembly by modulating local microclimates on which widespread dung beetles might rely (Williamson et al. 2022). Experimental gaps reduced the abundance of a key forest species (*Anoplotrupes stercorosus*), without compensatory recruitment of open land species (Staab et al. 2022). In contrast, transformed habitats present lower species richness, with fewer widespread species. This pattern might be explained by the availability and quality of dungs which are either present but poisonous for dung beetles or scarce. When cattle are present, farmers often use ivermectin that intoxicate dung beetle species potentially provoking a cascading effect in turnover (Verdú et al. 2015). Furthermore, many pasturelands have been abandoned in high Andean elevations (Clark et al. 2012), suggesting the availability of dung might be low or dependent on (often overhunted) mammals from nearby natural habitats (Nichols et al. 2009). To deepen our understanding of whether deterministic processes are occurring, traits can be used to reveal the mechanisms involved in community turnover (Edwards et al. 2021).

Orchid communities are composed of highly specialized species that become locally extinct after habitat transformation and orchid conservation should therefore focus on species richness (no signal of widespread species; Parra‐Sanchez et al. 2023a). Thus, land‐use drives species loss and alters the patterns of orchid turnover. Orchid communities have an inherent high turnover across elevation due to species rarity, but transformation of habitats at high elevations (> 3000 m) leads to complete local extirpation of species (Parra‐Sanchez et al. 2024). Our results underscore the dependency of epiphytic orchid species (~92% of orchids in this study) upon good‐quality habitat structure (Gentry and Dodson 1987; Zotz 2016; Reid et al. 2016), and align with the notion that orchid communities might require connectivity across landscapes (Janzen et al. 2020).

Following elevation, the second largest effect we found is that transforming natural habitats into pastures appears to change the scale at which different biophysical factors determine community structure, shifting from local to landscape‐scale. In natural habitats, local‐scale canopy cover is the second most important driver of bird and dung beetle turnover for rare and widespread species (but the first one in widespread dung beetles ζ_4_, Figure 3). By contrast, in transformed habitats, geographical distance and landscape‐scale forest cover emerge as a strong force of community turnover after elevation. As a result, anthropogenic habitat transformation restructures biodiversity in such a way that communities may increasingly depend on dispersal ability to move across the landscape. One potential strategy to counter this trend is the retention and expansion of high forest cover (> 40%) to ensure sufficient landscape connectivity, enabling species dispersal and reducing extinction risks, especially in response to the hostile conditions of pasturelands (Banks‐Leite et al. 2014; Arroyo‐Rodríguez et al. 2020). Thus, the reduced habitat quality and connectivity in transformed landscapes make dispersal highly relevant for communities.

### 4.2 Land‐Use Drives Both Biotic Homogenization and Heterogenization

The zeta diversity framework allows the decoupling of land‐use change effects on the spectrum of rare‐to‐widespread species to detect co‐occurring signals of both biotic homogenization (bird communities) and biotic heterogenization (dung beetles) as a product of habitat transformation (McKinney and Lockwood 1999; Newbold et al. 2018). However, orchid communities lack any set of ‘winner’ species or increased differentiation after deforestation. These patterns are obscured when considering only beta‐diversity, underscoring the value of using zeta diversity to understand community‐level responses to habitat changes.

Biotic homogenization of bird communities in transformed habitats might be explained by the loss of sensitive species, with spatially proximal bird communities having twice the amount of more widespread species than natural habitats potentially driven by environmental filtering increasing more disturbance‐tolerant ‘widespread’ species (McKinney and Lockwood 1999; Tabarelli et al. 2012; Püttker et al. 2015; Newbold et al. 2018). This is also supported by the decrease in explanatory power of MS‐GDM at high zeta orders in transformed habitats, suggesting that communities of widespread bird species are more stochastically structured. Whilst some studies have concluded that homogenization in bird communities was driven by the changing distributional patterns of species with large ranges (Gilroy et al. 2014b; Newbold et al. 2018), we detected concurrent decrease in *α* diversity. At low elevations, local extirpation of rare species led to overall homogenisation, and this is likely to be of interest to conservationists, given the loss of species that were locally rare and did not colonize new areas within the locale to offset extirpations. Decreased dissimilarity contributes to large‐scale homogenization, which poses the idea that some species are colonizing new elevations (e.g., eastern meadowlark *Sturnella magna* , Andean lapwing *Vanellus resplendens* ) and potentially displacing natural occurring widespread species (Gilroy et al. 2014b). This highlights the importance of locally widespread species in driving regional patterns of community structure (Lennon et al. 2004; McGeoch et al. 2019).

In contrast, land‐use change in dung beetles provokes biotic heterogenization, primarily driven by the loss (subtraction) of widespread beetle species in natural habitats. Our findings show that natural habitats retain four times more widespread dung beetle species than pasturelands. Likewise, variance explained declines more steeply in transformed habitats, suggesting highly fragmented communities, with fewer species consistently shared across sites. Dung beetles exhibit rapid species turnover at lower zeta orders in pastures, indicating a more immediate response to habitat loss. However, dung beetles display a progressive loss of community structure rather than a sharp threshold effect. This pattern is likely a product of the historical connectivity of the montane biomes in Colombia's eastern Cordillera which facilitated species dispersal (Escobar et al. 2005). However, widespread species (e.g., *Deltochilum hypponum*, a telecoprid, copronecrophagous beetle found across 36 plots in natural but absent in transformed habitats) are highly sensitive to forest loss due to soil compaction in pastures and can quickly experience range declines (González FA et al. 2009; Sweeney and Jarzyna 2022). In comparison, pasture species such as *Andinocopris achamas*, *Uroxys coarctatus*, and *Onthophagus curvicornis* are relatively rare in natural habitats. This may result from species adapted to exploit spatially contiguous natural conditions in which connectivity is disrupted by human disturbance (Noriega et al. 2021). For instance, the loss of canopy cover increases temperatures that reduce dung beetle diversity (Halffter and Arellano 2002). Additionally, dung and carrion, critical resources for dung beetles, are more abundant in connected natural habitats where mammal movement is less restricted (Horgan 2005; Nichols et al. 2009).

Finally, we provide evidence of orchid communities' high sensitivity to land‐use change as they are the most negatively impacted taxon in our study. We also support the notion of a unique set of species structuring Andean orchid communities, with spatial rarity at their core (Parra‐Sanchez et al. 2023b), which explains the similarity in patterns of species turnover across the whole range of rare to widespread species. Although turnover is high in both natural and transformed habitats, pastures are hostile for over 90% of species likely due to the lack of large old‐growth trees for forest‐dependant epiphytes impacting stochastic processes of orchid establishment (Parra‐Sanchez and Banks‐Leite 2022; Ospina‐Calderón et al. 2023). The transformed habitat might act as a barrier due to altered microclimatic conditions, reduced tree density, and fragmented landscapes, which may limit the dispersal of epiphytic orchids (Parra‐Sanchez et al. 2023a). A niche‐based process may also be acting at a finer spatial scale. For instance, orchid seeds cannot tolerate drought in pastures (Olaya‐Arenas et al. 2011; Mondragon et al. 2014; Ospina‐Calderón et al. 2023) and orchids have very specific interactions with pollinators (Kindlmann et al. 2014; Ackerman et al. 2023), and the mycorrhizal fungi necessary for germination and survival that might be extirpated in isolated trees in pasturelands (Mccormick and Jacquemyn 2014; Romero‐Salazar et al. 2022).

## 5 Conclusion

Habitat transformation in the Andes not only results in species‐poor communities, but also fundamentally reshapes the biogeographical forces that govern community assembly across taxa (such as community‐elevation relations). The zeta‐diversity framework offers a complementary approach for exploring such patterns, whilst addressing two key issues of beta diversity: the challenge of interpreting patterns across large numbers of communities; and the bias introduced by averaging non‐independent pairwise values (Jost 2007; Hui and McGeoch 2014). Worldwide projections of habitat transformation and deforestation predict substantial increases in the extinction risk of forest‐dependent vertebrate and plant species (Betts et al. 2019). In the Andes, which cover less than 0.6% of the Earth's land area (Mittermeier et al. 2011) but host over 10% of global plant species and more than 3380 vertebrate species (Myers et al. 2000; Pérez‐Escobar et al. 2022), our results show that the effect of local deforestation is doubly devastating. Even relatively local reductions in natural habitats can significantly raise the extinction risk for communities containing both range‐restricted and widespread species (Rahbek et al. 2019). Maintaining natural habitats and enhancing connectivity to protect and restore altitudinal corridors must be priorities to help retain biogeographical patterns and support climate change adaptation strategies.

## Author Contributions

**Edicson Parra‐Sanchez:** conceptualization, data curation, formal analysis, investigation, methodology, software, validation, visualization, writing – original draft, writing – review and editing. **Guillaume Latombe:** conceptualization, formal analysis, investigation, methodology, software, validation, visualization, writing – review and editing. **Simon C. Mills:** data curation, validation, writing – review and editing. **Jacob B. Socolar:** data curation, writing – review and editing. **Felicity A. Edwards:** data curation, validation, writing – review and editing. **Diego Martinez‐Revelo:** data curation, validation, writing – review and editing. **Oscar A. Perez‐Escobar:** data curation, writing – review and editing. **Robert W. Davies:** data curation, writing – review and editing. **Christopher G. Bousfield:** data curation, validation, writing – review and editing. **Gianluca R. Cerullo:** data curation, validation, writing – review and editing. **Jose Manuel Ochoa‐Quintero:** project administration, resources, writing – review and editing. **Torbjørn Haugaasen:** funding acquisition, project administration, resources, writing – review and editing. **Jos Barlow:** validation, writing – review and editing. **Robert P. Freckleton:** supervision, validation, writing – review and editing. **David P. Edwards:** funding acquisition, investigation, methodology, project administration, resources, supervision, validation, writing – original draft, writing – review and editing.

## Conflicts of Interest

The authors declare no conflicts of interest.

## Supporting information


Data S1.

## Data Availability

The data and code supporting the results of this study can be accessed via the following link: https://doi.org/10.6084/m9.figshare.25954063.v1. Landscape metrics were obtained using the Global Change Forest at https://data.globalforestwatch.org/maps/gfw::tree‐cover‐loss‐1/about. Disturbance data were obtained from Tropical Forest Monitoring via the EU science hub at https://forobs.jrc.ec.europa.eu/TMF/data#downloads (v1). Mean annual precipitation was extracted from CHELSA database at https://doi.org/10.1038/sdata.2017.122. Digital Elevation Model was obtained from the Alaska Satellite Facility Distributed Active Archive Center (ASF DAAC) at https://doi.org/10.5067/Z97HFCNKR6VA.
