# Supplementary material for: Tropical Land‐Use Change Disrupts Zeta‐Diversity Across Taxa
Source: Glob Chang Biol. 2025 May 14;31(5):e70245. doi: 10.1111/gcb.70245 (PMC12076182; doi:10.1111/gcb.70245)

Tropical land-use change disrupts zeta-diversity across taxa .

Supplementary material

Parra-Sanchez, E, Latombe, G., Mills, S, Edwards, F., Medina, C.A., Perez-Escobar, O., Davies, R., Bousfield, C., Cerullo, G., Ochoa, J.M., & Edwards D.P.

Supplementary Information: Table of contents

Supplementary Table 1 3

Supplementary Table 2 4

Supplementary Table 3 5

Supplementary Table 4 6

Supplementary Figure 1 8

Supplementary Figure 2 10

Supplementary Figure 3 12

Supplementary Figure 4 14

Supplementary Figure 5 16

Supplementary Figure 6 18

Supplementary Figure 7 20

Supplementary Figure 8 22

Supplementary Figure 9 24

Supplementary Figure 10 26

Supplementary Figure 11 28

Supplementary Figure 12 30

Supplementary Figure 13 32

Forest non-forest map filtering criteria

Landscape metrics were measured using the 30-m resolution "global change forest map" from 2018 (hereafter termed GCF; Hansen et al., 2013). We defined a threshold of 50% forest cover (non-forest <=50, and >50% as forest), after validating 334 field points within and outside forest fragments to five forest cover thresholds (40%, 50%, 60%, 70%, 90%). To validate our threshold of 50% forest cover, we transformed all pixels into binary forest/non-forest maps for each threshold percentage, then we extracted the cover type of each of our 334 validation points, thus each point would be either forest or non-forest. Finally, we compared the proportion of points per threshold that matches our validation points and the threshold with the highest proportion of points was selected (Table S2).

Supplementary Table 1. Evaluation of forest/non-forest map´s forest cover thresholds.

Forest cover	Forest	Non-forests	Matching percentage
40%	133	122	76,3
50%	160	144	91,0
60%	161	117	83,2
70%	164	84	74,3
90%	167	72	71,6

Supplementary Table 2. Generalised mixed model output across bird, dung beetles, and orchid communities. Poisson mixed models were estimated using ML and Nelder-Mead optimizer and included patch as random effect (formula: ~1 |patch). Standardized parameters were obtained by fitting the model on a standardized version of the dataset. Profile Likelihood Confidence Intervals 95% (CIs).

	**Birds**		**Dung beetles**		**Orchids**	
	**Estimates**	**CI**	**Estimates**	**CI**	**Estimates**	**CI**
**Intercept**	2.69***	2.56 – 2.81	1.54***	1.179 – 6.53	1.65***	1.40 – 1.86
**Transformed**	-0.60***	-0.77 –-0.43	-2.04***	-2.74 – -1.41	-0.99***	-1.47 – -0.52
**N**	325		177		179	
**R2 (marginal)**	0.3		0.43		0.22	
**R2 (total)**	0.69		0.79		0.66	
**Likelihood ratio test (*vs null model*)**	<0.005		<0.005		<0.005	

Supplementary Table 3. Zeta diversity decline calculations across bird, dung beetle, and orchid communities in the Colombian Andes. Zeta diversity was calculated with increases in zeta order (number of combinations of sites), indicating the rate of change in species turnover from rare to common species. The zeta decline used the "all combinations" (ALL) spatial arrangement where combinations of sites independently from their geographical position. In contrast, the "nearest neighbours non-directional" (NON) sampling scheme combines sites based on a nearest-neighbour approach based on their spatial coordinates. Confidential intervals (2.5%, 97.5%) and model AIC.

	Habitat	Method	Parametric form	Intercept	CI 2.5%	CI 97.5%	AIC
Birds	Forest	ALL	Exponential	0.9721	0.574	1.37	4.625
Birds	Forest	ALL	power-law	1.243	1.181	1.306	-34.367
Birds	Forest	NON	Exponential	1.164	1.045	1.283	-19.464
Birds	Forest	NON	power-law	1.263	1.165	1.361	-25.463
Birds	Transformed	ALL	Exponential	0.802	0.531	1.072	-3.094
Birds	Transformed	ALL	power-law	0.999	0.866	1.134	-19.153
Birds	Transformed	NON	Exponential	0.833	0.65	1.016	-10.914
Birds	Transformed	NON	power-law	0.941	0.889	0.992	-38.267
Dung beetles	Forest	ALL	Exponential	0.836	0.659	1.013	-11.638
Dung beetles	Forest	ALL	power-law	1.031	0.714	1.348	-1.939
Dung beetles	Forest	NON	Exponential	0.835	0.794	0.876	-40.765
Dung beetles	Forest	NON	power-law	0.882	0.748	1.017	-19.11
Dung beetles	Transformed	ALL	Exponential	0.875	0.785	0.964	-25.153
Dung beetles	Transformed	ALL	power-law	1.15	0.235	2.066	19.259
Dung beetles	Transformed	NON	Exponential	0.451	0.279	0.622	-12.195
Dung beetles	Transformed	NON	power-law	0.558	0.341	0.775	-9.545
Orchids	Forest	ALL	Exponential	1.866	1.723	2.01	-15.85
Orchids	Forest	ALL	power-law	2.438	0.471	4.404	34.551
Orchids	Forest	NON	Exponential	1.474	1.107	1.842	-4.429
Orchids	Forest	NON	power-law	1.044	0.159	1.929	6.16
Orchids	Transformed	ALL	Exponential	1.794	1.378	2.209	-3.2
Orchids	Transformed	ALL	power-law	0.819	-0.981	2.619	13.261
Orchids	Transformed	NON	Exponential	0.723	-0.361	1.808	1.932
Orchids	Transformed	NON	power-law	0.344	0.029	0.659	-5.61

Supplementary Table 4. Correlation matrix of predictors. The matrix displays Pearson correlation coefficients between pairs of predictors, with values ranging from -1 (perfect negative correlation) to +1 (perfect positive correlation). The diagonal represents the correlation of each variable with itself (r = 1). This matrix was used to assess multicollinearity among predictors prior to modelling.

**Predictors**	**Elevation**	**Canopy cover**	**Tree density**	**Fragmentation**	**Forest cover**	**Precipitation**	**Disturbance**
**Elevation**	**1**	-0.1226	-0.0238	0.0416	-0.1651	-0.1024	0.1668
**Canopy cover**	-0.1226	**1**	0.4883	-0.1113	0.3031	-0.0686	0.0418
**Tree density**	-0.0238	0.4883	**1**	-0.1295	0.2169	-0.0072	0.0739
**Fragmentation**	0.0416	-0.1113	-0.1295	**1**	-0.4223	-0.3247	-0.4064
**Forest cover**	-0.1651	0.3031	0.2169	-0.4223	**1**	0.1817	0.3705
**Precipitation**	-0.1024	-0.0686	-0.0072	-0.3247	0.1817	**1**	0.3040
**Disturbance**	0.1668	0.0418	0.0739	-0.4064	0.5705	0.3040	**1**

Land-use change alters drivers and patterns of community structure

Alpha diversity

To assess the validity of the Poisson generalized linear mixed model (GLMM), we used the DHARMa package to evaluate residual uniformity, overdispersion, homogeneity of variance, and outlier influence. The Kolmogorov-Smirnov test for uniformity indicated that residuals did not significantly deviate from the expected distribution ($D = 0.0737$, $p = 0.0585$), suggesting that the model's residuals followed a reasonable pattern. The nonparametric dispersion test confirmed that overdispersion was not present (*dispersion* = 1.0597, $p = 0.654$), supporting the appropriateness of the Poisson distribution. Additionally, the quantile test did not return any indications of heteroscedasticity, suggesting that variance was homogeneously distributed across fitted values. Finally, the outlier test revealed no extreme observations ($p = 1$), indicating that no individual data points unduly influenced the model. These diagnostics confirm that the Poisson GLMM assumptions were met, validating its use for analysing species richness in relation to land use.

Supplementary Figure 1. MS-GDM splines for bird communities in natural habitats from 50 iterations (Binomial family (link="log") for each order of zeta and each predictor, using a random sample of 1000 site combinations for each MS-GDM. The variability between the 50 replicates provides an estimate of confidence for each predictor.

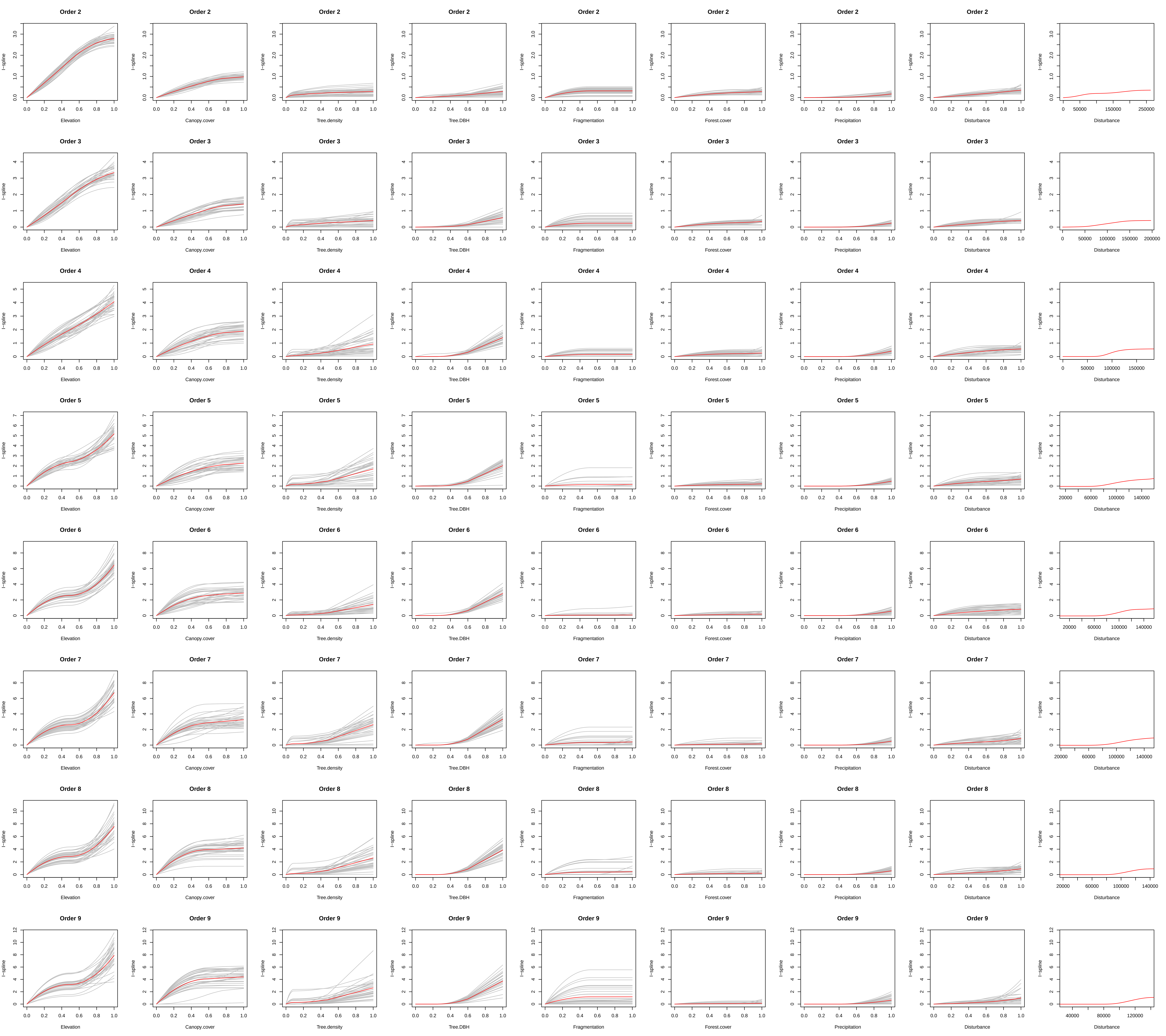

Order 2
Order 3
Order 4
Order 5
Order 6
Order 7
Order 8
Order 9
I−spline
Elevation
Canopy.cover
Tree.density
Tree.DBH
Fragmentation
Forest.cover
Precipitation
Disturbance

Supplementary Figure 2. MS-GDM splines for bird communities in transformed habitats from 50 iterations (Binomial family (link="log") for each order of zeta and predictor, using a random sample of 1000 site combinations for each MS-GDM. The variability between the 50 replicates provides an estimate of confidence for each predictor.

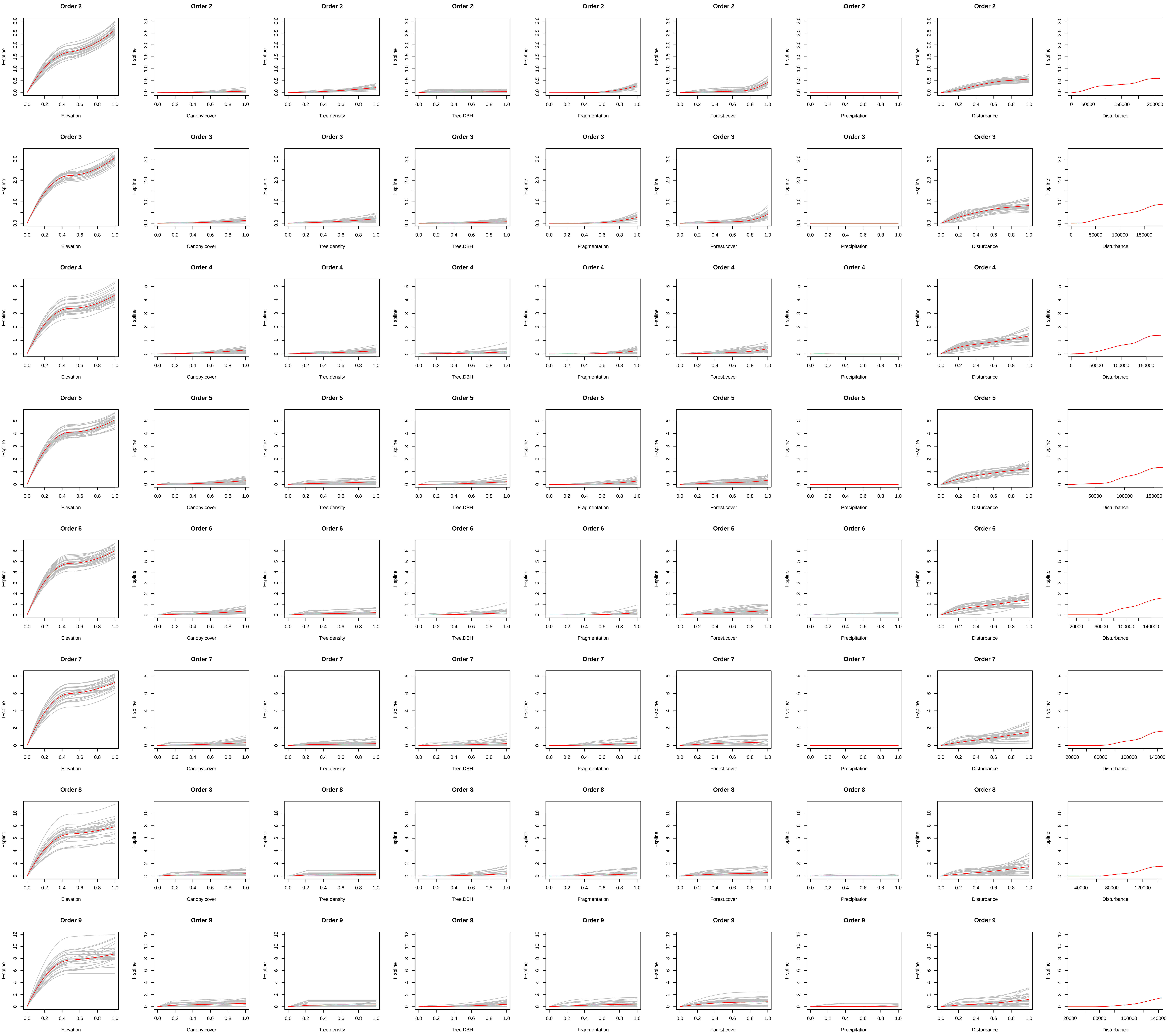

Order 2
Order 3
Order 4
Order 5
Order 6
Order 7
Order 8
Order 9
I−spline
Elevation
Canopy.cover
Tree.density
Tree.DBH
Fragmentation
Forest.cover
Precipitation
Disturbance

Supplementary Figure 3. MS-GDM median *i*-splines for bird communities in natural habitats from 50 iterations (Binomial family (link="log") for each order of zeta, using a random sample of 1000 site combinations for each MS-GDM. The variability between the 50 replicates provides an estimate of confidence for each predictor.

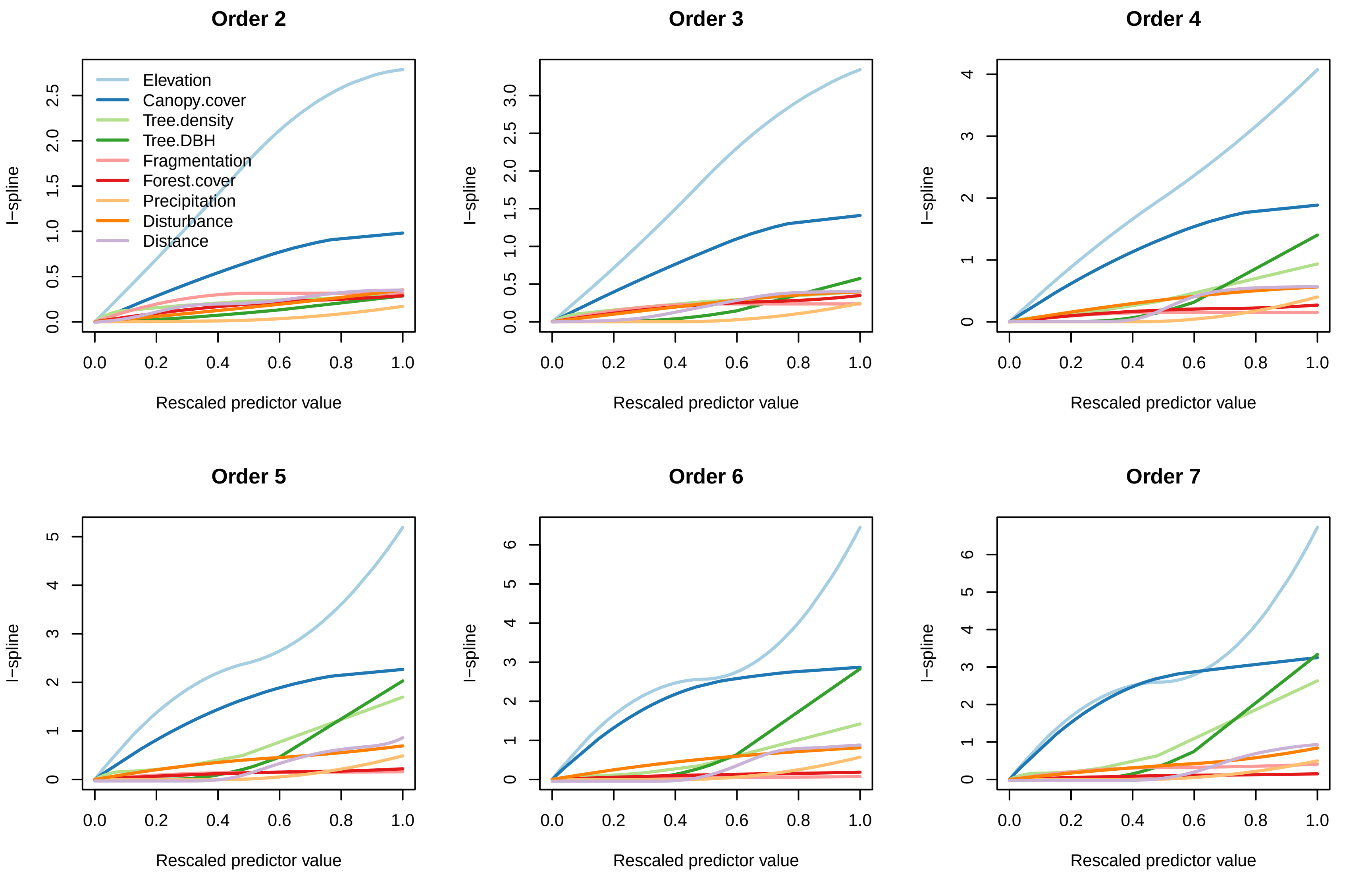

Order 2
Order 3
Order 4
Order 5
Order 6
Order 7
Elevation
Canopy.cover
Tree.density
Tree.DBH
Fragmentation
Forest.cover
Precipitation
Disturbance
Distance
I−spline
Rescaled predictor value

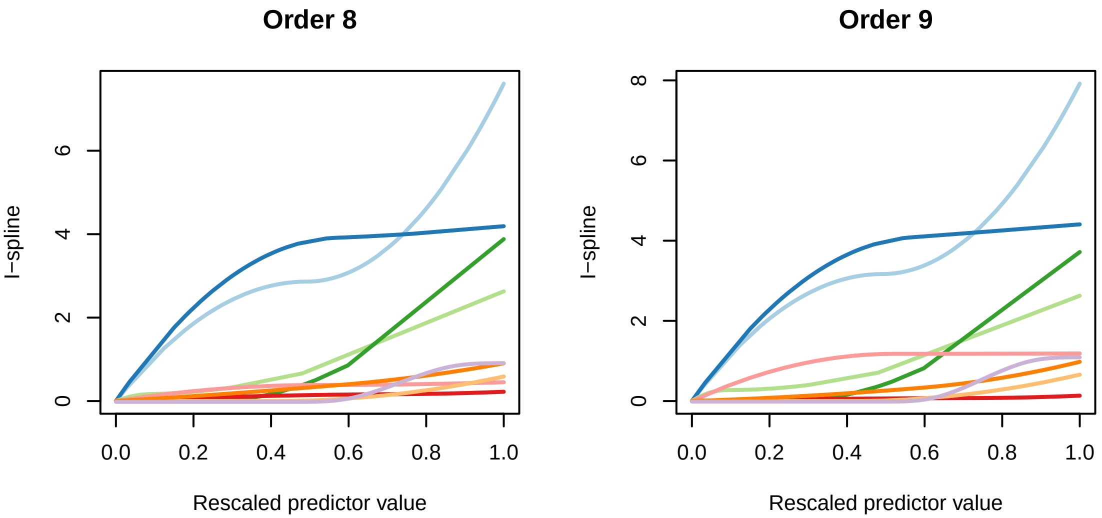
Order 8
Order 9
I−spline
Rescaled predictor value
0
2
4
6
8
0.0
0.2
0.4
0.6
0.8
1.0

Supplementary Figure 4. MS-GDM median *i*-splines for bird communities in transformed habitats from 50 iterations (Binomial family [link="log"]) for each order of zeta, using a random sample of 1000 site combinations for each MS-GDM. The variability between the 50 replicates provides an estimate of confidence for each predictor.

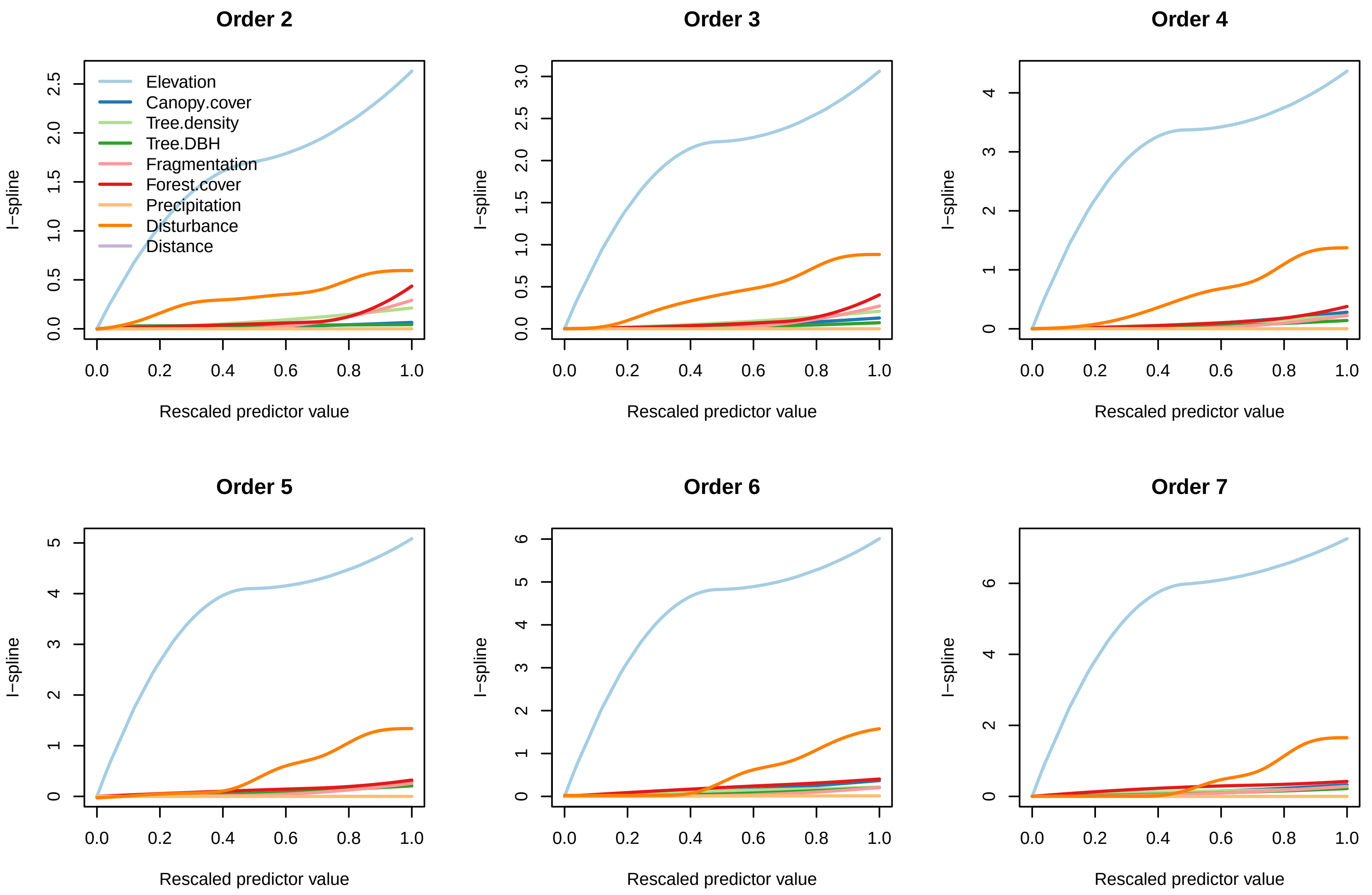

Order 2
Order 3
Order 4
Order 5
Order 6
Order 7
Elevation
Canopy.cover
Tree.density
Tree.DBH
Fragmentation
Forest.cover
Precipitation
Disturbance
Distance
I−spline
Rescaled predictor value

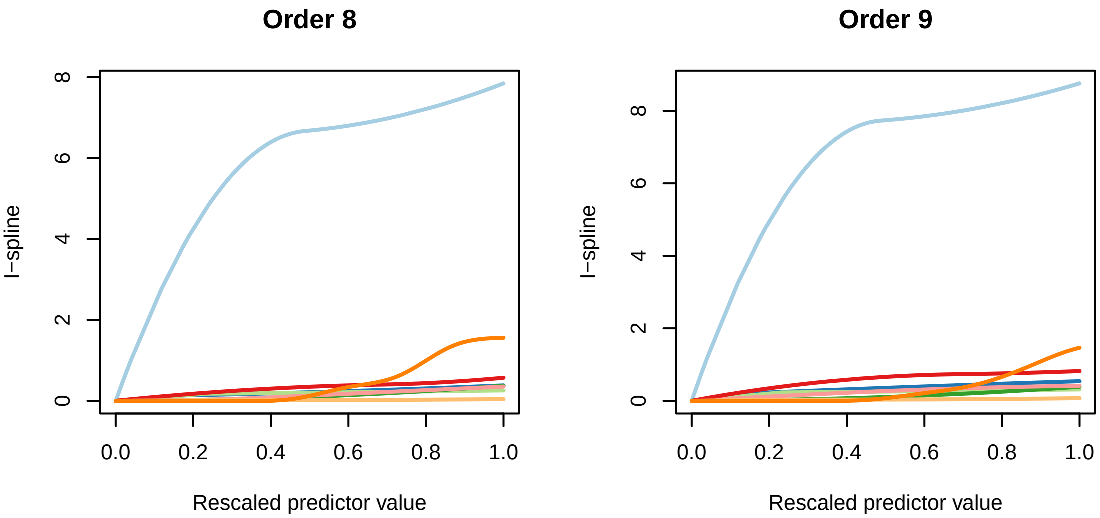

Order 8
Order 9
I−spline
Rescaled predictor value
0
2
4
6
8
0.0
0.2
0.4
0.6
0.8
1.0

Supplementary Figure 5. MS-GDM splines for dung beetle communities in natural habitats from 50 iterations (Binomial family (link="log") for each order of zeta and each predictor, using a random sample of 1000 site combinations for each MS-GDM. The variability between the 50 replicates provides an estimate of confidence for each predictor.

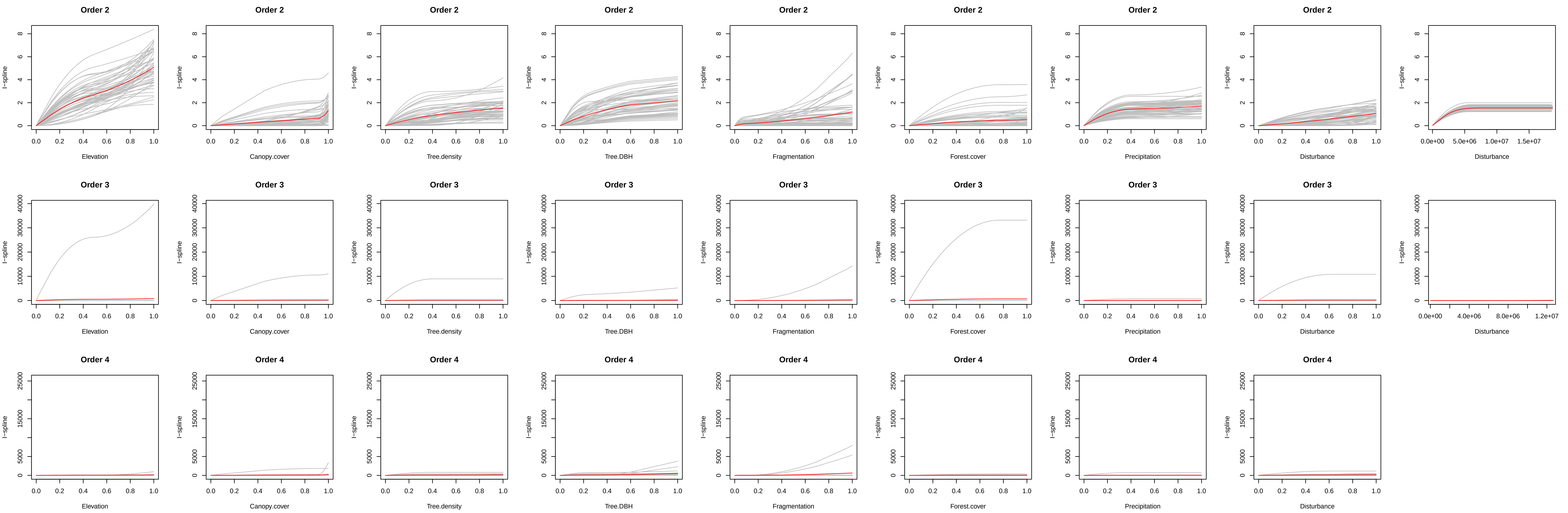
Order 2
Order 3
Order 4
I−spline
Elevation
Canopy.cover
Tree.density
Tree.DBH
Fragmentation
Forest.cover
Precipitation
Disturbance

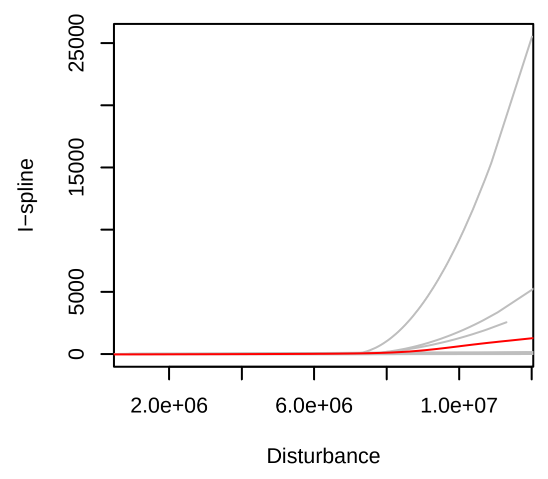
I−spline
25000
15000
5000
0
2.0e+06
6.0e+06
1.0e+07
Disturbance

Supplementary Figure 6. MS-GDM splines for dung beetle communities in transformed habitats from 50 iterations (Binomial family (link="log") for each order of zeta and predictor, using a random sample of 1000 site combinations for each MS-GDM. The variability between the 50 replicates provides an estimate of confidence for each predictor.

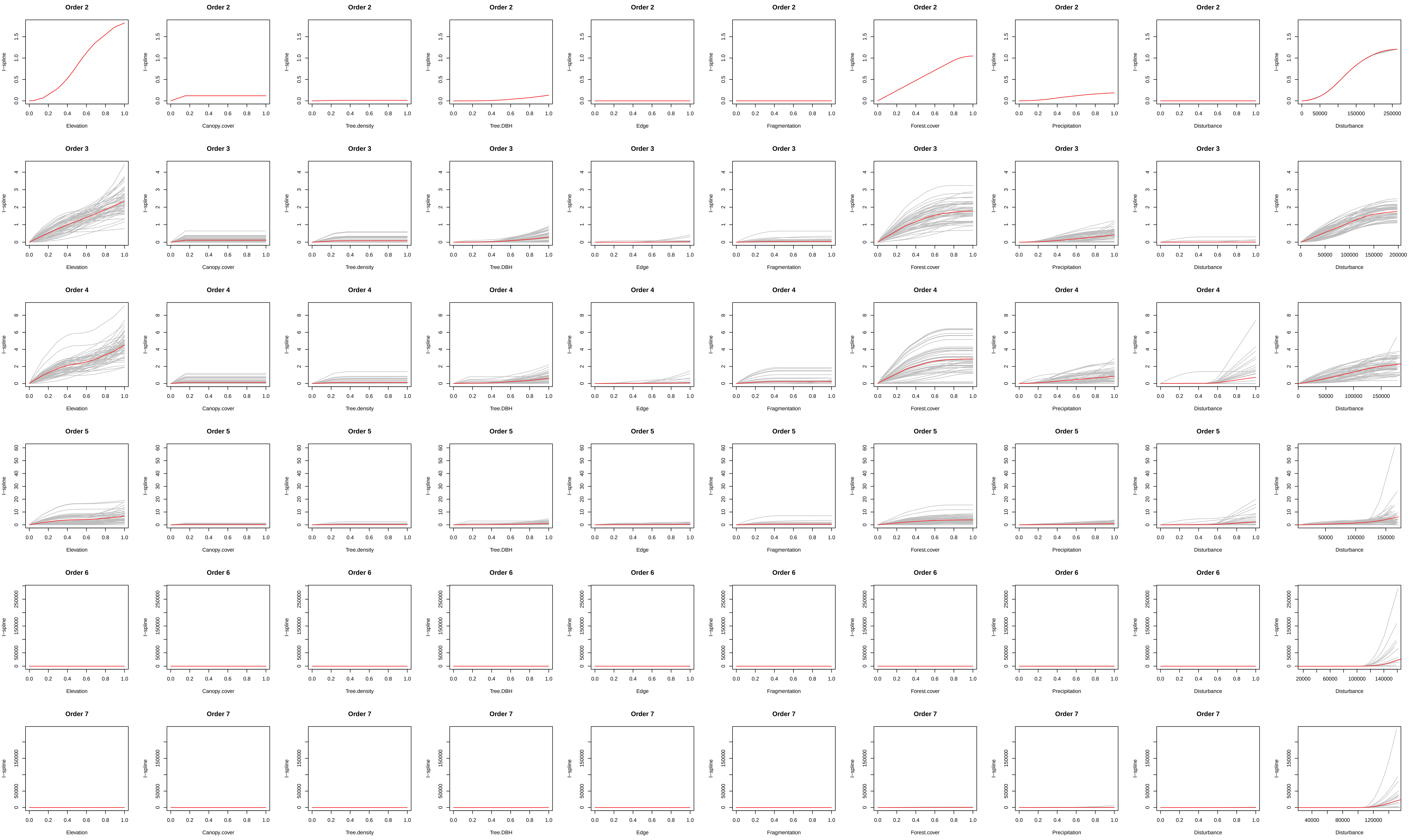

Order 2
Order 3
Order 4
Order 5
Order 6
Order 7
I−spline
Elevation
Canopy.cover
Tree.density
Tree.DBH
Edge
Fragmentation
Forest.cover
Precipitation
Disturbance

Supplementary Figure 7. MS-GDM median *i*-splines for dung beetle communities in natural habitats from 50 iterations (Binomial family (link="log") for each order of zeta, using a random sample of 1000 site combinations for each MS-GDM. The variability between the 50 replicates provides an estimate of confidence for each predictor.

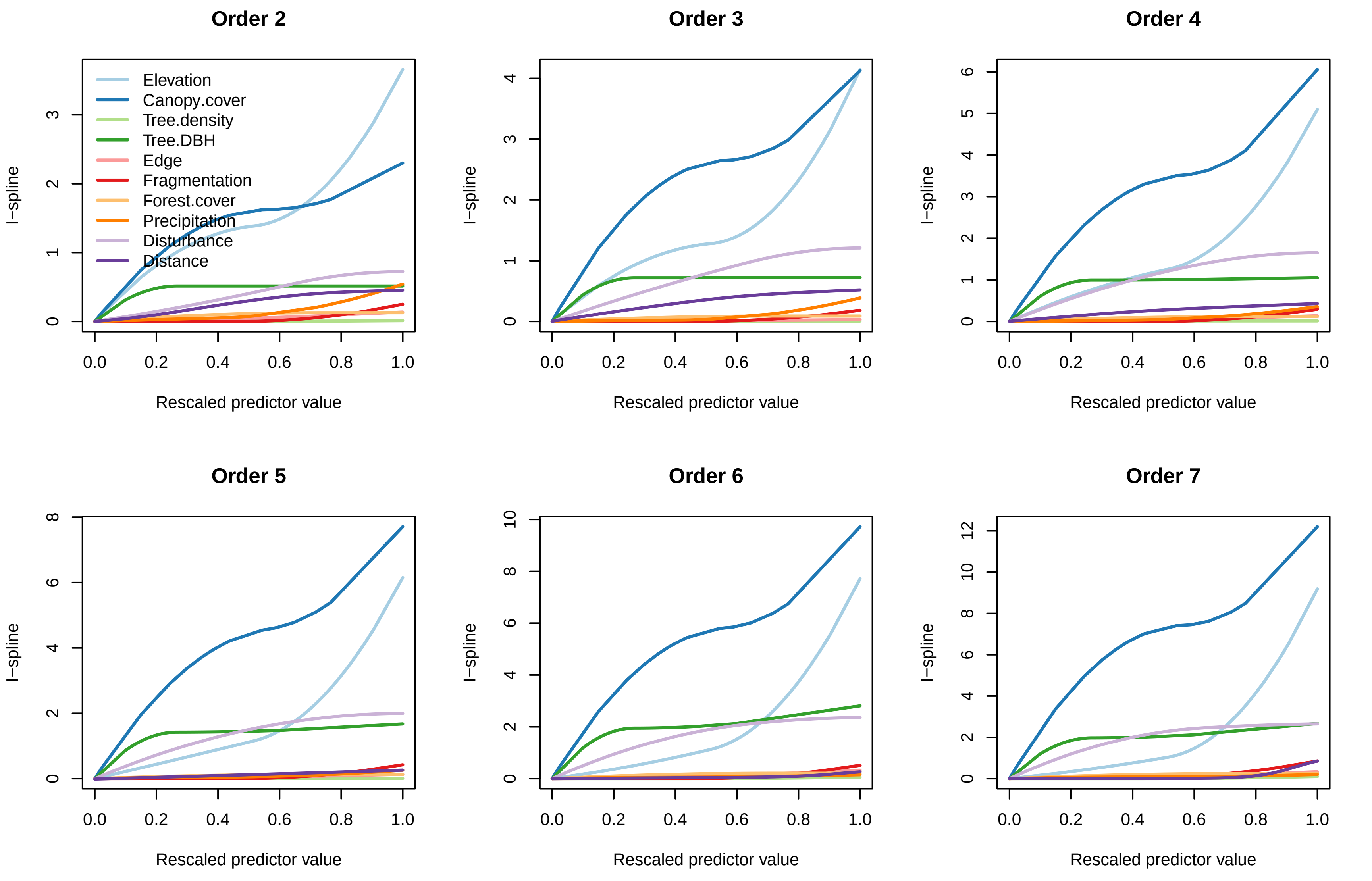
Order 2
Order 3
Order 4
Order 5
Order 6
Order 7
Elevation
Canopy.cover
Tree.density
Tree.DBH
Edge
Fragmentation
Forest.cover
Precipitation
Disturbance
Distance
I−spline
Rescaled predictor value

Supplementary Figure 8. MS-GDM median *i*-splines for dung beetle communities in transformed habitats from 50 iterations (Binomial family [link="log"]) for each order of zeta, using a random sample of 1000 site combinations for each MS-GDM. The variability between the 50 replicates provides an estimate of confidence for each predictor.

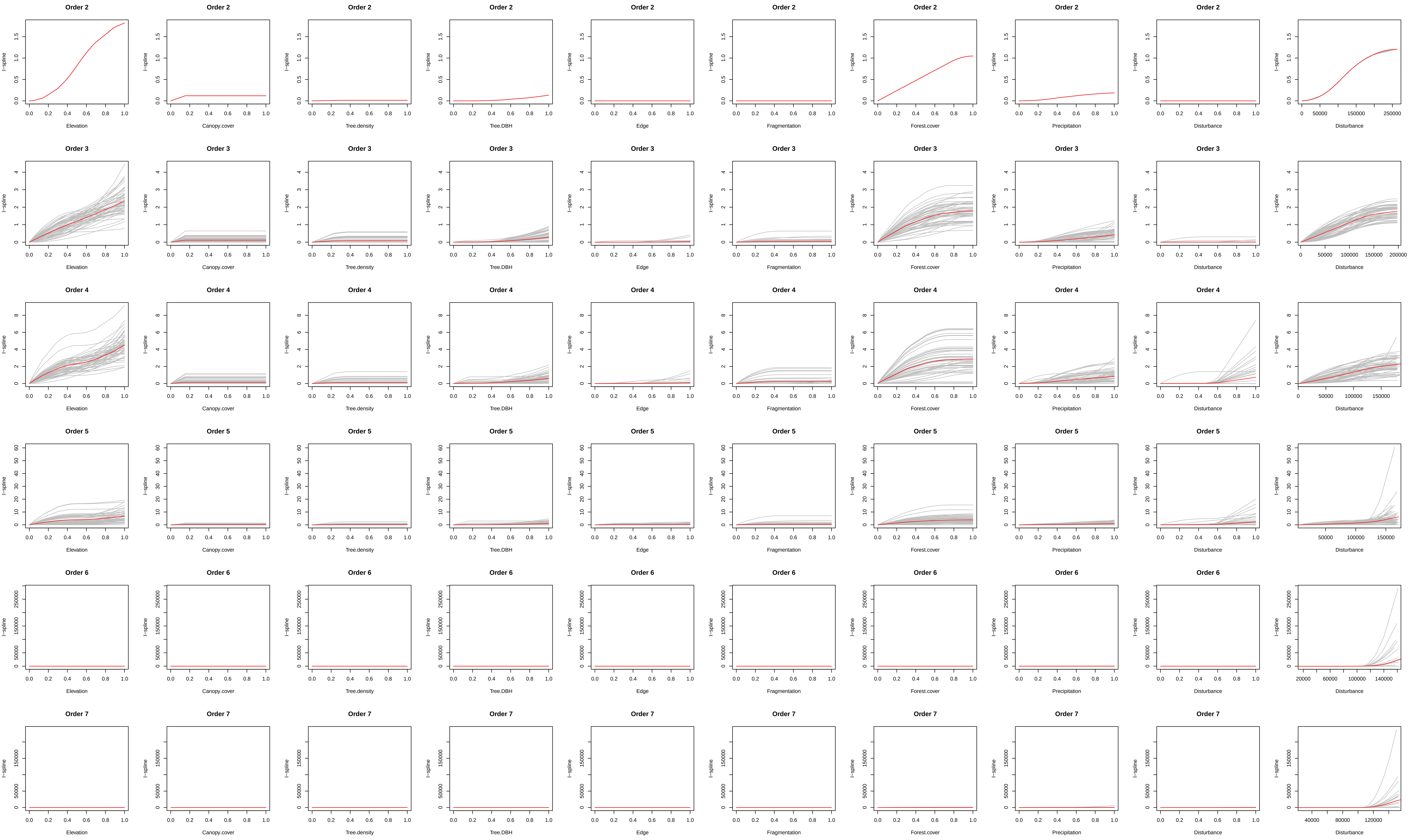
Order 2
Order 3
Order 4
Order 5
Order 6
Order 7
I−spline
Elevation
Canopy.cover
Tree.density
Tree.DBH
Edge
Fragmentation
Forest.cover
Precipitation
Disturbance

Supplementary Figure 9. MS-GDM splines for orchid communities in natural habitats from 50 iterations (Binomial family (link="log") for each order of zeta and each predictor, using a random sample of 1000 site combinations for each MS-GDM. The variability between the 50 replicates provides an estimate of confidence for each predictor.

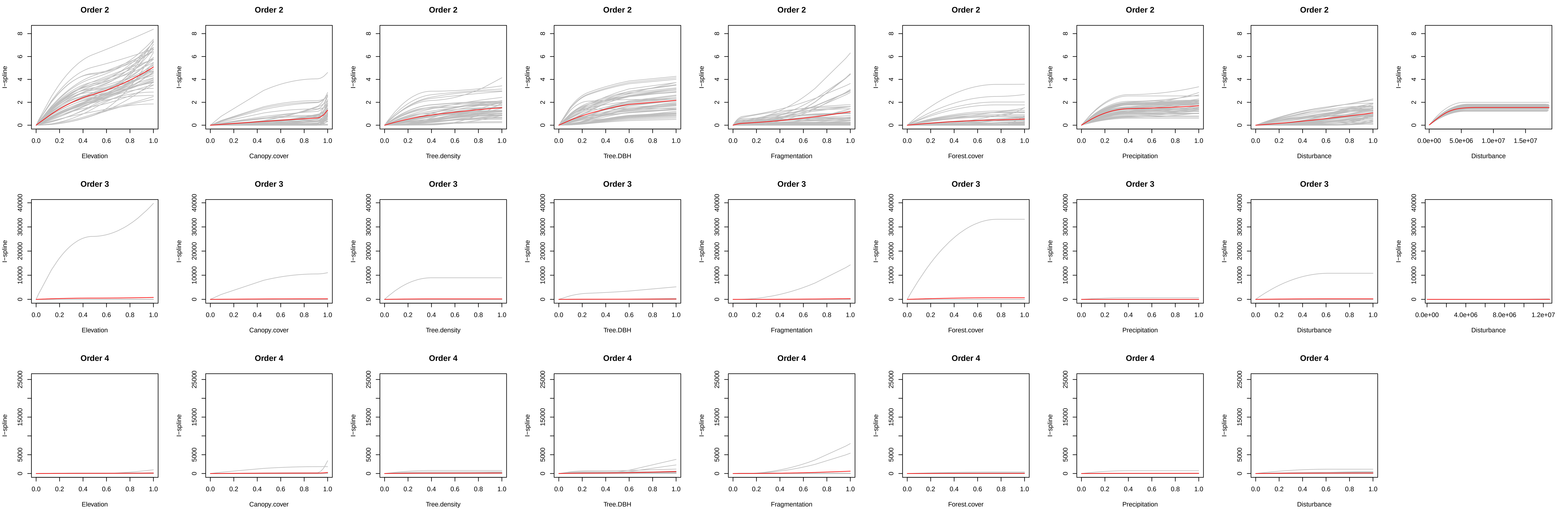

Order 2
Order 3
Order 4
I−spline
Elevation
Canopy.cover
Tree.density
Tree.DBH
Fragmentation
Forest.cover
Precipitation
Disturbance
0.0 0.2 0.4 0.6 0.8 1.0
0 2 4 6 8
0 10000 20000 30000 40000
0 5000 15000 25000
0.0e+00 5.0e+06 1.0e+07 1.5e+07
0.0e+00 4.0e+06 8.0e+06 1.2e+07

Supplementary Figure 10. MS-GDM splines for orchid communities in transformed habitats from 50 iterations (Binomial family (link="log") for each order of zeta and predictor, using a random sample of 1000 site combinations for each MS-GDM. The variability between the 50 replicates provides an estimate of confidence for each predictor.

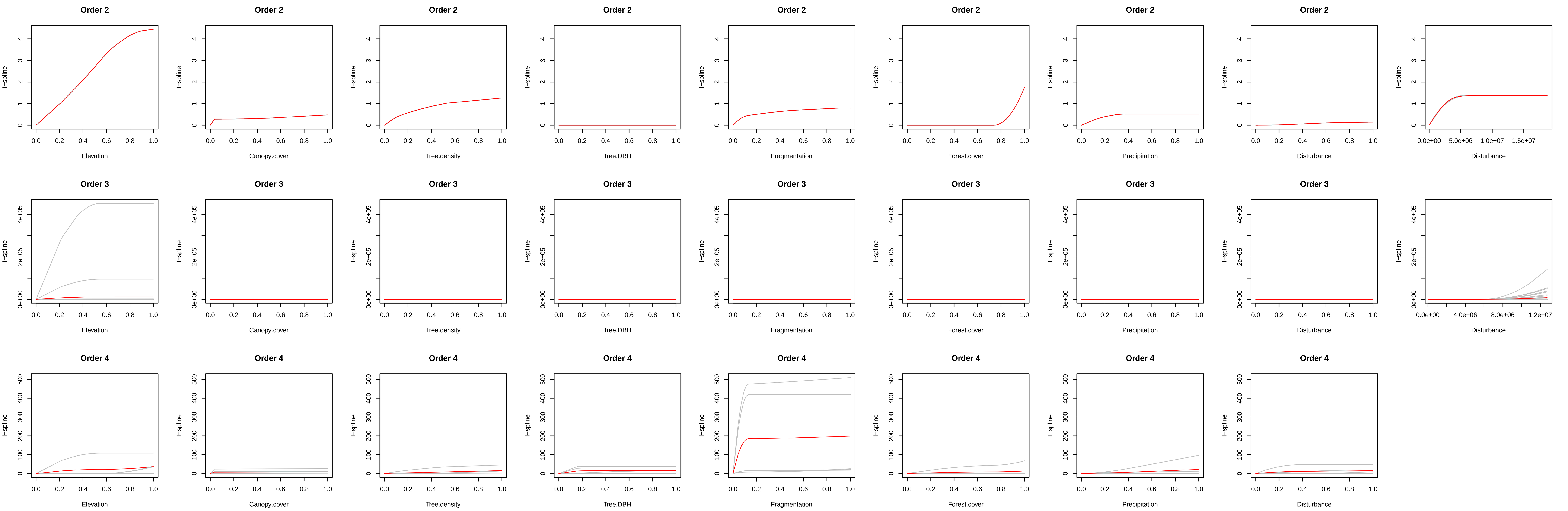
Order 2
Order 3
Order 4
I−spline
Elevation
Canopy.cover
Tree.density
Tree.DBH
Fragmentation
Forest.cover
Precipitation
Disturbance

Supplementary Figure 11. MS-GDM median *i*-splines for orchid communities in natural habitats from 50 iterations (Binomial family (link="log") for each order of zeta, using a random sample of 1000 site combinations for each MS-GDM. The variability between the 50 replicates provides an estimate of confidence for each predictor.

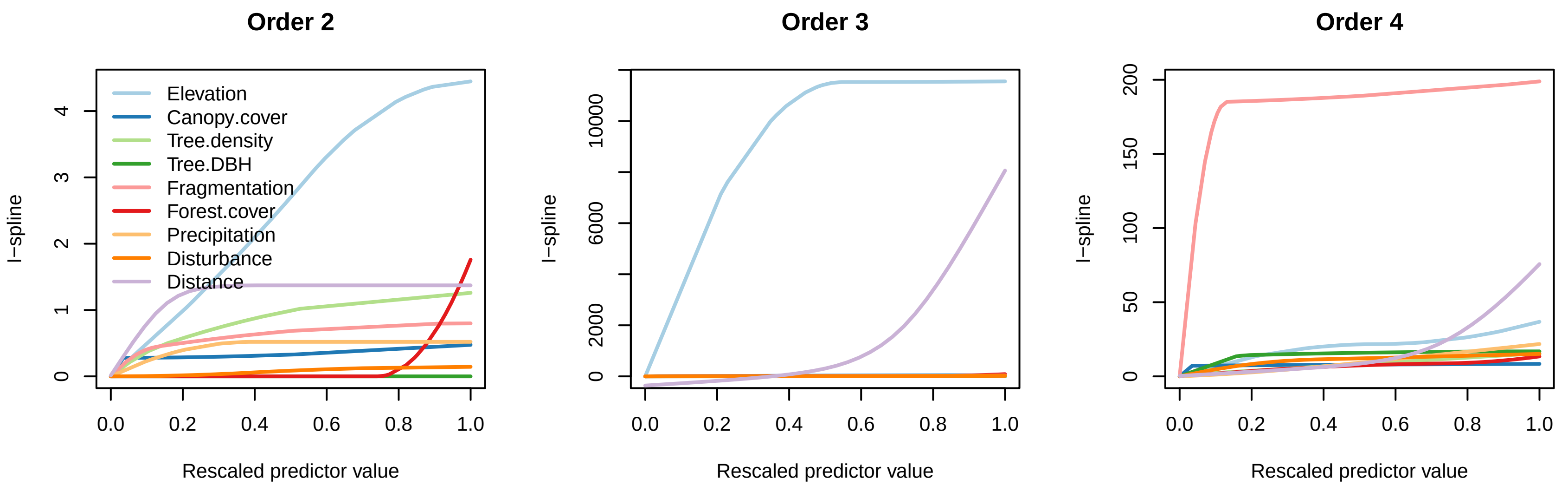
Order 2
Order 3
Order 4
Elevation
Canopy.cover
Tree.density
Tree.DBH
Fragmentation
Forest.cover
Precipitation
Disturbance
Distance
I−spline
Rescaled predictor value
0.0
0.2
0.4
0.6
0.8
1.0
0
1
2
3
4
2000
6000
10000
50
100
150
200

Supplementary Figure 12. MS-GDM median *i*-splines for orchid communities in transformed habitats from 50 iterations (Binomial family [link="log"]) for each order of zeta, using a random sample of 1000 site combinations for each MS-GDM. The variability between the 50 replicates provides an estimate of confidence for each predictor.

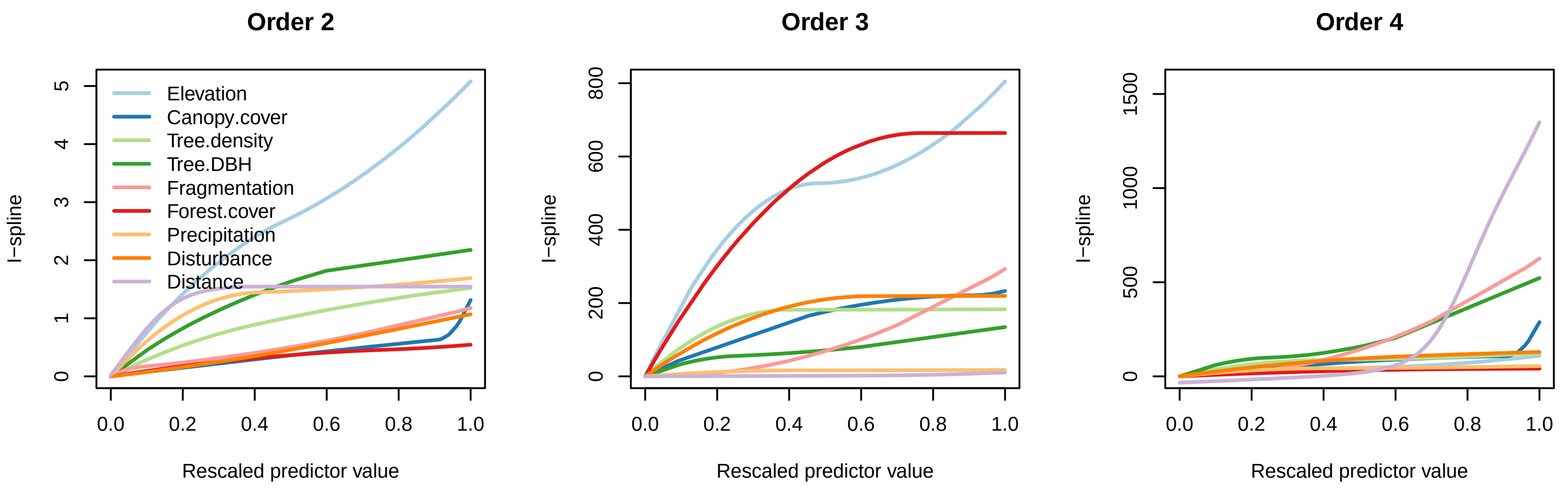

Order 2
Order 3
Order 4
I−spline
Rescaled predictor value
Elevation
Canopy.cover
Tree.density
Tree.DBH
Fragmentation
Forest.cover
Precipitation
Disturbance
Distance

Supplementary Figure 13. The variance explained for each model (i.e. for each replicate) from Pearson r^2 between the observed and the predicted zeta values. Results are presented as the full range of variance of zeta-values and the mean and standard deviation at each zeta order. The variability between the 50 replicates provides an estimate of confidence for each predictor.

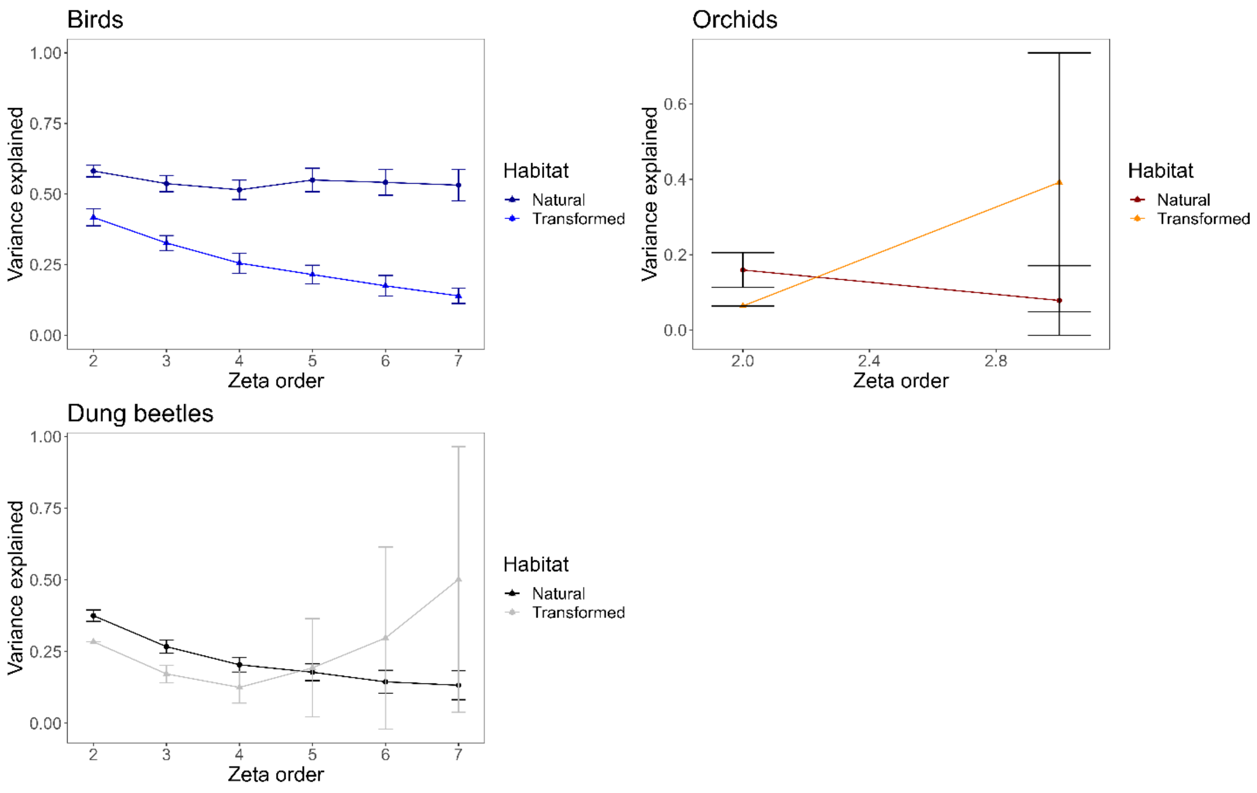

Supplement: Supplementary file 1 — Data S1. [file GCB-31-e70245-s001.zip › gcb70245-sup-0003-Supinfo02.pdf]
