# Supplementary material for: Tropical Land‐Use Change Disrupts Zeta‐Diversity Across Taxa
Source: Glob Chang Biol. 2025 May 14;31(5):e70245. doi: 10.1111/gcb.70245 (PMC12076182; doi:10.1111/gcb.70245)
Supplement: Supplementary file 1 — Data S1. [file GCB-31-e70245-s001.zip › gcb70245-sup-0001-Appendix1.zip!/SM-2-MS_birds_forest.html]

table output


MS-GDM Coefficients for bird communities in natural habitats from 50 iterations (Binomial family (link=log) for each order of zeta and each predictor, using a random sample of 1000 site combinations for each MS-GDM. Here are the 50 replicates for each predictor, AIC, and deviance.

| (Intercept) | Elevation1 | Elevation2 | Elevation3 | Canopy.cover1 | Canopy.cover2 | Canopy.cover3 | Tree.density1 | Tree.density2 | Tree.density3 | Fragmentation1 | Fragmentation2 | Fragmentation3 | Forest.cover1 | Forest.cover2 | Forest.cover3 | Precipitation1 | Precipitation2 | Precipitation3 | Disturbance1 | Disturbance2 | Disturbance3 | distance1 | distance2 | distance3 | Rep | Group | AIC | Residual\_Deviance |
| --- | --- | --- | --- | --- | --- | --- | --- | --- | --- | --- | --- | --- | --- | --- | --- | --- | --- | --- | --- | --- | --- | --- | --- | --- | --- | --- | --- | --- |
| 0.0000000 | -0.8450396 | -1.8702756 | -0.5458168 | 0.0000000 | -0.4791264 | -0.3634412 | -0.0839282 | -0.4831778 | 0.0000000 | -0.1586938 | -0.1615264 | -0.3233182 | 0.0000000 | 0.0000000 | 0.0000000 | -0.1919029 | -0.1513969 | 0.0000000 | 0.0000000 | -0.2765196 | -0.1860315 | -0.2249947 | 0.0000000 | -0.2163151 | 1 | 1 | 173.92256 | 118.673913 |
| 0.0000000 | -0.7691708 | -2.2153593 | -0.4307274 | 0.0000000 | -1.2308542 | -0.1736211 | 0.0000000 | -0.4449895 | 0.0000000 | 0.0000000 | -0.4829369 | -0.7051306 | 0.0000000 | 0.0000000 | 0.0000000 | -0.3027606 | -0.1265978 | 0.0000000 | 0.0000000 | 0.0000000 | -0.3579370 | -0.0923440 | -0.3123868 | -0.1769325 | 1 | 2 | 121.76649 | 68.684043 |
| 0.0000000 | -1.1851624 | -1.7848789 | -1.2135988 | 0.0000000 | -1.2404333 | -0.4796388 | 0.0000000 | -0.2535851 | -0.5576512 | -0.0398732 | 0.0000000 | -1.1334362 | -1.1097405 | 0.0000000 | 0.0000000 | -0.4890065 | 0.0000000 | 0.0000000 | 0.0000000 | 0.0000000 | -0.5556168 | -0.4416169 | 0.0000000 | -0.2965412 | 1 | 3 | 102.15659 | 50.342396 |
| 0.0000000 | -2.4362789 | -0.3832781 | -3.7751046 | 0.0000000 | -1.5142089 | -0.1102284 | 0.0000000 | 0.0000000 | -0.1400104 | 0.0000000 | -0.6902487 | -3.5004208 | 0.0000000 | 0.0000000 | 0.0000000 | -0.0541714 | 0.0000000 | 0.0000000 | 0.0000000 | 0.0000000 | -0.8249096 | -0.7436458 | -0.3104912 | 0.0000000 | 1 | 4 | 77.76237 | 26.637285 |
| 0.0000000 | -2.6020184 | 0.0000000 | -3.0934028 | -5.2195408 | -0.2826201 | -0.2577336 | 0.0000000 | 0.0000000 | -0.5444668 | -0.1244721 | 0.0000000 | -6.4690377 | 0.0000000 | 0.0000000 | 0.0000000 | 0.0000000 | 0.0000000 | 0.0000000 | 0.0000000 | 0.0000000 | -0.1205799 | -0.9635638 | -0.4963027 | 0.0000000 | 1 | 5 | 66.92633 | 16.036233 |
| 0.9745613 | -2.2981395 | 0.0000000 | -3.1386108 | 0.0000000 | -4.3686221 | 0.0000000 | 0.0000000 | -0.3346632 | -0.6668559 | 0.0000000 | 0.0000000 | -4.8544574 | 0.0000000 | 0.0000000 | 0.0000000 | -0.2423828 | -0.2476758 | 0.0000000 | 0.0000000 | 0.0000000 | -0.3612477 | -1.5230683 | 0.0000000 | 0.0000000 | 1 | 6 | 65.18902 | 14.693100 |
| 0.0000000 | -1.0210666 | -1.9174274 | -0.6197397 | 0.0000000 | -0.6963486 | -0.2176733 | -0.1069396 | -0.5068498 | 0.0000000 | 0.0000000 | -0.3096865 | 0.0000000 | -0.0496933 | -0.1338026 | 0.0000000 | -0.1374694 | -0.3111829 | -0.0134576 | 0.0000000 | 0.0000000 | -0.2985381 | -0.2493790 | 0.0000000 | -0.1256559 | 2 | 1 | 170.27802 | 115.184447 |
| 0.0000000 | -1.0584939 | -2.0902701 | -0.3630571 | 0.0000000 | -1.0241792 | -0.2119304 | -0.1614860 | 0.0000000 | -0.1955073 | 0.0000000 | -0.5228065 | -0.2517855 | -0.0779793 | 0.0000000 | 0.0000000 | -0.2487850 | -0.1940737 | 0.0000000 | 0.0000000 | -0.1206413 | -0.3286127 | -0.4342377 | -0.0045942 | -0.6697881 | 2 | 2 | 127.54848 | 74.410401 |
| 0.1862261 | -1.0695131 | -2.4941731 | -0.0608762 | -0.3054948 | -2.2339937 | -0.1680837 | 0.0000000 | 0.0000000 | -0.2268903 | 0.0000000 | -1.0007013 | 0.0000000 | 0.0000000 | 0.0000000 | 0.0000000 | -0.2031752 | -0.0529090 | 0.0000000 | 0.0000000 | 0.0000000 | -0.5241192 | -0.6761388 | 0.0000000 | -0.1013701 | 2 | 3 | 104.32169 | 52.401818 |
| 0.0000000 | -1.9506290 | -0.4934321 | -3.0829011 | -0.1426558 | -1.1347763 | -0.0834103 | 0.0000000 | 0.0000000 | -1.0639013 | 0.0000000 | 0.0000000 | -4.9550730 | 0.0000000 | 0.0000000 | 0.0000000 | -0.4089264 | 0.0000000 | 0.0000000 | 0.0000000 | -0.0432632 | -0.8428410 | -0.8383745 | 0.0000000 | -0.0178791 | 2 | 4 | 80.88658 | 29.619545 |
| 0.0000000 | -1.9869794 | -1.6625883 | -3.3046583 | -2.2388026 | -0.4994856 | -0.0500972 | 0.0000000 | 0.0000000 | -0.1429364 | 0.0000000 | 0.0000000 | -4.1684222 | 0.0000000 | 0.0000000 | 0.0000000 | -0.3675409 | -0.7849183 | 0.0000000 | 0.0000000 | 0.0000000 | -0.2154698 | -0.5485285 | 0.0000000 | 0.0000000 | 2 | 5 | 70.59361 | 19.839500 |
| 0.0000000 | -2.7098489 | 0.0000000 | -4.1080542 | -3.8504717 | -2.6544278 | 0.0000000 | 0.0000000 | 0.0000000 | -0.0369014 | 0.0000000 | 0.0000000 | -6.5382457 | 0.0000000 | 0.0000000 | 0.0000000 | -0.9435864 | 0.0000000 | 0.0000000 | 0.0000000 | 0.0000000 | -1.2537515 | -0.2025160 | -0.1666552 | -0.9175486 | 2 | 6 | 61.60508 | 11.138120 |
| 0.0000000 | -0.9519362 | -2.0571593 | -0.0310022 | 0.0000000 | -0.5578909 | -0.2669569 | -0.0235350 | -0.4194692 | -0.0235974 | -0.0356587 | 0.0000000 | -0.6420498 | -0.4668207 | 0.0000000 | 0.0000000 | -0.1698236 | -0.2009548 | 0.0000000 | 0.0000000 | -0.0718975 | -0.1801458 | -0.3154582 | -0.0418917 | 0.0000000 | 3 | 1 | 171.82320 | 116.537967 |
| 0.0000000 | -0.7748894 | -2.1468879 | 0.0000000 | 0.0000000 | -1.1168377 | -0.1363150 | 0.0000000 | -0.4821682 | 0.0000000 | 0.0000000 | -0.5730595 | -0.1301122 | -0.1898292 | 0.0000000 | 0.0000000 | -0.1859785 | -0.0442898 | 0.0000000 | 0.0000000 | -0.0315971 | -0.3316979 | -0.4598337 | -0.0057266 | -0.3209207 | 3 | 2 | 130.14029 | 76.899762 |
| 0.0000000 | -1.3278605 | -1.6976272 | 0.0000000 | 0.0000000 | -2.1953323 | -0.0993425 | 0.0000000 | 0.0000000 | -0.2533378 | 0.0000000 | -0.7183343 | -1.1926098 | -0.6736009 | 0.0000000 | 0.0000000 | -0.1409208 | -0.1969652 | 0.0000000 | 0.0000000 | 0.0000000 | -0.4605687 | -0.6526191 | 0.0000000 | 0.0000000 | 3 | 3 | 95.98992 | 44.254697 |
| 0.0000000 | -2.3170108 | -1.2402331 | -2.3312496 | 0.0000000 | -0.9391527 | -0.3490722 | 0.0000000 | 0.0000000 | -1.4335434 | 0.0000000 | -1.0605660 | -2.5871587 | 0.0000000 | 0.0000000 | 0.0000000 | -0.5528432 | -0.1620802 | 0.0000000 | 0.0000000 | 0.0000000 | -0.6567036 | -0.1354244 | -0.2009885 | 0.0000000 | 3 | 4 | 81.95628 | 30.724193 |
| 0.0000000 | -3.2208523 | 0.0000000 | -4.0947876 | -0.3011124 | -1.7203078 | 0.0000000 | 0.0000000 | 0.0000000 | -1.9414015 | 0.0000000 | 0.0000000 | -3.6069459 | 0.0000000 | 0.0000000 | 0.0000000 | -0.0873245 | -0.3914559 | 0.0000000 | 0.0000000 | 0.0000000 | -0.5594024 | -0.7181934 | 0.0000000 | 0.0000000 | 3 | 5 | 75.83606 | 25.064774 |
| 0.0000000 | -3.1335935 | 0.0000000 | -3.0476027 | -1.3991441 | -1.9831159 | -0.7624279 | 0.0000000 | 0.0000000 | -0.5236728 | 0.0000000 | 0.0000000 | -7.6342733 | -0.1288509 | 0.0000000 | 0.0000000 | -0.2674012 | -0.1569824 | -0.2231929 | 0.0000000 | 0.0000000 | -0.7239350 | -0.2760647 | 0.0000000 | 0.0000000 | 3 | 6 | 67.48411 | 16.912212 |
| 0.0000000 | -0.7358369 | -2.2108760 | 0.0000000 | 0.0000000 | -0.7713193 | -0.2587197 | -0.0396868 | -0.4025410 | 0.0000000 | -0.1960566 | 0.0000000 | -0.5081856 | -0.0367100 | 0.0000000 | 0.0000000 | -0.1913701 | -0.2803682 | 0.0000000 | 0.0000000 | -0.2705221 | 0.0000000 | -0.1712341 | -0.1651213 | 0.0000000 | 4 | 1 | 172.53239 | 117.316974 |
| 0.0000000 | -0.7008137 | -2.4327018 | -0.4305144 | 0.0000000 | -0.7697490 | -0.3288915 | 0.0000000 | -0.2552506 | -0.1720312 | 0.0000000 | -0.3428133 | -0.3176453 | 0.0000000 | 0.0000000 | 0.0000000 | -0.1928437 | 0.0000000 | 0.0000000 | 0.0000000 | 0.0000000 | -0.1643204 | -0.5416980 | -0.0878636 | 0.0000000 | 4 | 2 | 138.45052 | 85.177078 |
| 0.0000000 | -0.9542813 | -1.8903648 | -1.6623112 | 0.0000000 | -1.8776975 | -0.3708382 | 0.0000000 | -0.7993573 | 0.0000000 | 0.0000000 | -0.4438352 | -0.7118488 | 0.0000000 | 0.0000000 | 0.0000000 | -0.3090740 | 0.0000000 | -0.1455889 | 0.0000000 | 0.0000000 | -0.2106563 | -0.4220037 | 0.0000000 | -0.1204551 | 4 | 3 | 98.27022 | 46.398014 |
| 0.0000000 | -2.0649081 | -1.5232170 | -2.2755343 | -0.1677268 | -1.3042948 | -0.1075499 | 0.0000000 | 0.0000000 | -0.7941234 | 0.0000000 | 0.0000000 | -3.7734035 | 0.0000000 | 0.0000000 | 0.0000000 | -0.0433045 | 0.0000000 | -0.4557226 | 0.0000000 | 0.0000000 | -0.3021206 | -0.3541385 | -0.6260185 | 0.0000000 | 4 | 4 | 81.57796 | 30.366121 |
| 0.0000000 | -2.4469411 | 0.0000000 | -3.6571537 | -2.5966051 | 0.0000000 | -0.1961104 | 0.0000000 | -0.1586965 | -0.3740744 | 0.0000000 | 0.0000000 | -7.6503492 | 0.0000000 | 0.0000000 | 0.0000000 | 0.0000000 | 0.0000000 | -0.0842193 | 0.0000000 | 0.0000000 | -1.1647353 | -0.9660038 | -0.0950640 | -0.1147880 | 4 | 5 | 68.29531 | 17.551078 |
| 0.0000000 | -2.9676010 | -1.1131814 | -5.3157084 | -0.5142894 | -1.0497092 | -0.1956603 | 0.0000000 | 0.0000000 | -0.3321778 | 0.0000000 | 0.0000000 | -4.7410016 | 0.0000000 | 0.0000000 | 0.0000000 | -0.1713184 | 0.0000000 | -0.7376889 | 0.0000000 | 0.0000000 | -0.1726807 | -0.3733466 | -0.0132557 | -0.0023189 | 4 | 6 | 66.25182 | 15.649359 |
| 0.0000000 | -0.8484098 | -1.9367060 | -0.3994287 | 0.0000000 | -0.7016614 | -0.2161879 | -0.0427741 | -0.3782097 | 0.0000000 | -0.1681503 | -0.2448304 | -0.3991081 | 0.0000000 | 0.0000000 | 0.0000000 | -0.0587226 | -0.2199784 | 0.0000000 | 0.0000000 | -0.0780027 | 0.0000000 | -0.3146138 | -0.1617135 | 0.0000000 | 5 | 1 | 171.38538 | 116.133216 |
| 0.0000000 | -1.2612538 | -1.4425846 | -0.9850332 | 0.0000000 | -0.9478961 | -0.5691649 | 0.0000000 | 0.0000000 | -0.2538477 | -0.0186708 | -0.3411101 | -1.0304159 | 0.0000000 | 0.0000000 | 0.0000000 | -0.2608866 | -0.1689028 | 0.0000000 | 0.0000000 | 0.0000000 | -0.3446701 | -0.6387961 | 0.0000000 | 0.0000000 | 5 | 2 | 126.08958 | 73.102940 |
| 0.0000000 | -1.2160804 | -1.6020504 | -0.9233029 | -0.2664791 | -1.7394211 | -0.4034657 | 0.0000000 | -0.3893244 | -0.1098532 | 0.0000000 | -0.0719227 | -1.7155684 | -0.9165447 | 0.0000000 | 0.0000000 | -0.1856332 | 0.0000000 | -0.3542264 | 0.0000000 | 0.0000000 | -0.2830751 | -0.2508139 | -0.5763519 | 0.0000000 | 5 | 3 | 93.34226 | 41.529278 |
| 0.0000000 | -2.1456811 | -0.0682832 | -3.2473502 | -1.7174094 | -1.8410490 | -0.2346000 | 0.0000000 | 0.0000000 | -0.5134826 | 0.0000000 | -0.7327847 | -1.7910533 | 0.0000000 | 0.0000000 | 0.0000000 | -0.1845372 | -0.3518816 | 0.0000000 | 0.0000000 | 0.0000000 | -0.9975673 | -0.3451623 | -0.2537115 | 0.0000000 | 5 | 4 | 77.99407 | 26.720167 |
| 0.0000000 | -1.9133587 | -0.3214088 | -4.8574366 | 0.0000000 | -1.5107637 | 0.0000000 | 0.0000000 | 0.0000000 | -0.3690912 | 0.0000000 | 0.0000000 | -3.6320033 | 0.0000000 | 0.0000000 | 0.0000000 | -0.1521109 | 0.0000000 | 0.0000000 | 0.0000000 | -0.3946554 | -0.5720902 | -0.8582830 | 0.0000000 | -1.0759672 | 5 | 5 | 74.43017 | 23.577097 |
| 0.0000000 | -2.9041510 | 0.0000000 | -3.7203691 | -3.1793935 | -1.6827025 | -0.3462477 | 0.0000000 | 0.0000000 | -0.7508620 | 0.0000000 | 0.0000000 | -6.1026643 | 0.0000000 | 0.0000000 | 0.0000000 | 0.0000000 | -0.3012303 | 0.0000000 | 0.0000000 | 0.0000000 | -0.7715060 | -0.3189218 | -0.5987501 | -0.7699000 | 5 | 6 | 65.07460 | 14.561005 |
| 0.0000000 | -1.0089126 | -2.0679840 | -0.0574945 | -0.1920005 | -0.6444416 | -0.1797537 | -0.0169885 | -0.5926530 | 0.0000000 | -0.1358143 | -0.0348734 | -0.3840162 | -0.0666235 | 0.0000000 | 0.0000000 | -0.0775967 | -0.1889042 | 0.0000000 | -0.1462181 | 0.0000000 | -0.1007026 | -0.2586210 | -0.0704963 | -0.2359297 | 6 | 1 | 178.03062 | 122.923304 |
| 0.0000000 | -0.6535804 | -2.2187049 | -0.4585208 | 0.0000000 | -1.1436235 | -0.6404603 | 0.0000000 | -0.1965199 | 0.0000000 | -0.1052376 | -0.5907975 | -0.1077083 | 0.0000000 | 0.0000000 | 0.0000000 | -0.2452263 | 0.0000000 | 0.0000000 | 0.0000000 | 0.0000000 | -0.2651258 | -0.4048591 | 0.0000000 | 0.0000000 | 6 | 2 | 129.72278 | 76.672291 |
| 0.0000000 | -1.3538102 | -2.4737917 | 0.0000000 | 0.0000000 | -1.9728089 | -0.2543580 | 0.0000000 | -0.1462026 | -0.2025284 | -0.1473877 | -0.4624385 | -1.6745419 | 0.0000000 | 0.0000000 | 0.0000000 | -0.3501217 | -0.0261105 | -0.0882711 | 0.0000000 | 0.0000000 | -0.3772743 | -0.3308527 | 0.0000000 | 0.0000000 | 6 | 3 | 105.24052 | 53.345757 |
| 0.0000000 | -1.9394680 | -1.3400531 | -1.6048552 | -0.3020734 | -1.6892282 | 0.0000000 | 0.0000000 | -0.4315509 | -0.0329618 | 0.0000000 | 0.0000000 | -2.7696301 | -0.4865959 | 0.0000000 | 0.0000000 | -0.1363299 | -0.2106688 | -0.2266395 | 0.0000000 | 0.0000000 | -0.4871601 | -0.5465717 | 0.0000000 | -0.6668989 | 6 | 4 | 83.97428 | 32.705245 |
| 0.0000000 | -2.4545394 | -0.8431572 | -2.6040629 | -2.1053151 | -0.2642611 | 0.0000000 | 0.0000000 | 0.0000000 | -1.0165902 | 0.0000000 | 0.0000000 | -6.0014988 | -0.4701514 | 0.0000000 | 0.0000000 | 0.0000000 | 0.0000000 | 0.0000000 | 0.0000000 | 0.0000000 | -0.5043037 | -0.5984074 | -0.6901584 | 0.0000000 | 6 | 5 | 73.18574 | 22.338670 |
| 2.0474970 | -2.5401976 | 0.0000000 | -4.5873071 | -2.5756603 | -0.6610456 | 0.0000000 | 0.0000000 | -0.3787813 | -1.3959927 | 0.0000000 | 0.0000000 | -8.9494270 | 0.0000000 | 0.0000000 | 0.0000000 | 0.0000000 | -0.2902609 | 0.0000000 | 0.0000000 | 0.0000000 | -0.5181783 | -2.6497378 | -0.1008279 | 0.0000000 | 6 | 6 | 63.29662 | 12.809486 |
| 0.0000000 | -1.0063634 | -1.8515060 | -0.3314578 | -0.4039856 | -0.6759571 | -0.0821420 | -0.0233611 | -0.2815942 | -0.1742471 | -0.2351911 | -0.1487995 | -0.1902968 | -0.0015483 | 0.0000000 | 0.0000000 | -0.1664219 | -0.0833628 | 0.0000000 | 0.0000000 | -0.1556658 | -0.0642857 | -0.3326445 | -0.1691733 | -0.0000508 | 7 | 1 | 176.26207 | 121.115367 |
| 0.0000000 | -0.8835527 | -2.4655131 | -0.2028591 | 0.0000000 | -1.0630897 | -0.3029895 | -0.1972171 | 0.0000000 | -0.1768085 | 0.0000000 | -0.3243114 | -0.4062259 | 0.0000000 | 0.0000000 | 0.0000000 | -0.3229320 | -0.0632449 | -0.0793050 | 0.0000000 | 0.0000000 | -0.2405304 | -0.4474460 | -0.0455198 | 0.0000000 | 7 | 2 | 133.18904 | 80.054958 |
| 0.1715257 | -1.3903583 | -1.7615349 | -1.6065104 | 0.0000000 | -1.0895826 | -0.7079766 | 0.0000000 | -0.1078152 | -0.7317133 | 0.0000000 | -0.7850359 | -0.6660249 | 0.0000000 | 0.0000000 | 0.0000000 | -0.3513816 | 0.0000000 | -0.1992438 | 0.0000000 | 0.0000000 | -0.3504168 | -0.6368120 | 0.0000000 | -0.3004830 | 7 | 3 | 106.46370 | 54.376985 |
| 0.0000000 | -1.6668439 | -1.3906548 | -2.9037627 | -0.5561095 | -1.1120246 | -0.3041354 | 0.0000000 | 0.0000000 | -0.5026376 | 0.0000000 | 0.0000000 | -2.7607924 | 0.0000000 | 0.0000000 | 0.0000000 | -0.3708042 | 0.0000000 | 0.0000000 | 0.0000000 | 0.0000000 | -0.3913330 | -0.7300277 | -0.1713031 | 0.0000000 | 7 | 4 | 79.22335 | 27.917239 |
| 0.0000000 | -2.1685777 | 0.0000000 | -2.5220188 | -0.9396652 | -1.5900555 | -0.4335924 | 0.0000000 | -0.3997758 | -0.3725381 | 0.0000000 | -0.1279827 | -4.2391521 | -0.2187276 | 0.0000000 | 0.0000000 | -0.7380730 | 0.0000000 | -0.3316567 | 0.0000000 | 0.0000000 | -0.4494746 | -0.5644373 | -0.2726866 | -0.1803064 | 7 | 5 | 77.60719 | 26.773102 |
| 0.5583071 | -2.5257874 | 0.0000000 | -3.2929710 | -1.6536259 | -2.3530554 | -0.2212783 | 0.0000000 | 0.0000000 | -0.7616024 | 0.0000000 | 0.0000000 | -9.3577687 | 0.0000000 | 0.0000000 | -0.0867957 | 0.0000000 | 0.0000000 | 0.0000000 | 0.0000000 | 0.0000000 | -0.4042319 | -1.2570213 | -0.2643814 | -0.8931033 | 7 | 6 | 63.77902 | 13.152087 |
| 0.0000000 | -0.7438108 | -1.8740669 | 0.0000000 | -0.1604925 | -0.8220934 | -0.3055054 | -0.1486681 | -0.3968941 | 0.0000000 | -0.1151655 | 0.0000000 | -0.4467511 | 0.0000000 | 0.0000000 | 0.0000000 | -0.1840546 | -0.0976255 | 0.0000000 | -0.1500578 | -0.1264610 | -0.1763535 | -0.3871663 | 0.0000000 | -0.0655220 | 8 | 1 | 183.98848 | 128.802693 |
| 0.0000000 | -0.7796874 | -2.0541955 | -0.4879898 | 0.0000000 | -1.2885117 | -0.1611301 | 0.0000000 | -0.3277376 | -0.1058303 | 0.0000000 | -0.1989786 | -0.6420047 | -0.2734367 | 0.0000000 | 0.0000000 | -0.1391348 | 0.0000000 | 0.0000000 | 0.0000000 | 0.0000000 | -0.2477901 | -0.5114762 | -0.1638320 | 0.0000000 | 8 | 2 | 121.23486 | 68.223869 |
| 0.0000000 | -1.1568633 | -1.7517701 | -0.4492177 | 0.0000000 | -1.9398419 | -0.4956212 | -0.0679016 | 0.0000000 | -0.4323881 | 0.0000000 | 0.0000000 | -0.8723728 | -1.5078409 | 0.0000000 | 0.0000000 | -0.2384765 | -0.0381299 | 0.0000000 | 0.0000000 | 0.0000000 | -0.3229471 | -0.7477963 | 0.0000000 | -0.0005089 | 8 | 3 | 100.44633 | 48.653146 |
| 0.0000000 | -0.9425814 | -2.8228541 | -0.0963988 | -0.3528839 | -1.5855136 | -0.4548842 | 0.0000000 | 0.0000000 | -0.1776231 | -0.2560941 | 0.0000000 | -1.1900600 | -1.5125439 | 0.0000000 | 0.0000000 | -0.6977856 | 0.0000000 | 0.0000000 | 0.0000000 | 0.0000000 | 0.0000000 | -0.3175163 | 0.0000000 | -0.3266741 | 8 | 4 | 88.37063 | 37.190531 |
| 0.8734207 | -1.5775185 | 0.0000000 | -4.4080801 | -3.0649731 | -0.2589190 | 0.0000000 | -0.0241741 | 0.0000000 | -0.9648065 | 0.0000000 | 0.0000000 | -6.2925713 | 0.0000000 | 0.0000000 | 0.0000000 | 0.0000000 | 0.0000000 | 0.0000000 | 0.0000000 | 0.0000000 | -0.2239857 | -2.3649410 | 0.0000000 | -1.0416380 | 8 | 5 | 68.42237 | 17.606377 |
| 0.0000000 | -3.5948233 | 0.0000000 | -3.0462476 | -0.3778432 | -1.8388440 | 0.0000000 | 0.0000000 | -0.2438689 | -0.7201261 | 0.0000000 | 0.0000000 | -5.2596115 | -0.7038083 | 0.0000000 | 0.0000000 | 0.0000000 | 0.0000000 | 0.0000000 | 0.0000000 | 0.0000000 | -1.3654767 | -0.1963928 | -0.3382112 | 0.0000000 | 8 | 6 | 72.06257 | 21.416017 |
| 0.0000000 | -0.7546081 | -2.1526664 | -0.0193580 | 0.0000000 | -0.8430538 | -0.3432097 | -0.0307258 | -0.4092806 | 0.0000000 | -0.0809515 | -0.2659039 | -0.0859049 | -0.2838789 | 0.0000000 | 0.0000000 | -0.2144429 | -0.3132708 | 0.0000000 | -0.0127824 | -0.1196421 | -0.0665651 | -0.1594781 | -0.1632275 | 0.0000000 | 9 | 1 | 175.31253 | 120.319209 |
| 0.0000000 | -0.6263162 | -2.1225279 | 0.0000000 | 0.0000000 | -1.4734873 | -0.1771333 | -0.2007925 | -0.2291068 | -0.1592488 | 0.0000000 | -0.6597827 | -0.0351118 | 0.0000000 | 0.0000000 | 0.0000000 | -0.2728072 | -0.0239181 | 0.0000000 | 0.0000000 | 0.0000000 | -0.3343932 | -0.5222640 | -0.0379674 | 0.0000000 | 9 | 2 | 128.09835 | 74.995667 |
| 0.0000000 | -0.9678363 | -2.1880712 | 0.0000000 | 0.0000000 | -1.6326939 | -0.0704951 | 0.0000000 | -0.0468307 | -0.1416347 | -0.1707883 | -1.1433570 | -0.9032664 | 0.0000000 | 0.0000000 | 0.0000000 | -0.2936528 | 0.0000000 | 0.0000000 | 0.0000000 | 0.0000000 | -0.1939737 | -0.6321466 | 0.0000000 | 0.0000000 | 9 | 3 | 106.16526 | 54.151774 |
| 0.0000000 | -1.1719359 | -1.9973856 | -1.5596492 | 0.0000000 | -2.2001512 | -0.1422552 | 0.0000000 | 0.0000000 | 0.0000000 | 0.0000000 | -0.2587903 | -2.1061174 | 0.0000000 | 0.0000000 | 0.0000000 | -0.1821429 | 0.0000000 | 0.0000000 | 0.0000000 | 0.0000000 | -0.3888977 | -0.7803592 | 0.0000000 | 0.0000000 | 9 | 4 | 87.38255 | 36.127965 |
| 0.8111492 | -2.6502701 | 0.0000000 | -3.7920565 | -2.2825844 | -0.9947074 | 0.0000000 | 0.0000000 | 0.0000000 | -0.5759956 | 0.0000000 | 0.0000000 | -5.1733198 | 0.0000000 | 0.0000000 | 0.0000000 | 0.0000000 | 0.0000000 | 0.0000000 | 0.0000000 | 0.0000000 | -0.4426591 | -1.5524562 | -0.8169538 | 0.0000000 | 9 | 5 | 70.72658 | 19.922235 |
| 0.0000000 | -2.9233240 | 0.0000000 | -7.1742950 | -4.0814130 | -1.8801259 | 0.0000000 | 0.0000000 | 0.0000000 | -0.7085802 | 0.0000000 | 0.0000000 | -5.7008812 | 0.0000000 | 0.0000000 | 0.0000000 | -0.7510539 | -0.0945783 | 0.0000000 | 0.0000000 | 0.0000000 | -0.3033218 | -0.5919914 | 0.0000000 | -0.7519249 | 9 | 6 | 59.57033 | 9.059285 |
| 0.0000000 | -0.9807274 | -1.5961490 | -0.5232632 | -0.0599824 | -0.9954573 | -0.2762634 | 0.0000000 | -0.2365861 | 0.0000000 | -0.1783618 | -0.3575133 | -0.0081760 | 0.0000000 | 0.0000000 | 0.0000000 | -0.2159152 | -0.1530016 | 0.0000000 | 0.0000000 | -0.2318399 | -0.0252091 | -0.2524421 | -0.1943180 | -0.0171886 | 10 | 1 | 179.27482 | 124.089975 |
| 0.0000000 | -0.8061394 | -2.2584627 | -0.0260810 | 0.0000000 | -1.4608696 | -0.3857683 | -0.0955223 | -0.3367498 | -0.2195815 | -0.0246271 | -0.2160355 | -0.5395274 | 0.0000000 | 0.0000000 | 0.0000000 | -0.0441061 | 0.0000000 | 0.0000000 | 0.0000000 | -0.0598409 | -0.0942502 | -0.4956515 | 0.0000000 | 0.0000000 | 10 | 2 | 129.58012 | 76.463012 |
| 0.0000000 | -0.8767730 | -2.0729795 | -0.3072397 | 0.0000000 | -1.5198956 | -0.2161794 | 0.0000000 | -0.8957628 | 0.0000000 | 0.0000000 | 0.0000000 | -0.8781064 | -1.0613047 | 0.0000000 | 0.0000000 | -0.4685050 | 0.0000000 | -0.2777118 | 0.0000000 | 0.0000000 | -0.0444641 | -0.3114183 | -0.2919224 | 0.0000000 | 10 | 3 | 102.41647 | 50.537726 |
| 0.0000000 | -2.2333917 | -0.0926801 | -3.9056701 | 0.0000000 | -1.0396099 | -0.2632907 | 0.0000000 | 0.0000000 | -0.4506049 | 0.0000000 | 0.0000000 | -3.6591548 | 0.0000000 | 0.0000000 | 0.0000000 | -0.1469546 | 0.0000000 | 0.0000000 | 0.0000000 | 0.0000000 | -0.3614838 | -1.3021300 | 0.0000000 | 0.0000000 | 10 | 4 | 81.91421 | 30.714700 |
| 1.0687509 | -1.9580806 | -1.9689184 | -1.8153749 | 0.0000000 | -3.1369591 | -0.3293423 | 0.0000000 | 0.0000000 | 0.0000000 | 0.0000000 | -1.8317725 | -0.3322715 | 0.0000000 | 0.0000000 | 0.0000000 | -0.1929702 | -0.5407659 | 0.0000000 | 0.0000000 | 0.0000000 | -0.4620587 | -0.5361075 | -0.5517432 | 0.0000000 | 10 | 5 | 75.32092 | 24.525657 |
| 0.0000000 | -4.3117903 | 0.0000000 | -4.6310560 | -1.7668158 | -0.5689846 | -0.3535363 | 0.0000000 | 0.0000000 | 0.0000000 | 0.0000000 | 0.0000000 | -5.7998466 | -0.2389120 | 0.0000000 | 0.0000000 | -0.8201425 | 0.0000000 | 0.0000000 | 0.0000000 | 0.0000000 | -1.4196408 | -0.2211591 | 0.0000000 | -0.6070536 | 10 | 6 | 68.28052 | 17.813209 |
| 0.0000000 | -1.0554931 | -1.7813125 | -0.2823058 | -0.0211857 | -0.9043780 | -0.1858978 | 0.0000000 | -0.3908118 | 0.0000000 | -0.0037322 | -0.5254614 | -0.0822307 | 0.0000000 | 0.0000000 | 0.0000000 | -0.1112992 | -0.2819891 | 0.0000000 | -0.0364112 | 0.0000000 | -0.1397654 | -0.3330950 | -0.0440695 | 0.0000000 | 11 | 1 | 176.80196 | 121.662433 |
| 0.0000000 | -0.8634503 | -2.3205427 | 0.0000000 | -0.3370517 | -0.6945852 | -0.4192666 | 0.0000000 | -0.4509244 | 0.0000000 | 0.0000000 | -0.7064235 | -0.4233927 | 0.0000000 | 0.0000000 | 0.0000000 | -0.1118908 | -0.0866545 | 0.0000000 | 0.0000000 | 0.0000000 | -0.2489446 | -0.4425405 | -0.0258116 | -0.0880666 | 11 | 2 | 131.00112 | 77.924112 |
| 0.0000000 | -0.8239100 | -2.0167138 | -0.1681570 | 0.0000000 | -2.2743190 | -0.0191620 | 0.0000000 | 0.0000000 | -0.0929167 | 0.0000000 | -0.0336248 | -0.5433104 | -1.3637985 | 0.0000000 | 0.0000000 | -0.2380833 | 0.0000000 | 0.0000000 | 0.0000000 | 0.0000000 | -0.4028955 | -0.6707596 | -0.1959127 | 0.0000000 | 11 | 3 | 100.29269 | 48.324841 |
| 0.0000000 | -2.1085609 | -1.5700376 | -1.7105720 | 0.0000000 | -2.0761674 | -0.0297163 | 0.0000000 | -0.5071768 | -0.2967603 | -0.7527534 | 0.0000000 | -2.5559684 | 0.0000000 | 0.0000000 | 0.0000000 | -0.1545764 | -0.0191561 | 0.0000000 | 0.0000000 | 0.0000000 | -0.1023711 | -0.2583134 | -0.1419913 | 0.0000000 | 11 | 4 | 82.34771 | 31.251662 |
| 0.0000000 | -1.7056425 | 0.0000000 | -3.8045367 | -3.1619184 | -1.1850331 | 0.0000000 | 0.0000000 | 0.0000000 | -1.0308857 | 0.0000000 | 0.0000000 | -6.5536962 | 0.0000000 | 0.0000000 | 0.0000000 | 0.0000000 | -0.3597813 | 0.0000000 | 0.0000000 | 0.0000000 | -0.7033001 | -0.6409626 | -0.3747801 | -0.4056950 | 11 | 5 | 68.77464 | 17.917881 |
| 0.0000000 | -1.8878891 | 0.0000000 | -5.2525526 | -3.5287114 | -0.3149909 | 0.0000000 | 0.0000000 | 0.0000000 | -0.2729345 | 0.0000000 | 0.0000000 | -7.6830957 | 0.0000000 | 0.0000000 | 0.0000000 | 0.0000000 | 0.0000000 | 0.0000000 | 0.0000000 | -0.0088386 | -0.5971031 | -1.2858232 | 0.0000000 | 0.0000000 | 11 | 6 | 64.22723 | 13.711637 |
| 0.0000000 | -0.9935784 | -1.8948709 | -0.0515504 | 0.0000000 | -0.8297272 | -0.3808638 | -0.2139181 | -0.2778531 | 0.0000000 | -0.0644431 | 0.0000000 | -0.0769926 | -0.3636588 | 0.0000000 | 0.0000000 | -0.2688074 | -0.1713203 | 0.0000000 | 0.0000000 | -0.1659942 | -0.2480959 | -0.3532217 | 0.0000000 | -0.1353746 | 12 | 1 | 185.40229 | 130.200243 |
| 0.0000000 | -0.6610545 | -2.3684544 | -0.2374924 | -0.0095115 | -1.3616327 | -0.1659627 | -0.1403663 | -0.1584236 | -0.3862814 | 0.0000000 | -0.5620787 | 0.0000000 | 0.0000000 | 0.0000000 | 0.0000000 | -0.3189792 | 0.0000000 | -0.1525590 | 0.0000000 | 0.0000000 | -0.1661178 | -0.4607856 | 0.0000000 | 0.0000000 | 12 | 2 | 126.65246 | 73.607582 |
| 0.0076721 | -0.8142003 | -2.5138752 | -0.4631669 | 0.0000000 | -1.6781582 | 0.0000000 | 0.0000000 | 0.0000000 | -0.5737057 | 0.0000000 | -1.1767669 | -0.3826403 | 0.0000000 | 0.0000000 | 0.0000000 | -0.2672741 | 0.0000000 | 0.0000000 | 0.0000000 | 0.0000000 | -0.2540407 | -0.3170528 | -0.3853517 | 0.0000000 | 12 | 3 | 108.17543 | 56.179311 |
| 0.0000000 | -1.9174089 | -0.3063206 | -2.5355364 | 0.0000000 | -1.4355948 | 0.0000000 | 0.0000000 | 0.0000000 | -1.1541824 | 0.0000000 | 0.0000000 | -3.6560852 | -0.7847123 | 0.0000000 | 0.0000000 | -0.2450856 | 0.0000000 | -0.1599867 | 0.0000000 | 0.0000000 | -0.3583272 | -1.2209202 | 0.0000000 | 0.0000000 | 12 | 4 | 79.17682 | 28.028776 |
| 0.0000000 | -1.7061424 | 0.0000000 | -4.0089642 | -1.8187849 | -0.8512337 | -0.0296711 | 0.0000000 | 0.0000000 | -0.9198833 | -0.5056027 | 0.0000000 | -4.3886501 | -0.3267777 | 0.0000000 | 0.0000000 | -0.2567570 | 0.0000000 | 0.0000000 | 0.0000000 | 0.0000000 | -0.0416714 | -0.6241301 | -0.9249187 | 0.0000000 | 12 | 5 | 70.77379 | 20.003328 |
| 0.6626839 | -3.0393852 | -0.2498095 | -3.4572894 | 0.0000000 | -1.8838945 | -0.2453035 | 0.0000000 | 0.0000000 | -0.4092034 | 0.0000000 | 0.0000000 | -4.7746528 | -0.5190062 | 0.0000000 | 0.0000000 | 0.0000000 | 0.0000000 | 0.0000000 | 0.0000000 | 0.0000000 | -0.2122539 | -1.3445768 | -0.5353370 | -0.9518334 | 12 | 6 | 69.46174 | 18.903028 |
| 0.0000000 | -1.3784247 | -1.7227003 | -0.3458981 | -0.0244685 | -0.9848880 | -0.0629626 | -0.1120011 | -0.4426707 | -0.1174171 | 0.0000000 | -0.3249856 | -0.1325511 | 0.0000000 | 0.0000000 | 0.0000000 | -0.1321286 | -0.2483965 | 0.0000000 | 0.0000000 | -0.0311325 | -0.2091309 | -0.2948423 | -0.0146229 | -0.1625538 | 13 | 1 | 185.97002 | 131.023939 |
| 0.0000000 | -0.8794701 | -1.7480822 | -0.6258508 | 0.0000000 | -1.2685431 | -0.3213971 | 0.0000000 | -0.4861118 | -0.1464677 | 0.0000000 | -0.7097837 | 0.0000000 | 0.0000000 | 0.0000000 | 0.0000000 | -0.1374296 | -0.0436932 | -0.0307873 | 0.0000000 | -0.0978040 | -0.3472483 | -0.4237119 | -0.0144757 | 0.0000000 | 13 | 2 | 126.81187 | 73.615044 |
| 0.0000000 | -0.9365397 | -2.1476971 | -0.5466531 | -0.1745822 | -1.7039492 | -0.3788911 | 0.0000000 | 0.0000000 | -0.1196096 | 0.0000000 | 0.0000000 | -2.1696449 | 0.0000000 | 0.0000000 | 0.0000000 | -0.1934553 | 0.0000000 | 0.0000000 | 0.0000000 | 0.0000000 | -0.4155966 | -0.5958247 | -0.1379591 | 0.0000000 | 13 | 3 | 104.90897 | 52.919637 |
| 0.0000000 | -1.6275366 | -1.0318155 | -2.1499013 | 0.0000000 | -1.6039236 | 0.0000000 | 0.0000000 | 0.0000000 | -0.6425785 | 0.0000000 | 0.0000000 | -2.7778258 | -0.5079036 | 0.0000000 | 0.0000000 | -0.4400056 | 0.0000000 | -0.2518297 | 0.0000000 | 0.0000000 | -0.2413758 | -0.7541643 | -0.3107257 | 0.0000000 | 13 | 4 | 81.71215 | 30.384357 |
| 0.0000000 | -1.3485203 | -1.4492822 | -2.2393892 | 0.0000000 | -1.6963343 | -0.2550211 | 0.0000000 | 0.0000000 | -0.5934967 | 0.0000000 | 0.0000000 | -3.2232289 | -1.0854286 | 0.0000000 | 0.0000000 | -0.3117362 | 0.0000000 | 0.0000000 | 0.0000000 | 0.0000000 | -0.5163595 | -0.0782546 | -0.8838123 | 0.0000000 | 13 | 5 | 76.49324 | 25.688532 |
| 0.0000000 | -2.0425127 | 0.0000000 | -1.2590392 | -2.0047434 | -1.3255920 | 0.0000000 | 0.0000000 | 0.0000000 | -0.0909479 | 0.0000000 | 0.0000000 | -18.8357435 | 0.0000000 | 0.0000000 | 0.0000000 | 0.0000000 | 0.0000000 | 0.0000000 | 0.0000000 | 0.0000000 | -1.5054199 | -0.8636377 | 0.0000000 | 0.0000000 | 13 | 6 | 61.50047 | 10.955283 |
| 0.0000000 | -1.0020557 | -2.0619451 | -0.1730015 | -0.3977610 | -0.3313999 | -0.5661355 | -0.0327618 | -0.4198560 | 0.0000000 | -0.0952684 | -0.3730031 | -0.2317345 | -0.1149630 | 0.0000000 | 0.0000000 | -0.1551177 | -0.1169794 | 0.0000000 | 0.0000000 | -0.0120309 | -0.1107557 | -0.2951455 | -0.0852676 | -0.0898955 | 14 | 1 | 166.23200 | 111.023107 |
| 0.0000000 | -0.6360404 | -2.5944011 | 0.0000000 | 0.0000000 | -0.7079200 | -0.5004298 | 0.0000000 | -0.0517245 | -0.5091533 | -0.1477829 | -0.1911426 | -0.3993017 | -0.8605272 | 0.0000000 | 0.0000000 | -0.4006700 | 0.0000000 | -0.1875518 | 0.0000000 | 0.0000000 | -0.3102421 | -0.1891970 | -0.1792761 | 0.0000000 | 14 | 2 | 125.05475 | 71.996249 |
| 0.0000000 | -1.7102637 | -1.9973362 | 0.0000000 | 0.0000000 | -1.7491045 | -0.3145635 | 0.0000000 | -0.1399995 | 0.0000000 | 0.0000000 | 0.0000000 | -1.3739604 | -0.7063346 | 0.0000000 | 0.0000000 | -0.1257517 | 0.0000000 | 0.0000000 | 0.0000000 | 0.0000000 | -0.5043386 | -0.5833484 | -0.2194896 | 0.0000000 | 14 | 3 | 105.26682 | 53.389871 |
| 0.0000000 | -1.7490188 | -0.5273505 | -2.4928913 | 0.0000000 | -1.8749466 | 0.0000000 | 0.0000000 | -0.0627866 | -0.6733574 | 0.0000000 | -0.2106589 | -2.4922230 | -0.1433952 | 0.0000000 | 0.0000000 | -0.7071281 | 0.0000000 | 0.0000000 | 0.0000000 | 0.0000000 | -0.4281118 | -1.0069768 | -0.0852969 | 0.0000000 | 14 | 4 | 84.47530 | 33.341030 |
| 0.0000000 | -2.2074738 | -0.4277482 | -4.2590775 | -1.2584013 | -0.9685897 | -0.1093703 | 0.0000000 | 0.0000000 | -1.0247899 | 0.0000000 | 0.0000000 | -3.0097514 | -0.2748605 | 0.0000000 | 0.0000000 | -0.1917537 | 0.0000000 | 0.0000000 | 0.0000000 | -0.2421559 | -0.7599324 | -0.3223694 | -0.5615860 | 0.0000000 | 14 | 5 | 72.06738 | 21.206657 |
| 0.0000000 | -3.3666994 | 0.0000000 | -2.0187341 | -1.4754988 | -1.7016582 | 0.0000000 | 0.0000000 | 0.0000000 | -1.0138565 | 0.0000000 | 0.0000000 | -15.6420998 | 0.0000000 | 0.0000000 | 0.0000000 | -0.3954399 | 0.0000000 | 0.0000000 | 0.0000000 | 0.0000000 | 0.0000000 | -0.9402668 | -0.4704662 | -0.4214879 | 14 | 6 | 64.42733 | 13.894568 |
| 0.0000000 | -1.0841090 | -1.8970528 | -0.0030651 | -0.0533081 | -0.7465733 | -0.4269320 | -0.0833299 | -0.5480108 | -0.0829231 | 0.0000000 | -0.1638526 | -0.3371685 | -0.1823980 | 0.0000000 | 0.0000000 | -0.1609678 | -0.2736540 | 0.0000000 | 0.0000000 | -0.0645025 | -0.2073185 | -0.2040981 | -0.0910998 | -0.1558251 | 15 | 1 | 182.48256 | 127.300993 |
| 0.0000000 | -0.6771992 | -2.0333289 | -0.5032015 | 0.0000000 | -1.2649701 | -0.3757020 | 0.0000000 | -0.1367734 | -0.2751678 | -0.0923837 | -0.9430325 | -0.0262945 | 0.0000000 | 0.0000000 | 0.0000000 | -0.1638156 | -0.1636352 | 0.0000000 | 0.0000000 | 0.0000000 | -0.1845489 | -0.3343198 | 0.0000000 | -0.2801183 | 15 | 2 | 125.26293 | 72.166201 |
| 0.1053614 | -0.9753136 | -2.9596509 | 0.0000000 | 0.0000000 | -1.4468063 | -0.0809949 | -0.1296459 | -0.4956982 | -0.1472706 | 0.0000000 | -0.4964658 | -0.8792691 | -0.8344204 | 0.0000000 | 0.0000000 | -0.5271484 | 0.0000000 | 0.0000000 | 0.0000000 | 0.0000000 | 0.0000000 | -0.5001862 | 0.0000000 | 0.0000000 | 15 | 3 | 97.63489 | 45.754930 |
| 0.5578952 | -1.7322674 | -1.2470669 | -1.4719707 | 0.0000000 | -1.6062445 | -0.5816549 | 0.0000000 | -0.5864703 | 0.0000000 | 0.0000000 | -0.9186331 | -1.4452278 | -1.0386592 | 0.0000000 | 0.0000000 | -0.4502645 | 0.0000000 | 0.0000000 | 0.0000000 | 0.0000000 | -0.9474891 | -0.5695370 | -0.0863261 | 0.0000000 | 15 | 4 | 84.22064 | 32.952729 |
| 0.5518030 | -3.2088743 | 0.0000000 | -3.5392283 | 0.0000000 | -2.2327079 | -0.3026709 | -0.3497772 | -0.4033886 | -0.5635889 | 0.0000000 | -1.1707598 | -1.9019741 | -0.5346632 | 0.0000000 | 0.0000000 | -0.0141936 | 0.0000000 | 0.0000000 | 0.0000000 | 0.0000000 | -0.8137291 | -0.7786998 | -0.2837458 | 0.0000000 | 15 | 5 | 71.89407 | 21.073715 |
| 0.0000000 | -3.0417347 | 0.0000000 | -3.7215140 | -2.2476613 | -1.9755210 | -0.4815315 | 0.0000000 | 0.0000000 | -0.6690940 | 0.0000000 | 0.0000000 | -5.5054270 | 0.0000000 | 0.0000000 | -0.5660736 | -0.4433353 | 0.0000000 | 0.0000000 | 0.0000000 | 0.0000000 | 0.0000000 | -0.1220237 | -0.7264591 | 0.0000000 | 15 | 6 | 66.76023 | 16.121326 |
| 0.0000000 | -1.2350380 | -1.8202586 | -0.1592697 | -0.0329222 | -0.8412659 | -0.1197606 | -0.2340350 | -0.4529334 | 0.0000000 | -0.1610046 | -0.2822016 | 0.0000000 | 0.0000000 | 0.0000000 | 0.0000000 | -0.3516512 | -0.1951582 | -0.0304051 | -0.0866004 | -0.0690649 | 0.0000000 | -0.2004209 | -0.2182503 | 0.0000000 | 16 | 1 | 176.10659 | 120.907288 |
| 0.0000000 | -0.6254213 | -2.6848183 | -0.1899698 | -0.1196022 | -1.2186646 | 0.0000000 | 0.0000000 | 0.0000000 | 0.0000000 | 0.0000000 | -0.6005473 | 0.0000000 | 0.0000000 | 0.0000000 | 0.0000000 | -0.0502528 | -0.2143089 | -0.0424753 | 0.0000000 | -0.0748082 | -0.1045674 | -0.5460882 | 0.0000000 | 0.0000000 | 16 | 2 | 120.21528 | 67.113330 |
| 0.0000000 | -1.3430858 | -1.7215241 | -1.4782704 | 0.0000000 | -1.6203791 | 0.0000000 | 0.0000000 | -0.4396325 | -0.2571974 | 0.0000000 | -0.9909180 | -0.5766932 | 0.0000000 | 0.0000000 | 0.0000000 | -0.1543033 | 0.0000000 | 0.0000000 | 0.0000000 | 0.0000000 | -0.1184261 | -0.6353666 | -0.0846894 | 0.0000000 | 16 | 3 | 99.41558 | 47.608874 |
| 0.0000000 | -1.4230299 | -1.2378682 | -2.9070961 | 0.0000000 | -2.1433308 | 0.0000000 | 0.0000000 | 0.0000000 | 0.0000000 | 0.0000000 | 0.0000000 | -2.1328208 | 0.0000000 | 0.0000000 | 0.0000000 | -0.0900850 | 0.0000000 | 0.0000000 | 0.0000000 | 0.0000000 | -0.4083960 | -1.0941644 | 0.0000000 | -0.2315375 | 16 | 4 | 82.68019 | 31.640722 |
| 0.0000000 | -1.9916248 | -0.2806707 | -2.2533853 | 0.0000000 | -2.6589119 | 0.0000000 | 0.0000000 | -0.1142492 | -0.5314218 | 0.0000000 | -0.4245593 | -4.2740186 | 0.0000000 | 0.0000000 | -0.4524765 | -0.0490916 | 0.0000000 | 0.0000000 | 0.0000000 | 0.0000000 | -0.0585092 | -1.0096907 | -0.1346420 | -0.3220398 | 16 | 5 | 74.97237 | 24.218674 |
| 0.0000000 | -2.0872862 | 0.0000000 | -3.6679560 | -1.9580622 | -2.3209660 | 0.0000000 | 0.0000000 | 0.0000000 | -0.9576885 | 0.0000000 | 0.0000000 | -4.8826189 | 0.0000000 | 0.0000000 | 0.0000000 | -0.3369168 | 0.0000000 | 0.0000000 | -0.1411006 | -0.6651743 | -0.7667791 | -0.4069358 | 0.0000000 | -1.7526659 | 16 | 6 | 66.93734 | 16.306201 |
| 0.0000000 | -1.0087637 | -1.6322362 | -0.4565748 | 0.0000000 | -0.9894467 | -0.2430660 | -0.0496277 | -0.4378187 | 0.0000000 | -0.0178482 | -0.3796633 | -0.1512316 | -0.0039633 | 0.0000000 | 0.0000000 | -0.2127472 | -0.1280216 | 0.0000000 | 0.0000000 | -0.0279636 | -0.1946541 | -0.3062628 | -0.1979308 | 0.0000000 | 17 | 1 | 173.84247 | 118.757142 |
| 0.0000000 | -0.8801071 | -2.5557738 | -0.1194203 | -0.4926288 | -0.7294571 | 0.0000000 | 0.0000000 | -0.3343938 | -0.4614578 | 0.0000000 | -0.1500790 | -0.5244466 | -0.2619659 | 0.0000000 | 0.0000000 | -0.1798308 | -0.3745337 | -0.0039418 | 0.0000000 | 0.0000000 | -0.1442091 | -0.2252383 | -0.2963704 | 0.0000000 | 17 | 2 | 121.00589 | 68.056442 |
| 0.0000000 | -1.4473907 | -1.8159935 | -0.8766651 | 0.0000000 | -2.3250276 | 0.0000000 | 0.0000000 | -0.1208986 | 0.0000000 | 0.0000000 | -0.7053966 | -0.8623291 | -0.3360231 | 0.0000000 | 0.0000000 | -0.2268934 | -0.4815486 | 0.0000000 | 0.0000000 | 0.0000000 | -0.5157533 | -0.2109664 | -0.1865869 | 0.0000000 | 17 | 3 | 99.05305 | 47.196534 |
| 0.0000000 | -1.2278893 | -1.5336936 | -2.5258585 | 0.0000000 | -1.3878222 | -0.2983235 | 0.0000000 | -0.5428160 | -0.7666162 | 0.0000000 | 0.0000000 | -2.5179316 | -0.3182680 | 0.0000000 | 0.0000000 | -0.7195373 | 0.0000000 | 0.0000000 | 0.0000000 | 0.0000000 | -0.0913805 | -0.4269951 | -0.2343122 | 0.0000000 | 17 | 4 | 82.12576 | 30.902152 |
| 0.0000000 | -2.5443394 | 0.0000000 | -3.3065234 | -0.1500714 | -2.6152421 | 0.0000000 | 0.0000000 | 0.0000000 | -0.2277850 | 0.0000000 | 0.0000000 | -4.3202625 | 0.0000000 | 0.0000000 | 0.0000000 | -0.4773820 | -0.0052408 | 0.0000000 | 0.0000000 | 0.0000000 | -0.4852831 | -1.0727085 | 0.0000000 | -0.1105985 | 17 | 5 | 72.17247 | 21.354051 |
| 1.6487402 | -2.4133033 | 0.0000000 | -4.1793321 | -1.2168933 | -1.2904644 | 0.0000000 | 0.0000000 | 0.0000000 | -0.7942406 | 0.0000000 | 0.0000000 | -10.7405001 | 0.0000000 | 0.0000000 | 0.0000000 | 0.0000000 | 0.0000000 | -0.3628632 | 0.0000000 | 0.0000000 | -0.3580893 | -2.6362384 | 0.0000000 | -0.1874638 | 17 | 6 | 66.15301 | 15.560275 |
| 0.0000000 | -0.9070992 | -1.9294432 | 0.0000000 | 0.0000000 | -0.9996330 | -0.3697726 | -0.1098673 | -0.4852144 | -0.1513978 | -0.0185536 | -0.1664027 | -0.3077387 | -0.1157685 | 0.0000000 | 0.0000000 | -0.1353239 | -0.2939729 | 0.0000000 | 0.0000000 | 0.0000000 | -0.2750376 | -0.2514659 | -0.0422152 | -0.1613023 | 18 | 1 | 184.68444 | 129.554853 |
| 0.0000000 | -0.8359568 | -1.9748651 | -0.8246435 | 0.0000000 | -1.1355577 | -0.2469065 | 0.0000000 | -0.5150903 | 0.0000000 | 0.0000000 | -0.5253989 | -0.1939000 | 0.0000000 | 0.0000000 | 0.0000000 | -0.1842721 | 0.0000000 | -0.0482702 | 0.0000000 | 0.0000000 | -0.2884183 | -0.4538212 | -0.0223107 | 0.0000000 | 18 | 2 | 130.20786 | 77.355317 |
| 0.0000000 | -1.1306569 | -2.3132827 | -0.2253587 | -0.3114628 | -1.0717337 | -0.6528239 | 0.0000000 | 0.0000000 | -0.4436566 | 0.0000000 | 0.0000000 | -0.8945777 | -1.1413219 | 0.0000000 | 0.0000000 | -0.7523902 | 0.0000000 | -0.1318203 | 0.0000000 | 0.0000000 | -0.2211162 | -0.5635589 | 0.0000000 | 0.0000000 | 18 | 3 | 106.63172 | 54.540535 |
| 0.1666616 | -1.2224416 | -2.6383013 | -1.0690768 | 0.0000000 | -1.8001415 | 0.0000000 | 0.0000000 | -0.1060577 | -0.5078188 | 0.0000000 | -0.5636451 | -1.5021724 | -0.3152161 | 0.0000000 | 0.0000000 | -0.2050237 | 0.0000000 | -0.1061088 | 0.0000000 | -0.0273083 | -0.1689856 | -0.5614022 | -0.0781840 | -0.3142934 | 18 | 4 | 87.54585 | 36.380471 |
| 0.0000000 | -2.8232197 | -0.3320062 | -3.1993356 | -1.1533661 | -1.6663964 | -0.1271092 | 0.0000000 | -0.0071594 | -0.0778656 | 0.0000000 | 0.0000000 | -3.7161167 | -0.8511992 | 0.0000000 | 0.0000000 | -0.1334813 | 0.0000000 | 0.0000000 | 0.0000000 | 0.0000000 | -0.6734840 | -0.9666568 | 0.0000000 | 0.0000000 | 18 | 5 | 73.49520 | 22.667575 |
| 0.0000000 | -3.2672369 | -0.9601381 | -1.3971304 | -1.9196527 | -2.1329353 | 0.0000000 | -0.2985053 | 0.0000000 | -0.3261588 | 0.0000000 | 0.0000000 | -4.7988556 | -0.4904627 | 0.0000000 | 0.0000000 | -0.2549915 | 0.0000000 | 0.0000000 | 0.0000000 | 0.0000000 | -0.1117807 | -0.5318235 | -0.3738696 | -0.4167376 | 18 | 6 | 68.82317 | 18.273864 |
| 0.0000000 | -0.7777999 | -2.0257535 | -0.1396143 | 0.0000000 | -0.7552987 | -0.2503097 | -0.1293502 | -0.4088988 | 0.0000000 | -0.1216175 | 0.0000000 | -0.2371469 | -0.0745543 | 0.0000000 | 0.0000000 | -0.1877580 | -0.3354412 | 0.0000000 | 0.0000000 | -0.2095461 | -0.0156583 | -0.3060952 | -0.0649540 | -0.0308107 | 19 | 1 | 170.91479 | 115.660476 |
| 0.0000000 | -0.6658737 | -2.2247888 | -0.5440132 | -0.5633377 | -0.5109847 | -0.4230250 | -0.0424637 | -0.5266701 | 0.0000000 | -0.0254624 | -0.7656532 | -0.1845227 | 0.0000000 | 0.0000000 | 0.0000000 | -0.1062512 | -0.2619546 | -0.1120877 | 0.0000000 | 0.0000000 | -0.0460198 | -0.1556226 | -0.2868982 | 0.0000000 | 19 | 2 | 123.15698 | 70.103919 |
| 0.0000000 | -1.0723810 | -2.2238298 | -0.2524386 | 0.0000000 | -1.8714236 | -0.3270995 | 0.0000000 | -0.2186941 | -0.1389861 | 0.0000000 | -0.5470518 | -0.8276291 | -0.4730482 | 0.0000000 | 0.0000000 | -0.0577937 | 0.0000000 | 0.0000000 | 0.0000000 | 0.0000000 | -0.1071237 | -0.7233993 | 0.0000000 | -0.4616292 | 19 | 3 | 100.54353 | 48.506658 |
| 0.0000000 | -1.8312167 | 0.0000000 | -3.7048446 | -3.0294698 | -0.0668800 | 0.0000000 | 0.0000000 | 0.0000000 | -0.7580586 | 0.0000000 | 0.0000000 | -3.9672232 | -0.7882521 | 0.0000000 | 0.0000000 | -0.3203536 | 0.0000000 | 0.0000000 | 0.0000000 | 0.0000000 | -0.5838895 | -1.3171319 | 0.0000000 | -0.0705854 | 19 | 4 | 73.49531 | 22.359208 |
| 0.0000000 | -2.4017194 | -0.1216581 | -3.9983407 | 0.0000000 | -1.8601063 | 0.0000000 | 0.0000000 | 0.0000000 | -1.5216077 | 0.0000000 | 0.0000000 | -6.5452317 | 0.0000000 | 0.0000000 | -0.0830040 | -0.2044892 | 0.0000000 | 0.0000000 | 0.0000000 | -0.1047285 | -0.7044035 | -0.8097971 | 0.0000000 | -0.0538747 | 19 | 5 | 68.89803 | 18.106983 |
| 0.0000000 | -2.3867764 | 0.0000000 | -4.2866982 | -2.0791024 | -2.3675177 | 0.0000000 | 0.0000000 | 0.0000000 | -1.4934556 | 0.0000000 | 0.0000000 | -8.4029155 | 0.0000000 | 0.0000000 | 0.0000000 | 0.0000000 | 0.0000000 | 0.0000000 | 0.0000000 | 0.0000000 | -0.5572005 | -0.5368926 | -0.4347193 | -0.3230667 | 19 | 6 | 61.82310 | 11.333274 |
| 0.0000000 | -1.1564473 | -1.7176667 | -0.4938931 | 0.0000000 | -0.7690399 | -0.2316921 | -0.0549761 | -0.3783992 | -0.0070194 | -0.0004626 | -0.4051663 | -0.0679571 | 0.0000000 | 0.0000000 | 0.0000000 | -0.1577564 | 0.0000000 | -0.2971451 | 0.0000000 | -0.0420494 | -0.2650594 | -0.3960408 | -0.0152683 | 0.0000000 | 20 | 1 | 177.05718 | 122.029393 |
| 0.0000000 | -0.9366419 | -1.8999527 | -0.7728265 | 0.0000000 | -1.0704919 | -0.3221664 | -0.0946801 | 0.0000000 | -0.2831659 | 0.0000000 | -0.0050385 | -0.6148451 | 0.0000000 | 0.0000000 | 0.0000000 | -0.3309077 | -0.0752465 | -0.0361441 | 0.0000000 | 0.0000000 | -0.3630524 | -0.6082226 | 0.0000000 | 0.0000000 | 20 | 2 | 126.34278 | 73.005233 |
| 0.0000000 | -1.1875191 | -1.9419625 | -0.9776575 | 0.0000000 | -1.6298848 | -0.2545334 | -0.1124845 | 0.0000000 | -0.2877428 | 0.0000000 | -0.4096057 | -1.1774925 | 0.0000000 | 0.0000000 | 0.0000000 | -0.2800691 | -0.0137768 | 0.0000000 | 0.0000000 | 0.0000000 | -0.2263699 | -0.6974608 | 0.0000000 | -0.2121830 | 20 | 3 | 108.39087 | 56.435776 |
| 0.3306086 | -1.6683062 | -1.6479778 | -0.7002528 | -0.1536054 | -2.0992627 | 0.0000000 | 0.0000000 | -0.6897242 | -0.1385581 | 0.0000000 | -1.8156035 | -0.8904798 | -0.0279162 | 0.0000000 | 0.0000000 | -0.1644228 | -0.2670208 | -0.0056299 | 0.0000000 | 0.0000000 | -0.5126011 | -0.1472744 | -0.4388121 | 0.0000000 | 20 | 4 | 80.88463 | 29.716965 |
| 0.0000000 | -2.4120759 | -0.1565509 | -4.4706763 | -0.5153000 | -1.4517806 | -0.0604144 | 0.0000000 | 0.0000000 | -1.3066641 | 0.0000000 | 0.0000000 | -6.0547276 | -0.5203489 | 0.0000000 | 0.0000000 | -0.1100092 | -0.6195821 | 0.0000000 | 0.0000000 | 0.0000000 | -0.6823436 | -0.5443339 | 0.0000000 | -0.2766902 | 20 | 5 | 67.59180 | 16.902144 |
| 1.3206189 | -2.2719333 | 0.0000000 | -3.7049734 | -0.7241184 | -1.6403376 | 0.0000000 | 0.0000000 | 0.0000000 | -1.0830660 | 0.0000000 | 0.0000000 | -5.1604302 | 0.0000000 | 0.0000000 | 0.0000000 | -0.0828904 | -0.7192009 | -0.1212055 | 0.0000000 | 0.0000000 | -0.5465855 | -1.9665570 | 0.0000000 | 0.0000000 | 20 | 6 | 71.51332 | 21.016488 |
| 0.0000000 | -0.7398299 | -1.6996513 | -0.3362522 | 0.0000000 | -0.7974955 | -0.2918932 | -0.1340819 | -0.5084303 | 0.0000000 | -0.1890172 | -0.2569527 | -0.0668585 | 0.0000000 | 0.0000000 | 0.0000000 | -0.1915071 | -0.2374893 | 0.0000000 | 0.0000000 | -0.2728064 | -0.0305876 | -0.2147868 | 0.0000000 | -0.3213302 | 21 | 1 | 177.22031 | 122.068546 |
| 0.0000000 | -0.9325089 | -2.0364602 | -0.3863902 | -0.6547644 | -1.0420886 | -0.2144746 | 0.0000000 | -0.4317567 | -0.0234152 | 0.0000000 | -0.4699270 | 0.0000000 | 0.0000000 | 0.0000000 | 0.0000000 | -0.3746433 | -0.3099165 | 0.0000000 | 0.0000000 | 0.0000000 | -0.3028870 | -0.0239024 | -0.2939973 | -0.0206405 | 21 | 2 | 130.57136 | 77.402862 |
| 0.0000000 | -1.4974954 | -2.4273402 | -0.6815844 | -0.0414605 | -1.4696979 | -0.0108801 | -0.1026064 | 0.0000000 | 0.0000000 | 0.0000000 | 0.0000000 | -1.4268880 | -0.5035345 | 0.0000000 | 0.0000000 | -0.2836860 | 0.0000000 | 0.0000000 | 0.0000000 | 0.0000000 | -0.8049479 | -0.4773786 | -0.1341942 | 0.0000000 | 21 | 3 | 99.70949 | 47.804664 |
| 0.0000000 | -1.8987591 | -1.0801399 | -1.7712312 | 0.0000000 | -2.6604646 | -0.1756144 | 0.0000000 | -0.0105488 | -0.3418087 | 0.0000000 | -0.1276883 | -2.2158943 | -0.6826829 | 0.0000000 | 0.0000000 | -0.5681164 | 0.0000000 | 0.0000000 | 0.0000000 | 0.0000000 | -0.7129147 | -0.4420619 | 0.0000000 | 0.0000000 | 21 | 4 | 79.23992 | 28.056589 |
| 0.0000000 | -2.2004171 | 0.0000000 | -4.2288639 | -2.3355958 | -0.6350886 | 0.0000000 | 0.0000000 | 0.0000000 | -0.0659100 | 0.0000000 | 0.0000000 | -4.9905043 | 0.0000000 | 0.0000000 | 0.0000000 | 0.0000000 | 0.0000000 | 0.0000000 | 0.0000000 | 0.0000000 | -0.2450754 | -1.7270575 | 0.0000000 | 0.0000000 | 21 | 5 | 71.01983 | 20.227903 |
| 0.3861336 | -2.2840231 | 0.0000000 | -4.3505037 | -2.8755975 | 0.0000000 | -0.5118790 | 0.0000000 | 0.0000000 | 0.0000000 | 0.0000000 | 0.0000000 | -7.2247808 | 0.0000000 | 0.0000000 | 0.0000000 | -0.5827100 | 0.0000000 | -0.3417415 | 0.0000000 | 0.0000000 | -0.9853962 | -0.8112850 | -0.5795347 | -0.2423540 | 21 | 6 | 65.66268 | 15.047116 |
| 0.0000000 | -1.0774142 | -1.9629082 | -0.1852581 | -0.0258877 | -0.5766424 | -0.2172611 | -0.2122029 | -0.3783732 | -0.1903848 | -0.0136267 | -0.3684398 | 0.0000000 | -0.2531454 | 0.0000000 | 0.0000000 | -0.1202089 | -0.0416401 | 0.0000000 | -0.0542361 | -0.1152028 | -0.1861523 | -0.2209413 | -0.1699262 | -0.1818623 | 22 | 1 | 176.57506 | 121.489085 |
| 0.0000000 | -0.7204839 | -2.1356930 | 0.0000000 | -0.0920143 | -1.0090579 | -0.1467755 | -0.1479892 | -0.5920650 | 0.0000000 | 0.0000000 | -0.0839673 | -0.4754059 | -0.5835269 | 0.0000000 | 0.0000000 | -0.3736580 | 0.0000000 | 0.0000000 | 0.0000000 | 0.0000000 | -0.2438656 | -0.5202264 | 0.0000000 | 0.0000000 | 22 | 2 | 129.67237 | 76.622542 |
| 0.0000000 | -0.5052087 | -2.4832142 | -0.6883945 | -0.6188126 | -1.4480241 | -0.2252366 | 0.0000000 | -0.0256398 | -0.2847748 | -0.0168448 | -1.4104741 | -0.0373140 | 0.0000000 | 0.0000000 | 0.0000000 | -0.4488330 | 0.0000000 | 0.0000000 | 0.0000000 | 0.0000000 | -0.4505813 | -0.4214998 | -0.0276846 | 0.0000000 | 22 | 3 | 100.91808 | 48.876124 |
| 0.0000000 | -1.8521002 | -0.9674078 | -1.8710123 | -0.7812097 | -1.5002953 | -0.2616757 | 0.0000000 | -0.2523441 | -0.0832411 | 0.0000000 | 0.0000000 | -3.5411797 | 0.0000000 | 0.0000000 | 0.0000000 | 0.0000000 | -0.1262747 | 0.0000000 | 0.0000000 | 0.0000000 | -0.4737581 | -0.9006129 | 0.0000000 | -0.4357098 | 22 | 4 | 82.90785 | 31.592133 |
| 0.0000000 | -2.8603645 | 0.0000000 | -2.3797496 | -2.9847673 | -0.4934451 | 0.0000000 | 0.0000000 | 0.0000000 | -0.5957233 | 0.0000000 | 0.0000000 | -7.0865967 | -0.6839433 | 0.0000000 | 0.0000000 | -0.5498358 | 0.0000000 | 0.0000000 | 0.0000000 | 0.0000000 | -0.1587061 | -1.1430238 | -0.3476737 | 0.0000000 | 22 | 5 | 68.47804 | 17.785979 |
| 3.1660924 | -3.8375127 | -1.4031976 | -4.4089603 | -1.7208248 | -1.1284147 | 0.0000000 | -0.4902697 | 0.0000000 | -0.2214676 | 0.0000000 | -1.2461334 | -0.9028716 | 0.0000000 | 0.0000000 | 0.0000000 | -0.3892921 | 0.0000000 | -0.4634818 | 0.0000000 | 0.0000000 | -0.3880644 | -3.3426052 | -0.0566621 | 0.0000000 | 22 | 6 | 66.98918 | 16.502559 |
| 0.0000000 | -1.3463148 | -1.7176770 | -0.0522286 | 0.0000000 | -0.9191570 | 0.0000000 | -0.0301071 | -0.5106571 | 0.0000000 | -0.1155363 | -0.1233285 | -0.6135888 | 0.0000000 | 0.0000000 | 0.0000000 | -0.0837455 | -0.1915331 | 0.0000000 | 0.0000000 | -0.2976298 | -0.0932144 | -0.3216938 | 0.0000000 | -0.1616676 | 23 | 1 | 178.87920 | 123.850719 |
| 0.0000000 | -0.6930960 | -2.2809239 | -0.0825987 | 0.0000000 | -1.5717852 | -0.0939654 | 0.0000000 | -0.2305746 | -0.0949156 | 0.0000000 | -0.2243403 | -0.2474680 | -0.1673980 | 0.0000000 | 0.0000000 | -0.3759296 | -0.1359679 | 0.0000000 | -0.0425050 | 0.0000000 | -0.2196882 | -0.4906121 | 0.0000000 | 0.0000000 | 23 | 2 | 124.74067 | 71.737567 |
| 0.0000000 | -1.3644194 | -2.4318753 | -0.6640101 | 0.0000000 | -1.6699129 | -0.1015495 | 0.0000000 | -0.6798976 | 0.0000000 | 0.0000000 | 0.0000000 | -0.4813487 | -0.9620135 | 0.0000000 | 0.0000000 | -0.0432037 | 0.0000000 | 0.0000000 | 0.0000000 | 0.0000000 | -0.0303794 | -0.6240220 | 0.0000000 | 0.0000000 | 23 | 3 | 101.52307 | 49.633180 |
| 0.0000000 | -1.5445571 | -2.2220942 | 0.0000000 | 0.0000000 | -2.5416928 | -0.2147263 | -0.0642165 | 0.0000000 | -0.6773416 | 0.0000000 | 0.0000000 | -3.9594196 | 0.0000000 | 0.0000000 | 0.0000000 | -0.1838383 | -0.0289208 | 0.0000000 | 0.0000000 | 0.0000000 | -0.4596776 | -0.5353126 | 0.0000000 | 0.0000000 | 23 | 4 | 81.98674 | 30.823979 |
| 0.0000000 | -1.9683133 | -1.2172015 | -3.5423280 | 0.0000000 | -1.3995576 | -0.1957306 | 0.0000000 | -0.3571973 | -0.0768295 | 0.0000000 | 0.0000000 | -2.6327758 | 0.0000000 | 0.0000000 | 0.0000000 | -0.2839108 | 0.0000000 | 0.0000000 | 0.0000000 | 0.0000000 | -0.1193859 | -0.9616701 | 0.0000000 | -0.4238898 | 23 | 5 | 78.53440 | 27.706552 |
| 4.1447137 | -3.7134978 | -0.9050965 | -4.2053309 | -0.8034665 | -1.1630857 | -0.0047232 | 0.0000000 | 0.0000000 | -0.9665401 | 0.0000000 | 0.0000000 | -7.0135087 | -0.0684475 | 0.0000000 | -0.0888340 | 0.0000000 | 0.0000000 | 0.0000000 | 0.0000000 | -0.2821177 | -0.2268720 | -4.9106060 | 0.0000000 | 0.0000000 | 23 | 6 | 63.91254 | 13.403275 |
| 0.0000000 | -1.0857404 | -1.7537218 | -0.1169867 | 0.0000000 | -0.7395689 | -0.2344060 | -0.0255755 | -0.6206741 | 0.0000000 | -0.0174460 | -0.4369526 | 0.0000000 | -0.4268573 | 0.0000000 | 0.0000000 | -0.0750848 | -0.2496483 | 0.0000000 | 0.0000000 | -0.0266051 | -0.1639433 | -0.2311614 | 0.0000000 | -0.3035095 | 24 | 1 | 185.30096 | 130.299349 |
| 0.0000000 | -0.7008952 | -2.7257589 | 0.0000000 | 0.0000000 | -1.1069147 | -0.0430882 | 0.0000000 | -0.1159180 | -0.1756937 | -0.2250658 | -0.2446650 | -0.8259386 | -0.1618498 | 0.0000000 | 0.0000000 | -0.2387001 | -0.1321493 | 0.0000000 | 0.0000000 | 0.0000000 | 0.0000000 | -0.4748172 | 0.0000000 | 0.0000000 | 24 | 2 | 131.42544 | 78.483082 |
| 0.0000000 | -1.0309038 | -2.7056577 | -0.7483979 | 0.0000000 | -2.1984930 | -0.1981166 | 0.0000000 | -0.1780336 | -0.0553595 | 0.0000000 | -0.1087027 | -0.9110413 | 0.0000000 | 0.0000000 | 0.0000000 | -0.5047314 | 0.0000000 | 0.0000000 | 0.0000000 | 0.0000000 | -0.4387469 | -0.3890921 | -0.1225773 | 0.0000000 | 24 | 3 | 98.02823 | 46.252077 |
| 0.0000000 | -1.7339553 | -1.4635566 | -0.4480479 | 0.0000000 | -2.5104711 | 0.0000000 | 0.0000000 | 0.0000000 | -0.6214483 | -0.1639056 | -0.0137128 | -3.3451819 | -0.5537496 | 0.0000000 | 0.0000000 | -0.8859695 | 0.0000000 | 0.0000000 | 0.0000000 | 0.0000000 | -0.7515352 | -0.4091118 | 0.0000000 | 0.0000000 | 24 | 4 | 82.18597 | 30.923552 |
| 0.0000000 | -2.9959003 | -0.0012521 | -5.3312301 | -0.1770517 | -2.5144472 | -0.1094754 | 0.0000000 | 0.0000000 | -0.6326750 | 0.0000000 | 0.0000000 | -3.9590429 | 0.0000000 | 0.0000000 | 0.0000000 | -0.6313719 | 0.0000000 | 0.0000000 | 0.0000000 | 0.0000000 | -0.4280371 | -0.8032564 | 0.0000000 | 0.0000000 | 24 | 5 | 67.42623 | 16.556352 |
| 0.0000000 | -1.8645858 | 0.0000000 | -2.6975752 | -2.7280521 | -2.7262373 | -0.6598568 | 0.0000000 | 0.0000000 | -1.2924360 | 0.0000000 | 0.0000000 | -9.0841848 | 0.0000000 | 0.0000000 | 0.0000000 | -0.5991913 | 0.0000000 | -0.4434213 | 0.0000000 | 0.0000000 | -0.4278685 | -0.2175068 | -0.3297128 | 0.0000000 | 24 | 6 | 63.03781 | 12.499327 |
| 0.0000000 | -0.8280358 | -1.6560330 | -0.3936846 | -0.1550517 | -0.7760366 | -0.3183473 | -0.0418879 | -0.4795564 | 0.0000000 | -0.0354440 | -0.5842205 | -0.0883724 | 0.0000000 | 0.0000000 | 0.0000000 | -0.0702464 | -0.3168993 | 0.0000000 | 0.0000000 | -0.1208622 | -0.2361711 | -0.2810397 | -0.0426427 | 0.0000000 | 25 | 1 | 176.58014 | 121.576625 |
| 0.0000000 | -0.6399948 | -2.0636680 | -0.4495162 | -0.1102488 | -0.9193544 | -0.3153581 | 0.0000000 | -0.0522359 | -0.6350522 | 0.0000000 | -0.7646734 | -0.0539230 | 0.0000000 | 0.0000000 | 0.0000000 | -0.1287928 | -0.1566491 | 0.0000000 | 0.0000000 | 0.0000000 | -0.2514548 | -0.5104506 | -0.0347581 | -0.1697413 | 25 | 2 | 128.05041 | 74.874828 |
| 0.0000000 | -0.8750265 | -2.1246265 | 0.0000000 | -0.2081860 | -1.9524364 | -0.2604084 | 0.0000000 | -0.3470235 | -0.1891012 | 0.0000000 | -0.3871664 | -0.6751383 | -0.7055397 | 0.0000000 | 0.0000000 | -0.0840262 | -0.0730184 | 0.0000000 | 0.0000000 | 0.0000000 | -0.5555735 | -0.4540619 | -0.1241491 | -0.1167952 | 25 | 3 | 102.69338 | 50.843334 |
| 0.0000000 | -1.8606607 | -1.0002916 | -3.3013616 | -0.0383489 | -1.8527787 | -0.1149596 | 0.0000000 | 0.0000000 | 0.0000000 | 0.0000000 | 0.0000000 | -3.2558780 | 0.0000000 | 0.0000000 | 0.0000000 | -0.5088280 | -0.0235208 | 0.0000000 | 0.0000000 | 0.0000000 | -0.4970776 | -0.8417941 | -0.1074807 | 0.0000000 | 25 | 4 | 79.06398 | 27.899473 |
| 0.0000000 | -1.5484048 | -0.2011053 | -4.0375550 | -4.4497643 | -0.6030055 | 0.0000000 | 0.0000000 | 0.0000000 | -0.6409584 | 0.0000000 | 0.0000000 | -4.4058986 | 0.0000000 | 0.0000000 | 0.0000000 | 0.0000000 | 0.0000000 | 0.0000000 | 0.0000000 | -0.1796217 | -0.4601147 | -1.3837884 | 0.0000000 | 0.0000000 | 25 | 5 | 70.36145 | 19.599994 |
| 0.0000000 | -1.8591614 | 0.0000000 | -4.6615200 | -0.7911466 | -1.4853930 | 0.0000000 | 0.0000000 | 0.0000000 | -0.6031603 | 0.0000000 | 0.0000000 | -5.3311923 | 0.0000000 | 0.0000000 | -0.2247918 | -0.0562833 | -0.0326874 | 0.0000000 | 0.0000000 | 0.0000000 | -0.7629114 | -0.8972338 | 0.0000000 | -0.9479881 | 25 | 6 | 69.68822 | 19.061166 |
| 0.0000000 | -1.1381107 | -1.9771970 | -0.2678253 | -0.3348668 | -0.6141376 | -0.2007170 | -0.0600139 | -0.4091497 | -0.1603267 | -0.1420047 | 0.0000000 | -0.4121812 | -0.0160609 | 0.0000000 | 0.0000000 | -0.3040115 | -0.2798555 | 0.0000000 | 0.0000000 | -0.0859632 | -0.0932166 | -0.1953549 | -0.1653331 | -0.0294587 | 26 | 1 | 179.68122 | 124.524156 |
| 0.0000000 | -0.8355540 | -2.0862220 | -0.2434870 | 0.0000000 | -1.6915655 | -0.1875689 | 0.0000000 | -0.0823002 | -0.1625057 | 0.0000000 | -0.5382611 | -0.1245478 | 0.0000000 | 0.0000000 | 0.0000000 | -0.1309345 | -0.0098474 | 0.0000000 | 0.0000000 | 0.0000000 | -0.3118920 | -0.6073680 | -0.0126676 | 0.0000000 | 26 | 2 | 122.47383 | 69.276675 |
| 0.0000000 | -1.3925353 | -2.5953463 | -0.8862277 | -0.2772100 | -1.7330871 | 0.0000000 | 0.0000000 | -0.1581402 | -0.2425443 | -0.0133976 | -0.9822795 | -0.4814759 | 0.0000000 | 0.0000000 | 0.0000000 | -0.1698687 | -0.0628108 | 0.0000000 | 0.0000000 | 0.0000000 | -0.2396584 | -0.4139361 | -0.1156251 | -0.1190166 | 26 | 3 | 96.11884 | 44.364238 |
| 0.0000000 | -1.9004543 | -0.0972489 | -3.1787622 | 0.0000000 | -1.7317249 | -0.1158340 | 0.0000000 | 0.0000000 | -0.5441302 | 0.0000000 | 0.0000000 | -3.3840058 | -0.0759971 | 0.0000000 | 0.0000000 | -0.1282158 | 0.0000000 | -0.0041177 | 0.0000000 | 0.0000000 | -0.9848006 | -0.6464102 | -0.4917575 | -0.1726417 | 26 | 4 | 85.10519 | 33.877423 |
| 0.9775617 | -2.4754578 | 0.0000000 | -4.2206751 | -3.1633961 | 0.0000000 | 0.0000000 | 0.0000000 | 0.0000000 | 0.0000000 | 0.0000000 | 0.0000000 | -5.6944652 | 0.0000000 | 0.0000000 | 0.0000000 | -0.1950305 | 0.0000000 | -0.0900349 | 0.0000000 | 0.0000000 | -0.5826050 | -2.2542743 | -0.0477775 | -0.8450691 | 26 | 5 | 71.58710 | 20.791808 |
| 0.0000000 | -2.7154963 | 0.0000000 | -4.1425073 | -3.7900559 | -0.6500301 | -0.4268522 | 0.0000000 | 0.0000000 | -1.6586586 | 0.0000000 | 0.0000000 | -7.8658753 | 0.0000000 | 0.0000000 | 0.0000000 | 0.0000000 | 0.0000000 | 0.0000000 | 0.0000000 | 0.0000000 | -0.6994310 | -0.6542916 | -0.2067925 | 0.0000000 | 26 | 6 | 66.31295 | 15.773679 |
| 0.0000000 | -0.6432182 | -2.0642108 | 0.0000000 | 0.0000000 | -0.4845067 | -0.4607393 | -0.1286389 | -0.5474368 | 0.0000000 | -0.1420751 | -0.2895832 | -0.2475928 | -0.3139196 | 0.0000000 | 0.0000000 | -0.1479858 | -0.2432482 | -0.0636612 | 0.0000000 | -0.0757094 | -0.0593466 | -0.1548124 | -0.1270505 | 0.0000000 | 27 | 1 | 174.20979 | 118.947164 |
| 0.0000000 | -1.0269384 | -2.3869186 | 0.0000000 | 0.0000000 | -1.0470422 | -0.3403919 | -0.0040946 | 0.0000000 | -0.2124166 | 0.0000000 | -0.6551392 | -0.2516153 | -0.1974365 | 0.0000000 | 0.0000000 | -0.1923841 | -0.0366601 | -0.1050751 | 0.0000000 | 0.0000000 | -0.1686942 | -0.3778481 | -0.1809490 | 0.0000000 | 27 | 2 | 129.99397 | 76.846152 |
| 0.0000000 | -1.2004715 | -2.0685812 | -1.0498942 | -0.1838781 | -1.2076596 | -0.5306967 | 0.0000000 | -0.1121086 | -0.2399968 | 0.0000000 | -0.0638496 | -1.9990853 | 0.0000000 | 0.0000000 | 0.0000000 | -0.3864302 | 0.0000000 | 0.0000000 | 0.0000000 | 0.0000000 | -0.4217301 | -0.5080402 | -0.1698064 | -0.4785530 | 27 | 3 | 102.67014 | 50.621546 |
| 0.0000000 | -1.5239215 | -1.9805164 | -2.1043123 | -0.6678233 | -0.8742983 | 0.0000000 | 0.0000000 | -0.3059968 | -0.5837627 | 0.0000000 | 0.0000000 | -3.0833736 | -0.4501173 | 0.0000000 | 0.0000000 | -0.3369384 | 0.0000000 | 0.0000000 | 0.0000000 | 0.0000000 | -0.6463475 | -0.5269760 | 0.0000000 | -0.1564299 | 27 | 4 | 80.05288 | 28.806769 |
| 0.0000000 | -2.4220437 | -1.4951999 | -1.3977978 | 0.0000000 | -2.9141434 | 0.0000000 | 0.0000000 | 0.0000000 | -1.6647687 | 0.0000000 | 0.0000000 | -1.6571978 | -1.1651987 | 0.0000000 | 0.0000000 | -0.4220797 | 0.0000000 | 0.0000000 | 0.0000000 | 0.0000000 | -0.9480722 | -0.0793255 | 0.0000000 | -0.7742131 | 27 | 5 | 76.84635 | 25.959596 |
| 0.6659539 | -2.6383765 | -1.3091382 | -3.1519323 | 0.0000000 | -1.3409415 | 0.0000000 | 0.0000000 | 0.0000000 | -0.0751646 | -0.2111479 | 0.0000000 | -9.0213298 | 0.0000000 | 0.0000000 | 0.0000000 | 0.0000000 | 0.0000000 | 0.0000000 | 0.0000000 | 0.0000000 | -0.2162276 | -1.5775565 | -0.0651554 | 0.0000000 | 27 | 6 | 64.16637 | 13.695538 |
| 0.0000000 | -0.9056610 | -2.1163024 | 0.0000000 | -0.0846440 | -0.9362336 | -0.2572615 | -0.0657407 | -0.4232718 | 0.0000000 | -0.0715292 | -0.3447824 | -0.2917442 | -0.0812485 | 0.0000000 | 0.0000000 | -0.2580564 | -0.1471388 | 0.0000000 | 0.0000000 | 0.0000000 | -0.2196653 | -0.2610792 | -0.0182849 | -0.1288016 | 28 | 1 | 168.81894 | 113.983692 |
| 0.0000000 | -0.5913589 | -2.8250736 | -0.4056290 | -0.1249754 | -0.7597696 | -0.2158012 | 0.0000000 | -0.1711482 | -0.2844896 | 0.0000000 | -0.4297210 | -0.2304476 | 0.0000000 | 0.0000000 | 0.0000000 | -0.1502092 | -0.3184418 | -0.0121498 | 0.0000000 | 0.0000000 | -0.1386065 | -0.3400324 | -0.0955276 | 0.0000000 | 28 | 2 | 127.78487 | 74.780588 |
| 0.0000000 | -1.0497552 | -2.1196888 | -1.3590098 | -0.1010547 | -1.3300472 | -0.4140658 | 0.0000000 | 0.0000000 | -0.4865811 | 0.0000000 | 0.0000000 | -1.4832978 | -0.6557035 | 0.0000000 | 0.0000000 | -0.0594052 | -0.2226359 | 0.0000000 | 0.0000000 | 0.0000000 | -0.3427785 | -0.4107913 | -0.5013178 | 0.0000000 | 28 | 3 | 95.05494 | 43.129053 |
| 0.0000000 | -1.8884633 | -0.1136167 | -4.2717553 | -1.3676017 | -1.2718199 | 0.0000000 | 0.0000000 | 0.0000000 | -0.4369962 | 0.0000000 | 0.0000000 | -2.4794920 | -0.3507912 | 0.0000000 | 0.0000000 | -0.0497698 | 0.0000000 | 0.0000000 | 0.0000000 | -0.0675821 | -1.0680670 | -1.0047908 | -0.1879912 | 0.0000000 | 28 | 4 | 72.84319 | 21.674357 |
| 0.0000000 | -2.5982120 | 0.0000000 | -4.3851473 | -2.2146820 | -0.5270467 | 0.0000000 | 0.0000000 | -0.2439212 | 0.0000000 | 0.0000000 | 0.0000000 | -5.3260763 | -0.3719486 | 0.0000000 | 0.0000000 | -0.1295095 | -0.1038792 | 0.0000000 | 0.0000000 | 0.0000000 | -0.4961353 | -0.9643984 | -0.2571836 | -0.2583347 | 28 | 5 | 73.73127 | 22.915037 |
| 0.6900119 | -3.7235579 | 0.0000000 | -5.7289306 | -0.5087013 | -3.5116816 | -0.8892528 | -0.0538829 | 0.0000000 | -1.4276282 | 0.0000000 | 0.0000000 | -11.2113148 | 0.0000000 | 0.0000000 | 0.0000000 | -1.2234904 | 0.0000000 | 0.0000000 | 0.0000000 | 0.0000000 | -0.8354871 | 0.0000000 | -0.1682945 | -0.5381002 | 28 | 6 | 59.87251 | 9.378667 |
| 0.0000000 | -0.8955962 | -1.7193862 | -0.4906809 | -0.1803672 | -0.4969619 | -0.4659470 | 0.0000000 | -0.6180128 | -0.0216289 | -0.0503191 | -0.2896362 | -0.1279048 | -0.0922911 | 0.0000000 | 0.0000000 | -0.0082031 | -0.1909176 | 0.0000000 | 0.0000000 | 0.0000000 | -0.0589626 | -0.3655251 | -0.1790536 | -0.0618578 | 29 | 1 | 164.06018 | 108.782165 |
| 0.0000000 | -0.8580712 | -2.2305922 | -0.5292896 | 0.0000000 | -1.0470676 | -0.1096392 | 0.0000000 | 0.0000000 | -0.2272556 | -0.0004032 | -0.2383716 | -0.7840906 | 0.0000000 | 0.0000000 | 0.0000000 | -0.3639389 | 0.0000000 | 0.0000000 | 0.0000000 | -0.0835532 | -0.3013734 | -0.6090416 | 0.0000000 | 0.0000000 | 29 | 2 | 124.96300 | 71.981592 |
| 0.0000000 | -1.2854583 | -2.3032660 | -0.1139239 | 0.0000000 | -2.1037978 | -0.3429368 | 0.0000000 | -0.3848233 | 0.0000000 | 0.0000000 | 0.0000000 | -3.1277748 | 0.0000000 | 0.0000000 | 0.0000000 | -0.0894873 | 0.0000000 | 0.0000000 | 0.0000000 | 0.0000000 | -0.3852896 | -0.5026913 | 0.0000000 | -0.4422714 | 29 | 3 | 97.36764 | 45.645299 |
| 0.0000000 | -1.0734698 | -0.5708419 | -2.7721325 | -0.1388904 | -1.8257568 | -0.1292951 | 0.0000000 | 0.0000000 | -0.0620376 | 0.0000000 | 0.0000000 | -2.2461505 | -0.3173975 | 0.0000000 | 0.0000000 | -0.4086088 | 0.0000000 | 0.0000000 | 0.0000000 | 0.0000000 | -0.7698525 | -0.8037933 | -0.4455002 | 0.0000000 | 29 | 4 | 83.49037 | 32.303469 |
| 0.0000000 | -2.1904942 | 0.0000000 | -2.9737770 | -2.3959704 | -1.4652536 | 0.0000000 | 0.0000000 | -0.1137018 | -0.3447063 | 0.0000000 | 0.0000000 | -7.2921528 | 0.0000000 | 0.0000000 | 0.0000000 | -0.0064485 | 0.0000000 | 0.0000000 | 0.0000000 | 0.0000000 | -0.5687347 | -0.9128550 | -0.4089559 | -0.2361324 | 29 | 5 | 68.22618 | 17.376007 |
| 0.0000000 | -2.2488269 | 0.0000000 | -4.0055623 | -2.0273753 | -1.4833702 | 0.0000000 | 0.0000000 | 0.0000000 | -0.0018794 | 0.0000000 | 0.0000000 | -6.3104217 | 0.0000000 | 0.0000000 | 0.0000000 | 0.0000000 | 0.0000000 | 0.0000000 | 0.0000000 | -0.0692886 | -0.1959689 | -1.4424075 | 0.0000000 | 0.0000000 | 29 | 6 | 67.95451 | 17.384459 |
| 0.0000000 | -0.7954303 | -2.0524814 | -0.0720211 | -0.0034964 | -0.9381155 | -0.1621713 | -0.0145866 | -0.3875012 | 0.0000000 | -0.0656182 | -0.2502727 | -0.0539629 | 0.0000000 | 0.0000000 | 0.0000000 | -0.1784556 | -0.1888617 | -0.1044047 | -0.0916221 | -0.0204698 | -0.1246981 | -0.3625555 | 0.0000000 | -0.0622319 | 30 | 1 | 175.39366 | 120.218519 |
| 0.0000000 | -0.9971266 | -1.9604776 | -0.5758546 | 0.0000000 | -1.4099113 | -0.0428336 | 0.0000000 | -0.2523604 | -0.1327415 | 0.0000000 | -0.6110204 | -0.0402822 | 0.0000000 | 0.0000000 | 0.0000000 | -0.3580839 | -0.1169220 | 0.0000000 | 0.0000000 | -0.0359828 | -0.3691108 | -0.3797012 | 0.0000000 | 0.0000000 | 30 | 2 | 129.70415 | 76.630118 |
| 0.0000000 | -1.1309796 | -2.6439013 | 0.0000000 | 0.0000000 | -2.4111251 | -0.4604128 | 0.0000000 | -0.4600135 | 0.0000000 | 0.0000000 | -0.2897044 | -1.1589820 | -0.6154580 | 0.0000000 | 0.0000000 | -0.1051936 | 0.0000000 | 0.0000000 | 0.0000000 | 0.0000000 | -0.4007222 | 0.0000000 | -0.4188833 | 0.0000000 | 30 | 3 | 96.09359 | 44.389216 |
| 0.0000000 | -1.2877012 | -2.4011802 | -1.9033768 | -1.4696190 | -0.2947984 | -0.4479066 | 0.0000000 | 0.0000000 | -0.4533683 | 0.0000000 | 0.0000000 | -0.8646497 | -1.1195402 | 0.0000000 | 0.0000000 | -0.0403363 | 0.0000000 | 0.0000000 | 0.0000000 | 0.0000000 | -0.4176471 | -0.1036784 | -0.8251474 | 0.0000000 | 30 | 4 | 88.59876 | 37.255291 |
| 0.0000000 | -2.5738864 | 0.0000000 | -2.5245570 | -1.6109292 | -1.6576284 | 0.0000000 | 0.0000000 | -0.0983511 | 0.0000000 | 0.0000000 | 0.0000000 | -5.4257000 | 0.0000000 | 0.0000000 | 0.0000000 | -0.2767334 | -0.0774593 | 0.0000000 | 0.0000000 | 0.0000000 | -0.8231814 | -0.8059180 | -0.4034182 | -0.5268267 | 30 | 5 | 72.49257 | 21.715844 |
| 0.1001307 | -3.1422571 | 0.0000000 | -2.3979290 | -2.0789915 | -1.2362874 | 0.0000000 | 0.0000000 | 0.0000000 | -0.0620598 | 0.0000000 | 0.0000000 | -7.4001431 | -0.8869838 | 0.0000000 | 0.0000000 | 0.0000000 | 0.0000000 | 0.0000000 | 0.0000000 | 0.0000000 | -0.8393535 | -1.1652847 | 0.0000000 | 0.0000000 | 30 | 6 | 67.20941 | 16.531754 |
| 0.0000000 | -1.1885846 | -1.8770066 | -0.1554441 | -0.2149582 | -0.7833508 | -0.2423018 | -0.1355629 | -0.1507691 | -0.0627515 | -0.0630402 | -0.2598035 | -0.2864316 | 0.0000000 | 0.0000000 | 0.0000000 | -0.2353328 | -0.3233940 | 0.0000000 | 0.0000000 | -0.1306352 | -0.1812037 | -0.2784445 | -0.0709985 | 0.0000000 | 31 | 1 | 183.81065 | 128.872936 |
| 0.0000000 | -1.2242205 | -2.1898823 | -0.2997455 | 0.0000000 | -1.2365690 | -0.4027423 | -0.0649044 | -0.3055738 | -0.0248437 | 0.0000000 | -0.4126493 | 0.0000000 | -0.2633335 | 0.0000000 | 0.0000000 | -0.2470593 | 0.0000000 | 0.0000000 | 0.0000000 | 0.0000000 | -0.2300772 | -0.4956803 | 0.0000000 | 0.0000000 | 31 | 2 | 135.14880 | 82.223926 |
| 0.2403665 | -0.9201190 | -2.7602067 | -0.2485320 | 0.0000000 | -1.7705257 | -0.6328766 | 0.0000000 | -0.0648396 | -0.2942461 | 0.0000000 | 0.0000000 | -2.0001478 | -0.1957626 | -0.1689162 | 0.0000000 | -0.4075764 | -0.1623174 | 0.0000000 | 0.0000000 | 0.0000000 | -0.5898648 | -0.0235561 | -0.6192210 | 0.0000000 | 31 | 3 | 100.26157 | 48.443650 |
| 0.0000000 | -2.1126402 | 0.0000000 | -3.8638025 | -3.1081230 | -0.7086973 | 0.0000000 | 0.0000000 | 0.0000000 | -0.1137181 | 0.0000000 | 0.0000000 | -5.0458702 | 0.0000000 | 0.0000000 | 0.0000000 | -0.2399356 | 0.0000000 | 0.0000000 | 0.0000000 | 0.0000000 | -0.4523384 | -1.3461435 | 0.0000000 | 0.0000000 | 31 | 4 | 74.51037 | 23.139108 |
| 0.0000000 | -3.1429055 | 0.0000000 | -4.8082540 | -1.0160962 | -1.1877234 | -0.2885742 | 0.0000000 | -0.1163791 | -0.1339530 | 0.0000000 | 0.0000000 | -3.8954695 | -0.1935522 | 0.0000000 | 0.0000000 | -0.1475835 | 0.0000000 | 0.0000000 | 0.0000000 | 0.0000000 | -0.7077857 | -0.9283827 | 0.0000000 | 0.0000000 | 31 | 5 | 71.38959 | 20.591465 |
| 0.0000000 | -3.2772317 | 0.0000000 | -6.1449851 | -2.8239775 | 0.0000000 | 0.0000000 | 0.0000000 | 0.0000000 | -1.7473967 | 0.0000000 | 0.0000000 | -6.2218622 | 0.0000000 | 0.0000000 | 0.0000000 | -0.7209866 | 0.0000000 | 0.0000000 | 0.0000000 | 0.0000000 | 0.0000000 | -0.8142162 | -0.3410283 | 0.0000000 | 31 | 6 | 63.98938 | 13.426829 |
| 0.0000000 | -0.9183553 | -1.8667381 | -0.3275832 | -0.2057248 | -0.7594148 | -0.3381357 | -0.2556714 | -0.1619236 | -0.2854324 | -0.1087544 | -0.1686139 | -0.2707367 | -0.0350110 | 0.0000000 | 0.0000000 | -0.0514931 | -0.0359154 | -0.1639028 | 0.0000000 | -0.0652047 | -0.1985911 | -0.3758012 | -0.0927113 | 0.0000000 | 32 | 1 | 175.33260 | 120.298285 |
| 0.0000000 | -0.9307569 | -2.0016817 | -0.8334665 | 0.0000000 | -1.2209082 | -0.2292932 | -0.1490129 | -0.3557712 | 0.0000000 | -0.2708571 | -0.0152017 | -0.5031968 | 0.0000000 | 0.0000000 | 0.0000000 | -0.2559923 | 0.0000000 | -0.1080721 | 0.0000000 | 0.0000000 | -0.2137332 | -0.3983034 | -0.0839277 | 0.0000000 | 32 | 2 | 127.65242 | 74.465551 |
| 0.2259715 | -1.0575215 | -2.2705647 | -0.5781919 | 0.0000000 | -2.0958761 | 0.0000000 | -0.0530474 | 0.0000000 | -0.0001383 | 0.0000000 | -0.9533009 | -0.5600445 | 0.0000000 | 0.0000000 | 0.0000000 | -0.4063668 | -0.0744207 | 0.0000000 | 0.0000000 | 0.0000000 | -0.4934287 | -0.6545367 | 0.0000000 | -0.0652808 | 32 | 3 | 99.09175 | 47.132093 |
| 0.0000000 | -1.8030621 | -1.3462826 | -1.8298368 | 0.0000000 | -1.1649590 | 0.0000000 | 0.0000000 | 0.0000000 | -0.9109922 | 0.0000000 | -0.1480340 | -3.2760968 | -0.4388226 | 0.0000000 | -0.0495177 | -0.1957872 | -0.0167575 | -0.0762115 | 0.0000000 | 0.0000000 | -0.5501123 | -0.7513804 | -0.1597556 | 0.0000000 | 32 | 4 | 84.36900 | 33.178335 |
| 0.0000000 | -2.2286277 | 0.0000000 | -2.8320757 | -0.7352096 | -1.6940122 | -0.4646903 | 0.0000000 | -0.1053112 | -0.2654482 | 0.0000000 | 0.0000000 | -6.6460612 | 0.0000000 | 0.0000000 | 0.0000000 | -0.1884170 | -0.4269001 | 0.0000000 | 0.0000000 | 0.0000000 | -0.4837108 | -0.7692539 | 0.0000000 | -0.4482054 | 32 | 5 | 71.48764 | 20.645157 |
| 0.0000000 | -2.7301896 | 0.0000000 | -5.9943414 | -2.3214990 | -0.5039156 | 0.0000000 | 0.0000000 | 0.0000000 | -1.1732719 | 0.0000000 | 0.0000000 | -4.8212795 | 0.0000000 | 0.0000000 | 0.0000000 | -0.1133759 | -0.4491840 | 0.0000000 | 0.0000000 | 0.0000000 | 0.0000000 | -1.0788710 | 0.0000000 | 0.0000000 | 32 | 6 | 66.45178 | 15.834295 |
| 0.0000000 | -0.9928094 | -2.0946053 | -0.1158175 | -0.2473880 | -0.5663939 | -0.2918095 | -0.1228021 | -0.1803559 | -0.2597591 | -0.2258350 | -0.2555624 | 0.0000000 | -0.1877090 | 0.0000000 | 0.0000000 | -0.3311085 | -0.2062680 | 0.0000000 | 0.0000000 | -0.1580062 | 0.0000000 | -0.2296186 | -0.0056028 | -0.5787084 | 33 | 1 | 174.09470 | 119.064586 |
| 0.0000000 | -0.7592070 | -2.4240214 | 0.0000000 | 0.0000000 | -1.1874708 | -0.5281701 | -0.2147929 | 0.0000000 | -0.3937795 | 0.0000000 | -0.3557987 | -0.2573332 | -0.4858287 | 0.0000000 | 0.0000000 | -0.3216128 | -0.2207607 | -0.0589879 | 0.0000000 | 0.0000000 | -0.2462814 | -0.1884687 | -0.0830274 | 0.0000000 | 33 | 2 | 126.32152 | 73.436508 |
| 0.0000000 | -0.5773923 | -2.6276643 | 0.0000000 | 0.0000000 | -1.5295859 | -0.1504818 | -0.1846613 | -0.2708402 | 0.0000000 | -0.0253940 | -0.2062296 | -0.9904964 | -0.5829311 | 0.0000000 | 0.0000000 | -0.3064940 | -0.0890184 | 0.0000000 | 0.0000000 | 0.0000000 | -0.2062628 | -0.7049713 | 0.0000000 | 0.0000000 | 33 | 3 | 108.32309 | 56.452377 |
| 0.0000000 | -1.2476819 | -0.9999924 | -1.7263561 | -0.9810830 | -0.9106651 | 0.0000000 | 0.0000000 | 0.0000000 | -0.7261963 | -0.1083118 | 0.0000000 | -3.3783788 | -0.2953363 | 0.0000000 | 0.0000000 | 0.0000000 | -0.0589756 | 0.0000000 | 0.0000000 | 0.0000000 | -0.7200417 | -1.0265429 | 0.0000000 | -0.0620174 | 33 | 4 | 83.93577 | 32.651637 |
| 0.0000000 | -2.6220632 | 0.0000000 | -3.8076296 | -2.0249630 | -1.0904147 | 0.0000000 | 0.0000000 | 0.0000000 | -0.4174658 | 0.0000000 | 0.0000000 | -3.6799453 | -0.6776638 | 0.0000000 | 0.0000000 | 0.0000000 | 0.0000000 | -0.0617156 | 0.0000000 | 0.0000000 | -0.6173866 | -0.8580128 | -0.4393805 | -0.0245637 | 33 | 5 | 73.46938 | 22.569042 |
| 0.0000000 | -3.6658237 | 0.0000000 | -2.6282605 | -2.0343564 | -1.5486501 | 0.0000000 | 0.0000000 | 0.0000000 | -0.3074407 | 0.0000000 | 0.0000000 | -4.5773207 | -0.5485543 | 0.0000000 | 0.0000000 | -0.1741355 | 0.0000000 | 0.0000000 | 0.0000000 | -0.7415059 | -0.3605108 | -0.4290398 | -0.4844126 | -1.3674510 | 33 | 6 | 68.24048 | 17.737693 |
| 0.0000000 | -0.9399494 | -1.9554577 | -0.3207971 | -0.2207166 | -0.5521226 | -0.1097525 | 0.0000000 | -0.3436867 | -0.0329094 | -0.0362083 | -0.4910403 | -0.2504873 | 0.0000000 | 0.0000000 | 0.0000000 | -0.1648149 | -0.0759381 | 0.0000000 | 0.0000000 | -0.0231919 | -0.1733445 | -0.4600341 | 0.0000000 | -0.0154447 | 34 | 1 | 175.85008 | 120.780691 |
| 0.0000000 | -0.8830618 | -1.9598586 | -0.4519881 | 0.0000000 | -1.3622713 | -0.1676538 | 0.0000000 | -0.4544503 | -0.0469924 | -0.1336160 | -0.6714631 | -0.1188963 | 0.0000000 | 0.0000000 | 0.0000000 | -0.1645571 | 0.0000000 | -0.0989588 | 0.0000000 | 0.0000000 | -0.3006260 | -0.3371894 | 0.0000000 | 0.0000000 | 34 | 2 | 130.65880 | 77.655301 |
| 0.0354736 | -1.1598985 | -1.8113122 | -1.0213571 | 0.0000000 | -1.6427408 | 0.0000000 | 0.0000000 | 0.0000000 | -0.0848887 | -0.0931982 | 0.0000000 | -1.1499589 | -0.8188872 | 0.0000000 | 0.0000000 | -0.2685397 | 0.0000000 | 0.0000000 | 0.0000000 | 0.0000000 | -0.2609884 | -0.8165868 | -0.1373164 | -0.1383766 | 34 | 3 | 103.11364 | 51.176353 |
| 0.0000000 | -2.1932602 | -0.0360837 | -3.0709887 | 0.0000000 | -1.6493005 | -0.2234984 | 0.0000000 | 0.0000000 | -1.2770400 | 0.0000000 | -0.2641266 | -3.9646946 | 0.0000000 | 0.0000000 | -0.0227159 | 0.0000000 | 0.0000000 | 0.0000000 | 0.0000000 | 0.0000000 | -0.9667732 | -0.6585480 | -0.2969230 | 0.0000000 | 34 | 4 | 78.57451 | 27.415561 |
| 0.0000000 | -3.1538127 | 0.0000000 | -4.1174579 | -2.6475022 | -0.8419380 | -0.1053422 | 0.0000000 | 0.0000000 | -0.7012029 | 0.0000000 | 0.0000000 | -4.1204922 | 0.0000000 | 0.0000000 | 0.0000000 | 0.0000000 | 0.0000000 | 0.0000000 | 0.0000000 | 0.0000000 | -0.6741643 | -0.8069018 | -0.4058369 | -0.1675641 | 34 | 5 | 70.08371 | 19.237822 |
| 0.0000000 | -2.9225106 | 0.0000000 | -4.9580250 | -3.3256995 | -0.9722795 | 0.0000000 | 0.0000000 | 0.0000000 | -0.4036215 | 0.0000000 | 0.0000000 | -7.6899297 | 0.0000000 | 0.0000000 | 0.0000000 | -0.6599550 | 0.0000000 | -0.0711667 | 0.0000000 | 0.0000000 | -0.6624367 | -0.7395832 | 0.0000000 | -0.6662162 | 34 | 6 | 61.86865 | 11.367230 |
| 0.0000000 | -0.8228809 | -2.1149975 | -0.1197807 | 0.0000000 | -0.8555371 | -0.1898651 | 0.0000000 | -0.3276954 | -0.2208177 | -0.0729164 | -0.3078069 | -0.1670906 | 0.0000000 | 0.0000000 | 0.0000000 | -0.1543087 | -0.2332588 | 0.0000000 | 0.0000000 | -0.1110821 | -0.1640119 | -0.2486449 | -0.0450400 | -0.1766889 | 35 | 1 | 182.96340 | 127.843180 |
| 0.0000000 | -0.6050036 | -2.3239033 | 0.0000000 | 0.0000000 | -1.0316101 | -0.2467620 | 0.0000000 | -0.2550325 | 0.0000000 | 0.0000000 | -0.4279764 | 0.0000000 | -0.5350563 | 0.0000000 | 0.0000000 | -0.1597716 | -0.1097384 | 0.0000000 | 0.0000000 | 0.0000000 | -0.2861540 | -0.5223683 | 0.0000000 | -0.0385388 | 35 | 2 | 126.71693 | 73.503781 |
| 0.0000000 | -0.7272846 | -2.8616928 | -0.7919449 | 0.0000000 | -1.0221349 | -0.2773854 | 0.0000000 | 0.0000000 | 0.0000000 | 0.0000000 | -0.1375869 | -1.7278708 | -0.3952141 | 0.0000000 | 0.0000000 | -0.2829119 | -0.2549148 | 0.0000000 | 0.0000000 | 0.0000000 | -0.4410508 | -0.4754276 | 0.0000000 | -0.0548963 | 35 | 3 | 104.66698 | 52.881351 |
| 0.0069632 | -2.1865706 | -0.5684285 | -1.9235854 | -0.4077629 | -1.4942627 | 0.0000000 | 0.0000000 | 0.0000000 | -0.1344143 | 0.0000000 | 0.0000000 | -2.1313421 | -1.4026942 | 0.0000000 | 0.0000000 | -0.3991870 | 0.0000000 | -0.2845441 | 0.0000000 | 0.0000000 | -0.6725685 | -0.6514133 | -0.3545757 | -0.0066677 | 35 | 4 | 84.55388 | 33.202503 |
| 0.0000000 | -1.9904638 | 0.0000000 | -5.2147384 | -0.1626688 | -0.6034091 | -0.0282488 | 0.0000000 | -0.4489230 | 0.0000000 | 0.0000000 | 0.0000000 | -5.5824693 | 0.0000000 | 0.0000000 | 0.0000000 | -0.0976442 | 0.0000000 | 0.0000000 | 0.0000000 | 0.0000000 | -0.7316801 | -0.8484184 | -0.4757670 | 0.0000000 | 35 | 5 | 72.85890 | 22.061356 |
| 0.5842237 | -2.8099845 | 0.0000000 | -3.2193313 | 0.0000000 | -1.7498947 | 0.0000000 | 0.0000000 | 0.0000000 | -0.4553091 | 0.0000000 | 0.0000000 | -7.6888243 | 0.0000000 | 0.0000000 | 0.0000000 | -0.5074542 | 0.0000000 | 0.0000000 | 0.0000000 | 0.0000000 | -0.7431389 | -1.5186643 | 0.0000000 | 0.0000000 | 35 | 6 | 68.56524 | 18.016727 |
| 0.0000000 | -1.0686107 | -2.0468874 | -0.0935384 | -0.1332470 | -0.7574057 | -0.2357532 | 0.0000000 | -0.3559697 | 0.0000000 | -0.2136491 | -0.4699493 | 0.0000000 | -0.1086729 | 0.0000000 | 0.0000000 | -0.2252221 | -0.3402710 | -0.0791792 | 0.0000000 | 0.0000000 | -0.0801115 | -0.1351824 | -0.1218208 | -0.0731989 | 36 | 1 | 176.07374 | 120.939530 |
| 0.0000000 | -0.9242408 | -1.8140052 | -0.0857257 | -0.0943972 | -1.2342044 | -0.0355733 | 0.0000000 | -0.0795921 | -0.0269671 | -0.0505139 | -0.6802409 | -0.1503138 | -0.1912135 | 0.0000000 | 0.0000000 | -0.1894126 | -0.0791147 | 0.0000000 | 0.0000000 | 0.0000000 | -0.3128610 | -0.6498190 | 0.0000000 | 0.0000000 | 36 | 2 | 133.44130 | 80.313231 |
| 0.0000000 | -1.4892374 | -1.4601725 | -1.5459877 | -0.1506941 | -1.2339872 | 0.0000000 | 0.0000000 | -0.3261401 | 0.0000000 | 0.0000000 | 0.0000000 | -2.4060619 | 0.0000000 | 0.0000000 | 0.0000000 | -0.4420399 | 0.0000000 | -0.0427846 | 0.0000000 | 0.0000000 | -0.5261196 | -0.6272205 | -0.1139797 | 0.0000000 | 36 | 3 | 100.08652 | 48.108678 |
| 0.0000000 | -1.2588505 | -1.9756577 | -1.5455420 | -1.5016899 | -1.3056577 | 0.0000000 | 0.0000000 | 0.0000000 | -0.2199960 | 0.0000000 | -1.5828117 | -0.0351692 | 0.0000000 | 0.0000000 | 0.0000000 | -0.3574905 | 0.0000000 | 0.0000000 | 0.0000000 | 0.0000000 | -0.6398749 | -0.2428453 | -0.3488232 | 0.0000000 | 36 | 4 | 84.86463 | 33.604326 |
| 0.1417436 | -2.4942238 | 0.0000000 | -6.0753930 | -3.8102533 | -0.1198459 | 0.0000000 | 0.0000000 | 0.0000000 | -0.3456673 | 0.0000000 | 0.0000000 | -4.1516019 | 0.0000000 | 0.0000000 | 0.0000000 | -0.2851689 | 0.0000000 | 0.0000000 | 0.0000000 | 0.0000000 | -0.7949924 | -1.2734830 | 0.0000000 | -0.5311729 | 36 | 5 | 66.16422 | 15.340883 |
| 0.6486029 | -3.2248775 | 0.0000000 | -4.4305648 | -2.1269288 | -0.8657483 | 0.0000000 | 0.0000000 | 0.0000000 | -1.2179134 | 0.0000000 | 0.0000000 | -5.6587432 | 0.0000000 | 0.0000000 | 0.0000000 | 0.0000000 | 0.0000000 | 0.0000000 | 0.0000000 | 0.0000000 | -0.3518948 | -1.5886513 | -0.4304366 | 0.0000000 | 36 | 6 | 64.86752 | 14.354079 |
| 0.0000000 | -0.9049998 | -2.2436510 | -0.3659037 | 0.0000000 | -0.7120758 | -0.4016273 | 0.0000000 | -0.2905995 | 0.0000000 | -0.1402657 | -0.2319032 | -0.2043276 | 0.0000000 | 0.0000000 | 0.0000000 | -0.3139241 | -0.2549284 | 0.0000000 | -0.0464567 | -0.0240665 | -0.0849348 | -0.2703637 | 0.0000000 | -0.0380346 | 37 | 1 | 176.14256 | 121.031055 |
| 0.0000000 | -0.9889025 | -2.1019511 | -0.3290610 | -0.2001634 | -1.1797440 | -0.0913098 | 0.0000000 | -0.2930524 | 0.0000000 | -0.2722146 | -0.3380337 | -0.3040830 | -0.1786371 | 0.0000000 | 0.0000000 | -0.2047984 | -0.4498606 | 0.0000000 | 0.0000000 | 0.0000000 | -0.0444517 | -0.3148520 | 0.0000000 | 0.0000000 | 37 | 2 | 127.82001 | 74.847307 |
| 0.0000000 | -1.4195369 | -2.2619951 | -1.1615956 | 0.0000000 | -1.1121879 | -0.5119074 | 0.0000000 | -0.4974159 | -0.2143213 | 0.0000000 | -0.1982576 | -2.0755357 | 0.0000000 | 0.0000000 | 0.0000000 | -0.3537841 | 0.0000000 | -0.2122505 | 0.0000000 | -0.0709750 | -0.5205769 | 0.0000000 | -0.2725787 | 0.0000000 | 37 | 3 | 104.72112 | 52.733053 |
| 0.0000000 | -1.9833069 | -0.5742479 | -3.5818258 | -0.7937438 | -0.7234813 | -0.1561329 | 0.0000000 | 0.0000000 | -0.1196345 | 0.0000000 | 0.0000000 | -1.9552942 | -0.0042851 | 0.0000000 | 0.0000000 | 0.0000000 | -0.0489655 | 0.0000000 | 0.0000000 | 0.0000000 | -0.5496748 | -1.1146688 | -0.1488774 | -0.2811813 | 37 | 4 | 83.46697 | 32.137410 |
| 0.0000000 | -2.0672900 | 0.0000000 | -3.3598580 | 0.0000000 | -1.8506149 | -0.0845865 | 0.0000000 | 0.0000000 | -1.2071925 | 0.0000000 | 0.0000000 | -4.9209043 | -0.4455709 | 0.0000000 | 0.0000000 | 0.0000000 | -0.4644595 | 0.0000000 | 0.0000000 | 0.0000000 | -0.0427185 | -0.8432226 | -0.2510645 | 0.0000000 | 37 | 5 | 74.22168 | 23.443182 |
| 0.6157423 | -2.8858082 | 0.0000000 | -3.2463421 | 0.0000000 | -2.3514165 | 0.0000000 | 0.0000000 | 0.0000000 | 0.0000000 | 0.0000000 | 0.0000000 | -8.9761314 | 0.0000000 | 0.0000000 | 0.0000000 | 0.0000000 | 0.0000000 | 0.0000000 | 0.0000000 | 0.0000000 | -0.9234871 | -1.5791399 | -0.0272027 | 0.0000000 | 37 | 6 | 65.07018 | 14.556192 |
| 0.0000000 | -0.6979881 | -1.9465018 | -0.0607684 | 0.0000000 | -0.8087244 | -0.5366837 | -0.1561782 | -0.4502632 | 0.0000000 | 0.0000000 | -0.0029040 | -0.4133366 | -0.4887758 | 0.0000000 | 0.0000000 | -0.2158539 | -0.1328008 | 0.0000000 | 0.0000000 | 0.0000000 | -0.1981143 | -0.3518116 | -0.0349706 | -0.0050237 | 38 | 1 | 172.12756 | 117.024366 |
| 0.0000000 | -0.8732557 | -2.2278580 | -0.5570061 | -0.2817244 | -0.9068840 | -0.3567325 | 0.0000000 | -0.2259856 | -0.1393099 | -0.0195712 | 0.0000000 | -0.1959239 | -0.2321556 | 0.0000000 | 0.0000000 | -0.2957815 | -0.1209057 | 0.0000000 | 0.0000000 | -0.0955791 | -0.2473589 | -0.3518838 | -0.2395180 | -0.0752081 | 38 | 2 | 131.03873 | 77.702642 |
| 0.0000000 | -0.4514692 | -3.0685175 | -0.2766274 | 0.0000000 | -1.6032048 | -0.5212820 | -0.0421100 | -0.4074840 | -0.6866580 | 0.0000000 | 0.0000000 | -0.8839383 | -0.4415467 | 0.0000000 | 0.0000000 | -0.4231600 | 0.0000000 | -0.1406535 | 0.0000000 | 0.0000000 | -0.2600765 | 0.0000000 | -0.3999129 | -0.3436946 | 38 | 3 | 105.32301 | 53.445463 |
| 0.0000000 | -1.5758864 | -1.5299328 | -1.3611614 | -0.4977143 | -2.2654808 | -0.2090089 | 0.0000000 | -0.1992816 | -0.1293676 | -0.0187188 | -0.2465803 | -1.4394337 | -0.3966618 | 0.0000000 | 0.0000000 | -0.3044372 | -0.2034095 | 0.0000000 | 0.0000000 | -0.2429208 | -0.4205737 | -0.1609494 | -0.3618611 | 0.0000000 | 38 | 4 | 84.07335 | 32.889133 |
| 0.4181638 | -2.6262247 | 0.0000000 | -3.5456707 | -0.3011653 | -2.0268974 | 0.0000000 | 0.0000000 | -0.1244064 | 0.0000000 | 0.0000000 | 0.0000000 | -3.7450062 | 0.0000000 | 0.0000000 | 0.0000000 | -0.1059467 | 0.0000000 | 0.0000000 | 0.0000000 | 0.0000000 | -0.4749517 | -1.7295456 | 0.0000000 | -0.0198523 | 38 | 5 | 76.32678 | 25.408493 |
| 0.0000000 | -2.9964503 | 0.0000000 | -4.2728035 | -4.4604524 | -2.6230053 | -0.2134848 | 0.0000000 | 0.0000000 | 0.0000000 | 0.0000000 | 0.0000000 | -7.7917654 | 0.0000000 | 0.0000000 | 0.0000000 | -0.4181045 | 0.0000000 | 0.0000000 | 0.0000000 | 0.0000000 | -0.5545845 | -0.9041655 | 0.0000000 | 0.0000000 | 38 | 6 | 61.27830 | 10.802582 |
| 0.0000000 | -0.9927066 | -1.9030348 | -0.2008628 | -0.0858116 | -0.8379179 | -0.3001139 | -0.1582549 | -0.1657125 | -0.0983535 | -0.2313227 | -0.3119074 | 0.0000000 | 0.0000000 | 0.0000000 | 0.0000000 | -0.2687187 | -0.2950881 | 0.0000000 | 0.0000000 | -0.0271800 | -0.0187633 | -0.2351887 | -0.1681610 | 0.0000000 | 39 | 1 | 172.34539 | 117.090073 |
| 0.0000000 | -0.9896589 | -1.7847262 | -0.6260038 | 0.0000000 | -0.8751954 | -0.2023801 | 0.0000000 | -0.4680237 | 0.0000000 | -0.0937986 | -0.7575044 | -0.2227020 | 0.0000000 | 0.0000000 | 0.0000000 | -0.2529205 | -0.1009781 | -0.2417275 | 0.0000000 | 0.0000000 | -0.1269237 | -0.4431523 | 0.0000000 | 0.0000000 | 39 | 2 | 129.68067 | 76.502409 |
| 0.0000000 | -1.4070582 | -2.7573915 | 0.0000000 | 0.0000000 | -1.5968507 | -0.2663132 | 0.0000000 | -0.0426981 | 0.0000000 | 0.0000000 | -0.2570898 | -0.8953093 | -0.9477284 | 0.0000000 | 0.0000000 | -0.2663861 | 0.0000000 | 0.0000000 | 0.0000000 | 0.0000000 | -0.4303955 | -0.5252975 | 0.0000000 | 0.0000000 | 39 | 3 | 105.41056 | 53.628683 |
| 0.0000000 | -1.9823336 | -0.8769623 | -2.2095265 | -0.1273242 | -1.7579441 | 0.0000000 | 0.0000000 | 0.0000000 | -0.7918436 | 0.0000000 | 0.0000000 | -3.0404299 | -0.0515474 | 0.0000000 | 0.0000000 | -0.0398224 | 0.0000000 | 0.0000000 | 0.0000000 | 0.0000000 | -0.2114473 | -0.9799635 | -0.2759134 | -0.3938537 | 39 | 4 | 83.35604 | 32.038525 |
| 0.0000000 | -2.1200692 | -0.1719264 | -3.2011392 | -2.5161616 | -1.6273272 | 0.0000000 | 0.0000000 | 0.0000000 | 0.0000000 | 0.0000000 | 0.0000000 | -2.6499290 | 0.0000000 | 0.0000000 | 0.0000000 | -0.6179304 | 0.0000000 | 0.0000000 | 0.0000000 | 0.0000000 | -0.5119732 | -1.1485075 | 0.0000000 | 0.0000000 | 39 | 5 | 73.48518 | 22.725519 |
| 0.0054032 | -3.7970639 | 0.0000000 | -3.6320070 | -5.0223580 | -1.7394229 | -0.1873736 | 0.0000000 | 0.0000000 | -0.5711735 | 0.0000000 | 0.0000000 | -7.2193789 | 0.0000000 | 0.0000000 | 0.0000000 | -0.3696013 | -0.3269436 | 0.0000000 | 0.0000000 | 0.0000000 | -0.5301568 | -0.3594674 | -0.2079571 | -0.3257133 | 39 | 6 | 62.64579 | 12.120631 |
| 0.0000000 | -0.9149961 | -2.0197080 | -0.0923557 | 0.0000000 | -0.8115600 | -0.2206454 | 0.0000000 | -0.4813796 | -0.0058851 | -0.0366781 | -0.3279197 | -0.3363997 | 0.0000000 | 0.0000000 | 0.0000000 | -0.1266920 | -0.4151656 | -0.0013479 | 0.0000000 | -0.1261507 | -0.1671052 | -0.1535785 | 0.0000000 | -0.3585263 | 40 | 1 | 183.28067 | 128.155774 |
| 0.0000000 | -0.6683470 | -2.2151743 | -0.5607810 | -0.1741701 | -0.9312453 | -0.3925818 | 0.0000000 | -0.1232620 | -0.5780752 | -0.0240152 | -0.3632884 | -0.6210672 | -0.1574566 | 0.0000000 | 0.0000000 | -0.3770210 | -0.0252109 | 0.0000000 | 0.0000000 | 0.0000000 | -0.2740136 | -0.3439892 | -0.1538062 | 0.0000000 | 40 | 2 | 127.84263 | 74.774759 |
| 0.1341957 | -1.1896921 | -2.2189215 | -0.3180445 | 0.0000000 | -1.6617344 | -0.0814823 | -0.5914946 | 0.0000000 | -0.6065704 | 0.0000000 | 0.0000000 | -0.0649188 | -2.0483837 | 0.0000000 | 0.0000000 | -0.4380364 | -0.0927721 | 0.0000000 | 0.0000000 | 0.0000000 | -0.4171348 | -0.5491710 | -0.0102352 | -0.3152929 | 40 | 3 | 94.54791 | 42.674050 |
| 0.3305448 | -1.6565810 | -2.5348515 | -0.6193276 | 0.0000000 | -2.4507743 | 0.0000000 | 0.0000000 | -0.3076814 | 0.0000000 | 0.0000000 | -1.2543020 | 0.0000000 | -0.1411955 | 0.0000000 | 0.0000000 | -0.2405257 | 0.0000000 | 0.0000000 | 0.0000000 | 0.0000000 | -0.5311625 | -0.5430241 | 0.0000000 | -0.3920986 | 40 | 4 | 90.26614 | 39.064035 |
| 0.0000000 | -2.1394225 | -1.4665589 | -1.8601230 | 0.0000000 | -2.2486521 | -1.0082095 | 0.0000000 | 0.0000000 | -0.6843544 | -0.3931251 | 0.0000000 | -3.0658098 | -0.9619456 | 0.0000000 | 0.0000000 | -0.3582442 | 0.0000000 | 0.0000000 | 0.0000000 | 0.0000000 | -0.2099119 | -0.1491965 | 0.0000000 | 0.0000000 | 40 | 5 | 76.57507 | 25.719456 |
| 0.0000000 | -2.8657122 | 0.0000000 | -3.7929978 | -1.3419026 | -1.4363932 | 0.0000000 | 0.0000000 | 0.0000000 | -0.5708347 | 0.0000000 | 0.0000000 | -8.3136017 | 0.0000000 | 0.0000000 | 0.0000000 | -0.4524751 | 0.0000000 | -0.2746001 | 0.0000000 | 0.0000000 | -0.5049649 | -0.2820680 | -0.6431386 | -0.0467491 | 40 | 6 | 66.00551 | 15.428597 |
| 0.0000000 | -0.8653820 | -1.6716220 | -0.1424970 | -0.1809436 | -0.8898679 | -0.0185081 | 0.0000000 | -0.4348790 | 0.0000000 | -0.2485160 | -0.0668776 | -0.2782112 | -0.2179101 | 0.0000000 | 0.0000000 | -0.1269185 | -0.1571397 | 0.0000000 | 0.0000000 | -0.2529381 | 0.0000000 | -0.3437445 | 0.0000000 | -0.3439790 | 41 | 1 | 176.80894 | 121.595558 |
| 0.0000000 | -0.7596524 | -2.2486328 | -0.3165722 | 0.0000000 | -1.1290375 | -0.1297300 | -0.0205842 | -0.3334215 | 0.0000000 | -0.2233997 | -0.1009326 | -0.4414407 | -0.0701426 | 0.0000000 | 0.0000000 | -0.2692799 | -0.3153437 | 0.0000000 | 0.0000000 | -0.3500855 | -0.0150747 | -0.0598705 | -0.1781285 | -0.2749996 | 41 | 2 | 133.23717 | 79.994582 |
| 0.0000000 | -0.9646226 | -2.8396926 | -0.3529901 | 0.0000000 | -1.2938097 | -0.4987916 | 0.0000000 | 0.0000000 | -0.6180732 | 0.0000000 | -0.2337840 | -0.8681998 | -1.0655064 | 0.0000000 | 0.0000000 | -0.3760596 | 0.0000000 | 0.0000000 | 0.0000000 | 0.0000000 | -0.2724264 | -0.1466259 | -0.6173147 | 0.0000000 | 41 | 3 | 99.41752 | 47.633584 |
| 0.2514642 | -1.4663434 | -1.1823134 | -1.5362230 | -0.5670987 | -2.0006815 | 0.0000000 | 0.0000000 | -1.0457214 | 0.0000000 | 0.0000000 | -1.1758136 | -0.9243092 | 0.0000000 | 0.0000000 | 0.0000000 | -0.1685950 | -0.3290537 | 0.0000000 | 0.0000000 | 0.0000000 | -0.4266629 | -0.4447822 | 0.0000000 | 0.0000000 | 41 | 4 | 82.66881 | 31.524907 |
| 0.0000000 | -3.3026808 | 0.0000000 | -4.9288799 | -2.1620066 | -0.4455092 | 0.0000000 | 0.0000000 | 0.0000000 | -0.5573641 | 0.0000000 | 0.0000000 | -4.8703709 | 0.0000000 | 0.0000000 | 0.0000000 | -0.4453982 | 0.0000000 | 0.0000000 | 0.0000000 | 0.0000000 | -0.7370669 | -0.9423703 | -0.1238970 | -0.3336779 | 41 | 5 | 69.41808 | 18.737683 |
| 0.0000000 | -2.6405433 | 0.0000000 | -2.4739507 | 0.0000000 | -1.8595578 | 0.0000000 | 0.0000000 | 0.0000000 | -1.0878919 | 0.0000000 | 0.0000000 | -5.7967498 | -1.0164855 | 0.0000000 | 0.0000000 | -0.2679361 | 0.0000000 | 0.0000000 | 0.0000000 | 0.0000000 | -0.3481361 | -0.7538724 | -0.4192693 | -0.1922627 | 41 | 6 | 71.61840 | 20.903897 |
| 0.0000000 | -1.1022101 | -1.8239242 | -0.2371339 | 0.0000000 | -0.8762291 | -0.3014701 | -0.1046620 | -0.3279960 | -0.1286706 | 0.0000000 | -0.2992577 | -0.0221255 | -0.2306133 | 0.0000000 | 0.0000000 | -0.1407503 | -0.1261780 | 0.0000000 | 0.0000000 | -0.0291715 | -0.2778317 | -0.3163058 | -0.1073192 | -0.1672153 | 42 | 1 | 179.71003 | 124.497960 |
| 0.0000000 | -0.6223875 | -2.2665876 | -0.2184910 | -0.0439290 | -1.2280073 | -0.2266647 | -0.1294398 | -0.0788325 | -0.3809460 | 0.0000000 | -0.4951300 | -0.1399250 | 0.0000000 | 0.0000000 | 0.0000000 | -0.1654287 | -0.0143364 | 0.0000000 | 0.0000000 | 0.0000000 | -0.2548056 | -0.5890034 | 0.0000000 | 0.0000000 | 42 | 2 | 128.82605 | 75.696289 |
| 0.0726680 | -1.0193405 | -2.7419289 | -0.4689879 | 0.0000000 | -1.8969132 | -0.2361651 | 0.0000000 | -0.3316332 | 0.0000000 | 0.0000000 | -1.2308831 | -0.2061802 | 0.0000000 | 0.0000000 | 0.0000000 | -0.2497014 | -0.0115063 | -0.4149974 | 0.0000000 | 0.0000000 | -0.3369361 | -0.1179766 | -0.1650909 | -0.0879641 | 42 | 3 | 96.83884 | 45.019395 |
| 0.5496417 | -1.3697223 | -2.4074417 | -2.2577721 | 0.0000000 | -1.4502895 | -1.1026537 | 0.0000000 | 0.0000000 | -0.7294468 | 0.0000000 | -0.4117754 | -1.2762911 | -0.5670527 | 0.0000000 | 0.0000000 | -0.4063661 | 0.0000000 | 0.0000000 | 0.0000000 | 0.0000000 | -0.3767123 | -0.3328464 | -0.4633035 | 0.0000000 | 42 | 4 | 83.90241 | 32.727115 |
| 0.0000000 | -2.7392406 | 0.0000000 | -3.8646572 | -1.8725296 | -1.3964965 | 0.0000000 | 0.0000000 | 0.0000000 | -0.9403794 | 0.0000000 | 0.0000000 | -7.8305318 | 0.0000000 | 0.0000000 | 0.0000000 | 0.0000000 | 0.0000000 | 0.0000000 | 0.0000000 | 0.0000000 | -0.3747888 | -1.2790569 | 0.0000000 | 0.0000000 | 42 | 5 | 67.04405 | 16.327268 |
| 0.0000000 | -3.7482097 | 0.0000000 | -4.2683878 | 0.0000000 | -3.5817148 | 0.0000000 | 0.0000000 | 0.0000000 | 0.0000000 | 0.0000000 | 0.0000000 | -6.0850207 | 0.0000000 | 0.0000000 | -0.4254779 | -0.3042533 | -0.4902964 | 0.0000000 | 0.0000000 | 0.0000000 | -0.1677067 | -0.2831351 | 0.0000000 | -0.6490757 | 42 | 6 | 63.71885 | 13.153228 |
| 0.0000000 | -0.9086389 | -1.9498038 | -0.0306137 | 0.0000000 | -1.1082682 | -0.2060803 | -0.0308519 | -0.1708617 | -0.1449096 | -0.1227625 | -0.1694177 | -0.5353727 | 0.0000000 | 0.0000000 | 0.0000000 | -0.2941104 | -0.0636864 | 0.0000000 | 0.0000000 | -0.1375294 | -0.1937357 | -0.3148097 | 0.0000000 | 0.0000000 | 43 | 1 | 175.90903 | 120.870436 |
| 0.0000000 | -0.8103101 | -2.1319396 | -0.2769120 | -0.1373431 | -1.2349467 | -0.1466717 | 0.0000000 | -0.3711362 | 0.0000000 | 0.0000000 | -0.4323087 | -0.3450783 | 0.0000000 | 0.0000000 | 0.0000000 | -0.1081192 | -0.0449706 | 0.0000000 | 0.0000000 | 0.0000000 | -0.3445275 | -0.5484079 | 0.0000000 | 0.0000000 | 43 | 2 | 123.60813 | 70.610954 |
| 0.0000000 | -1.1592788 | -1.4233093 | -1.1641074 | 0.0000000 | -1.3488522 | -0.8956101 | -0.0431701 | -0.0834861 | -0.3919537 | 0.0000000 | 0.0000000 | -2.3058009 | -0.5006647 | 0.0000000 | 0.0000000 | -0.2588963 | 0.0000000 | -0.0446776 | 0.0000000 | 0.0000000 | -0.4852476 | -0.5144964 | -0.1913556 | -0.4051813 | 43 | 3 | 99.58235 | 47.640012 |
| 0.0000000 | -2.6274310 | 0.0000000 | -3.6337005 | 0.0000000 | -1.6345671 | -0.7480686 | 0.0000000 | 0.0000000 | -0.1742437 | 0.0000000 | -0.6831601 | -1.6915355 | 0.0000000 | 0.0000000 | 0.0000000 | -0.2336908 | 0.0000000 | 0.0000000 | 0.0000000 | 0.0000000 | -0.5965380 | -0.7646954 | -0.2222099 | 0.0000000 | 43 | 4 | 84.05480 | 32.780248 |
| 0.0000000 | -2.0151130 | 0.0000000 | -3.2640977 | -1.1806566 | -1.7512878 | -0.0781946 | 0.0000000 | -0.4113508 | -0.2310803 | 0.0000000 | 0.0000000 | -4.5702049 | -0.1835915 | 0.0000000 | 0.0000000 | -0.3216472 | 0.0000000 | 0.0000000 | 0.0000000 | 0.0000000 | -0.4330240 | -0.8944502 | -0.1881220 | -0.1396848 | 43 | 5 | 75.30064 | 24.296215 |
| 0.0493457 | -2.3108110 | 0.0000000 | -2.5585053 | -0.6729751 | -1.8703813 | -0.2619581 | 0.0000000 | 0.0000000 | -0.1624028 | 0.0000000 | 0.0000000 | -4.1853136 | -1.2756741 | 0.0000000 | 0.0000000 | -0.2279112 | 0.0000000 | 0.0000000 | 0.0000000 | 0.0000000 | -0.9905915 | -0.5152139 | -0.5665496 | -0.5290940 | 43 | 6 | 70.65641 | 20.020158 |
| 0.0000000 | -1.0807984 | -1.8354885 | -0.1846366 | 0.0000000 | -0.8692416 | -0.5029193 | -0.0000129 | -0.1761358 | -0.0550029 | 0.0000000 | -0.2665907 | -0.2110036 | -0.1988147 | 0.0000000 | 0.0000000 | -0.3114996 | -0.1271969 | 0.0000000 | 0.0000000 | -0.1925112 | -0.3598623 | -0.2611841 | 0.0000000 | -0.3503945 | 44 | 1 | 170.32287 | 115.242232 |
| 0.0000000 | -0.8370673 | -1.7654909 | -0.4370638 | 0.0000000 | -1.3408647 | 0.0000000 | 0.0000000 | -0.2366802 | -0.1598085 | 0.0000000 | -0.7144812 | 0.0000000 | 0.0000000 | 0.0000000 | 0.0000000 | -0.2276902 | 0.0000000 | 0.0000000 | 0.0000000 | -0.0382929 | -0.4784601 | -0.5041896 | -0.0279544 | -0.2279338 | 44 | 2 | 129.83869 | 76.628130 |
| 0.0000000 | -0.5111537 | -2.1536972 | -0.7533664 | 0.0000000 | -1.6868253 | -0.2484945 | 0.0000000 | -0.2582705 | -0.1608022 | 0.0000000 | -1.0977146 | -0.2763170 | 0.0000000 | 0.0000000 | 0.0000000 | -0.2449141 | 0.0000000 | 0.0000000 | 0.0000000 | 0.0000000 | -0.4921099 | -0.4675955 | -0.1064316 | -0.0795857 | 44 | 3 | 105.12612 | 53.212710 |
| 0.0000000 | -1.9940874 | -0.6012485 | -2.2471461 | -1.0733269 | -1.3191052 | -0.1021874 | 0.0000000 | 0.0000000 | -0.1224224 | 0.0000000 | 0.0000000 | -3.9191060 | 0.0000000 | 0.0000000 | 0.0000000 | -0.4295615 | 0.0000000 | 0.0000000 | 0.0000000 | 0.0000000 | -0.6479992 | -0.8304634 | -0.2740936 | -0.3000934 | 44 | 4 | 81.96453 | 30.717963 |
| 0.0000000 | -1.6342993 | -1.0040691 | -3.5869745 | -0.2226176 | -1.7479890 | -0.6178672 | 0.0000000 | 0.0000000 | -0.4429003 | 0.0000000 | 0.0000000 | -3.3410870 | -0.6743420 | 0.0000000 | 0.0000000 | -0.5619541 | 0.0000000 | 0.0000000 | 0.0000000 | 0.0000000 | -0.4687148 | -0.1967413 | -0.5538702 | 0.0000000 | 44 | 5 | 73.45355 | 22.589343 |
| 0.0000000 | -2.6034642 | 0.0000000 | -4.3456661 | -2.7784322 | -0.1352354 | -0.0780738 | 0.0000000 | -0.5042040 | -0.1946428 | 0.0000000 | 0.0000000 | -8.2609556 | 0.0000000 | 0.0000000 | 0.0000000 | -0.0372080 | 0.0000000 | 0.0000000 | 0.0000000 | 0.0000000 | -0.2944928 | -0.6386524 | -1.0253417 | 0.0000000 | 44 | 6 | 64.14624 | 13.679323 |
| 0.0000000 | -0.9038824 | -1.9490925 | -0.2632495 | 0.0000000 | -0.7285978 | -0.3338250 | -0.0769878 | -0.2193655 | -0.4361967 | -0.0623721 | -0.4153454 | -0.2244367 | 0.0000000 | 0.0000000 | 0.0000000 | -0.1954890 | -0.1160280 | 0.0000000 | 0.0000000 | -0.0236651 | -0.1084193 | -0.3894652 | -0.0171896 | 0.0000000 | 45 | 1 | 169.93999 | 114.806999 |
| 0.0000000 | -0.8282771 | -2.3898016 | 0.0000000 | 0.0000000 | -1.2955030 | -0.4356336 | 0.0000000 | -0.3195106 | 0.0000000 | 0.0000000 | -0.1304744 | -0.5634401 | -0.2590736 | 0.0000000 | 0.0000000 | -0.2305204 | -0.0220849 | 0.0000000 | 0.0000000 | 0.0000000 | -0.2985401 | -0.4425730 | -0.1621753 | 0.0000000 | 45 | 2 | 127.29442 | 74.385068 |
| 0.0000000 | -0.9603478 | -2.2732640 | 0.0000000 | 0.0000000 | -1.3906576 | -0.1994190 | -0.3590973 | -0.4231986 | 0.0000000 | 0.0000000 | -0.5405790 | -0.7763398 | -0.7793393 | 0.0000000 | 0.0000000 | -0.2646783 | 0.0000000 | 0.0000000 | 0.0000000 | 0.0000000 | -0.6163591 | -0.3884713 | -0.0458318 | -0.1645292 | 45 | 3 | 102.47987 | 50.532237 |
| 0.0000000 | -1.7746280 | -0.8731228 | -1.4783353 | 0.0000000 | -2.2236901 | -0.3778670 | 0.0000000 | 0.0000000 | -0.9184649 | 0.0000000 | 0.0000000 | -5.0824595 | 0.0000000 | 0.0000000 | 0.0000000 | -0.4403933 | -0.0532974 | -0.0570330 | 0.0000000 | 0.0000000 | -0.6988515 | -0.3205562 | -0.1331202 | 0.0000000 | 45 | 4 | 80.82192 | 29.445326 |
| 0.0000000 | -1.8013717 | -0.6956874 | -4.3040815 | -0.2175521 | -0.6963758 | -1.1173187 | 0.0000000 | 0.0000000 | -0.7155163 | 0.0000000 | -0.4156679 | -3.0563752 | 0.0000000 | 0.0000000 | 0.0000000 | -0.6304196 | 0.0000000 | 0.0000000 | 0.0000000 | 0.0000000 | -0.7665900 | -0.0956664 | -0.4310811 | -0.0749016 | 45 | 5 | 78.15433 | 27.286277 |
| 3.4737617 | -3.6254717 | 0.0000000 | -3.7394133 | -0.5289627 | -2.5072077 | 0.0000000 | 0.0000000 | 0.0000000 | -0.9259841 | 0.0000000 | 0.0000000 | -8.9499352 | 0.0000000 | 0.0000000 | 0.0000000 | -0.1257101 | 0.0000000 | 0.0000000 | 0.0000000 | 0.0000000 | -0.6084457 | -4.3313512 | 0.0000000 | 0.0000000 | 45 | 6 | 62.81343 | 12.342015 |
| 0.0000000 | -0.9330417 | -1.9800501 | 0.0000000 | -0.0570844 | -0.9600780 | -0.0646451 | 0.0000000 | -0.2894456 | 0.0000000 | -0.0933338 | -0.2765332 | -0.1659662 | -0.0740625 | 0.0000000 | 0.0000000 | -0.0698266 | -0.1866903 | 0.0000000 | -0.0856872 | 0.0000000 | -0.1486037 | -0.3753782 | 0.0000000 | -0.4449574 | 46 | 1 | 170.24210 | 115.071016 |
| 0.0000000 | -0.5486339 | -2.1899593 | -0.3975462 | -0.1496269 | -0.9116527 | -0.7298584 | 0.0000000 | -0.3512336 | -0.1396274 | 0.0000000 | -0.1737217 | 0.0000000 | -0.5614511 | -0.0026874 | 0.0000000 | -0.2316960 | -0.0801665 | 0.0000000 | 0.0000000 | 0.0000000 | -0.2574879 | -0.2656891 | -0.1968754 | -0.3918777 | 46 | 2 | 129.29704 | 76.193772 |
| 0.0000000 | -1.2789404 | -2.3668591 | -0.9477867 | 0.0000000 | -2.0901855 | 0.0000000 | 0.0000000 | -0.4502913 | -0.2636429 | 0.0000000 | -0.0954522 | -0.6590535 | -0.4429335 | 0.0000000 | 0.0000000 | -0.1609828 | 0.0000000 | 0.0000000 | -0.1278158 | 0.0000000 | -0.3633895 | -0.4571072 | 0.0000000 | 0.0000000 | 46 | 3 | 97.96922 | 46.176380 |
| 0.0000000 | -2.7207843 | -0.3275648 | -2.8208158 | -0.1693096 | -1.9760204 | -0.0041997 | 0.0000000 | -0.3212814 | 0.0000000 | 0.0000000 | 0.0000000 | -3.8516815 | 0.0000000 | 0.0000000 | 0.0000000 | -0.3226004 | -0.0298391 | 0.0000000 | 0.0000000 | 0.0000000 | -0.6759136 | -0.5779012 | -0.3713429 | 0.0000000 | 46 | 4 | 80.39422 | 29.157569 |
| 0.0000000 | -1.4362795 | -2.0596152 | -2.3772892 | 0.0000000 | -2.9711236 | -0.9391815 | -0.0930282 | 0.0000000 | 0.0000000 | 0.0000000 | -0.9284732 | -0.2184659 | -0.1332523 | 0.0000000 | 0.0000000 | -0.1776178 | 0.0000000 | 0.0000000 | 0.0000000 | 0.0000000 | 0.0000000 | -0.3977746 | 0.0000000 | -0.6737875 | 46 | 5 | 75.62805 | 24.838765 |
| 0.0000000 | -2.0461992 | -1.2286275 | -2.6480473 | 0.0000000 | -1.7774085 | 0.0000000 | -0.7800058 | 0.0000000 | 0.0000000 | -0.8111117 | 0.0000000 | -3.7566241 | -1.2014984 | 0.0000000 | 0.0000000 | -0.3532525 | 0.0000000 | 0.0000000 | 0.0000000 | 0.0000000 | 0.0000000 | -0.1908707 | -0.5907915 | 0.0000000 | 46 | 6 | 67.67983 | 17.051253 |
| 0.0000000 | -1.0156823 | -1.7104446 | -0.0506325 | 0.0000000 | -0.9929467 | 0.0000000 | -0.2225242 | -0.4529351 | 0.0000000 | -0.1143813 | -0.2482883 | -0.2872807 | 0.0000000 | 0.0000000 | 0.0000000 | -0.2181588 | -0.2032607 | 0.0000000 | -0.0241253 | -0.2762258 | 0.0000000 | -0.2889177 | 0.0000000 | -0.2518124 | 47 | 1 | 172.81430 | 117.644916 |
| 0.0000000 | -0.9710877 | -2.3694109 | 0.0000000 | 0.0000000 | -1.2849408 | -0.0127641 | -0.1091799 | -0.1249578 | 0.0000000 | -0.1378013 | -0.4501782 | -0.6129497 | 0.0000000 | 0.0000000 | 0.0000000 | -0.2254874 | -0.2869842 | -0.0524288 | 0.0000000 | -0.1929485 | -0.2911138 | -0.0612330 | -0.1833603 | 0.0000000 | 47 | 2 | 122.66484 | 69.690917 |
| 0.0000000 | -1.0442804 | -2.0090286 | -1.0739817 | 0.0000000 | -1.4310631 | -0.2435793 | 0.0000000 | 0.0000000 | -0.2640587 | 0.0000000 | -0.1837258 | -1.5359779 | -0.6751154 | 0.0000000 | 0.0000000 | -0.4734531 | 0.0000000 | 0.0000000 | 0.0000000 | 0.0000000 | -0.4486838 | -0.6299824 | -0.0300113 | 0.0000000 | 47 | 3 | 96.77754 | 44.968833 |
| 0.0000000 | -2.0811824 | -0.9126735 | -2.6021290 | -0.7963002 | -0.9141072 | 0.0000000 | 0.0000000 | 0.0000000 | -0.7209178 | -0.0650624 | 0.0000000 | -4.0069897 | 0.0000000 | 0.0000000 | 0.0000000 | -0.3950903 | 0.0000000 | 0.0000000 | 0.0000000 | 0.0000000 | -0.3470825 | -1.0738003 | 0.0000000 | -0.4100657 | 47 | 4 | 79.40676 | 28.235958 |
| 0.0000000 | -2.4495202 | 0.0000000 | -3.1910115 | -2.9914983 | 0.0000000 | -0.8301957 | 0.0000000 | 0.0000000 | -0.2524448 | 0.0000000 | 0.0000000 | -7.9168864 | 0.0000000 | 0.0000000 | 0.0000000 | -0.0383917 | 0.0000000 | 0.0000000 | 0.0000000 | 0.0000000 | -0.7007571 | -0.9624276 | -0.2060441 | -1.2655104 | 47 | 5 | 69.30777 | 18.543859 |
| 0.0000000 | -3.2130326 | 0.0000000 | -4.0812003 | -0.0028201 | -1.4601736 | 0.0000000 | 0.0000000 | 0.0000000 | -0.3346305 | -0.2596484 | 0.0000000 | -5.7349182 | 0.0000000 | 0.0000000 | 0.0000000 | -0.5736059 | 0.0000000 | 0.0000000 | 0.0000000 | 0.0000000 | -0.4863228 | -0.9569073 | 0.0000000 | 0.0000000 | 47 | 6 | 68.56352 | 17.970649 |
| 0.0000000 | -0.9958809 | -1.9030805 | -0.5613094 | -0.2424291 | -0.4456528 | -0.4435273 | -0.0752875 | -0.4580410 | 0.0000000 | -0.0878796 | -0.4599379 | -0.0054973 | -0.0436605 | 0.0000000 | 0.0000000 | -0.1622405 | -0.2097497 | 0.0000000 | 0.0000000 | 0.0000000 | -0.1971718 | -0.2398807 | -0.1057752 | 0.0000000 | 48 | 1 | 177.91341 | 122.826758 |
| 0.0000000 | -0.7793207 | -2.2781233 | 0.0000000 | -0.0627451 | -1.0916474 | 0.0000000 | 0.0000000 | -0.6525929 | 0.0000000 | 0.0000000 | -0.2795535 | -0.4921803 | -0.4140772 | 0.0000000 | 0.0000000 | -0.3999052 | -0.1853886 | -0.0357551 | 0.0000000 | 0.0000000 | -0.2704831 | -0.2936037 | 0.0000000 | 0.0000000 | 48 | 2 | 130.71931 | 77.765260 |
| 0.0000000 | -1.9018374 | -1.9676630 | -0.8362055 | 0.0000000 | -1.5386295 | 0.0000000 | 0.0000000 | -0.3826090 | -0.0360729 | 0.0000000 | -0.6839958 | -1.6989851 | -0.1059007 | 0.0000000 | 0.0000000 | -0.0439021 | -0.1176455 | 0.0000000 | 0.0000000 | 0.0000000 | -0.6137639 | -0.3549272 | -0.1671983 | 0.0000000 | 48 | 3 | 95.54040 | 43.706998 |
| 0.0000000 | -2.4641421 | 0.0000000 | -3.2518181 | -0.3630704 | -1.7728117 | 0.0000000 | 0.0000000 | 0.0000000 | -0.6034960 | 0.0000000 | -0.2516514 | -3.1699409 | -0.1872223 | 0.0000000 | 0.0000000 | 0.0000000 | -0.4077733 | 0.0000000 | 0.0000000 | -0.1746711 | -0.6631738 | -0.7359324 | -0.0521656 | 0.0000000 | 48 | 4 | 77.35487 | 26.134483 |
| 0.0000000 | -2.4260321 | 0.0000000 | -2.8802820 | -0.9418242 | -1.6961194 | 0.0000000 | 0.0000000 | 0.0000000 | -0.9527499 | 0.0000000 | 0.0000000 | -4.9608824 | -0.1520230 | 0.0000000 | 0.0000000 | -0.6788742 | -0.4605608 | 0.0000000 | 0.0000000 | 0.0000000 | -0.8300466 | -0.3997541 | 0.0000000 | -0.6945944 | 48 | 5 | 73.41334 | 22.589586 |
| 0.0000000 | -2.6459134 | 0.0000000 | -3.1713018 | -1.9931774 | -1.9769333 | 0.0000000 | 0.0000000 | 0.0000000 | -1.1258799 | 0.0000000 | 0.0000000 | -7.5086208 | 0.0000000 | 0.0000000 | 0.0000000 | 0.0000000 | 0.0000000 | 0.0000000 | 0.0000000 | 0.0000000 | 0.0000000 | -1.4658813 | 0.0000000 | 0.0000000 | 48 | 6 | 62.34238 | 11.891629 |
| 0.0000000 | -1.1547345 | -1.7289067 | -0.3956424 | -0.0478646 | -0.7660075 | -0.4827594 | -0.2350501 | -0.5135627 | 0.0000000 | -0.0271409 | -0.1791704 | 0.0000000 | 0.0000000 | 0.0000000 | 0.0000000 | -0.2141747 | -0.1105620 | 0.0000000 | 0.0000000 | -0.1199158 | -0.3011429 | -0.2993877 | -0.0599755 | -0.0501706 | 49 | 1 | 169.82391 | 114.596474 |
| 0.0000000 | -0.6067481 | -2.1588707 | 0.0000000 | -0.1188907 | -0.9550654 | -0.1290076 | 0.0000000 | -0.2961976 | 0.0000000 | 0.0000000 | -0.2934614 | -0.1724141 | -0.7913549 | 0.0000000 | 0.0000000 | -0.2220728 | -0.3011419 | 0.0000000 | 0.0000000 | 0.0000000 | -0.4502333 | -0.3735534 | 0.0000000 | 0.0000000 | 49 | 2 | 130.61651 | 77.551628 |
| 0.0000000 | -1.0517804 | -2.1276407 | -0.5650597 | 0.0000000 | -2.2484970 | -0.4151095 | 0.0000000 | -0.0255460 | -0.3336040 | 0.0000000 | 0.0000000 | -1.4399323 | -0.5027335 | 0.0000000 | 0.0000000 | -0.4777076 | 0.0000000 | 0.0000000 | 0.0000000 | 0.0000000 | -0.5062087 | -0.5396951 | 0.0000000 | 0.0000000 | 49 | 3 | 96.91231 | 45.030637 |
| 0.0539987 | -0.9107587 | -2.4101288 | -0.3279357 | -0.8489951 | -1.6219864 | -0.2174852 | 0.0000000 | 0.0000000 | -0.1789776 | 0.0000000 | 0.0000000 | -2.3986641 | -0.3916276 | 0.0000000 | 0.0000000 | -0.4042398 | 0.0000000 | 0.0000000 | 0.0000000 | 0.0000000 | -0.5655568 | -0.7634581 | 0.0000000 | 0.0000000 | 49 | 4 | 86.04140 | 34.859176 |
| 0.0000000 | -2.2258468 | 0.0000000 | -3.6135439 | -5.4431425 | -0.7415887 | 0.0000000 | 0.0000000 | 0.0000000 | -0.6585393 | 0.0000000 | 0.0000000 | -6.3500209 | 0.0000000 | 0.0000000 | 0.0000000 | 0.0000000 | 0.0000000 | 0.0000000 | 0.0000000 | 0.0000000 | -0.8310926 | -1.1710591 | 0.0000000 | -0.4110370 | 49 | 5 | 66.59777 | 15.755749 |
| 0.0784173 | -2.8655493 | 0.0000000 | -4.1712716 | -4.1813601 | -1.5806055 | 0.0000000 | 0.0000000 | 0.0000000 | 0.0000000 | 0.0000000 | 0.0000000 | -7.5375287 | 0.0000000 | 0.0000000 | 0.0000000 | 0.0000000 | -0.4362876 | 0.0000000 | 0.0000000 | 0.0000000 | -0.8676857 | -0.8766107 | 0.0000000 | 0.0000000 | 49 | 6 | 64.05930 | 13.509803 |
| 0.0000000 | -0.8737384 | -2.1042491 | -0.0779645 | -0.3835071 | -0.3990472 | -0.3629989 | -0.0793588 | -0.3170770 | -0.0541930 | -0.1456347 | -0.4193686 | -0.1101370 | -0.0390833 | 0.0000000 | 0.0000000 | -0.1298283 | -0.2598408 | -0.0230281 | 0.0000000 | -0.1488940 | -0.0097718 | -0.2560880 | -0.0034705 | -0.2972501 | 50 | 1 | 180.04908 | 124.920130 |
| 0.0000000 | -0.8914681 | -2.0443624 | -1.0617791 | 0.0000000 | -0.9103418 | -0.3701861 | 0.0000000 | -0.3543835 | 0.0000000 | 0.0000000 | 0.0000000 | -0.4886561 | -0.1719225 | 0.0000000 | 0.0000000 | -0.1884540 | -0.1211665 | 0.0000000 | -0.1304521 | 0.0000000 | -0.3764372 | -0.5560020 | 0.0000000 | 0.0000000 | 50 | 2 | 120.65611 | 67.568442 |
| 0.0000000 | -1.4034897 | -1.7403116 | -1.1133202 | 0.0000000 | -1.0613313 | 0.0000000 | -0.1430013 | 0.0000000 | -0.3991799 | 0.0000000 | 0.0000000 | -1.4759260 | -1.0564642 | 0.0000000 | 0.0000000 | -0.3116474 | -0.0001184 | -0.2201633 | 0.0000000 | 0.0000000 | -0.5363776 | -0.7684855 | 0.0000000 | 0.0000000 | 50 | 3 | 97.27120 | 45.354101 |
| 0.0000000 | -1.4092814 | -1.6306217 | -1.9986600 | 0.0000000 | -1.2856242 | -0.2915327 | 0.0000000 | 0.0000000 | -0.6145478 | 0.0000000 | 0.0000000 | -2.5409390 | -1.1152821 | 0.0000000 | 0.0000000 | -0.0222763 | 0.0000000 | -0.1272667 | 0.0000000 | 0.0000000 | -0.6677150 | -0.4324496 | -0.2642005 | 0.0000000 | 50 | 4 | 82.20769 | 31.034081 |
| 0.0000000 | -2.6104332 | 0.0000000 | -2.8709413 | -1.0725838 | -2.1824187 | -0.5463196 | 0.0000000 | 0.0000000 | 0.0000000 | 0.0000000 | 0.0000000 | -5.1931705 | 0.0000000 | 0.0000000 | 0.0000000 | -0.2453578 | 0.0000000 | 0.0000000 | 0.0000000 | 0.0000000 | -0.5412270 | -1.0022140 | -0.0366605 | 0.0000000 | 50 | 5 | 71.80800 | 21.148193 |
| 0.3104880 | -2.5477711 | -2.5778561 | -3.4480965 | -0.9138033 | -1.0355812 | 0.0000000 | 0.0000000 | -0.1424829 | -1.1576880 | 0.0000000 | 0.0000000 | -4.7011746 | 0.0000000 | 0.0000000 | 0.0000000 | -0.1211817 | 0.0000000 | 0.0000000 | 0.0000000 | 0.0000000 | -0.1479499 | 0.0000000 | -0.8637607 | 0.0000000 | 50 | 6 | 66.53463 | 16.133192 |
