# Supplementary material for: Tropical Land‐Use Change Disrupts Zeta‐Diversity Across Taxa
Source: Glob Chang Biol. 2025 May 14;31(5):e70245. doi: 10.1111/gcb.70245 (PMC12076182; doi:10.1111/gcb.70245)
Supplement: Supplementary file 1 — Data S1. [file GCB-31-e70245-s001.zip › gcb70245-sup-0001-Appendix1.zip!/SM-4-DB_forest.html]

table output


MS-GDM Coefficients for dung beetle communities in natural habitats from 50 iterations (Binomial family (link=log) for each order of zeta and each predictor, using a random sample of 1000 site combinations for each MS-GDM. Here are the 50 replicates for each predictor, AIC, and deviance.

| (Intercept) | Elevation1 | Elevation2 | Elevation3 | Canopy.cover1 | Canopy.cover2 | Canopy.cover3 | Tree.density1 | Tree.density2 | Tree.density3 | Fragmentation1 | Fragmentation2 | Fragmentation3 | Forest.cover1 | Forest.cover2 | Forest.cover3 | Precipitation1 | Precipitation2 | Precipitation3 | Disturbance1 | Disturbance2 | Disturbance3 | distance1 | distance2 | distance3 | Rep | Group | AIC | Residual\_Deviance |
| --- | --- | --- | --- | --- | --- | --- | --- | --- | --- | --- | --- | --- | --- | --- | --- | --- | --- | --- | --- | --- | --- | --- | --- | --- | --- | --- | --- | --- |
| 0.0000000 | -1.5326416 | 0.0000000 | -1.2873511 | 0.0000000 | -0.3963759 | -0.2874369 | 0.0000000 | 0.0000000 | -0.0053358 | 0.0000000 | 0.0000000 | -0.4715989 | 0.0000000 | 0.0000000 | 0.0000000 | 0.0000000 | -0.0142633 | -0.4020302 | -0.0915071 | -0.7017336 | 0.0000000 | -0.1400531 | -0.3619701 | 0.0000000 | 1 | 1 | 489.4229 | 435.13655 |
| 0.3175845 | -0.9522371 | -0.7112979 | -1.5338552 | -11.2825405 | 0.0000000 | -0.9679879 | 0.0000000 | 0.0000000 | 0.0000000 | 0.0000000 | 0.0000000 | -1.4342218 | 0.0000000 | 0.0000000 | 0.0000000 | 0.0000000 | -0.2883018 | 0.0000000 | 0.0000000 | -0.7708571 | 0.0000000 | -0.4074234 | -0.1815752 | 0.0000000 | 1 | 2 | 420.6396 | 367.72590 |
| 0.5899874 | -1.4353015 | -0.5687714 | -2.2650956 | -11.5493120 | 0.0000000 | -1.7252665 | 0.0000000 | 0.0000000 | 0.0000000 | 0.0000000 | -0.0539117 | -0.8770128 | 0.0000000 | 0.0000000 | 0.0000000 | 0.0000000 | 0.0000000 | -0.1960842 | -0.1183405 | -1.3966015 | 0.0000000 | -0.6825612 | 0.0000000 | 0.0000000 | 1 | 3 | 373.1516 | 321.39165 |
| 1.8677636 | -2.1436250 | -0.6533263 | -2.0927606 | -13.1492323 | -0.0002714 | -2.0659974 | 0.0000000 | 0.0000000 | 0.0000000 | 0.0000000 | 0.0000000 | -0.7505525 | 0.0000000 | 0.0000000 | 0.0000000 | 0.0000000 | 0.0000000 | 0.0000000 | 0.0000000 | -2.9298121 | 0.0000000 | -1.2862242 | 0.0000000 | -0.4896351 | 1 | 4 | 334.9651 | 284.04115 |
| 2.1406052 | 0.0000000 | -3.0527983 | -0.6082835 | -22.9833306 | 0.0000000 | -3.8647414 | 0.0000000 | 0.0000000 | 0.0000000 | -0.1206368 | 0.0000000 | -2.4774357 | 0.0000000 | 0.0000000 | 0.0000000 | 0.0000000 | 0.0000000 | 0.0000000 | 0.0000000 | -2.9363384 | 0.0000000 | -0.4866304 | -0.3116804 | 0.0000000 | 1 | 5 | 208.0618 | 157.51677 |
| 1.7635315 | 0.0000000 | -1.5403251 | -5.0309897 | -42.3326181 | 0.0000000 | -3.1711094 | 0.0000000 | 0.0000000 | 0.0000000 | 0.0000000 | 0.0000000 | -2.8353313 | 0.0000000 | 0.0000000 | 0.0000000 | 0.0000000 | 0.0000000 | -1.2429675 | -0.2067228 | -2.3174534 | 0.0000000 | -0.3764214 | 0.0000000 | 0.0000000 | 1 | 6 | 157.8282 | 107.42074 |
| 0.0000000 | -1.2059994 | -0.4403884 | -0.7260766 | -10.1435050 | 0.0000000 | -0.2567337 | 0.0000000 | 0.0000000 | 0.0000000 | 0.0000000 | 0.0000000 | -0.9999835 | 0.0000000 | 0.0000000 | 0.0000000 | 0.0000000 | -0.0927447 | -0.5068091 | 0.0000000 | -0.4271254 | 0.0000000 | -0.0897797 | -0.3745040 | 0.0000000 | 2 | 1 | 483.9161 | 429.54091 |
| 0.1840321 | -0.5178069 | -0.9079138 | -0.7891903 | -18.9935328 | 0.0000000 | -1.1322299 | 0.0000000 | 0.0000000 | -0.0939707 | 0.0000000 | 0.0000000 | -0.2546629 | 0.0000000 | 0.0000000 | 0.0000000 | 0.0000000 | 0.0000000 | -0.1687021 | -0.3609047 | -0.6562209 | 0.0000000 | -0.4900128 | -0.0000507 | 0.0000000 | 2 | 2 | 455.4367 | 402.44201 |
| 0.9533222 | -0.4230387 | -1.5034434 | -1.5193879 | -13.6759020 | 0.0000000 | -1.8769157 | 0.0000000 | 0.0000000 | 0.0000000 | 0.0000000 | 0.0000000 | -0.2100572 | 0.0000000 | 0.0000000 | 0.0000000 | 0.0000000 | 0.0000000 | -0.5640768 | -1.0925540 | -0.9604507 | 0.0000000 | -0.9054785 | 0.0000000 | 0.0000000 | 2 | 3 | 374.4829 | 322.82038 |
| 1.0681957 | 0.0000000 | -2.4730214 | -0.9786868 | -25.0343067 | 0.0000000 | -1.4970479 | 0.0000000 | 0.0000000 | 0.0000000 | 0.0000000 | 0.0000000 | -1.3576003 | 0.0000000 | 0.0000000 | 0.0000000 | -0.0227889 | 0.0000000 | -0.5480749 | 0.0000000 | -1.7529055 | 0.0000000 | -0.6126540 | 0.0000000 | 0.0000000 | 2 | 4 | 291.6570 | 240.57316 |
| 2.0975189 | 0.0000000 | -4.4672868 | -1.9499446 | -24.2308823 | 0.0000000 | -3.1835316 | 0.0000000 | 0.0000000 | 0.0000000 | 0.0000000 | 0.0000000 | -1.9475371 | 0.0000000 | 0.0000000 | 0.0000000 | 0.0000000 | 0.0000000 | -1.0848715 | 0.0000000 | -1.9800509 | 0.0000000 | 0.0000000 | 0.0000000 | 0.0000000 | 2 | 5 | 215.7646 | 165.13316 |
| 0.9798231 | 0.0000000 | -2.6293366 | -5.5795359 | -5.8695478 | -0.4201730 | -5.4133657 | 0.0000000 | 0.0000000 | -0.5351969 | 0.0000000 | 0.0000000 | -3.8391483 | 0.0000000 | 0.0000000 | 0.0000000 | 0.0000000 | -0.0658639 | 0.0000000 | -0.7206058 | 0.0000000 | 0.0000000 | 0.0000000 | 0.0000000 | 0.0000000 | 2 | 6 | 142.2861 | 91.93674 |
| 0.0000000 | -1.1099827 | -0.3868856 | -0.9995635 | -10.9066475 | 0.0000000 | -0.4660449 | 0.0000000 | -0.0036560 | 0.0000000 | 0.0000000 | 0.0000000 | -0.5564197 | 0.0000000 | 0.0000000 | 0.0000000 | 0.0000000 | -0.1867842 | -0.5233538 | -0.3876475 | -0.2618391 | 0.0000000 | -0.0434684 | -0.3737768 | 0.0000000 | 3 | 1 | 494.7458 | 440.47329 |
| 0.0903596 | -1.0830888 | -0.4885528 | -0.8122801 | -9.6955463 | -0.1954793 | -0.6671952 | -0.0006893 | -0.0003144 | 0.0000000 | 0.0000000 | -0.0138153 | -0.7635850 | -0.0127174 | -0.0130460 | 0.0000000 | -0.0096473 | -0.0127076 | -0.3586159 | -0.0142945 | -0.9930083 | 0.0000000 | -0.2266548 | -0.2637241 | -0.0019029 | 3 | 2 | 453.0930 | 399.94358 |
| 1.1697782 | -0.2724443 | -1.5960603 | -2.1665704 | -12.5655680 | 0.0000000 | -1.1361111 | 0.0000000 | 0.0000000 | 0.0000000 | 0.0000000 | 0.0000000 | -1.2527420 | 0.0000000 | 0.0000000 | 0.0000000 | 0.0000000 | 0.0000000 | -0.7913552 | -0.1470181 | -1.3665479 | 0.0000000 | -1.1036208 | 0.0000000 | 0.0000000 | 3 | 3 | 336.7758 | 284.94847 |
| 1.9055896 | 0.0000000 | -2.9981261 | -0.7243227 | -32.7386770 | 0.0000000 | -2.0042632 | 0.0000000 | 0.0000000 | 0.0000000 | 0.0000000 | 0.0000000 | -1.5314902 | 0.0000000 | 0.0000000 | 0.0000000 | 0.0000000 | 0.0000000 | 0.0000000 | 0.0000000 | -1.9118521 | 0.0000000 | -1.1706262 | 0.0000000 | -0.0342633 | 3 | 4 | 297.1302 | 246.10044 |
| 1.4512711 | 0.0000000 | -3.0152524 | -4.1746341 | -20.7700836 | 0.0000000 | -3.8027954 | 0.0000000 | 0.0000000 | 0.0000000 | 0.0000000 | 0.0000000 | -2.2118594 | 0.0000000 | 0.0000000 | 0.0000000 | -0.4468334 | 0.0000000 | -0.7833695 | 0.0000000 | -0.9012330 | 0.0000000 | 0.0000000 | -0.3008876 | 0.0000000 | 3 | 5 | 203.2596 | 152.65651 |
| 1.2996352 | -0.5022444 | -2.2579785 | -5.4610566 | -58.2059601 | 0.0000000 | -3.3984158 | 0.0000000 | 0.0000000 | 0.0000000 | 0.0000000 | 0.0000000 | -3.5748002 | 0.0000000 | 0.0000000 | 0.0000000 | -0.1746739 | 0.0000000 | -0.5758134 | -2.1470766 | 0.0000000 | 0.0000000 | -0.4491663 | 0.0000000 | 0.0000000 | 3 | 6 | 153.1772 | 102.85496 |
| 0.0000000 | -1.1081412 | -0.2127800 | -0.8106812 | -10.6305937 | 0.0000000 | -0.5228996 | 0.0000000 | 0.0000000 | -0.0059025 | 0.0000000 | -0.0367881 | -0.0724092 | 0.0000000 | 0.0000000 | 0.0000000 | 0.0000000 | -0.1159617 | -0.3911204 | -0.4814315 | -0.3753564 | 0.0000000 | -0.1666016 | -0.4372715 | 0.0000000 | 4 | 1 | 481.2118 | 426.83481 |
| 0.2959973 | -0.8898167 | -0.5089259 | -0.8514851 | -24.5070719 | 0.0000000 | -0.9683785 | 0.0000000 | 0.0000000 | 0.0000000 | 0.0000000 | 0.0000000 | -0.8511747 | 0.0000000 | 0.0000000 | 0.0000000 | 0.0000000 | 0.0000000 | -0.1382938 | 0.0000000 | -1.2505390 | 0.0000000 | -0.5658868 | 0.0000000 | 0.0000000 | 4 | 2 | 442.4673 | 389.55326 |
| 0.7164196 | -0.4097593 | -1.0408478 | -2.9538981 | -28.5597523 | 0.0000000 | -2.2969470 | 0.0000000 | 0.0000000 | 0.0000000 | 0.0000000 | 0.0000000 | -1.0303991 | 0.0000000 | 0.0000000 | 0.0000000 | 0.0000000 | 0.0000000 | -0.9183343 | -0.4502363 | -0.7896469 | 0.0000000 | -0.5919738 | 0.0000000 | 0.0000000 | 4 | 3 | 337.6423 | 285.95195 |
| 2.3235812 | 0.0000000 | -3.2739462 | -1.9798462 | -22.9733451 | 0.0000000 | -2.8362569 | 0.0000000 | 0.0000000 | -0.1075109 | 0.0000000 | 0.0000000 | -0.8004751 | 0.0000000 | 0.0000000 | 0.0000000 | 0.0000000 | -0.5941898 | 0.0000000 | -0.5290302 | -1.0243683 | 0.0000000 | -1.0905167 | 0.0000000 | 0.0000000 | 4 | 4 | 301.4772 | 250.39078 |
| 1.3232097 | 0.0000000 | -2.3064937 | -4.6159470 | -32.7820529 | 0.0000000 | -1.5684318 | 0.0000000 | -0.1317879 | 0.0000000 | 0.0000000 | 0.0000000 | -2.2007051 | 0.0000000 | 0.0000000 | 0.0000000 | 0.0000000 | 0.0000000 | 0.0000000 | 0.0000000 | -1.4509770 | 0.0000000 | -1.1740867 | 0.0000000 | 0.0000000 | 4 | 5 | 184.2629 | 133.65292 |
| 1.3747473 | 0.0000000 | -3.8950411 | -0.1361758 | -52.1800032 | 0.0000000 | -3.5583434 | 0.0000000 | 0.0000000 | 0.0000000 | 0.0000000 | 0.0000000 | -3.6252834 | 0.0000000 | 0.0000000 | 0.0000000 | 0.0000000 | 0.0000000 | -0.3210669 | -0.1003289 | -1.4432429 | 0.0000000 | 0.0000000 | 0.0000000 | 0.0000000 | 4 | 6 | 177.1295 | 126.71813 |
| 0.0000000 | -1.1344147 | -0.2816916 | -1.3265512 | -4.5722719 | 0.0000000 | -0.4484646 | 0.0000000 | 0.0000000 | -0.0223835 | 0.0000000 | 0.0000000 | -0.5647669 | 0.0000000 | 0.0000000 | 0.0000000 | -0.0019418 | -0.0206237 | -0.5101429 | 0.0000000 | -0.7032117 | 0.0000000 | -0.0972895 | -0.2532886 | 0.0000000 | 5 | 1 | 499.5937 | 445.41133 |
| 0.2421028 | -1.4355837 | -0.1624736 | -2.1308093 | -11.4908667 | -0.1969196 | -1.0464898 | -0.0004696 | 0.0000000 | 0.0000000 | 0.0000000 | -0.0019229 | -0.2349695 | -0.0027623 | -0.0019164 | 0.0000000 | 0.0000000 | -0.0596895 | -0.1015360 | -0.5609554 | -0.7550988 | 0.0000000 | -0.4013909 | -0.3325965 | -0.0012192 | 5 | 2 | 447.4810 | 394.59602 |
| 1.1292870 | -1.1063140 | -0.7232361 | -2.2905707 | -11.2610360 | 0.0000000 | -1.8213214 | 0.0000000 | 0.0000000 | 0.0000000 | 0.0000000 | 0.0000000 | -0.9027682 | 0.0000000 | 0.0000000 | 0.0000000 | 0.0000000 | 0.0000000 | -0.3911619 | -0.4539375 | -1.4131759 | 0.0000000 | -1.0715571 | 0.0000000 | 0.0000000 | 5 | 3 | 369.1248 | 317.32401 |
| 0.9162967 | 0.0000000 | -2.0720724 | -3.4486456 | -27.1354391 | 0.0000000 | -1.3084831 | 0.0000000 | 0.0000000 | 0.0000000 | 0.0000000 | 0.0000000 | 0.0000000 | 0.0000000 | 0.0000000 | 0.0000000 | -0.0233851 | 0.0000000 | -0.0585276 | -0.9915119 | -1.5130149 | 0.0000000 | -0.5100496 | -0.4060302 | 0.0000000 | 5 | 4 | 278.3896 | 227.39240 |
| 2.3075288 | 0.0000000 | -4.2477620 | -1.1504431 | -23.0712199 | -3.4553566 | -3.6097395 | 0.0000000 | 0.0000000 | 0.0000000 | 0.0000000 | 0.0000000 | -2.6955514 | 0.0000000 | 0.0000000 | 0.0000000 | 0.0000000 | 0.0000000 | -0.4918008 | 0.0000000 | -1.8402625 | 0.0000000 | -0.2331601 | 0.0000000 | 0.0000000 | 5 | 5 | 209.3591 | 158.82876 |
| 1.5567961 | -0.0896262 | -0.4099381 | -9.4136992 | -13.3761792 | 0.0000000 | -4.4428802 | 0.0000000 | 0.0000000 | 0.0000000 | 0.0000000 | 0.0000000 | -3.8001903 | 0.0000000 | 0.0000000 | 0.0000000 | -0.3083076 | -0.3619603 | -0.9733850 | -1.9802659 | 0.0000000 | 0.0000000 | -0.6765241 | 0.0000000 | 0.0000000 | 5 | 6 | 146.6024 | 96.29481 |
| 0.0000000 | -1.3930988 | -0.0699250 | -1.5225197 | -7.7200286 | 0.0000000 | -0.3674743 | 0.0000000 | 0.0000000 | -0.0307999 | 0.0000000 | 0.0000000 | -0.6103336 | 0.0000000 | 0.0000000 | 0.0000000 | 0.0000000 | -0.1715266 | -0.4899135 | 0.0000000 | -0.5606648 | 0.0000000 | -0.2138035 | -0.1718403 | 0.0000000 | 6 | 1 | 451.5926 | 397.16134 |
| 0.2108059 | -0.8369313 | -0.3767477 | -1.7514455 | -9.5057145 | 0.0000000 | -1.3672574 | 0.0000000 | 0.0000000 | 0.0000000 | 0.0000000 | 0.0000000 | -0.3085809 | 0.0000000 | 0.0000000 | 0.0000000 | -0.0202654 | 0.0000000 | -0.4556621 | -0.7153288 | -0.5430507 | 0.0000000 | -0.4851370 | 0.0000000 | 0.0000000 | 6 | 2 | 436.8907 | 383.78651 |
| 1.5825991 | 0.0000000 | -2.6649476 | -1.4128726 | -24.7959627 | 0.0000000 | -1.8972193 | 0.0000000 | 0.0000000 | 0.0000000 | 0.0000000 | 0.0000000 | -1.4047029 | 0.0000000 | 0.0000000 | 0.0000000 | 0.0000000 | 0.0000000 | -0.0865295 | 0.0000000 | -1.6829627 | 0.0000000 | -0.9359283 | 0.0000000 | -0.1533924 | 6 | 3 | 316.0624 | 264.37804 |
| 1.0619851 | -1.2750486 | -0.4752719 | -3.5782490 | -35.1547029 | 0.0000000 | -1.4148645 | 0.0000000 | 0.0000000 | 0.0000000 | -0.2343041 | 0.0000000 | -1.4156616 | 0.0000000 | 0.0000000 | 0.0000000 | 0.0000000 | -0.3865472 | -0.0857339 | -0.6731481 | -1.0819454 | 0.0000000 | -1.0549194 | -0.1004476 | 0.0000000 | 6 | 4 | 285.5276 | 234.56632 |
| 3.8377446 | 0.0000000 | -2.9147014 | -1.3711563 | -29.0878153 | 0.0000000 | -3.5064001 | 0.0000000 | 0.0000000 | 0.0000000 | 0.0000000 | 0.0000000 | -1.3296808 | 0.0000000 | 0.0000000 | 0.0000000 | 0.0000000 | -0.0880587 | 0.0000000 | 0.0000000 | -4.0393442 | 0.0000000 | -1.5085615 | -0.6067635 | -1.1214528 | 6 | 5 | 181.6229 | 131.06743 |
| 2.9646443 | -0.7346810 | -2.7141873 | -2.0342678 | -14.3232812 | -1.2779351 | -4.5555699 | 0.0000000 | 0.0000000 | -0.6584259 | 0.0000000 | 0.0000000 | -3.9889713 | 0.0000000 | 0.0000000 | 0.0000000 | -0.8281264 | 0.0000000 | -0.0250309 | -0.5187248 | -1.9875953 | 0.0000000 | -1.0242710 | 0.0000000 | -0.2051209 | 6 | 6 | 157.4285 | 107.13343 |
| 0.0000000 | -1.1518302 | -0.3077807 | -0.7618302 | -6.8250890 | 0.0000000 | -0.4220497 | 0.0000000 | 0.0000000 | -0.0259593 | 0.0000000 | 0.0000000 | -0.6729009 | 0.0000000 | 0.0000000 | 0.0000000 | 0.0000000 | 0.0000000 | -0.5301126 | -0.1500649 | -0.5904460 | 0.0000000 | -0.0378769 | -0.5115661 | 0.0000000 | 7 | 1 | 464.3463 | 410.04315 |
| 0.3028574 | -1.3834733 | -0.2204122 | -1.9426845 | -15.8629329 | 0.0000000 | -0.8154124 | 0.0000000 | 0.0000000 | 0.0000000 | 0.0000000 | 0.0000000 | -0.3804823 | 0.0000000 | 0.0000000 | 0.0000000 | 0.0000000 | -0.0186686 | -0.2481095 | 0.0000000 | -1.0423424 | 0.0000000 | -0.5513757 | 0.0000000 | 0.0000000 | 7 | 2 | 471.0540 | 418.06103 |
| 0.8144189 | -1.0184913 | -0.8445223 | -3.5730077 | -4.2467079 | 0.0000000 | -1.3185825 | 0.0000000 | 0.0000000 | 0.0000000 | 0.0000000 | 0.0000000 | -1.4744794 | 0.0000000 | 0.0000000 | 0.0000000 | 0.0000000 | 0.0000000 | -0.3825157 | -0.0821085 | -1.4741011 | 0.0000000 | -0.7630737 | 0.0000000 | 0.0000000 | 7 | 3 | 365.4213 | 313.62606 |
| 1.6418065 | -0.7054180 | -1.8271530 | -1.5684806 | -37.9588234 | 0.0000000 | -2.4915213 | 0.0000000 | 0.0000000 | 0.0000000 | 0.0000000 | 0.0000000 | -0.3005444 | 0.0000000 | 0.0000000 | 0.0000000 | -0.0684560 | 0.0000000 | -0.2728560 | 0.0000000 | -2.8600285 | 0.0000000 | -0.3393181 | -0.2831467 | 0.0000000 | 7 | 4 | 331.3194 | 280.28049 |
| 1.0967088 | 0.0000000 | -3.8367320 | -2.4502517 | -17.0955439 | 0.0000000 | -1.8879888 | 0.0000000 | 0.0000000 | 0.0000000 | 0.0000000 | 0.0000000 | -1.9016341 | 0.0000000 | 0.0000000 | 0.0000000 | -0.0983085 | 0.0000000 | 0.0000000 | 0.0000000 | -1.3993183 | 0.0000000 | -0.4566287 | 0.0000000 | 0.0000000 | 7 | 5 | 214.2134 | 163.56484 |
| 0.7444689 | 0.0000000 | -3.0267527 | -2.0593854 | -26.5066276 | 0.0000000 | -4.3310309 | 0.0000000 | 0.0000000 | 0.0000000 | 0.0000000 | 0.0000000 | -2.7517965 | 0.0000000 | 0.0000000 | 0.0000000 | 0.0000000 | 0.0000000 | -0.9663382 | 0.0000000 | -0.1007558 | 0.0000000 | 0.0000000 | 0.0000000 | 0.0000000 | 7 | 6 | 159.5113 | 109.16613 |
| 0.0000000 | -1.0224032 | -0.2861957 | -1.1011047 | -6.3954893 | 0.0000000 | -0.2623040 | 0.0000000 | 0.0000000 | -0.0857763 | 0.0000000 | 0.0000000 | -0.2002785 | 0.0000000 | 0.0000000 | 0.0000000 | 0.0000000 | -0.0461260 | -0.4430087 | -0.2426742 | -0.5377180 | 0.0000000 | -0.1098991 | -0.4608502 | 0.0000000 | 8 | 1 | 482.5178 | 428.14827 |
| 0.2644623 | -0.9669726 | -0.6942805 | -0.8846721 | -18.2893582 | 0.0000000 | -0.9645219 | 0.0000000 | -0.1295812 | 0.0000000 | 0.0000000 | 0.0000000 | -0.7296245 | 0.0000000 | 0.0000000 | 0.0000000 | 0.0000000 | 0.0000000 | -0.4883100 | 0.0000000 | -0.9593696 | 0.0000000 | -0.1350274 | -0.3944548 | 0.0000000 | 8 | 2 | 461.0399 | 408.02987 |
| 0.7534747 | -0.2805351 | -1.6155131 | -1.9668877 | -21.1416387 | 0.0000000 | -1.0482738 | 0.0000000 | 0.0000000 | 0.0000000 | 0.0000000 | 0.0000000 | -1.3598961 | 0.0000000 | 0.0000000 | 0.0000000 | 0.0000000 | 0.0000000 | -0.2083320 | 0.0000000 | -0.8521198 | 0.0000000 | -1.1219208 | 0.0000000 | 0.0000000 | 8 | 3 | 363.7547 | 312.08291 |
| 2.6268833 | 0.0000000 | -2.7553166 | -2.3791702 | -12.0739791 | 0.0000000 | -2.0955324 | 0.0000000 | 0.0000000 | -0.0247972 | 0.0000000 | 0.0000000 | -1.6123864 | 0.0000000 | 0.0000000 | 0.0000000 | 0.0000000 | -0.7879508 | -0.0079026 | 0.0000000 | -2.0114437 | 0.0000000 | -1.3193593 | 0.0000000 | 0.0000000 | 8 | 4 | 293.9355 | 242.88502 |
| 1.7384402 | 0.0000000 | -2.6798814 | -0.8828050 | -38.2892784 | 0.0000000 | -3.8402125 | -0.0882039 | 0.0000000 | 0.0000000 | 0.0000000 | 0.0000000 | -0.2401429 | 0.0000000 | 0.0000000 | 0.0000000 | -0.1026374 | -0.1520689 | 0.0000000 | 0.0000000 | -1.8220496 | 0.0000000 | -0.7256953 | 0.0000000 | 0.0000000 | 8 | 5 | 250.2545 | 199.56687 |
| 2.2096140 | -0.7565962 | -2.2855238 | -7.0142433 | -25.3406376 | 0.0000000 | -4.8076181 | 0.0000000 | 0.0000000 | 0.0000000 | 0.0000000 | 0.0000000 | -4.8072189 | 0.0000000 | 0.0000000 | 0.0000000 | 0.0000000 | 0.0000000 | -0.2988895 | 0.0000000 | 0.0000000 | -0.3698317 | -0.5686383 | -0.9784551 | 0.0000000 | 8 | 6 | 136.3600 | 85.94849 |
| 0.0110367 | -1.4673517 | 0.0000000 | -0.9702997 | -12.3907674 | 0.0000000 | -0.7141977 | 0.0000000 | 0.0000000 | 0.0000000 | 0.0000000 | 0.0000000 | -0.5603664 | 0.0000000 | 0.0000000 | 0.0000000 | -0.0464800 | -0.2302830 | -0.2942668 | 0.0000000 | -0.7055764 | 0.0000000 | -0.0384146 | -0.3699675 | 0.0000000 | 9 | 1 | 474.5362 | 420.18357 |
| 0.1753247 | -1.6544443 | -0.3071078 | -1.3809070 | -12.2923247 | 0.0000000 | -1.0758899 | 0.0000000 | 0.0000000 | 0.0000000 | 0.0000000 | 0.0000000 | -1.0497435 | 0.0000000 | 0.0000000 | 0.0000000 | 0.0000000 | 0.0000000 | -0.3222593 | 0.0000000 | -1.1438674 | 0.0000000 | -0.1222482 | -0.2797994 | 0.0000000 | 9 | 2 | 461.3434 | 408.60242 |
| 0.2513931 | -0.5451818 | -1.0276729 | -2.0353397 | -10.6723021 | 0.0000000 | -2.0919122 | 0.0000000 | 0.0000000 | 0.0000000 | 0.0000000 | 0.0000000 | -0.2515365 | 0.0000000 | 0.0000000 | 0.0000000 | 0.0000000 | 0.0000000 | -0.7026264 | -0.4769246 | -1.1841938 | 0.0000000 | -0.0264122 | -0.0960366 | 0.0000000 | 9 | 3 | 389.4398 | 337.62737 |
| 1.5970931 | -0.0869616 | -2.1265523 | -2.3325910 | -27.0865102 | 0.0000000 | -2.3991736 | 0.0000000 | 0.0000000 | 0.0000000 | 0.0000000 | 0.0000000 | -1.5242519 | 0.0000000 | 0.0000000 | 0.0000000 | 0.0000000 | 0.0000000 | 0.0000000 | 0.0000000 | -1.5247088 | 0.0000000 | -1.1276502 | 0.0000000 | 0.0000000 | 9 | 4 | 300.5153 | 249.53860 |
| 3.4560126 | -0.1335089 | -2.4087808 | -2.8461594 | -22.8823898 | 0.0000000 | -2.2126952 | 0.0000000 | 0.0000000 | -0.3817633 | 0.0000000 | 0.0000000 | -1.5287572 | 0.0000000 | 0.0000000 | 0.0000000 | 0.0000000 | -0.8205496 | -0.0659508 | 0.0000000 | -3.4911169 | 0.0000000 | -1.5348688 | 0.0000000 | -0.5871170 | 9 | 5 | 229.2867 | 178.70999 |
| 1.8752028 | 0.0000000 | -3.0778125 | -5.9764071 | -6.8936226 | 0.0000000 | -2.9733301 | 0.0000000 | 0.0000000 | 0.0000000 | 0.0000000 | 0.0000000 | -5.1048667 | 0.0000000 | 0.0000000 | 0.0000000 | 0.0000000 | -0.3991690 | 0.0000000 | 0.0000000 | -2.3166523 | 0.0000000 | 0.0000000 | 0.0000000 | 0.0000000 | 9 | 6 | 154.8560 | 104.44633 |
| 0.0000000 | -1.0585150 | -0.1890836 | -1.2129723 | -7.7699394 | 0.0000000 | -0.3531680 | 0.0000000 | 0.0000000 | 0.0000000 | 0.0000000 | 0.0000000 | -0.5079069 | 0.0000000 | 0.0000000 | 0.0000000 | 0.0000000 | -0.2005367 | -0.2337312 | 0.0000000 | -0.7383604 | 0.0000000 | -0.0718935 | -0.3652678 | 0.0000000 | 10 | 1 | 464.0327 | 409.63686 |
| 0.4818993 | -1.0438462 | -0.5667719 | -1.3958125 | -9.3855939 | 0.0000000 | -1.0697501 | 0.0000000 | 0.0000000 | 0.0000000 | 0.0000000 | 0.0000000 | -0.7684700 | 0.0000000 | 0.0000000 | 0.0000000 | 0.0000000 | -0.3865698 | -0.0231907 | -0.0169753 | -0.7746884 | 0.0000000 | -0.6965819 | 0.0000000 | 0.0000000 | 10 | 2 | 482.2629 | 429.40563 |
| 0.8089398 | -0.4460025 | -1.3086640 | -2.0328862 | -16.4028556 | 0.0000000 | -1.8286685 | 0.0000000 | 0.0000000 | 0.0000000 | 0.0000000 | 0.0000000 | -0.5915543 | 0.0000000 | 0.0000000 | 0.0000000 | 0.0000000 | -0.2671201 | -0.3319728 | -0.7921442 | -0.4227351 | 0.0000000 | -1.0010883 | 0.0000000 | 0.0000000 | 10 | 3 | 362.9444 | 311.15291 |
| 1.3802642 | -0.8774568 | -0.6464207 | -3.2936664 | -23.6508657 | 0.0000000 | -2.5357436 | 0.0000000 | 0.0000000 | 0.0000000 | 0.0000000 | 0.0000000 | -1.7720144 | 0.0000000 | 0.0000000 | 0.0000000 | 0.0000000 | -0.8706423 | 0.0000000 | 0.0000000 | -2.2858214 | 0.0000000 | -0.4225616 | 0.0000000 | 0.0000000 | 10 | 4 | 292.9828 | 241.95814 |
| 1.6987996 | 0.0000000 | -2.1423844 | -5.6066949 | -21.0638988 | 0.0000000 | -3.8671162 | 0.0000000 | 0.0000000 | 0.0000000 | 0.0000000 | 0.0000000 | -1.3003636 | 0.0000000 | 0.0000000 | 0.0000000 | -0.1138893 | 0.0000000 | -1.0825143 | -0.5436163 | -0.7410792 | 0.0000000 | -0.6667926 | 0.0000000 | -1.7449164 | 10 | 5 | 184.3961 | 133.81478 |
| 1.7021605 | -0.0396884 | -0.6710057 | -6.4874887 | -1.0490229 | 0.0000000 | -3.4782613 | 0.0000000 | 0.0000000 | 0.0000000 | -0.9031597 | 0.0000000 | -1.8491129 | 0.0000000 | 0.0000000 | 0.0000000 | 0.0000000 | -1.5655958 | 0.0000000 | -0.6071826 | 0.0000000 | 0.0000000 | -1.1869804 | 0.0000000 | 0.0000000 | 10 | 6 | 152.1157 | 101.83124 |
| 0.0000000 | -1.5303134 | -0.2051654 | -1.4510651 | -0.6189872 | -0.1559188 | -0.2420537 | 0.0000000 | 0.0000000 | 0.0000000 | 0.0000000 | 0.0000000 | -0.2533816 | 0.0000000 | 0.0000000 | 0.0000000 | 0.0000000 | -0.1159058 | -0.5582291 | -0.1182156 | -0.6854442 | 0.0000000 | 0.0000000 | -0.4128289 | 0.0000000 | 11 | 1 | 475.3387 | 421.09106 |
| 0.3002171 | -0.9500534 | -0.6591145 | -1.0401167 | -15.8985601 | 0.0000000 | -1.1271446 | 0.0000000 | 0.0000000 | 0.0000000 | 0.0000000 | 0.0000000 | -0.7517852 | 0.0000000 | 0.0000000 | 0.0000000 | 0.0000000 | -0.4514304 | -0.2751891 | 0.0000000 | -0.7251487 | 0.0000000 | -0.3175481 | -0.1754412 | 0.0000000 | 11 | 2 | 448.2308 | 395.38260 |
| 1.0808276 | -0.0193070 | -1.9879722 | -1.1380007 | -21.3136605 | 0.0000000 | -1.4913918 | 0.0000000 | 0.0000000 | 0.0000000 | 0.0000000 | 0.0000000 | -1.1081629 | 0.0000000 | 0.0000000 | 0.0000000 | 0.0000000 | 0.0000000 | -0.6263886 | 0.0000000 | -1.7151090 | 0.0000000 | -0.6980051 | 0.0000000 | 0.0000000 | 11 | 3 | 368.0738 | 316.23185 |
| 1.1195678 | 0.0000000 | -2.8869778 | -1.2712807 | -18.6320188 | 0.0000000 | -1.6313957 | 0.0000000 | 0.0000000 | 0.0000000 | 0.0000000 | 0.0000000 | -1.4325484 | 0.0000000 | 0.0000000 | 0.0000000 | -0.2323693 | 0.0000000 | 0.0000000 | 0.0000000 | -2.3149245 | 0.0000000 | -0.3060100 | 0.0000000 | 0.0000000 | 11 | 4 | 293.7502 | 242.89078 |
| 2.6094017 | 0.0000000 | -3.4783326 | -2.4222596 | -29.5910333 | 0.0000000 | -3.3387550 | 0.0000000 | 0.0000000 | 0.0000000 | 0.0000000 | 0.0000000 | -4.4701033 | 0.0000000 | 0.0000000 | 0.0000000 | 0.0000000 | 0.0000000 | 0.0000000 | 0.0000000 | -1.8108896 | 0.0000000 | -1.2400781 | 0.0000000 | 0.0000000 | 11 | 5 | 190.6833 | 140.08040 |
| 2.2473792 | 0.0000000 | -3.2940916 | -0.9095506 | -23.4988895 | 0.0000000 | -4.3038287 | 0.0000000 | 0.0000000 | 0.0000000 | 0.0000000 | 0.0000000 | -2.8399908 | 0.0000000 | 0.0000000 | 0.0000000 | 0.0000000 | 0.0000000 | 0.0000000 | -1.1694820 | -0.7852964 | 0.0000000 | -1.4160565 | 0.0000000 | 0.0000000 | 11 | 6 | 176.2513 | 125.84014 |
| 0.0000000 | -0.9078745 | -0.4396082 | -0.9586099 | -13.1105859 | 0.0000000 | -0.3450190 | 0.0000000 | 0.0000000 | -0.0551286 | 0.0000000 | 0.0000000 | -0.5139939 | 0.0000000 | 0.0000000 | 0.0000000 | -0.1013185 | -0.0491715 | -0.5426380 | -0.0587828 | -0.5507081 | 0.0000000 | -0.1591221 | -0.2269710 | 0.0000000 | 12 | 1 | 465.5739 | 411.15599 |
| 0.1891951 | -0.9618130 | -0.2404769 | -1.3947336 | -9.1793179 | 0.0000000 | -1.1905213 | 0.0000000 | 0.0000000 | 0.0000000 | 0.0000000 | 0.0000000 | -0.9897765 | 0.0000000 | 0.0000000 | 0.0000000 | 0.0000000 | 0.0000000 | -0.2673662 | -0.1028507 | -0.6737370 | 0.0000000 | -0.6602867 | 0.0000000 | 0.0000000 | 12 | 2 | 477.2786 | 424.36986 |
| 1.2797494 | -0.4649370 | -1.8556629 | -0.6995336 | -19.6116046 | -0.1508196 | -2.0749273 | 0.0000000 | 0.0000000 | 0.0000000 | 0.0000000 | -0.0001810 | -1.1578979 | -0.0002391 | -0.0001869 | 0.0000000 | 0.0000000 | -0.0002423 | -0.3662784 | -0.0002828 | -1.8896261 | 0.0000000 | -0.8247892 | -0.0001435 | -0.0000050 | 12 | 3 | 386.8642 | 335.28248 |
| 2.1231970 | -0.4260315 | -1.2139227 | -4.2826524 | -33.9994381 | 0.0000000 | -2.5863132 | 0.0000000 | 0.0000000 | 0.0000000 | 0.0000000 | 0.0000000 | -1.9498316 | 0.0000000 | 0.0000000 | 0.0000000 | 0.0000000 | 0.0000000 | -0.3390836 | -0.4844608 | -1.6908271 | 0.0000000 | -1.6212877 | 0.0000000 | 0.0000000 | 12 | 4 | 236.8995 | 186.02305 |
| 0.0424962 | -0.4948564 | -1.8331186 | -3.0507884 | -27.7613556 | 0.0000000 | -1.4244753 | 0.0000000 | 0.0000000 | 0.0000000 | 0.0000000 | 0.0000000 | -4.9509856 | 0.0000000 | 0.0000000 | 0.0000000 | 0.0000000 | 0.0000000 | -0.6806156 | 0.0000000 | -1.0088070 | 0.0000000 | 0.0000000 | 0.0000000 | 0.0000000 | 12 | 5 | 203.7604 | 153.24778 |
| 6.5813088 | 0.0000000 | -2.5883927 | -5.8022289 | -9.9600210 | -6.9097917 | -2.3301629 | 0.0000000 | 0.0000000 | -0.4389448 | 0.0000000 | 0.0000000 | -2.0929750 | 0.0000000 | 0.0000000 | 0.0000000 | -0.3856105 | 0.0000000 | 0.0000000 | -2.9726972 | -1.0663557 | 0.0000000 | -4.7593306 | 0.0000000 | 0.0000000 | 12 | 6 | 144.1482 | 93.83381 |
| 0.0000000 | -1.2611827 | -0.2666568 | -1.0248838 | -12.6243524 | 0.0000000 | -0.5336903 | 0.0000000 | 0.0000000 | 0.0000000 | 0.0000000 | 0.0000000 | -0.2169589 | 0.0000000 | 0.0000000 | 0.0000000 | -0.0159163 | -0.0418815 | -0.3767389 | -0.3399904 | -0.4877816 | 0.0000000 | -0.0861950 | -0.4141141 | 0.0000000 | 13 | 1 | 438.4268 | 383.80206 |
| 0.5240326 | -0.9936104 | -0.7011567 | -1.3838596 | -8.1469982 | 0.0000000 | -1.2586408 | 0.0000000 | 0.0000000 | 0.0000000 | 0.0000000 | 0.0000000 | -1.1092020 | 0.0000000 | 0.0000000 | 0.0000000 | 0.0000000 | 0.0000000 | -0.4078940 | 0.0000000 | -1.0632914 | 0.0000000 | -0.5574692 | 0.0000000 | 0.0000000 | 13 | 2 | 445.8158 | 392.95356 |
| 0.8379148 | -1.3614820 | -0.3675323 | -2.2950295 | -14.8787035 | 0.0000000 | -1.5606905 | 0.0000000 | 0.0000000 | -0.1279286 | 0.0000000 | 0.0000000 | -1.1123300 | 0.0000000 | 0.0000000 | 0.0000000 | -0.2056419 | 0.0000000 | 0.0000000 | 0.0000000 | -1.5806392 | 0.0000000 | -1.1446362 | 0.0000000 | 0.0000000 | 13 | 3 | 339.1854 | 287.47629 |
| 1.5436164 | -0.4803311 | -1.3902596 | -2.9459535 | -19.6035720 | 0.0000000 | -2.4846388 | 0.0000000 | 0.0000000 | 0.0000000 | 0.0000000 | 0.0000000 | -2.4758409 | 0.0000000 | 0.0000000 | 0.0000000 | 0.0000000 | 0.0000000 | -0.0268702 | -0.2608983 | -1.1433965 | 0.0000000 | -1.5093444 | 0.0000000 | 0.0000000 | 13 | 4 | 297.5777 | 246.59016 |
| 3.3000349 | 0.0000000 | -3.3806928 | -3.0964944 | -18.0574798 | -0.0000295 | -2.9630846 | 0.0000000 | 0.0000000 | -0.6724044 | 0.0000000 | 0.0000000 | -2.8193749 | 0.0000000 | 0.0000000 | 0.0000000 | -0.0167243 | -0.4527341 | 0.0000000 | -0.3108444 | -1.5646272 | 0.0000000 | -1.3253599 | -0.6365133 | 0.0000000 | 13 | 5 | 214.1305 | 163.43988 |
| 3.8110175 | 0.0000000 | -4.0864383 | -1.4431457 | -28.0341662 | 0.0000000 | -3.7258974 | 0.0000000 | 0.0000000 | 0.0000000 | -1.0323490 | 0.0000000 | -3.1834523 | 0.0000000 | 0.0000000 | 0.0000000 | 0.0000000 | 0.0000000 | 0.0000000 | -0.8230898 | -1.8563249 | 0.0000000 | -0.3636559 | -1.7113989 | 0.0000000 | 13 | 6 | 179.7096 | 129.35770 |
| 0.0000000 | -0.8711736 | -0.4618028 | -0.3769602 | -13.6080008 | 0.0000000 | -0.3622154 | 0.0000000 | 0.0000000 | 0.0000000 | 0.0000000 | 0.0000000 | -0.4893395 | 0.0000000 | 0.0000000 | 0.0000000 | 0.0000000 | -0.1564416 | -0.4041841 | -0.0116985 | -0.5674177 | 0.0000000 | -0.1376370 | -0.3530411 | 0.0000000 | 14 | 1 | 459.7084 | 405.30904 |
| 0.0000000 | -0.5663903 | -0.3276514 | -0.7171475 | -23.9961927 | 0.0000000 | -0.4739497 | 0.0000000 | 0.0000000 | 0.0000000 | 0.0000000 | 0.0000000 | -1.0499525 | 0.0000000 | 0.0000000 | 0.0000000 | 0.0000000 | -0.2904666 | 0.0000000 | 0.0000000 | -0.8106722 | 0.0000000 | -0.5133712 | 0.0000000 | 0.0000000 | 14 | 2 | 467.7352 | 414.83297 |
| 1.1254597 | -0.6859955 | -1.4549985 | -2.1038284 | -20.4362829 | 0.0000000 | -2.1449700 | 0.0000000 | 0.0000000 | 0.0000000 | 0.0000000 | 0.0000000 | -0.7128364 | 0.0000000 | 0.0000000 | 0.0000000 | 0.0000000 | 0.0000000 | -0.4911558 | -0.4814303 | -0.9597424 | 0.0000000 | -0.9388919 | 0.0000000 | 0.0000000 | 14 | 3 | 361.0293 | 309.34397 |
| 1.7230214 | -0.8724305 | -1.5076507 | -3.1172157 | -28.7293053 | 0.0000000 | -2.7314082 | 0.0000000 | 0.0000000 | 0.0000000 | 0.0000000 | 0.0000000 | -1.9099489 | 0.0000000 | 0.0000000 | 0.0000000 | 0.0000000 | 0.0000000 | -0.0769309 | 0.0000000 | -1.3844696 | 0.0000000 | -1.5610837 | 0.0000000 | 0.0000000 | 14 | 4 | 258.0677 | 207.21613 |
| 1.3083068 | -0.4639307 | -2.5187485 | -2.7453416 | -16.6244212 | -2.7195816 | -2.0358444 | 0.0000000 | 0.0000000 | 0.0000000 | 0.0000000 | 0.0000000 | -2.0048429 | 0.0000000 | 0.0000000 | 0.0000000 | 0.0000000 | -0.0905185 | -0.2064335 | -0.2168349 | -1.6428919 | 0.0000000 | -0.5686262 | 0.0000000 | 0.0000000 | 14 | 5 | 202.3150 | 151.72538 |
| 2.3396870 | 0.0000000 | -4.3368733 | -3.8026686 | -30.5567367 | 0.0000000 | -3.4991617 | 0.0000000 | 0.0000000 | 0.0000000 | -0.2885036 | 0.0000000 | -3.4619622 | 0.0000000 | 0.0000000 | 0.0000000 | 0.0000000 | 0.0000000 | -0.4886274 | 0.0000000 | -1.5204723 | -0.0907869 | -0.4291285 | 0.0000000 | 0.0000000 | 14 | 6 | 138.6918 | 88.33245 |
| 0.0000000 | -1.2232257 | -0.3230203 | -0.8345314 | -3.9003722 | 0.0000000 | -0.3100122 | 0.0000000 | 0.0000000 | -0.0170699 | 0.0000000 | 0.0000000 | -0.1262965 | 0.0000000 | 0.0000000 | 0.0000000 | 0.0000000 | 0.0000000 | -0.5913242 | -0.2423176 | -0.6016643 | 0.0000000 | -0.0958340 | -0.3283158 | 0.0000000 | 15 | 1 | 494.4216 | 440.03298 |
| 0.3865873 | -1.2818960 | -0.6921513 | -0.5267622 | -14.6830728 | -0.0413975 | -0.9136837 | -0.0000810 | 0.0000000 | 0.0000000 | 0.0000000 | -0.0009937 | -1.0030934 | -0.0009770 | -0.0007045 | 0.0000000 | -0.0000719 | -0.1255316 | -0.1360945 | -0.0011691 | -0.8627639 | 0.0000000 | -0.6515180 | -0.0005451 | 0.0000000 | 15 | 2 | 483.3422 | 430.44422 |
| 0.7750529 | -0.0681785 | -2.0362010 | -1.9937881 | -33.9651767 | 0.0000000 | -1.7727645 | 0.0000000 | 0.0000000 | 0.0000000 | 0.0000000 | 0.0000000 | -1.1996742 | 0.0000000 | 0.0000000 | 0.0000000 | 0.0000000 | 0.0000000 | -0.4507806 | -0.5349083 | -0.6572523 | 0.0000000 | -0.6761388 | 0.0000000 | 0.0000000 | 15 | 3 | 362.0390 | 310.39546 |
| 2.1056703 | -0.8007555 | -1.9506531 | -3.3815349 | -26.1257051 | 0.0000000 | -3.9380053 | 0.0000000 | 0.0000000 | 0.0000000 | 0.0000000 | 0.0000000 | -0.6262703 | 0.0000000 | 0.0000000 | 0.0000000 | 0.0000000 | -0.4121725 | 0.0000000 | -1.0327145 | -0.9984994 | 0.0000000 | -1.0427576 | -0.2821230 | 0.0000000 | 15 | 4 | 245.9549 | 194.96322 |
| 3.1925087 | 0.0000000 | -3.0809253 | -2.0672583 | -17.3098343 | 0.0000000 | -4.0479382 | 0.0000000 | 0.0000000 | 0.0000000 | 0.0000000 | 0.0000000 | 0.0000000 | 0.0000000 | 0.0000000 | 0.0000000 | -0.3000475 | -0.4332636 | 0.0000000 | -1.8192002 | -0.6416998 | -0.4461940 | -1.8599660 | 0.0000000 | 0.0000000 | 15 | 5 | 243.4632 | 192.87455 |
| 3.4317042 | 0.0000000 | -2.7627817 | -2.4780514 | -40.7949820 | 0.0000000 | -4.6707833 | 0.0000000 | 0.0000000 | 0.0000000 | 0.0000000 | 0.0000000 | -4.1580529 | 0.0000000 | 0.0000000 | 0.0000000 | -0.0523684 | 0.0000000 | 0.0000000 | -0.1876274 | -1.9355862 | 0.0000000 | -1.6339024 | 0.0000000 | 0.0000000 | 15 | 6 | 191.7648 | 141.36664 |
| 0.0000000 | -1.0064804 | -0.3448533 | -1.7770157 | 0.0000000 | -0.1284450 | -0.2998062 | 0.0000000 | 0.0000000 | -0.0491452 | 0.0000000 | 0.0000000 | -0.1523400 | 0.0000000 | 0.0000000 | 0.0000000 | 0.0000000 | -0.0490282 | -0.8344350 | -0.5354269 | -0.3380199 | 0.0000000 | -0.1164691 | -0.3374949 | 0.0000000 | 16 | 1 | 450.3904 | 395.88673 |
| 0.2970356 | -1.2939910 | -0.4338154 | -0.9519602 | -13.9750859 | 0.0000000 | -1.1835773 | 0.0000000 | 0.0000000 | 0.0000000 | 0.0000000 | 0.0000000 | -0.5924118 | 0.0000000 | 0.0000000 | 0.0000000 | 0.0000000 | -0.1467736 | -0.1534436 | -0.1330625 | -0.9025526 | 0.0000000 | -0.5021891 | 0.0000000 | 0.0000000 | 16 | 2 | 442.4111 | 389.27853 |
| 0.4084256 | -0.9850584 | -0.6519650 | -1.3503225 | -16.3587393 | 0.0000000 | -1.6710101 | 0.0000000 | 0.0000000 | 0.0000000 | 0.0000000 | 0.0000000 | -1.3441571 | 0.0000000 | 0.0000000 | 0.0000000 | -0.0437562 | 0.0000000 | -0.0843991 | -0.0540769 | -1.5617351 | 0.0000000 | -0.6249192 | 0.0000000 | 0.0000000 | 16 | 3 | 374.4401 | 322.65932 |
| 2.0767399 | 0.0000000 | -2.2572751 | -1.9238851 | -16.4665880 | 0.0000000 | -2.7956687 | 0.0000000 | 0.0000000 | 0.0000000 | 0.0000000 | 0.0000000 | -1.2143725 | 0.0000000 | 0.0000000 | 0.0000000 | -0.0523314 | 0.0000000 | -0.1775795 | 0.0000000 | -2.3099687 | 0.0000000 | -1.1536795 | 0.0000000 | 0.0000000 | 16 | 4 | 299.9517 | 248.84667 |
| 2.2502264 | 0.0000000 | -2.5928660 | -3.4752922 | -26.5937758 | 0.0000000 | -3.4504675 | 0.0000000 | 0.0000000 | 0.0000000 | 0.0000000 | 0.0000000 | -3.0490432 | 0.0000000 | 0.0000000 | 0.0000000 | 0.0000000 | 0.0000000 | 0.0000000 | 0.0000000 | -2.1411816 | 0.0000000 | -0.9048856 | 0.0000000 | 0.0000000 | 16 | 5 | 221.6006 | 170.91712 |
| 4.1723922 | 0.0000000 | -3.2643884 | -7.3700606 | -1.2192699 | 0.0000000 | -1.6372524 | -0.3483073 | 0.0000000 | 0.0000000 | 0.0000000 | 0.0000000 | -0.5845863 | 0.0000000 | 0.0000000 | 0.0000000 | 0.0000000 | 0.0000000 | 0.0000000 | 0.0000000 | -4.3629267 | 0.0000000 | -2.3134426 | 0.0000000 | 0.0000000 | 16 | 6 | 151.5852 | 101.21591 |
| 0.0000000 | -1.0486326 | -0.3254198 | -0.6226726 | -10.5321868 | 0.0000000 | -0.3698110 | 0.0000000 | -0.0505797 | 0.0000000 | 0.0000000 | 0.0000000 | -0.1980874 | 0.0000000 | 0.0000000 | 0.0000000 | 0.0000000 | -0.0515921 | -0.4267548 | 0.0000000 | -0.6147091 | 0.0000000 | -0.0744986 | -0.5300550 | 0.0000000 | 17 | 1 | 449.0318 | 394.50350 |
| 0.2182007 | -0.8456684 | -0.3159611 | -1.2930078 | -13.4867689 | -0.0014376 | -1.0563866 | 0.0000000 | 0.0000000 | 0.0000000 | 0.0000000 | 0.0000000 | -0.6114747 | 0.0000000 | 0.0000000 | 0.0000000 | 0.0000000 | 0.0000000 | -0.5495003 | -0.2433953 | -0.9050275 | 0.0000000 | -0.4316208 | -0.2160976 | 0.0000000 | 17 | 2 | 438.3862 | 385.53324 |
| 1.3388923 | -0.8535949 | -1.1988152 | -1.9380152 | -38.9757948 | 0.0000000 | -1.6125928 | 0.0000000 | 0.0000000 | 0.0000000 | 0.0000000 | 0.0000000 | -1.2671166 | 0.0000000 | 0.0000000 | 0.0000000 | 0.0000000 | -0.1179635 | -0.1041890 | -0.5501738 | -1.1697154 | 0.0000000 | -1.3360652 | 0.0000000 | 0.0000000 | 17 | 3 | 342.1112 | 290.31964 |
| 2.2103964 | -0.1794512 | -2.2798423 | -2.2317880 | -10.3072103 | 0.0000000 | -2.2005343 | 0.0000000 | 0.0000000 | 0.0000000 | 0.0000000 | 0.0000000 | -1.1621348 | 0.0000000 | 0.0000000 | 0.0000000 | 0.0000000 | 0.0000000 | 0.0000000 | -0.9104632 | -2.1684040 | 0.0000000 | -1.5416999 | -0.0427791 | 0.0000000 | 17 | 4 | 245.2274 | 194.24951 |
| 3.4846786 | 0.0000000 | -4.4336668 | -0.7915382 | -29.4663629 | 0.0000000 | -3.6078155 | 0.0000000 | 0.0000000 | 0.0000000 | 0.0000000 | 0.0000000 | -1.9393701 | 0.0000000 | 0.0000000 | 0.0000000 | -0.4292085 | 0.0000000 | -0.8447047 | -0.0970312 | -1.9580324 | 0.0000000 | -1.6753059 | 0.0000000 | 0.0000000 | 17 | 5 | 186.1496 | 135.56072 |
| 4.6316926 | 0.0000000 | -2.3594336 | -5.3594360 | -13.3697932 | 0.0000000 | -5.0971465 | 0.0000000 | 0.0000000 | 0.0000000 | 0.0000000 | 0.0000000 | -1.0143547 | 0.0000000 | 0.0000000 | 0.0000000 | -0.4323015 | 0.0000000 | 0.0000000 | 0.0000000 | -3.5026268 | 0.0000000 | -2.1743327 | 0.0000000 | 0.0000000 | 17 | 6 | 150.8147 | 100.47209 |
| 0.0000000 | -1.1676348 | -0.1300971 | -0.9605801 | -8.1657826 | 0.0000000 | -0.5037849 | 0.0000000 | 0.0000000 | 0.0000000 | 0.0000000 | 0.0000000 | -0.5118960 | 0.0000000 | 0.0000000 | 0.0000000 | 0.0000000 | -0.0469420 | -0.3867100 | 0.0000000 | -0.8508217 | 0.0000000 | -0.0542238 | -0.4463094 | 0.0000000 | 18 | 1 | 466.7916 | 412.51162 |
| 0.2607870 | -0.3767210 | -1.2718547 | -1.0940271 | -17.1553515 | -0.0000026 | -0.8704043 | 0.0000000 | 0.0000000 | 0.0000000 | 0.0000000 | 0.0000000 | -0.7367919 | 0.0000000 | 0.0000000 | 0.0000000 | -0.0105944 | 0.0000000 | -0.4487772 | 0.0000000 | -0.8521935 | 0.0000000 | -0.3995822 | -0.1281579 | 0.0000000 | 18 | 2 | 399.1894 | 346.22557 |
| 0.9196183 | -0.1205469 | -1.6560296 | -1.4182601 | -13.3813827 | 0.0000000 | -1.2831629 | 0.0000000 | 0.0000000 | 0.0000000 | 0.0000000 | 0.0000000 | -0.6191110 | 0.0000000 | 0.0000000 | 0.0000000 | 0.0000000 | 0.0000000 | 0.0000000 | -0.1147144 | -1.4275487 | 0.0000000 | -1.0441077 | 0.0000000 | 0.0000000 | 18 | 3 | 380.0334 | 328.23036 |
| 1.2660868 | 0.0000000 | -2.8413234 | -1.7291739 | -19.3065165 | 0.0000000 | -3.1217181 | 0.0000000 | 0.0000000 | 0.0000000 | 0.0000000 | 0.0000000 | -1.1886527 | 0.0000000 | 0.0000000 | 0.0000000 | 0.0000000 | 0.0000000 | -0.3293205 | -0.7526940 | -0.3652943 | 0.0000000 | -0.8441131 | 0.0000000 | 0.0000000 | 18 | 4 | 297.3921 | 246.37050 |
| 2.1907322 | -0.1497888 | -3.6113570 | -2.1881969 | -27.2085055 | 0.0000000 | -2.2265867 | 0.0000000 | 0.0000000 | 0.0000000 | 0.0000000 | 0.0000000 | 0.0000000 | 0.0000000 | 0.0000000 | 0.0000000 | 0.0000000 | 0.0000000 | 0.0000000 | -2.1675605 | -0.5035473 | 0.0000000 | -1.8035515 | 0.0000000 | 0.0000000 | 18 | 5 | 211.4296 | 160.83516 |
| 2.1037026 | 0.0000000 | -1.8303582 | -4.0500387 | -54.4457856 | 0.0000000 | -3.9170724 | 0.0000000 | 0.0000000 | 0.0000000 | 0.0000000 | 0.0000000 | -2.8534914 | 0.0000000 | 0.0000000 | 0.0000000 | 0.0000000 | -0.0379903 | -1.1520577 | -0.8938811 | -0.7406895 | 0.0000000 | -1.3358182 | 0.0000000 | 0.0000000 | 18 | 6 | 148.1041 | 97.78000 |
| 0.0104031 | -1.7337282 | -0.0551680 | -0.9882523 | -7.1332171 | 0.0000000 | -0.4406494 | 0.0000000 | 0.0000000 | 0.0000000 | 0.0000000 | 0.0000000 | -0.5410026 | 0.0000000 | 0.0000000 | 0.0000000 | 0.0000000 | -0.1105871 | -0.2896343 | -0.0407576 | -0.6686853 | 0.0000000 | -0.1537635 | -0.2944317 | 0.0000000 | 19 | 1 | 453.3982 | 399.00776 |
| 0.3731252 | -0.6691902 | -1.1397328 | -0.7594410 | -22.9854206 | 0.0000000 | -0.9661792 | 0.0000000 | 0.0000000 | 0.0000000 | 0.0000000 | 0.0000000 | -0.5379512 | 0.0000000 | 0.0000000 | 0.0000000 | 0.0000000 | 0.0000000 | -0.5331932 | -0.0994534 | -0.7361485 | 0.0000000 | -0.5961975 | 0.0000000 | 0.0000000 | 19 | 2 | 426.6557 | 373.71815 |
| 0.9247744 | -0.5911353 | -1.3436276 | -2.2499558 | -12.9810810 | 0.0000000 | -1.7245853 | 0.0000000 | 0.0000000 | 0.0000000 | 0.0000000 | 0.0000000 | -1.2668773 | 0.0000000 | 0.0000000 | 0.0000000 | 0.0000000 | 0.0000000 | -0.1332370 | 0.0000000 | -1.5647070 | 0.0000000 | -0.7691685 | 0.0000000 | 0.0000000 | 19 | 3 | 405.9462 | 354.32396 |
| 1.9716726 | -0.5240880 | -2.5081370 | -1.5782259 | -21.7969977 | -0.0000032 | -3.1063089 | 0.0000000 | 0.0000000 | 0.0000000 | 0.0000000 | 0.0000000 | -1.1916554 | 0.0000000 | 0.0000000 | 0.0000000 | 0.0000000 | 0.0000000 | 0.0000000 | 0.0000000 | -2.2037743 | 0.0000000 | -0.9092753 | 0.0000000 | 0.0000000 | 19 | 4 | 298.2108 | 247.15928 |
| 1.1523218 | -0.4008439 | -1.1134590 | -2.9712321 | -1.1358931 | -5.0998169 | -2.8550200 | 0.0000000 | 0.0000000 | 0.0000000 | 0.0000000 | 0.0000000 | -2.3240834 | 0.0000000 | 0.0000000 | 0.0000000 | -0.1823999 | -0.5599543 | 0.0000000 | 0.0000000 | -1.5519133 | 0.0000000 | -0.3679347 | 0.0000000 | 0.0000000 | 19 | 5 | 199.7888 | 149.28118 |
| 4.0110556 | 0.0000000 | -2.6462967 | -4.1158091 | -26.6925307 | 0.0000000 | -5.3313878 | 0.0000000 | 0.0000000 | -0.7955940 | -0.3731544 | -1.8506899 | -1.1753389 | 0.0000000 | 0.0000000 | 0.0000000 | 0.0000000 | 0.0000000 | -0.4290043 | 0.0000000 | -1.0229741 | 0.0000000 | -1.6260507 | 0.0000000 | 0.0000000 | 19 | 6 | 151.4134 | 100.99603 |
| 0.0000000 | -1.4476064 | -0.0568320 | -1.3694619 | -6.9657069 | 0.0000000 | -0.4048520 | -0.0345190 | 0.0000000 | 0.0000000 | 0.0000000 | 0.0000000 | 0.0000000 | 0.0000000 | 0.0000000 | 0.0000000 | 0.0000000 | -0.1241087 | -0.4430475 | -0.2401578 | -0.5235851 | 0.0000000 | -0.2030969 | -0.2865255 | 0.0000000 | 20 | 1 | 458.5129 | 404.05596 |
| 0.4286742 | -0.9838033 | -0.5510521 | -1.0745093 | -14.6886536 | 0.0000000 | -0.8333187 | 0.0000000 | 0.0000000 | 0.0000000 | 0.0000000 | 0.0000000 | -0.8063550 | 0.0000000 | 0.0000000 | 0.0000000 | 0.0000000 | -0.0308077 | -0.2999803 | 0.0000000 | -1.0797779 | 0.0000000 | -0.5951373 | -0.1932615 | 0.0000000 | 20 | 2 | 421.0608 | 367.94089 |
| 0.9456830 | -0.2309359 | -1.6237811 | -1.1949764 | -16.6527408 | 0.0000000 | -1.6737618 | 0.0000000 | 0.0000000 | 0.0000000 | 0.0000000 | 0.0000000 | -1.1218299 | 0.0000000 | 0.0000000 | 0.0000000 | -0.0826715 | 0.0000000 | 0.0000000 | 0.0000000 | -1.6955807 | 0.0000000 | -0.9177184 | 0.0000000 | 0.0000000 | 20 | 3 | 371.9613 | 320.21325 |
| 3.0042202 | 0.0000000 | -2.5748385 | -1.0532553 | -27.8469780 | 0.0000000 | -2.6409432 | 0.0000000 | 0.0000000 | 0.0000000 | 0.0000000 | 0.0000000 | 0.0000000 | 0.0000000 | 0.0000000 | 0.0000000 | -0.1644553 | 0.0000000 | 0.0000000 | 0.0000000 | -3.6054390 | 0.0000000 | -1.7624063 | 0.0000000 | 0.0000000 | 20 | 4 | 266.4772 | 215.55687 |
| 1.5193640 | 0.0000000 | -3.4434772 | -3.5568418 | -34.8013798 | 0.0000000 | -2.0860292 | 0.0000000 | 0.0000000 | -0.2836221 | 0.0000000 | 0.0000000 | -2.1753632 | 0.0000000 | 0.0000000 | 0.0000000 | 0.0000000 | 0.0000000 | 0.0000000 | -0.4809303 | -2.2747838 | 0.0000000 | -0.4368827 | 0.0000000 | 0.0000000 | 20 | 5 | 192.5383 | 142.03523 |
| 2.0981139 | -0.3253759 | -3.0682236 | -5.8166148 | -25.9487575 | 0.0000000 | -3.0871280 | 0.0000000 | 0.0000000 | 0.0000000 | 0.0000000 | 0.0000000 | 0.0000000 | 0.0000000 | 0.0000000 | 0.0000000 | -0.4254593 | 0.0000000 | 0.0000000 | -1.8641097 | -2.7027628 | 0.0000000 | -0.0531718 | 0.0000000 | 0.0000000 | 20 | 6 | 149.4008 | 99.08313 |
| 0.0000000 | -1.1502927 | -0.3757929 | -1.2931595 | -0.2248738 | 0.0000000 | -0.4057453 | 0.0000000 | -0.0456302 | -0.0330395 | 0.0000000 | 0.0000000 | -0.8606871 | 0.0000000 | 0.0000000 | 0.0000000 | 0.0000000 | 0.0000000 | -0.3271279 | -0.2028624 | -0.3621636 | 0.0000000 | -0.1850283 | -0.3261909 | 0.0000000 | 21 | 1 | 493.0184 | 438.52371 |
| 0.2015215 | -1.2351556 | -0.0580671 | -1.4496900 | -20.7739597 | 0.0000000 | -1.1145919 | 0.0000000 | 0.0000000 | 0.0000000 | 0.0000000 | 0.0000000 | -0.4977998 | 0.0000000 | 0.0000000 | 0.0000000 | 0.0000000 | -0.0700218 | -0.1032743 | -0.0486584 | -1.0171897 | 0.0000000 | -0.2931280 | -0.4424250 | 0.0000000 | 21 | 2 | 449.3833 | 396.44322 |
| 1.3911944 | -0.2146027 | -1.8604249 | -2.4842336 | -13.1357260 | 0.0000000 | -1.4742448 | 0.0000000 | 0.0000000 | 0.0000000 | 0.0000000 | 0.0000000 | -0.9493661 | 0.0000000 | 0.0000000 | 0.0000000 | -0.2413293 | 0.0000000 | -0.2123994 | 0.0000000 | -1.5301219 | 0.0000000 | -1.1421004 | 0.0000000 | -0.2778459 | 21 | 3 | 359.1372 | 307.34319 |
| 1.4385568 | 0.0000000 | -2.5037083 | -3.1085626 | -11.4228355 | 0.0000000 | -3.2100584 | 0.0000000 | 0.0000000 | 0.0000000 | 0.0000000 | 0.0000000 | -1.1960393 | 0.0000000 | 0.0000000 | 0.0000000 | 0.0000000 | 0.0000000 | -0.8145775 | 0.0000000 | -1.8371497 | 0.0000000 | -0.3877942 | 0.0000000 | 0.0000000 | 21 | 4 | 251.1900 | 200.19488 |
| 1.6409092 | -0.2080742 | -3.0777557 | -3.3977485 | -32.0553453 | 0.0000000 | -3.1720563 | 0.0000000 | 0.0000000 | -0.0818034 | 0.0000000 | 0.0000000 | -0.9634125 | 0.0000000 | 0.0000000 | 0.0000000 | -0.7900031 | 0.0000000 | -0.5994162 | 0.0000000 | -1.8873247 | 0.0000000 | 0.0000000 | 0.0000000 | -0.0188881 | 21 | 5 | 226.8820 | 176.27786 |
| 3.9575724 | -0.2872784 | -0.9293156 | -3.1203829 | -34.0105025 | 0.0000000 | -2.4027017 | -0.2165991 | 0.0000000 | 0.0000000 | 0.0000000 | 0.0000000 | -1.5058353 | 0.0000000 | 0.0000000 | 0.0000000 | 0.0000000 | 0.0000000 | 0.0000000 | 0.0000000 | -5.1730030 | 0.0000000 | -2.2559765 | -0.0162307 | -7.9604815 | 21 | 6 | 156.6125 | 106.25093 |
| 0.0000000 | -1.1398300 | -0.2796200 | -1.0325079 | -0.2314182 | -1.1627790 | -0.1771106 | 0.0000000 | -0.0765538 | 0.0000000 | 0.0000000 | 0.0000000 | -0.3874529 | 0.0000000 | 0.0000000 | 0.0000000 | 0.0000000 | 0.0000000 | -0.4273014 | -0.2306013 | -0.5968415 | 0.0000000 | -0.0143519 | -0.5242771 | 0.0000000 | 22 | 1 | 476.2805 | 421.95040 |
| 0.3219967 | -1.1799448 | -0.3687311 | -1.6279946 | -8.4617560 | 0.0000000 | -1.0683662 | 0.0000000 | 0.0000000 | 0.0000000 | 0.0000000 | 0.0000000 | -0.4813585 | 0.0000000 | 0.0000000 | 0.0000000 | -0.0410204 | 0.0000000 | -0.3355510 | 0.0000000 | -1.2539854 | 0.0000000 | -0.3279636 | -0.3296231 | 0.0000000 | 22 | 2 | 443.8028 | 390.97885 |
| 0.8756730 | 0.0000000 | -2.1958789 | -1.2924074 | -18.0989529 | 0.0000000 | -1.6410144 | 0.0000000 | 0.0000000 | -0.0117306 | 0.0000000 | 0.0000000 | -0.8948917 | 0.0000000 | 0.0000000 | 0.0000000 | -0.2286733 | -0.3214638 | 0.0000000 | -0.1542446 | -1.2396046 | 0.0000000 | -0.5769224 | 0.0000000 | 0.0000000 | 22 | 3 | 350.8135 | 299.11945 |
| 1.8748065 | 0.0000000 | -3.1182001 | -2.2273239 | -20.2589434 | 0.0000000 | -2.3600104 | 0.0000000 | 0.0000000 | 0.0000000 | -0.0827017 | -0.1535919 | 0.0000000 | 0.0000000 | 0.0000000 | 0.0000000 | 0.0000000 | -0.7062815 | 0.0000000 | 0.0000000 | -1.9342622 | 0.0000000 | -0.6721090 | 0.0000000 | 0.0000000 | 22 | 4 | 283.4879 | 232.42142 |
| 2.0861124 | 0.0000000 | -2.0955765 | -7.1073179 | -13.5057296 | 0.0000000 | -3.0761812 | 0.0000000 | 0.0000000 | 0.0000000 | 0.0000000 | 0.0000000 | -1.7105567 | 0.0000000 | 0.0000000 | 0.0000000 | 0.0000000 | 0.0000000 | -1.0181228 | -2.4775683 | -0.4717811 | -0.1688302 | -1.1690817 | 0.0000000 | 0.0000000 | 22 | 5 | 164.2910 | 113.67315 |
| 2.6999521 | 0.0000000 | -2.6676643 | -5.2481198 | 0.0000000 | -8.8229483 | -3.2311850 | 0.0000000 | 0.0000000 | 0.0000000 | 0.0000000 | 0.0000000 | -0.2942464 | 0.0000000 | 0.0000000 | 0.0000000 | -0.6853623 | 0.0000000 | -0.3995546 | -2.6565767 | 0.0000000 | 0.0000000 | -1.1433601 | 0.0000000 | -1.5872934 | 22 | 6 | 145.6635 | 95.31796 |
| 0.0000000 | -1.4216979 | -0.0923972 | -1.6605405 | -7.2694450 | 0.0000000 | -0.2706324 | 0.0000000 | 0.0000000 | 0.0000000 | 0.0000000 | 0.0000000 | 0.0000000 | 0.0000000 | 0.0000000 | 0.0000000 | 0.0000000 | -0.2133085 | -0.4117608 | -0.4808520 | -0.4427472 | 0.0000000 | -0.1334155 | -0.4152865 | 0.0000000 | 23 | 1 | 455.1651 | 400.71873 |
| 0.2099403 | -1.1197327 | -0.5058262 | -1.1661902 | -18.0232914 | 0.0000000 | -0.8927601 | 0.0000000 | 0.0000000 | 0.0000000 | 0.0000000 | 0.0000000 | -1.0323841 | 0.0000000 | 0.0000000 | 0.0000000 | 0.0000000 | -0.3501375 | -0.0502906 | 0.0000000 | -0.9410928 | 0.0000000 | -0.3143353 | -0.0670086 | 0.0000000 | 23 | 2 | 475.5884 | 422.82447 |
| 0.8068365 | -1.1320342 | -0.9362802 | -3.1359818 | -12.1322511 | 0.0000000 | -1.8702050 | 0.0000000 | 0.0000000 | 0.0000000 | 0.0000000 | 0.0000000 | -0.6335210 | 0.0000000 | 0.0000000 | 0.0000000 | -0.0131691 | 0.0000000 | -0.2256641 | -0.3993741 | -1.0685823 | 0.0000000 | -0.8570178 | 0.0000000 | 0.0000000 | 23 | 3 | 352.4342 | 300.67946 |
| 2.5089148 | -0.0857294 | -1.6710174 | -4.6471612 | -15.8496898 | 0.0000000 | -3.5819917 | 0.0000000 | 0.0000000 | 0.0000000 | 0.0000000 | 0.0000000 | -0.9555740 | 0.0000000 | 0.0000000 | 0.0000000 | 0.0000000 | 0.0000000 | -0.7308614 | 0.0000000 | -2.1435858 | 0.0000000 | -1.4905430 | 0.0000000 | 0.0000000 | 23 | 4 | 249.2033 | 198.28672 |
| 2.4484149 | 0.0000000 | -4.2946883 | -2.5445363 | -9.0836442 | 0.0000000 | -5.1122594 | 0.0000000 | 0.0000000 | 0.0000000 | 0.0000000 | 0.0000000 | -1.5208169 | 0.0000000 | 0.0000000 | 0.0000000 | 0.0000000 | 0.0000000 | 0.0000000 | -0.4829851 | -2.4634944 | 0.0000000 | 0.0000000 | 0.0000000 | -0.4964053 | 23 | 5 | 211.0695 | 160.48730 |
| 0.5499953 | 0.0000000 | -4.4685561 | -4.6869462 | -39.8828836 | 0.0000000 | -2.6900396 | 0.0000000 | 0.0000000 | 0.0000000 | 0.0000000 | 0.0000000 | -1.8905252 | 0.0000000 | 0.0000000 | 0.0000000 | -0.0964165 | 0.0000000 | -0.1188051 | 0.0000000 | -0.0705052 | 0.0000000 | 0.0000000 | 0.0000000 | 0.0000000 | 23 | 6 | 153.4898 | 103.12087 |
| 0.0043416 | -0.8298634 | -0.6587353 | -0.8421258 | -10.8734062 | 0.0000000 | -0.6105238 | 0.0000000 | 0.0000000 | 0.0000000 | 0.0000000 | 0.0000000 | -0.2981252 | -0.0947715 | 0.0000000 | 0.0000000 | 0.0000000 | 0.0000000 | -0.7347315 | -0.0752205 | -0.3187552 | 0.0000000 | -0.1719036 | -0.2493753 | 0.0000000 | 24 | 1 | 463.7905 | 409.40030 |
| 0.4026289 | -0.2104024 | -1.2977867 | -0.9211407 | -8.6282762 | 0.0000000 | -1.0562368 | 0.0000000 | 0.0000000 | 0.0000000 | 0.0000000 | 0.0000000 | -0.4620551 | 0.0000000 | 0.0000000 | 0.0000000 | 0.0000000 | 0.0000000 | -0.7020488 | 0.0000000 | -1.2834687 | 0.0000000 | -0.2513404 | 0.0000000 | 0.0000000 | 24 | 2 | 446.1601 | 393.17826 |
| 1.4353856 | -0.1569351 | -2.3885376 | -1.0721799 | -20.6357241 | 0.0000000 | -1.5029719 | 0.0000000 | 0.0000000 | 0.0000000 | 0.0000000 | 0.0000000 | -0.4380104 | 0.0000000 | 0.0000000 | 0.0000000 | 0.0000000 | 0.0000000 | -0.3930477 | 0.0000000 | -1.8090581 | 0.0000000 | -1.0374373 | 0.0000000 | 0.0000000 | 24 | 3 | 364.6891 | 312.82756 |
| 1.8104144 | 0.0000000 | -2.5229899 | -3.3913845 | -15.9664750 | 0.0000000 | -2.7994235 | 0.0000000 | 0.0000000 | -0.0524967 | 0.0000000 | 0.0000000 | -1.6515916 | 0.0000000 | 0.0000000 | 0.0000000 | 0.0000000 | -0.2441098 | -0.1219221 | 0.0000000 | -0.9519385 | 0.0000000 | -1.1876640 | 0.0000000 | 0.0000000 | 24 | 4 | 266.8426 | 215.69115 |
| 1.9751231 | 0.0000000 | -3.1752121 | -3.3448501 | -1.1284303 | -4.0904647 | -3.5605988 | 0.0000000 | 0.0000000 | 0.0000000 | -0.2926886 | 0.0000000 | -2.3852626 | 0.0000000 | 0.0000000 | 0.0000000 | 0.0000000 | 0.0000000 | 0.0000000 | -0.9082326 | -1.0185490 | -0.2852690 | -0.2014781 | -0.1774551 | -0.7089676 | 24 | 5 | 192.7415 | 142.08176 |
| 2.5753210 | 0.0000000 | -3.0254350 | -4.2693009 | -12.6680083 | 0.0000000 | -2.6767739 | 0.0000000 | 0.0000000 | 0.0000000 | 0.0000000 | 0.0000000 | -3.2702752 | 0.0000000 | 0.0000000 | 0.0000000 | -0.5008241 | 0.0000000 | 0.0000000 | 0.0000000 | -1.9231293 | 0.0000000 | -1.7452427 | 0.0000000 | 0.0000000 | 24 | 6 | 153.4026 | 103.10380 |
| 0.0319259 | -1.2357372 | -0.0910778 | -1.1225527 | -10.0205156 | 0.0000000 | -0.4532377 | 0.0000000 | 0.0000000 | 0.0000000 | 0.0000000 | 0.0000000 | -0.5938329 | 0.0000000 | 0.0000000 | 0.0000000 | 0.0000000 | -0.2191225 | -0.4663162 | 0.0000000 | -0.6136863 | 0.0000000 | -0.0773301 | -0.3358530 | 0.0000000 | 25 | 1 | 483.3615 | 429.04530 |
| 0.2432697 | -0.7958099 | -0.4507161 | -1.2605295 | -12.7335231 | 0.0000000 | -1.0508384 | -0.0365018 | 0.0000000 | 0.0000000 | 0.0000000 | 0.0000000 | -0.7407297 | 0.0000000 | 0.0000000 | 0.0000000 | 0.0000000 | -0.2352253 | 0.0000000 | 0.0000000 | -0.9086494 | 0.0000000 | -0.2453019 | -0.5539555 | 0.0000000 | 25 | 2 | 485.2256 | 432.45473 |
| 0.6965131 | -1.3450746 | -0.8105845 | -2.8942546 | -7.6188984 | 0.0000000 | -1.9464962 | 0.0000000 | 0.0000000 | 0.0000000 | 0.0000000 | 0.0000000 | -0.8279558 | 0.0000000 | 0.0000000 | 0.0000000 | 0.0000000 | 0.0000000 | -0.3908538 | -0.5796024 | -1.8472939 | 0.0000000 | -0.3426090 | 0.0000000 | 0.0000000 | 25 | 3 | 354.3280 | 302.76751 |
| 1.8001402 | 0.0000000 | -2.6418025 | -1.9816963 | -29.3370151 | -1.2821576 | -2.9267829 | 0.0000000 | 0.0000000 | 0.0000000 | 0.0000000 | 0.0000000 | -1.9358625 | 0.0000000 | 0.0000000 | 0.0000000 | 0.0000000 | 0.0000000 | 0.0000000 | -0.2073117 | -1.7805853 | 0.0000000 | -0.6732795 | 0.0000000 | -1.0682756 | 25 | 4 | 291.4925 | 240.58896 |
| 2.3008095 | -1.5737223 | -0.9412634 | -9.2754021 | -16.7510266 | 0.0000000 | -1.4243458 | 0.0000000 | 0.0000000 | -0.1944766 | 0.0000000 | 0.0000000 | -0.7512641 | 0.0000000 | 0.0000000 | 0.0000000 | 0.0000000 | -0.0472864 | 0.0000000 | -1.0722292 | -1.7550275 | 0.0000000 | -1.7728392 | -0.0556977 | -3.4797458 | 25 | 5 | 185.3289 | 134.68862 |
| 3.8188816 | 0.0000000 | -5.0753762 | -3.6427571 | -51.7657703 | -0.6567356 | -2.7555835 | 0.0000000 | 0.0000000 | 0.0000000 | 0.0000000 | 0.0000000 | -5.0852061 | 0.0000000 | 0.0000000 | 0.0000000 | 0.0000000 | -0.7210464 | 0.0000000 | -0.3829677 | -0.0770957 | 0.0000000 | -2.3197454 | 0.0000000 | 0.0000000 | 25 | 6 | 148.8548 | 98.51678 |
| 0.0000000 | -1.0559404 | -0.4284036 | -0.9446285 | -8.0677447 | 0.0000000 | -0.3779422 | 0.0000000 | 0.0000000 | -0.0897370 | 0.0000000 | 0.0000000 | -0.4325971 | 0.0000000 | 0.0000000 | 0.0000000 | 0.0000000 | -0.0991288 | -0.3278291 | 0.0000000 | -0.4559068 | 0.0000000 | -0.0908489 | -0.4659165 | 0.0000000 | 26 | 1 | 473.0859 | 418.74906 |
| 0.2455017 | -0.9180317 | -0.5253669 | -1.4325906 | -10.8151106 | 0.0000000 | -0.8347228 | 0.0000000 | 0.0000000 | 0.0000000 | 0.0000000 | 0.0000000 | -0.8956719 | 0.0000000 | 0.0000000 | 0.0000000 | 0.0000000 | -0.0064332 | -0.2298872 | -0.0934755 | -0.8952388 | 0.0000000 | -0.3753890 | -0.2750189 | 0.0000000 | 26 | 2 | 469.0844 | 416.24102 |
| 0.5666624 | -1.3067252 | -0.7422386 | -2.1609273 | -30.7686219 | 0.0000000 | -1.5115790 | 0.0000000 | 0.0000000 | 0.0000000 | 0.0000000 | 0.0000000 | 0.0000000 | 0.0000000 | 0.0000000 | 0.0000000 | 0.0000000 | 0.0000000 | -0.2650559 | -1.0245107 | -1.6204596 | 0.0000000 | 0.0000000 | -0.7293472 | 0.0000000 | 26 | 3 | 366.0573 | 314.29857 |
| 1.4620667 | -0.2710433 | -0.9417889 | -2.4989376 | -56.8547680 | 0.0000000 | -1.9388122 | 0.0000000 | 0.0000000 | 0.0000000 | 0.0000000 | 0.0000000 | -2.8628409 | 0.0000000 | 0.0000000 | 0.0000000 | 0.0000000 | 0.0000000 | 0.0000000 | 0.0000000 | -1.8889025 | 0.0000000 | -1.3503130 | 0.0000000 | 0.0000000 | 26 | 4 | 274.3271 | 223.31994 |
| 1.8575095 | 0.0000000 | -2.3032567 | -2.1659902 | -9.9586288 | 0.0000000 | -4.4949775 | 0.0000000 | 0.0000000 | 0.0000000 | 0.0000000 | 0.0000000 | -1.1696862 | 0.0000000 | 0.0000000 | 0.0000000 | 0.0000000 | 0.0000000 | 0.0000000 | -0.3981388 | -2.2024303 | 0.0000000 | -0.6460956 | 0.0000000 | 0.0000000 | 26 | 5 | 249.2178 | 198.60204 |
| 2.8042166 | 0.0000000 | -3.6624884 | -3.6301160 | -2.2150833 | -4.8946813 | -2.7506066 | 0.0000000 | 0.0000000 | 0.0000000 | 0.0000000 | 0.0000000 | -1.2223454 | 0.0000000 | 0.0000000 | 0.0000000 | 0.0000000 | -1.5240888 | -0.2880039 | 0.0000000 | -2.1525388 | 0.0000000 | 0.0000000 | -0.0777404 | 0.0000000 | 26 | 6 | 158.9831 | 108.61791 |
| 0.0000000 | -1.2630966 | -0.0781081 | -1.1914733 | -0.8542802 | 0.0000000 | -0.3914581 | -0.0120830 | 0.0000000 | 0.0000000 | 0.0000000 | 0.0000000 | -0.5305407 | 0.0000000 | 0.0000000 | 0.0000000 | 0.0000000 | -0.1094420 | -0.2743719 | -0.1669647 | -0.5982175 | 0.0000000 | -0.1013789 | -0.5349190 | 0.0000000 | 27 | 1 | 466.9507 | 412.54779 |
| 0.4013158 | -1.1784167 | -0.1813365 | -1.5681674 | -27.2097995 | 0.0000000 | -0.9533700 | 0.0000000 | 0.0000000 | 0.0000000 | 0.0000000 | 0.0000000 | -0.9014934 | 0.0000000 | 0.0000000 | 0.0000000 | -0.0695237 | 0.0000000 | -0.1786108 | 0.0000000 | -0.8844439 | 0.0000000 | -0.8450268 | 0.0000000 | 0.0000000 | 27 | 2 | 437.9352 | 385.07920 |
| 0.5248969 | -0.6642551 | -1.3882561 | -0.9524147 | -13.9226805 | 0.0000000 | -1.8749653 | 0.0000000 | 0.0000000 | 0.0000000 | 0.0000000 | 0.0000000 | -1.3219032 | 0.0000000 | 0.0000000 | 0.0000000 | 0.0000000 | 0.0000000 | -0.4208385 | -0.8291147 | -0.9802872 | 0.0000000 | -0.4360288 | 0.0000000 | 0.0000000 | 27 | 3 | 371.1407 | 319.22043 |
| 2.3747672 | -0.2913670 | -1.8677602 | -3.6357347 | -23.5867043 | 0.0000000 | -2.7149702 | 0.0000000 | 0.0000000 | -0.1889649 | -0.2001481 | 0.0000000 | -1.7399924 | 0.0000000 | 0.0000000 | 0.0000000 | 0.0000000 | 0.0000000 | 0.0000000 | -0.3593931 | -1.9097006 | 0.0000000 | -1.4213497 | 0.0000000 | 0.0000000 | 27 | 4 | 266.2598 | 215.07105 |
| 2.0148202 | 0.0000000 | -2.0180879 | -3.6320527 | -22.3418954 | 0.0000000 | -3.8431076 | 0.0000000 | 0.0000000 | 0.0000000 | 0.0000000 | 0.0000000 | -0.8827966 | 0.0000000 | 0.0000000 | 0.0000000 | 0.0000000 | 0.0000000 | -0.9945670 | -0.7772410 | -1.5454749 | 0.0000000 | -0.5588257 | 0.0000000 | 0.0000000 | 27 | 5 | 258.3688 | 207.69698 |
| 2.4769573 | 0.0000000 | -3.0551602 | -5.8682327 | -27.5606623 | 0.0000000 | -4.6961222 | 0.0000000 | 0.0000000 | 0.0000000 | 0.0000000 | 0.0000000 | -2.5600937 | 0.0000000 | 0.0000000 | 0.0000000 | 0.0000000 | 0.0000000 | -0.2310952 | 0.0000000 | -3.1436524 | 0.0000000 | 0.0000000 | 0.0000000 | -1.6894139 | 27 | 6 | 136.4014 | 86.12556 |
| 0.0000000 | -1.0542255 | -0.4717052 | -0.4308285 | -9.7633192 | 0.0000000 | -0.4975463 | -0.0411030 | 0.0000000 | 0.0000000 | 0.0000000 | 0.0000000 | -0.1264392 | -0.2632085 | 0.0000000 | 0.0000000 | -0.0733017 | 0.0000000 | -0.3635704 | -0.1987940 | -0.4777771 | 0.0000000 | -0.0548834 | -0.4839844 | 0.0000000 | 28 | 1 | 489.9379 | 435.54852 |
| 0.4119301 | -0.9452017 | -0.6964435 | -0.8441208 | -17.5903922 | 0.0000000 | -1.1084464 | 0.0000000 | 0.0000000 | 0.0000000 | 0.0000000 | 0.0000000 | -1.4852838 | 0.0000000 | 0.0000000 | 0.0000000 | 0.0000000 | -0.3464862 | -0.2040652 | 0.0000000 | -0.8772842 | 0.0000000 | -0.4465538 | 0.0000000 | 0.0000000 | 28 | 2 | 453.2225 | 400.24164 |
| 1.3375525 | -0.7804118 | -1.7655779 | -1.4574477 | -16.0717844 | 0.0000000 | -2.1394574 | 0.0000000 | 0.0000000 | 0.0000000 | 0.0000000 | 0.0000000 | -0.5299531 | 0.0000000 | 0.0000000 | 0.0000000 | 0.0000000 | 0.0000000 | -0.3328028 | 0.0000000 | -1.6511866 | 0.0000000 | -0.9920537 | 0.0000000 | 0.0000000 | 28 | 3 | 375.8928 | 324.14318 |
| 2.2435075 | 0.0000000 | -2.5956314 | -1.5645890 | -9.8875378 | 0.0000000 | -2.8997608 | 0.0000000 | 0.0000000 | -0.2994305 | 0.0000000 | 0.0000000 | -2.6824549 | 0.0000000 | 0.0000000 | 0.0000000 | -0.2629161 | -0.4406316 | -0.3831249 | 0.0000000 | -1.6992157 | 0.0000000 | -0.8736334 | 0.0000000 | 0.0000000 | 28 | 4 | 277.1872 | 226.14888 |
| 2.9086724 | -0.8460056 | -2.1057290 | -1.6679234 | -6.3568847 | -3.5847084 | -2.6277604 | -0.6295833 | 0.0000000 | 0.0000000 | 0.0000000 | 0.0000000 | -3.8965388 | 0.0000000 | 0.0000000 | 0.0000000 | -0.0876188 | -0.4033280 | 0.0000000 | 0.0000000 | -2.2526137 | 0.0000000 | -1.6671898 | 0.0000000 | 0.0000000 | 28 | 5 | 195.9039 | 145.36390 |
| 1.5946705 | 0.0000000 | -5.9948591 | -3.2785971 | -16.4548186 | 0.0000000 | -2.6243410 | -0.1761751 | 0.0000000 | 0.0000000 | 0.0000000 | 0.0000000 | -1.8732286 | 0.0000000 | 0.0000000 | 0.0000000 | -0.6125413 | 0.0000000 | 0.0000000 | -1.2858863 | -0.0402548 | 0.0000000 | 0.0000000 | -0.5021289 | -0.5970565 | 28 | 6 | 143.5100 | 93.25721 |
| 0.0000000 | -1.2973271 | -0.2863958 | -0.9848301 | -7.4952292 | 0.0000000 | -0.5473612 | 0.0000000 | 0.0000000 | -0.0689871 | 0.0000000 | 0.0000000 | -0.7151937 | 0.0000000 | 0.0000000 | 0.0000000 | -0.0441531 | -0.0424618 | -0.3054488 | 0.0000000 | -0.4369946 | 0.0000000 | -0.1398383 | -0.2911724 | 0.0000000 | 29 | 1 | 464.0599 | 409.49577 |
| 0.1643001 | -1.3135220 | -0.0942253 | -2.1596599 | -9.8049835 | 0.0000000 | -0.9581598 | 0.0000000 | 0.0000000 | 0.0000000 | 0.0000000 | 0.0000000 | -0.3340420 | 0.0000000 | 0.0000000 | 0.0000000 | 0.0000000 | -0.1399182 | -0.4841633 | -0.3859015 | -0.5554139 | 0.0000000 | -0.5566442 | 0.0000000 | 0.0000000 | 29 | 2 | 470.9620 | 418.16093 |
| 0.5763344 | -0.6948585 | -0.9199233 | -2.6989381 | -22.3673237 | 0.0000000 | -2.0002765 | 0.0000000 | 0.0000000 | 0.0000000 | 0.0000000 | 0.0000000 | -1.2270213 | 0.0000000 | 0.0000000 | 0.0000000 | 0.0000000 | 0.0000000 | -0.5457241 | -0.1795629 | -1.1574850 | 0.0000000 | -0.5146220 | 0.0000000 | 0.0000000 | 29 | 3 | 370.9794 | 319.22990 |
| 2.3231720 | 0.0000000 | -2.6969568 | -1.5378378 | -11.2573835 | 0.0000000 | -2.4535431 | 0.0000000 | 0.0000000 | 0.0000000 | 0.0000000 | 0.0000000 | -1.4064015 | 0.0000000 | 0.0000000 | 0.0000000 | 0.0000000 | -0.4757495 | -0.0225508 | 0.0000000 | -2.4201811 | 0.0000000 | -1.1651767 | 0.0000000 | 0.0000000 | 29 | 4 | 281.5287 | 230.49035 |
| 3.3483255 | -1.6155333 | -1.2596925 | -2.8016430 | -17.9834310 | 0.0000000 | -3.7018657 | -0.5195142 | 0.0000000 | 0.0000000 | 0.0000000 | 0.0000000 | -1.5215983 | 0.0000000 | 0.0000000 | 0.0000000 | -0.4468842 | 0.0000000 | 0.0000000 | -0.3495497 | -2.1563502 | 0.0000000 | -2.2306507 | 0.0000000 | 0.0000000 | 29 | 5 | 243.4839 | 192.94602 |
| 0.5215841 | 0.0000000 | -3.0991773 | -6.0187614 | -39.5102269 | 0.0000000 | -1.8814260 | 0.0000000 | 0.0000000 | 0.0000000 | 0.0000000 | 0.0000000 | -4.0155287 | 0.0000000 | 0.0000000 | 0.0000000 | 0.0000000 | 0.0000000 | -1.1885490 | 0.0000000 | -0.1376005 | -0.4209868 | 0.0000000 | 0.0000000 | 0.0000000 | 29 | 6 | 140.2925 | 89.94752 |
| 0.0000000 | -1.2966884 | 0.0000000 | -1.3112992 | -9.3130935 | 0.0000000 | -0.5564225 | 0.0000000 | -0.0085447 | 0.0000000 | 0.0000000 | 0.0000000 | -0.5910416 | 0.0000000 | 0.0000000 | 0.0000000 | 0.0000000 | 0.0000000 | -0.5470653 | -0.2369995 | -0.3836597 | 0.0000000 | -0.2380133 | -0.2511457 | 0.0000000 | 30 | 1 | 467.8221 | 413.45689 |
| 0.4173479 | -0.9508469 | -0.5555624 | -1.3387467 | -13.6333103 | 0.0000000 | -0.9649324 | 0.0000000 | 0.0000000 | 0.0000000 | 0.0000000 | 0.0000000 | -1.3853710 | 0.0000000 | 0.0000000 | 0.0000000 | 0.0000000 | -0.1446415 | -0.1675298 | -0.1249104 | -1.1169388 | 0.0000000 | -0.2758093 | -0.4098783 | 0.0000000 | 30 | 2 | 451.1193 | 398.35433 |
| 1.1923639 | -0.7353234 | -0.3320602 | -2.2229535 | -28.0343870 | 0.0000000 | -1.7003984 | 0.0000000 | 0.0000000 | 0.0000000 | 0.0000000 | 0.0000000 | -2.1441018 | 0.0000000 | 0.0000000 | 0.0000000 | 0.0000000 | 0.0000000 | -0.3003158 | 0.0000000 | -2.3684914 | 0.0000000 | -1.0417668 | 0.0000000 | -0.2080266 | 30 | 3 | 364.2643 | 312.78326 |
| 0.9327678 | -1.4399158 | -0.1522520 | -3.1719907 | -12.8748534 | 0.0000000 | -2.3198919 | 0.0000000 | -0.1218996 | -0.1215454 | -0.0433606 | 0.0000000 | -1.3883373 | 0.0000000 | 0.0000000 | 0.0000000 | 0.0000000 | 0.0000000 | -0.3416189 | -0.1881088 | -1.6209400 | 0.0000000 | -0.8752858 | 0.0000000 | -0.4680176 | 30 | 4 | 298.5552 | 247.61612 |
| 2.3337413 | 0.0000000 | -3.0359619 | -3.6082242 | -41.1210881 | 0.0000000 | -2.5669454 | 0.0000000 | 0.0000000 | 0.0000000 | 0.0000000 | 0.0000000 | -1.7329934 | 0.0000000 | 0.0000000 | 0.0000000 | 0.0000000 | 0.0000000 | -0.4030767 | -0.4321372 | -2.0438411 | 0.0000000 | -1.2508517 | 0.0000000 | 0.0000000 | 30 | 5 | 192.1543 | 141.61458 |
| 4.0830048 | 0.0000000 | -3.2818464 | -4.8535217 | -16.9672493 | 0.0000000 | -5.4584999 | 0.0000000 | 0.0000000 | -0.0353623 | 0.0000000 | 0.0000000 | -3.1914819 | -0.0108728 | 0.0000000 | 0.0000000 | 0.0000000 | 0.0000000 | -0.1476690 | 0.0000000 | -2.6237503 | 0.0000000 | -1.6709859 | 0.0000000 | 0.0000000 | 30 | 6 | 130.4782 | 80.15891 |
| 0.0202399 | -0.7783388 | -0.6983485 | -0.3885202 | -5.6997589 | -0.0654764 | -0.3904214 | 0.0000000 | 0.0000000 | -0.0003469 | -0.0018246 | -0.0259514 | -0.8447359 | -0.0250992 | -0.0228110 | 0.0000000 | -0.0077072 | -0.1170469 | -0.3855887 | -0.0841525 | -0.6474153 | -0.0083939 | -0.0911876 | -0.3799539 | -0.0077467 | 31 | 1 | 465.3065 | 410.93735 |
| 0.1462538 | -0.9023573 | -0.9140579 | -1.2209393 | -13.3675553 | 0.0000000 | -1.2313712 | 0.0000000 | 0.0000000 | 0.0000000 | 0.0000000 | 0.0000000 | -0.8602138 | 0.0000000 | 0.0000000 | 0.0000000 | 0.0000000 | 0.0000000 | -0.5893781 | 0.0000000 | -0.5255104 | 0.0000000 | -0.3814003 | 0.0000000 | 0.0000000 | 31 | 2 | 461.1229 | 408.37036 |
| 1.0081776 | -0.6002511 | -1.1779525 | -1.8378966 | -13.4824320 | 0.0000000 | -1.7815438 | 0.0000000 | 0.0000000 | 0.0000000 | 0.0000000 | 0.0000000 | -1.0575400 | 0.0000000 | 0.0000000 | 0.0000000 | 0.0000000 | 0.0000000 | -0.6567588 | 0.0000000 | -1.6376793 | 0.0000000 | -0.6134668 | -0.1973364 | 0.0000000 | 31 | 3 | 362.7912 | 310.90496 |
| 1.5235560 | -1.1287454 | -1.5087782 | -2.5531935 | -18.5748175 | 0.0000000 | -2.0361749 | 0.0000000 | 0.0000000 | 0.0000000 | 0.0000000 | 0.0000000 | -2.9230681 | 0.0000000 | 0.0000000 | 0.0000000 | 0.0000000 | 0.0000000 | 0.0000000 | 0.0000000 | -1.8608447 | 0.0000000 | -1.2662497 | 0.0000000 | 0.0000000 | 31 | 4 | 257.8717 | 206.82618 |
| 2.3501483 | -0.4420155 | -2.5323842 | -3.8937914 | -17.0059443 | -0.6924069 | -2.6837156 | 0.0000000 | 0.0000000 | 0.0000000 | 0.0000000 | 0.0000000 | -1.3439116 | 0.0000000 | 0.0000000 | 0.0000000 | 0.0000000 | 0.0000000 | 0.0000000 | -1.5409083 | -1.6674099 | 0.0000000 | -1.7133773 | 0.0000000 | 0.0000000 | 31 | 5 | 182.3712 | 131.93487 |
| 2.7729567 | 0.0000000 | -2.4187513 | -6.5513214 | -10.0170453 | 0.0000000 | -2.6535470 | 0.0000000 | 0.0000000 | 0.0000000 | 0.0000000 | 0.0000000 | -2.3681178 | 0.0000000 | 0.0000000 | 0.0000000 | 0.0000000 | 0.0000000 | 0.0000000 | 0.0000000 | -3.1474127 | 0.0000000 | -1.3741473 | 0.0000000 | 0.0000000 | 31 | 6 | 138.9429 | 88.56646 |
| 0.0167306 | -1.3254185 | -0.2193756 | -0.7977661 | -6.9382141 | 0.0000000 | -0.6058331 | 0.0000000 | 0.0000000 | 0.0000000 | 0.0000000 | 0.0000000 | -0.4795193 | 0.0000000 | 0.0000000 | 0.0000000 | 0.0000000 | -0.1685019 | -0.3333562 | 0.0000000 | -0.7176624 | 0.0000000 | -0.0825811 | -0.2982141 | 0.0000000 | 32 | 1 | 497.7843 | 443.55226 |
| 0.3251378 | -0.9545240 | -0.6594197 | -1.8077986 | -7.5020724 | 0.0000000 | -1.1933087 | 0.0000000 | 0.0000000 | 0.0000000 | 0.0000000 | 0.0000000 | -0.3234234 | 0.0000000 | 0.0000000 | 0.0000000 | 0.0000000 | -0.0877394 | -0.4397763 | -0.5280914 | -0.3942308 | 0.0000000 | -0.6534054 | 0.0000000 | 0.0000000 | 32 | 2 | 438.8575 | 385.92295 |
| 0.7218429 | -0.5304134 | -0.8457539 | -2.1492256 | -20.2748098 | 0.0000000 | -0.9054922 | 0.0000000 | 0.0000000 | 0.0000000 | 0.0000000 | 0.0000000 | -0.1900962 | 0.0000000 | 0.0000000 | 0.0000000 | 0.0000000 | 0.0000000 | -0.1517303 | -0.4103364 | -1.7990046 | 0.0000000 | -0.7722350 | 0.0000000 | 0.0000000 | 32 | 3 | 408.1533 | 356.46109 |
| 2.5154049 | 0.0000000 | -2.2238345 | -2.6515807 | -16.7849756 | 0.0000000 | -2.6195572 | 0.0000000 | 0.0000000 | 0.0000000 | 0.0000000 | 0.0000000 | -0.7200458 | 0.0000000 | 0.0000000 | 0.0000000 | 0.0000000 | 0.0000000 | 0.0000000 | 0.0000000 | -2.1354926 | 0.0000000 | -1.8064621 | 0.0000000 | 0.0000000 | 32 | 4 | 289.8200 | 238.65912 |
| 3.2584779 | 0.0000000 | -3.2773603 | -3.0441091 | -34.2646190 | 0.0000000 | -2.6905865 | 0.0000000 | 0.0000000 | 0.0000000 | -0.5460312 | -0.8556042 | -0.6233644 | 0.0000000 | 0.0000000 | 0.0000000 | -0.1101732 | 0.0000000 | 0.0000000 | 0.0000000 | -2.5944757 | -0.0386191 | -1.1106775 | -0.3326996 | -0.8449276 | 32 | 5 | 195.7439 | 145.13990 |
| 3.7631262 | -1.0681134 | -0.7213462 | -7.4292133 | -55.4305853 | 0.0000000 | -2.8114361 | 0.0000000 | 0.0000000 | 0.0000000 | 0.0000000 | 0.0000000 | -3.9472666 | 0.0000000 | 0.0000000 | 0.0000000 | 0.0000000 | 0.0000000 | 0.0000000 | 0.0000000 | -2.6088657 | 0.0000000 | -2.6196554 | 0.0000000 | 0.0000000 | 32 | 6 | 160.0305 | 109.64897 |
| 0.0000000 | -1.2710084 | -0.1407663 | -1.3753413 | -8.5063449 | 0.0000000 | -0.6151738 | 0.0000000 | 0.0000000 | 0.0000000 | 0.0000000 | 0.0000000 | -0.3690089 | 0.0000000 | 0.0000000 | 0.0000000 | 0.0000000 | -0.0440720 | -0.5786716 | 0.0000000 | -0.5766355 | 0.0000000 | -0.1405086 | -0.2365072 | 0.0000000 | 33 | 1 | 461.9733 | 407.52886 |
| 0.3898815 | -0.3642190 | -1.1879792 | -0.4321499 | -21.5222601 | 0.0000000 | -1.0042832 | 0.0000000 | 0.0000000 | 0.0000000 | 0.0000000 | 0.0000000 | -0.8899890 | 0.0000000 | 0.0000000 | 0.0000000 | 0.0000000 | 0.0000000 | -0.3594737 | 0.0000000 | -1.3338453 | 0.0000000 | -0.2366999 | -0.2323156 | 0.0000000 | 33 | 2 | 460.0587 | 407.42728 |
| 0.6613655 | -1.3805360 | -0.5697789 | -2.4769698 | -14.1054010 | 0.0000000 | -1.4076864 | 0.0000000 | 0.0000000 | 0.0000000 | 0.0000000 | 0.0000000 | -1.2119500 | 0.0000000 | 0.0000000 | 0.0000000 | 0.0000000 | 0.0000000 | -0.5200927 | 0.0000000 | -1.0876796 | 0.0000000 | -0.8740647 | 0.0000000 | 0.0000000 | 33 | 3 | 372.3933 | 320.58864 |
| 1.5270224 | 0.0000000 | -2.3663637 | -2.1216775 | -16.0133312 | 0.0000000 | -1.7146726 | 0.0000000 | 0.0000000 | 0.0000000 | 0.0000000 | 0.0000000 | -0.4963928 | 0.0000000 | 0.0000000 | 0.0000000 | 0.0000000 | 0.0000000 | -0.7926177 | 0.0000000 | -2.1952464 | 0.0000000 | -0.5932499 | 0.0000000 | 0.0000000 | 33 | 4 | 305.0844 | 253.94205 |
| 1.7255221 | 0.0000000 | -2.1366039 | -1.5150673 | -53.2447407 | 0.0000000 | -2.8429411 | 0.0000000 | 0.0000000 | 0.0000000 | 0.0000000 | 0.0000000 | -2.0451988 | 0.0000000 | 0.0000000 | 0.0000000 | -0.0039944 | 0.0000000 | -0.1306734 | -1.2182411 | -1.3419880 | 0.0000000 | -1.2636175 | 0.0000000 | 0.0000000 | 33 | 5 | 228.8155 | 178.27590 |
| 4.4627215 | -2.9886822 | -0.1379666 | -7.3430127 | -7.9277718 | 0.0000000 | -5.4332741 | -0.0971561 | -0.8983784 | 0.0000000 | 0.0000000 | 0.0000000 | -3.0174681 | 0.0000000 | 0.0000000 | 0.0000000 | -1.2125800 | 0.0000000 | 0.0000000 | 0.0000000 | -4.9999903 | 0.0000000 | -0.8272098 | 0.0000000 | 0.0000000 | 33 | 6 | 138.6845 | 88.41967 |
| 0.0000000 | -0.7107341 | -0.5148819 | -0.8656932 | -6.9778513 | 0.0000000 | -0.2248603 | 0.0000000 | 0.0000000 | -0.0067602 | 0.0000000 | 0.0000000 | -0.5277250 | 0.0000000 | 0.0000000 | 0.0000000 | 0.0000000 | -0.2095219 | -0.4692750 | -0.0227861 | -0.5382075 | 0.0000000 | -0.1471303 | -0.2875389 | 0.0000000 | 34 | 1 | 457.0407 | 402.59651 |
| 0.4876083 | -0.8772235 | -1.0584717 | -0.9257366 | -16.0271630 | 0.0000000 | -0.9612403 | 0.0000000 | 0.0000000 | 0.0000000 | 0.0000000 | 0.0000000 | -1.2892044 | 0.0000000 | 0.0000000 | 0.0000000 | 0.0000000 | -0.0994492 | 0.0000000 | 0.0000000 | -0.8377641 | 0.0000000 | -0.8058396 | 0.0000000 | 0.0000000 | 34 | 2 | 413.0674 | 360.04790 |
| 0.9641127 | -0.9452600 | -1.4512355 | -1.8953951 | -15.3725647 | 0.0000000 | -1.7290650 | 0.0000000 | 0.0000000 | -0.0429796 | 0.0000000 | 0.0000000 | -2.1350888 | 0.0000000 | 0.0000000 | 0.0000000 | 0.0000000 | 0.0000000 | -0.0294412 | -0.4844875 | -1.4449410 | 0.0000000 | -0.9090113 | 0.0000000 | 0.0000000 | 34 | 3 | 328.6620 | 276.88875 |
| 1.7167529 | -0.3369587 | -2.5282428 | -1.6572699 | -33.6180589 | 0.0000000 | -3.5444248 | 0.0000000 | 0.0000000 | 0.0000000 | -0.0916194 | 0.0000000 | -1.2062832 | 0.0000000 | 0.0000000 | 0.0000000 | 0.0000000 | 0.0000000 | -0.1686468 | 0.0000000 | -1.1869825 | 0.0000000 | -0.7601139 | -0.1914519 | -1.8060265 | 34 | 4 | 279.7872 | 228.72989 |
| 2.3940381 | 0.0000000 | -3.5752151 | -2.2553898 | -31.8049362 | 0.0000000 | -3.5129859 | 0.0000000 | 0.0000000 | 0.0000000 | 0.0000000 | 0.0000000 | -3.1655812 | 0.0000000 | 0.0000000 | 0.0000000 | -0.2255556 | -0.1874524 | 0.0000000 | -0.2243890 | -2.1352615 | 0.0000000 | -0.2514525 | -0.3625018 | -0.2006677 | 34 | 5 | 207.5500 | 156.86090 |
| 3.8431610 | -0.0371674 | -3.4925118 | -1.8791602 | -10.7639275 | 0.0000000 | -4.0847274 | 0.0000000 | -0.7837670 | 0.0000000 | -0.3623894 | 0.0000000 | -3.1297368 | 0.0000000 | 0.0000000 | 0.0000000 | 0.0000000 | 0.0000000 | 0.0000000 | 0.0000000 | -3.2363564 | 0.0000000 | -0.8969170 | 0.0000000 | 0.0000000 | 34 | 6 | 186.3966 | 136.01184 |
| 0.0343313 | -1.0966698 | -0.3250122 | -1.1850254 | -8.1639765 | 0.0000000 | -0.5416057 | 0.0000000 | -0.1499277 | -0.0167272 | 0.0000000 | 0.0000000 | -0.6516818 | 0.0000000 | 0.0000000 | 0.0000000 | 0.0000000 | -0.0821610 | -0.3222349 | -0.3059231 | -0.4245899 | 0.0000000 | -0.1337255 | -0.3866937 | 0.0000000 | 35 | 1 | 450.2567 | 395.83461 |
| 0.3122875 | -0.9101004 | -0.5169146 | -1.1351598 | -9.1626963 | 0.0000000 | -1.0815484 | 0.0000000 | 0.0000000 | 0.0000000 | 0.0000000 | 0.0000000 | -0.4512444 | 0.0000000 | 0.0000000 | 0.0000000 | 0.0000000 | -0.0988459 | -0.4486955 | -0.0373828 | -1.2832250 | 0.0000000 | -0.3590736 | 0.0000000 | 0.0000000 | 35 | 2 | 446.7999 | 393.68125 |
| 0.7637849 | -0.8164750 | -1.0555853 | -1.1939858 | -18.2215850 | 0.0000000 | -1.4795448 | 0.0000000 | 0.0000000 | 0.0000000 | 0.0000000 | 0.0000000 | -1.5179216 | 0.0000000 | 0.0000000 | 0.0000000 | 0.0000000 | 0.0000000 | -0.1949034 | -0.0473291 | -1.3837696 | 0.0000000 | -0.9538135 | 0.0000000 | -0.1850108 | 35 | 3 | 389.5493 | 337.81111 |
| 1.7066462 | 0.0000000 | -2.4046401 | -2.3274103 | -30.5315353 | 0.0000000 | -1.9928571 | 0.0000000 | 0.0000000 | 0.0000000 | 0.0000000 | 0.0000000 | -1.6998444 | 0.0000000 | 0.0000000 | 0.0000000 | 0.0000000 | -0.7407289 | 0.0000000 | 0.0000000 | -1.2668507 | 0.0000000 | -0.9123241 | 0.0000000 | 0.0000000 | 35 | 4 | 309.2564 | 258.16048 |
| 2.5195437 | -0.5768299 | -2.8059547 | -3.1645325 | -13.6723756 | 0.0000000 | -5.3956043 | 0.0000000 | 0.0000000 | 0.0000000 | 0.0000000 | 0.0000000 | -1.7270956 | 0.0000000 | 0.0000000 | 0.0000000 | 0.0000000 | 0.0000000 | -1.0940871 | -0.0427114 | -1.8033834 | 0.0000000 | -0.4919139 | 0.0000000 | 0.0000000 | 35 | 5 | 184.3128 | 133.76729 |
| 6.0108096 | 0.0000000 | -3.7808847 | -5.1420115 | -20.4818515 | -0.8927505 | -5.1186380 | 0.0000000 | 0.0000000 | -0.7713596 | 0.0000000 | 0.0000000 | -3.5500142 | 0.0000000 | 0.0000000 | -0.0789048 | -1.0464094 | 0.0000000 | 0.0000000 | 0.0000000 | -1.9204937 | 0.0000000 | -2.8633196 | 0.0000000 | -0.7176733 | 35 | 6 | 157.2505 | 106.87501 |
| 0.0000000 | -0.9767929 | -0.1293908 | -1.0059074 | -11.5484490 | 0.0000000 | -0.5357295 | 0.0000000 | 0.0000000 | -0.0853645 | 0.0000000 | 0.0000000 | -0.5415408 | 0.0000000 | 0.0000000 | 0.0000000 | 0.0000000 | -0.1915670 | -0.4977992 | -0.0941570 | -0.5165100 | 0.0000000 | -0.0648940 | -0.4315619 | 0.0000000 | 36 | 1 | 481.2808 | 426.95749 |
| 0.1887435 | -1.3306530 | -0.3365453 | -1.7926969 | -14.2249643 | 0.0000000 | -1.0949356 | 0.0000000 | 0.0000000 | 0.0000000 | 0.0000000 | 0.0000000 | -0.9833957 | 0.0000000 | 0.0000000 | 0.0000000 | 0.0000000 | -0.1522770 | -0.0493158 | 0.0000000 | -0.5237902 | 0.0000000 | -0.6323773 | 0.0000000 | 0.0000000 | 36 | 2 | 469.0914 | 416.21506 |
| 0.8494561 | -0.9687977 | -0.6287463 | -2.9067690 | -22.4867304 | 0.0000000 | -1.7726218 | 0.0000000 | 0.0000000 | 0.0000000 | 0.0000000 | 0.0000000 | -0.2447108 | 0.0000000 | 0.0000000 | 0.0000000 | -0.0739988 | 0.0000000 | 0.0000000 | -0.8034948 | -0.8616443 | 0.0000000 | -1.1997668 | 0.0000000 | 0.0000000 | 36 | 3 | 375.6659 | 323.89512 |
| 2.3278227 | -0.6976276 | -1.7077177 | -2.4634526 | -29.8631162 | 0.0000000 | -2.3588425 | 0.0000000 | 0.0000000 | 0.0000000 | 0.0000000 | 0.0000000 | -1.4376447 | 0.0000000 | 0.0000000 | 0.0000000 | 0.0000000 | 0.0000000 | 0.0000000 | -1.2146213 | -1.7394589 | 0.0000000 | -1.7588760 | 0.0000000 | 0.0000000 | 36 | 4 | 264.4626 | 213.46120 |
| 2.8028721 | 0.0000000 | -4.8068973 | 0.0000000 | -35.1849612 | 0.0000000 | -3.5857966 | 0.0000000 | 0.0000000 | 0.0000000 | 0.0000000 | 0.0000000 | -2.1685281 | 0.0000000 | 0.0000000 | 0.0000000 | -1.2595978 | -0.2012955 | -0.2819203 | -0.3727363 | -2.4630037 | 0.0000000 | -0.3873917 | 0.0000000 | 0.0000000 | 36 | 5 | 198.6426 | 148.16785 |
| 3.8226310 | 0.0000000 | -2.0167878 | -3.7857499 | 0.0000000 | -5.6683731 | -3.4748923 | 0.0000000 | 0.0000000 | 0.0000000 | -1.7690601 | 0.0000000 | -3.9125737 | 0.0000000 | 0.0000000 | 0.0000000 | -0.3220062 | 0.0000000 | -0.7248714 | -0.1948628 | -3.1193339 | 0.0000000 | 0.0000000 | -0.4085312 | -1.7760686 | 36 | 6 | 147.0320 | 96.65350 |
| 0.0000000 | -1.2549085 | 0.0000000 | -1.2698979 | -0.0652847 | -0.9493487 | -0.3682675 | 0.0000000 | 0.0000000 | -0.0022098 | 0.0000000 | 0.0000000 | -0.2093636 | 0.0000000 | 0.0000000 | 0.0000000 | 0.0000000 | -0.0638784 | -0.7388169 | -0.4217899 | -0.4603627 | 0.0000000 | -0.1717263 | -0.2408627 | 0.0000000 | 37 | 1 | 448.3060 | 393.87630 |
| 0.3484880 | -1.1334843 | -0.5076148 | -0.8841397 | -26.1375475 | -0.0098132 | -1.3113040 | 0.0000000 | 0.0000000 | 0.0000000 | 0.0000000 | -0.0000789 | -0.8907577 | -0.0000866 | -0.0000505 | 0.0000000 | -0.0307212 | -0.0304975 | -0.3421730 | -0.1310237 | -0.8685178 | 0.0000000 | -0.5984088 | -0.0000764 | -0.0975795 | 37 | 2 | 426.4038 | 373.57814 |
| 0.7607215 | -0.1079258 | -1.7466064 | -0.9125430 | -30.1968000 | 0.0000000 | -1.6735459 | 0.0000000 | 0.0000000 | 0.0000000 | 0.0000000 | 0.0000000 | -0.9441977 | 0.0000000 | 0.0000000 | 0.0000000 | 0.0000000 | 0.0000000 | -0.0949155 | -0.3908753 | -0.8200624 | 0.0000000 | -1.0566672 | 0.0000000 | 0.0000000 | 37 | 3 | 348.1873 | 296.51488 |
| 2.3377710 | -0.0528728 | -2.2263207 | -2.7187279 | -39.4494570 | 0.0000000 | -2.9976184 | 0.0000000 | 0.0000000 | -0.2365301 | 0.0000000 | 0.0000000 | -1.1100694 | 0.0000000 | 0.0000000 | 0.0000000 | -0.4437122 | 0.0000000 | 0.0000000 | -0.9362575 | -1.0886988 | 0.0000000 | -1.5942185 | 0.0000000 | -0.3364504 | 37 | 4 | 271.9158 | 220.92356 |
| 1.7524189 | 0.0000000 | -3.2217289 | -0.0798229 | -20.7019569 | 0.0000000 | -3.8714238 | 0.0000000 | 0.0000000 | 0.0000000 | 0.0000000 | 0.0000000 | -3.2253795 | 0.0000000 | 0.0000000 | 0.0000000 | -0.5281868 | 0.0000000 | -0.4748120 | 0.0000000 | -1.4325253 | 0.0000000 | -0.4117955 | 0.0000000 | 0.0000000 | 37 | 5 | 235.9058 | 185.34438 |
| 3.6542115 | 0.0000000 | -0.9803331 | -7.3555791 | -20.7420085 | -0.1431862 | -3.5334408 | 0.0000000 | 0.0000000 | 0.0000000 | 0.0000000 | 0.0000000 | -0.8218850 | 0.0000000 | 0.0000000 | 0.0000000 | 0.0000000 | 0.0000000 | 0.0000000 | 0.0000000 | -2.4773484 | 0.0000000 | -2.8200732 | 0.0000000 | 0.0000000 | 37 | 6 | 166.4830 | 116.11910 |
| 0.0307358 | -1.2002418 | -0.2098731 | -1.0586242 | -6.0367363 | 0.0000000 | -0.4250800 | 0.0000000 | -0.1004966 | 0.0000000 | 0.0000000 | 0.0000000 | -0.6161193 | 0.0000000 | 0.0000000 | 0.0000000 | 0.0000000 | -0.3807385 | -0.1552598 | -0.0603274 | -0.4671306 | 0.0000000 | -0.1217866 | -0.2989034 | 0.0000000 | 38 | 1 | 504.5984 | 450.31087 |
| 0.1824208 | -0.9850085 | -0.5861390 | -1.6285451 | -15.2334269 | 0.0000000 | -1.2190736 | 0.0000000 | 0.0000000 | 0.0000000 | 0.0000000 | 0.0000000 | -0.4151535 | 0.0000000 | 0.0000000 | 0.0000000 | 0.0000000 | 0.0000000 | -0.5292860 | 0.0000000 | -0.9333233 | 0.0000000 | -0.2654382 | 0.0000000 | 0.0000000 | 38 | 2 | 450.9875 | 397.93141 |
| 1.1277475 | -0.7213053 | -1.7128398 | -0.6458091 | -15.8549490 | 0.0000000 | -1.4258569 | 0.0000000 | 0.0000000 | 0.0000000 | 0.0000000 | 0.0000000 | -1.1738724 | 0.0000000 | 0.0000000 | 0.0000000 | 0.0000000 | 0.0000000 | -0.1008410 | -0.0706661 | -1.8299968 | 0.0000000 | -0.8870817 | -0.1538661 | -0.4061885 | 38 | 3 | 367.0305 | 315.28703 |
| 1.4229723 | 0.0000000 | -2.4631842 | -1.5981362 | -23.1267090 | 0.0000000 | -2.3325627 | 0.0000000 | 0.0000000 | 0.0000000 | 0.0000000 | 0.0000000 | -1.9332546 | 0.0000000 | 0.0000000 | 0.0000000 | -0.1009399 | 0.0000000 | -0.0069125 | 0.0000000 | -2.0308322 | 0.0000000 | -0.7637290 | 0.0000000 | 0.0000000 | 38 | 4 | 250.3988 | 199.28879 |
| 0.2793765 | -1.3857179 | -0.8513902 | -2.3322051 | -36.7458425 | 0.0000000 | -1.5477480 | 0.0000000 | 0.0000000 | 0.0000000 | 0.0000000 | 0.0000000 | -1.7264229 | 0.0000000 | 0.0000000 | 0.0000000 | 0.0000000 | 0.0000000 | -0.6049875 | -0.0680028 | -2.1554475 | 0.0000000 | 0.0000000 | 0.0000000 | 0.0000000 | 38 | 5 | 249.8731 | 199.31107 |
| 1.7265822 | -0.6409809 | -2.5409677 | -3.1299117 | -36.5075853 | 0.0000000 | -3.9956844 | 0.0000000 | 0.0000000 | 0.0000000 | 0.0000000 | 0.0000000 | -3.0233288 | 0.0000000 | 0.0000000 | 0.0000000 | 0.0000000 | 0.0000000 | 0.0000000 | -1.5600353 | -1.2873086 | 0.0000000 | -0.2732170 | -0.0506488 | -3.3723414 | 38 | 6 | 160.0439 | 109.70549 |
| 0.0080546 | -0.9160965 | -0.4063400 | -1.3626135 | -3.7367449 | 0.0000000 | -0.5373600 | 0.0000000 | 0.0000000 | 0.0000000 | 0.0000000 | 0.0000000 | -0.3370274 | 0.0000000 | 0.0000000 | 0.0000000 | 0.0000000 | -0.0782632 | -0.6737520 | 0.0000000 | -0.5653900 | 0.0000000 | -0.1308504 | -0.2093627 | 0.0000000 | 39 | 1 | 492.1676 | 438.01230 |
| 0.1603480 | -1.1103476 | -0.5271155 | -1.3465753 | -17.4620647 | 0.0000000 | -0.9983115 | 0.0000000 | 0.0000000 | 0.0000000 | 0.0000000 | 0.0000000 | -0.4967765 | 0.0000000 | 0.0000000 | 0.0000000 | 0.0000000 | 0.0000000 | -0.1526267 | -0.1580309 | -0.8726831 | 0.0000000 | -0.3347173 | -0.2547105 | 0.0000000 | 39 | 2 | 458.8502 | 405.98059 |
| 0.5361897 | -0.9167441 | -0.4513716 | -1.2907426 | -14.1928834 | 0.0000000 | -0.9414024 | 0.0000000 | 0.0000000 | 0.0000000 | 0.0000000 | 0.0000000 | -1.3466547 | 0.0000000 | 0.0000000 | 0.0000000 | 0.0000000 | 0.0000000 | 0.0000000 | 0.0000000 | -1.6212589 | 0.0000000 | -1.0531578 | 0.0000000 | 0.0000000 | 39 | 3 | 383.3012 | 331.46746 |
| 2.5514006 | -1.1514485 | -1.4083427 | -4.8554656 | -24.9309887 | 0.0000000 | -2.8422983 | 0.0000000 | 0.0000000 | 0.0000000 | 0.0000000 | 0.0000000 | -1.6026595 | 0.0000000 | 0.0000000 | 0.0000000 | 0.0000000 | 0.0000000 | 0.0000000 | 0.0000000 | -1.3263133 | 0.0000000 | -2.1869217 | 0.0000000 | 0.0000000 | 39 | 4 | 256.5491 | 205.53755 |
| 3.0723239 | -0.3192722 | -2.4179392 | -1.4257518 | -33.5816333 | 0.0000000 | -3.4540192 | 0.0000000 | 0.0000000 | 0.0000000 | 0.0000000 | 0.0000000 | -1.8529405 | 0.0000000 | 0.0000000 | 0.0000000 | -0.1664453 | 0.0000000 | 0.0000000 | 0.0000000 | -1.5024800 | 0.0000000 | -2.4255388 | 0.0000000 | 0.0000000 | 39 | 5 | 235.1302 | 184.54167 |
| 3.0655832 | -1.3746031 | -2.5225984 | -4.1181836 | -21.6312529 | 0.0000000 | -6.3863515 | 0.0000000 | 0.0000000 | 0.0000000 | 0.0000000 | 0.0000000 | -3.2906417 | 0.0000000 | 0.0000000 | 0.0000000 | 0.0000000 | -0.9976407 | 0.0000000 | 0.0000000 | -1.8691264 | 0.0000000 | 0.0000000 | 0.0000000 | -0.4372038 | 39 | 6 | 163.3243 | 112.99778 |
| 0.0123178 | -1.4930512 | -0.0643313 | -0.9629016 | -8.7804027 | 0.0000000 | -0.3815541 | 0.0000000 | 0.0000000 | -0.0929756 | 0.0000000 | 0.0000000 | -0.7293377 | 0.0000000 | 0.0000000 | 0.0000000 | 0.0000000 | -0.1311348 | -0.0882824 | 0.0000000 | -0.7305028 | 0.0000000 | -0.1348614 | -0.4114105 | 0.0000000 | 40 | 1 | 478.2598 | 424.00998 |
| 0.1237614 | -0.9676326 | -0.6288581 | -1.4656821 | -3.5642659 | -0.0682698 | -0.8965840 | 0.0000000 | 0.0000000 | -0.0046039 | 0.0000000 | -0.0184036 | -0.0159708 | -0.0175100 | -0.0167373 | 0.0000000 | 0.0000000 | -0.0215714 | -0.5543509 | -0.7046340 | -0.7071975 | 0.0000000 | -0.2057311 | -0.3129435 | -0.0068991 | 40 | 2 | 439.0575 | 386.19191 |
| 1.0881672 | 0.0000000 | -2.1737791 | -1.1425665 | -19.4542993 | 0.0000000 | -2.0489674 | 0.0000000 | 0.0000000 | 0.0000000 | 0.0000000 | 0.0000000 | -2.1801294 | 0.0000000 | 0.0000000 | 0.0000000 | -0.3292535 | 0.0000000 | 0.0000000 | 0.0000000 | -1.5552803 | 0.0000000 | -0.6426128 | 0.0000000 | 0.0000000 | 40 | 3 | 335.5340 | 283.72014 |
| 2.3610414 | -0.0150207 | -2.6148672 | -1.1685745 | -12.3951919 | 0.0000000 | -3.6076099 | 0.0000000 | 0.0000000 | 0.0000000 | 0.0000000 | 0.0000000 | -1.8656127 | 0.0000000 | 0.0000000 | 0.0000000 | 0.0000000 | 0.0000000 | -0.0652246 | 0.0000000 | -2.0331657 | 0.0000000 | -1.2317811 | 0.0000000 | 0.0000000 | 40 | 4 | 305.2713 | 254.22971 |
| 3.4087361 | -0.6131151 | -3.8536283 | -0.1127798 | -39.8818181 | 0.0000000 | -3.0067064 | 0.0000000 | 0.0000000 | 0.0000000 | -0.0651280 | 0.0000000 | -3.5714421 | 0.0000000 | 0.0000000 | 0.0000000 | 0.0000000 | 0.0000000 | -0.2186632 | 0.0000000 | -2.5966701 | 0.0000000 | -1.8006573 | 0.0000000 | 0.0000000 | 40 | 5 | 215.1013 | 164.55223 |
| 2.7895068 | 0.0000000 | -4.6393678 | -4.7180345 | -18.0038296 | 0.0000000 | -1.2156975 | 0.0000000 | -0.0221380 | 0.0000000 | -0.1080254 | 0.0000000 | -4.7358464 | 0.0000000 | 0.0000000 | 0.0000000 | 0.0000000 | 0.0000000 | 0.0000000 | -0.3285863 | -2.0379497 | 0.0000000 | -1.7857470 | 0.0000000 | 0.0000000 | 40 | 6 | 118.9088 | 68.59538 |
| 0.0000000 | -1.2039007 | -0.1435202 | -1.2488672 | -0.5934741 | 0.0000000 | -0.4613868 | 0.0000000 | 0.0000000 | -0.0078608 | 0.0000000 | 0.0000000 | -0.4461783 | 0.0000000 | 0.0000000 | 0.0000000 | 0.0000000 | -0.2129968 | -0.4580133 | -0.1767542 | -0.5507772 | 0.0000000 | 0.0000000 | -0.5016875 | 0.0000000 | 41 | 1 | 490.4430 | 436.05617 |
| 0.6218830 | -0.5334032 | -1.1458797 | -0.8165560 | -19.1620925 | 0.0000000 | -1.2364776 | 0.0000000 | 0.0000000 | 0.0000000 | 0.0000000 | 0.0000000 | -0.6408339 | 0.0000000 | 0.0000000 | 0.0000000 | 0.0000000 | 0.0000000 | -0.2616577 | -0.1451593 | -0.9153207 | 0.0000000 | -0.7861991 | 0.0000000 | 0.0000000 | 41 | 2 | 440.1311 | 387.19335 |
| 0.3988787 | -1.1283322 | -0.5825670 | -2.1013756 | -18.8054521 | 0.0000000 | -1.2905496 | 0.0000000 | 0.0000000 | 0.0000000 | 0.0000000 | 0.0000000 | -1.1562310 | 0.0000000 | 0.0000000 | 0.0000000 | -0.0510193 | 0.0000000 | -0.1716679 | -0.5773637 | -1.0053695 | 0.0000000 | -0.7973074 | 0.0000000 | 0.0000000 | 41 | 3 | 387.6151 | 336.08187 |
| 2.1074909 | -0.9013149 | -1.5130933 | -4.3460169 | -9.5592400 | 0.0000000 | -2.7934854 | 0.0000000 | 0.0000000 | 0.0000000 | 0.0000000 | 0.0000000 | -1.9536553 | 0.0000000 | 0.0000000 | 0.0000000 | 0.0000000 | 0.0000000 | 0.0000000 | -0.2900864 | -1.6259511 | 0.0000000 | -1.6097953 | 0.0000000 | -0.3245440 | 41 | 4 | 250.2534 | 199.22189 |
| 1.5976319 | 0.0000000 | -3.1558156 | -2.2514130 | -27.7266669 | -0.7331947 | -2.9213578 | 0.0000000 | 0.0000000 | 0.0000000 | 0.0000000 | 0.0000000 | -1.5205594 | 0.0000000 | 0.0000000 | 0.0000000 | 0.0000000 | 0.0000000 | 0.0000000 | -0.2660331 | -2.3217052 | 0.0000000 | -0.1635518 | 0.0000000 | -0.2060617 | 41 | 5 | 240.8937 | 190.33310 |
| 4.7888114 | 0.0000000 | -6.0514269 | -0.8844430 | -23.0193459 | 0.0000000 | -1.5860705 | 0.0000000 | 0.0000000 | 0.0000000 | -0.7330146 | 0.0000000 | -3.4653549 | 0.0000000 | 0.0000000 | 0.0000000 | -0.2069643 | 0.0000000 | 0.0000000 | 0.0000000 | -3.0196162 | 0.0000000 | -2.4166514 | 0.0000000 | 0.0000000 | 41 | 6 | 151.4007 | 101.04813 |
| 0.0000000 | -1.0022998 | -0.4894795 | -1.1397304 | -6.2344372 | 0.0000000 | -0.4154848 | 0.0000000 | -0.0396634 | 0.0000000 | 0.0000000 | -0.1479724 | -0.4572776 | 0.0000000 | 0.0000000 | 0.0000000 | -0.1715258 | -0.1090624 | -0.6832623 | -0.0021180 | -0.4535814 | 0.0000000 | -0.1010577 | -0.2000302 | 0.0000000 | 42 | 1 | 446.6856 | 392.26022 |
| 0.2468226 | -1.0984285 | -0.4556218 | -1.3632997 | -10.5162876 | 0.0000000 | -0.9134314 | 0.0000000 | 0.0000000 | 0.0000000 | 0.0000000 | 0.0000000 | -0.7692689 | 0.0000000 | 0.0000000 | 0.0000000 | 0.0000000 | -0.0544005 | -0.0635739 | -0.1895436 | -0.9726451 | 0.0000000 | -0.5342841 | -0.0847460 | 0.0000000 | 42 | 2 | 443.6475 | 390.77155 |
| 0.8057548 | -0.6863926 | -1.7923572 | -1.5033877 | -16.6013963 | 0.0000000 | -1.8347318 | 0.0000000 | 0.0000000 | 0.0000000 | 0.0000000 | 0.0000000 | -1.1489288 | 0.0000000 | 0.0000000 | 0.0000000 | -0.1785819 | 0.0000000 | -0.2354427 | -0.1511677 | -1.0516112 | 0.0000000 | -0.6658838 | 0.0000000 | 0.0000000 | 42 | 3 | 358.5393 | 306.68250 |
| 2.5287784 | -0.0034394 | -2.3839743 | -0.8021739 | -17.9933408 | 0.0000000 | -3.3722985 | 0.0000000 | 0.0000000 | 0.0000000 | 0.0000000 | 0.0000000 | -1.3051684 | 0.0000000 | 0.0000000 | 0.0000000 | 0.0000000 | 0.0000000 | 0.0000000 | -0.6799020 | -1.9614524 | 0.0000000 | -1.5455945 | -0.2823499 | 0.0000000 | 42 | 4 | 328.8665 | 277.88754 |
| 0.3303536 | 0.0000000 | -1.9010582 | -2.2190992 | -23.3764789 | 0.0000000 | -3.3189100 | 0.0000000 | 0.0000000 | 0.0000000 | 0.0000000 | 0.0000000 | -1.6618267 | 0.0000000 | 0.0000000 | 0.0000000 | 0.0000000 | 0.0000000 | 0.0000000 | 0.0000000 | -1.2280103 | 0.0000000 | 0.0000000 | 0.0000000 | 0.0000000 | 42 | 5 | 255.4107 | 204.76563 |
| 4.9819166 | -0.7991350 | -0.2914533 | -10.7125873 | -10.0972866 | 0.0000000 | -1.6615852 | 0.0000000 | 0.0000000 | -1.4777899 | -0.2824300 | 0.0000000 | -3.9057988 | 0.0000000 | 0.0000000 | -0.0425289 | 0.0000000 | 0.0000000 | 0.0000000 | 0.0000000 | -3.2287492 | 0.0000000 | -4.2928031 | 0.0000000 | 0.0000000 | 42 | 6 | 122.1294 | 71.87026 |
| 0.0000000 | -1.0098318 | -0.3062982 | -0.7822664 | -3.9164216 | 0.0000000 | -0.2799472 | 0.0000000 | 0.0000000 | -0.0586146 | 0.0000000 | 0.0000000 | -0.4596064 | 0.0000000 | 0.0000000 | 0.0000000 | 0.0000000 | -0.1667820 | -0.4493487 | 0.0000000 | -0.7704290 | 0.0000000 | 0.0000000 | -0.4586574 | 0.0000000 | 43 | 1 | 513.5007 | 459.27903 |
| 0.1829191 | -1.3789537 | -0.5486844 | -1.3974611 | -8.6893013 | 0.0000000 | -0.8174850 | 0.0000000 | 0.0000000 | -0.0884408 | 0.0000000 | 0.0000000 | -0.8457198 | 0.0000000 | 0.0000000 | 0.0000000 | 0.0000000 | 0.0000000 | -0.1605675 | -0.2200630 | -0.8921851 | 0.0000000 | -0.4684935 | -0.0261549 | 0.0000000 | 43 | 2 | 447.4644 | 394.56020 |
| 0.3069052 | -0.9501594 | -0.5913560 | -2.2268895 | -20.3853242 | 0.0000000 | -1.9344099 | 0.0000000 | 0.0000000 | 0.0000000 | 0.0000000 | 0.0000000 | -0.6164268 | 0.0000000 | 0.0000000 | 0.0000000 | 0.0000000 | 0.0000000 | -0.4477849 | -0.4216869 | -1.3395535 | 0.0000000 | -0.1854537 | 0.0000000 | -0.7956695 | 43 | 3 | 400.3113 | 348.64034 |
| 1.4245068 | 0.0000000 | -1.8011841 | -3.0007939 | -8.8727893 | 0.0000000 | -1.8737136 | 0.0000000 | 0.0000000 | 0.0000000 | 0.0000000 | 0.0000000 | -1.6103974 | 0.0000000 | 0.0000000 | 0.0000000 | 0.0000000 | 0.0000000 | 0.0000000 | 0.0000000 | -1.6055293 | 0.0000000 | -1.2309862 | 0.0000000 | -1.2422750 | 43 | 4 | 291.2509 | 240.17634 |
| 2.6552919 | 0.0000000 | -2.1729455 | -2.5763341 | -52.9007919 | 0.0000000 | -3.0615551 | 0.0000000 | 0.0000000 | 0.0000000 | 0.0000000 | 0.0000000 | -0.8042261 | 0.0000000 | 0.0000000 | 0.0000000 | -0.3684975 | 0.0000000 | 0.0000000 | -1.1647105 | -2.1586604 | 0.0000000 | -1.6998488 | 0.0000000 | -1.3761538 | 43 | 5 | 225.5131 | 175.00325 |
| 2.4506114 | -0.3582762 | -2.7689748 | -4.9279407 | -12.9848781 | 0.0000000 | -2.8023896 | 0.0000000 | 0.0000000 | -0.6245728 | -0.1411804 | 0.0000000 | -1.4160731 | 0.0000000 | 0.0000000 | 0.0000000 | -0.0427115 | 0.0000000 | 0.0000000 | -1.4497600 | 0.0000000 | 0.0000000 | -2.1024625 | 0.0000000 | -0.7957463 | 43 | 6 | 170.1634 | 119.81594 |
| 0.0018111 | -1.1379338 | -0.1093767 | -1.9542041 | -0.2143701 | -0.0624953 | -0.4241615 | 0.0000000 | 0.0000000 | 0.0000000 | 0.0000000 | 0.0000000 | -0.2321024 | -0.0349733 | 0.0000000 | 0.0000000 | 0.0000000 | -0.0579797 | -0.6044518 | -0.2447996 | -0.4563914 | 0.0000000 | -0.1265794 | -0.2960759 | 0.0000000 | 44 | 1 | 484.5601 | 430.35329 |
| 0.4171692 | -1.1167721 | -0.1689943 | -1.8310533 | -11.7947658 | 0.0000000 | -1.3419605 | 0.0000000 | 0.0000000 | 0.0000000 | 0.0000000 | 0.0000000 | -0.3614473 | 0.0000000 | 0.0000000 | 0.0000000 | 0.0000000 | 0.0000000 | 0.0000000 | -0.1089650 | -1.0214506 | 0.0000000 | -0.8410124 | 0.0000000 | 0.0000000 | 44 | 2 | 452.5735 | 399.68254 |
| 1.0906751 | -0.3432177 | -2.1014581 | -1.4477607 | -10.7078260 | 0.0000000 | -1.6842497 | -0.2681773 | 0.0000000 | 0.0000000 | 0.0000000 | 0.0000000 | -0.6789966 | 0.0000000 | 0.0000000 | 0.0000000 | -0.1086616 | 0.0000000 | -0.1389979 | -0.0545768 | -1.4433609 | 0.0000000 | -0.7775707 | 0.0000000 | 0.0000000 | 44 | 3 | 372.7413 | 320.89803 |
| 2.2163374 | -0.8201502 | -1.8075480 | -3.7331910 | -14.1982359 | 0.0000000 | -2.9945735 | 0.0000000 | 0.0000000 | 0.0000000 | 0.0000000 | 0.0000000 | -2.7281310 | 0.0000000 | 0.0000000 | 0.0000000 | -0.1091861 | 0.0000000 | 0.0000000 | -0.5371181 | -1.5063836 | 0.0000000 | -1.5315805 | 0.0000000 | -1.7316915 | 44 | 4 | 235.7135 | 184.81369 |
| 3.4102302 | -1.4722288 | -2.5177632 | -2.3863840 | -36.2625386 | -0.0530897 | -2.0100227 | 0.0000000 | 0.0000000 | 0.0000000 | 0.0000000 | 0.0000000 | -1.5102517 | 0.0000000 | 0.0000000 | 0.0000000 | -0.3252610 | 0.0000000 | 0.0000000 | -2.6734686 | -2.3984153 | 0.0000000 | -1.9137823 | 0.0000000 | -2.1927936 | 44 | 5 | 197.4464 | 146.94735 |
| 3.4440672 | 0.0000000 | -4.7847343 | -1.7601214 | -14.4759142 | 0.0000000 | -4.2002669 | 0.0000000 | 0.0000000 | 0.0000000 | 0.0000000 | 0.0000000 | -1.4218018 | 0.0000000 | 0.0000000 | 0.0000000 | -1.1545981 | 0.0000000 | 0.0000000 | 0.0000000 | -4.1227804 | 0.0000000 | 0.0000000 | 0.0000000 | 0.0000000 | 44 | 6 | 164.8857 | 114.59017 |
| 0.0006172 | -1.1078782 | -0.2933904 | -1.3573308 | 0.0000000 | -0.0750024 | -0.3902219 | 0.0000000 | 0.0000000 | -0.0440054 | 0.0000000 | 0.0000000 | -0.5614158 | 0.0000000 | 0.0000000 | 0.0000000 | 0.0000000 | -0.1194549 | -0.5892351 | 0.0000000 | -0.6713931 | 0.0000000 | 0.0000000 | -0.3571140 | 0.0000000 | 45 | 1 | 507.0889 | 452.90837 |
| 0.2413051 | -0.9409434 | -0.4842830 | -1.1206553 | -14.2688652 | 0.0000000 | -1.4308711 | 0.0000000 | 0.0000000 | 0.0000000 | 0.0000000 | 0.0000000 | -0.3252155 | 0.0000000 | 0.0000000 | 0.0000000 | -0.1009993 | 0.0000000 | -0.2598891 | -0.5295235 | -0.7839592 | 0.0000000 | -0.4119150 | 0.0000000 | 0.0000000 | 45 | 2 | 475.0553 | 422.21623 |
| 1.0967096 | 0.0000000 | -2.7643622 | -0.6385353 | -18.5076971 | 0.0000000 | -3.1087026 | 0.0000000 | 0.0000000 | 0.0000000 | 0.0000000 | 0.0000000 | -0.4869283 | 0.0000000 | 0.0000000 | 0.0000000 | -0.1347868 | 0.0000000 | -0.6601990 | -1.0207768 | -0.2604616 | 0.0000000 | -0.4985154 | 0.0000000 | 0.0000000 | 45 | 3 | 389.1700 | 337.49377 |
| 0.8997316 | -0.9187556 | -1.7541317 | -2.0276852 | -10.8171796 | 0.0000000 | -2.6443338 | 0.0000000 | 0.0000000 | 0.0000000 | 0.0000000 | 0.0000000 | -0.4857270 | 0.0000000 | 0.0000000 | 0.0000000 | 0.0000000 | 0.0000000 | -0.3597760 | 0.0000000 | -2.2567735 | 0.0000000 | 0.0000000 | 0.0000000 | -0.7787024 | 45 | 4 | 325.5226 | 274.51747 |
| 1.4989901 | -2.0544102 | 0.0000000 | -5.0620978 | -11.5403946 | 0.0000000 | -3.4607903 | 0.0000000 | 0.0000000 | 0.0000000 | 0.0000000 | 0.0000000 | -2.8411783 | 0.0000000 | 0.0000000 | 0.0000000 | 0.0000000 | 0.0000000 | -0.2382977 | 0.0000000 | -0.9723020 | 0.0000000 | -1.6391194 | 0.0000000 | -0.0947669 | 45 | 5 | 209.3328 | 158.74667 |
| 2.6211160 | 0.0000000 | -4.3672161 | -3.8070927 | -21.0279957 | 0.0000000 | -1.6125807 | 0.0000000 | -0.2532344 | -0.4465153 | 0.0000000 | 0.0000000 | -4.1817174 | 0.0000000 | 0.0000000 | 0.0000000 | 0.0000000 | 0.0000000 | 0.0000000 | 0.0000000 | -2.5387154 | 0.0000000 | -1.2027444 | 0.0000000 | 0.0000000 | 45 | 6 | 142.6660 | 92.36720 |
| 0.0000000 | -1.4724341 | -0.2609257 | -1.3365155 | -5.1590541 | 0.0000000 | -0.5158789 | 0.0000000 | -0.0025143 | 0.0000000 | 0.0000000 | 0.0000000 | -0.3333826 | 0.0000000 | 0.0000000 | 0.0000000 | 0.0000000 | 0.0000000 | -0.4972228 | -0.1680364 | -0.5966102 | 0.0000000 | -0.0963379 | -0.3670156 | 0.0000000 | 46 | 1 | 487.3677 | 433.02413 |
| 0.2774704 | -0.6702160 | -0.4169926 | -1.3076254 | -18.2077416 | 0.0000000 | -1.0686205 | 0.0000000 | 0.0000000 | 0.0000000 | 0.0000000 | 0.0000000 | -0.4134642 | 0.0000000 | 0.0000000 | 0.0000000 | 0.0000000 | 0.0000000 | -0.1920661 | -0.2379587 | -0.8073523 | 0.0000000 | -0.7695561 | 0.0000000 | 0.0000000 | 46 | 2 | 456.2531 | 403.36981 |
| 0.6706522 | -1.5132572 | -0.6029109 | -1.3490830 | -18.5814709 | 0.0000000 | -1.1822002 | 0.0000000 | 0.0000000 | 0.0000000 | 0.0000000 | 0.0000000 | -0.2446060 | 0.0000000 | 0.0000000 | 0.0000000 | -0.0366817 | 0.0000000 | -0.0541403 | 0.0000000 | -1.9021153 | 0.0000000 | -0.8147500 | 0.0000000 | 0.0000000 | 46 | 3 | 438.4696 | 386.64412 |
| 1.1735828 | -0.2600004 | -1.8967437 | -1.8803955 | -23.3438422 | 0.0000000 | -2.1413722 | 0.0000000 | 0.0000000 | 0.0000000 | 0.0000000 | 0.0000000 | -0.0980292 | 0.0000000 | 0.0000000 | 0.0000000 | 0.0000000 | -0.5223942 | 0.0000000 | -1.0270307 | -1.8428989 | 0.0000000 | -0.2644454 | 0.0000000 | 0.0000000 | 46 | 4 | 307.7178 | 256.70486 |
| 1.4987510 | 0.0000000 | -1.9360084 | -6.5134583 | -9.0016645 | 0.0000000 | -2.6942293 | 0.0000000 | 0.0000000 | 0.0000000 | 0.0000000 | 0.0000000 | -1.4049610 | 0.0000000 | 0.0000000 | 0.0000000 | 0.0000000 | 0.0000000 | -0.0914760 | 0.0000000 | -1.3105330 | -0.0300868 | -1.2878960 | 0.0000000 | 0.0000000 | 46 | 5 | 180.3023 | 129.68587 |
| 3.5765287 | -0.5719782 | -1.3145201 | -5.9803231 | -56.0467161 | 0.0000000 | -3.9295180 | 0.0000000 | 0.0000000 | -0.7448393 | 0.0000000 | 0.0000000 | -2.8732275 | -1.6877886 | 0.0000000 | 0.0000000 | 0.0000000 | 0.0000000 | -0.8009362 | 0.0000000 | -3.6121360 | 0.0000000 | -0.6359705 | 0.0000000 | -6.3002404 | 46 | 6 | 143.7527 | 93.39512 |
| 0.0000000 | -1.5676664 | 0.0000000 | -1.6477376 | -0.5158029 | 0.0000000 | -0.2620977 | 0.0000000 | 0.0000000 | -0.0211712 | 0.0000000 | 0.0000000 | -0.5007132 | 0.0000000 | 0.0000000 | 0.0000000 | 0.0000000 | -0.1004927 | -0.2917445 | -0.3142530 | -0.5403585 | 0.0000000 | -0.2037684 | -0.3535978 | 0.0000000 | 47 | 1 | 457.5312 | 403.10310 |
| 0.2434181 | -0.9615939 | -0.3647090 | -1.3687754 | -8.9384232 | 0.0000000 | -0.9812942 | 0.0000000 | -0.2093110 | -0.1508020 | 0.0000000 | 0.0000000 | -0.8563017 | 0.0000000 | 0.0000000 | 0.0000000 | 0.0000000 | 0.0000000 | -0.4116988 | 0.0000000 | -0.8180169 | 0.0000000 | -0.4846606 | -0.0082406 | 0.0000000 | 47 | 2 | 446.8110 | 393.71401 |
| 1.3345936 | -0.3360770 | -2.3591918 | -0.3000235 | -20.4718186 | 0.0000000 | -1.6082817 | 0.0000000 | 0.0000000 | 0.0000000 | 0.0000000 | 0.0000000 | -1.6446019 | 0.0000000 | 0.0000000 | 0.0000000 | -0.0127011 | -0.1540266 | 0.0000000 | -0.0439684 | -1.6549022 | 0.0000000 | -0.8851535 | 0.0000000 | 0.0000000 | 47 | 3 | 373.5203 | 321.80870 |
| 1.6749103 | -0.2129556 | -2.2349213 | -1.8472360 | -8.6012724 | 0.0000000 | -3.1789288 | 0.0000000 | 0.0000000 | 0.0000000 | 0.0000000 | 0.0000000 | -2.6891209 | -0.2269953 | 0.0000000 | 0.0000000 | 0.0000000 | 0.0000000 | 0.0000000 | -0.0119497 | -1.6931878 | 0.0000000 | -0.8852147 | -0.2442122 | 0.0000000 | 47 | 4 | 275.4473 | 224.45999 |
| 1.3329332 | 0.0000000 | -1.3019930 | -2.4301394 | -38.0406204 | 0.0000000 | -3.7617930 | 0.0000000 | 0.0000000 | 0.0000000 | -0.0607149 | 0.0000000 | -3.1025782 | 0.0000000 | 0.0000000 | 0.0000000 | 0.0000000 | 0.0000000 | -0.1337614 | -0.8208352 | -0.9745732 | 0.0000000 | -0.9110848 | 0.0000000 | 0.0000000 | 47 | 5 | 214.5966 | 163.93441 |
| 2.9101991 | 0.0000000 | -3.5727759 | -4.0815952 | -34.8230642 | 0.0000000 | -2.6777459 | -0.3106409 | -0.1867307 | 0.0000000 | 0.0000000 | 0.0000000 | 0.0000000 | 0.0000000 | 0.0000000 | 0.0000000 | 0.0000000 | -0.6210115 | -1.2211906 | -0.9195826 | -2.1255762 | 0.0000000 | -0.3648417 | 0.0000000 | 0.0000000 | 47 | 6 | 172.4830 | 122.13037 |
| 0.0000000 | -1.2179292 | -0.1362459 | -1.2484790 | -0.5016071 | 0.0000000 | -0.4344570 | 0.0000000 | 0.0000000 | -0.0465917 | 0.0000000 | 0.0000000 | -0.3259510 | 0.0000000 | 0.0000000 | 0.0000000 | -0.0554420 | -0.1704335 | -0.5189594 | 0.0000000 | -0.6473018 | 0.0000000 | -0.0926605 | -0.3071293 | 0.0000000 | 48 | 1 | 488.6142 | 434.13411 |
| 0.3037521 | -1.3342433 | -0.2222539 | -1.8844631 | -9.5012024 | -0.0631876 | -0.8493407 | 0.0000000 | 0.0000000 | 0.0000000 | 0.0000000 | -0.0030313 | -0.9045841 | -0.0031909 | -0.0031143 | 0.0000000 | 0.0000000 | -0.0036610 | -0.1293428 | -0.0039839 | -1.1192052 | -0.0000059 | -0.5714721 | -0.1134004 | -0.0012809 | 48 | 2 | 429.4782 | 376.49464 |
| 1.0720744 | -0.2667250 | -1.2574970 | -1.4184739 | -27.7231425 | 0.0000000 | -1.6640711 | 0.0000000 | 0.0000000 | 0.0000000 | 0.0000000 | 0.0000000 | -0.8277702 | 0.0000000 | 0.0000000 | 0.0000000 | 0.0000000 | 0.0000000 | -0.2279969 | -0.4085159 | -1.5235029 | 0.0000000 | -1.0822005 | 0.0000000 | 0.0000000 | 48 | 3 | 366.5527 | 314.71747 |
| 1.0271521 | -0.6435406 | -0.3812290 | -2.5475647 | -18.3176434 | 0.0000000 | -2.6763720 | 0.0000000 | 0.0000000 | 0.0000000 | 0.0000000 | 0.0000000 | -2.8687761 | 0.0000000 | 0.0000000 | 0.0000000 | 0.0000000 | 0.0000000 | -0.1057977 | 0.0000000 | -1.7051967 | 0.0000000 | -0.9598038 | 0.0000000 | 0.0000000 | 48 | 4 | 272.4131 | 221.30386 |
| 3.0271516 | -1.1835962 | -1.0315337 | -3.2489326 | -21.0197388 | 0.0000000 | -3.2569675 | 0.0000000 | 0.0000000 | 0.0000000 | 0.0000000 | 0.0000000 | -2.1427404 | 0.0000000 | 0.0000000 | -0.0791240 | 0.0000000 | 0.0000000 | 0.0000000 | 0.0000000 | -2.1105992 | 0.0000000 | -2.3676120 | -0.5397166 | 0.0000000 | 48 | 5 | 206.2089 | 155.70360 |
| 2.8172927 | 0.0000000 | -4.1506253 | -0.1379229 | -45.5794458 | -0.0001087 | -4.6822465 | 0.0000000 | 0.0000000 | 0.0000000 | 0.0000000 | 0.0000000 | -1.8711621 | 0.0000000 | 0.0000000 | 0.0000000 | -0.8346286 | -1.1462237 | -0.0000009 | -0.3814257 | -2.3913119 | 0.0000000 | 0.0000000 | 0.0000000 | -1.1740597 | 48 | 6 | 161.7999 | 111.54466 |
| 0.0000000 | -1.6273923 | 0.0000000 | -0.9704948 | -7.9343625 | 0.0000000 | -0.2494548 | 0.0000000 | 0.0000000 | -0.0250215 | 0.0000000 | 0.0000000 | -0.1151953 | 0.0000000 | 0.0000000 | 0.0000000 | 0.0000000 | 0.0000000 | -0.4804667 | -0.2315234 | -0.5663657 | 0.0000000 | -0.2792571 | -0.2173344 | 0.0000000 | 49 | 1 | 476.6781 | 422.26546 |
| 0.3626689 | -0.0993746 | -0.9291360 | -0.7865649 | -16.0258583 | 0.0000000 | -0.9926074 | 0.0000000 | 0.0000000 | 0.0000000 | 0.0000000 | 0.0000000 | -1.3406393 | 0.0000000 | 0.0000000 | 0.0000000 | 0.0000000 | -0.1214261 | -0.2810959 | 0.0000000 | -0.7144327 | 0.0000000 | -0.7489174 | 0.0000000 | 0.0000000 | 49 | 2 | 428.5347 | 375.55660 |
| 1.1025549 | -0.5705323 | -1.0260572 | -3.0865827 | -10.9975593 | 0.0000000 | -1.5283810 | 0.0000000 | 0.0000000 | -0.1081123 | 0.0000000 | 0.0000000 | 0.0000000 | 0.0000000 | 0.0000000 | 0.0000000 | 0.0000000 | 0.0000000 | -0.0632040 | -0.4303966 | -2.0422633 | 0.0000000 | -0.5924621 | -0.5617234 | 0.0000000 | 49 | 3 | 360.8951 | 309.33922 |
| 1.9917175 | -0.4978222 | -3.4142098 | 0.0000000 | -34.7462386 | 0.0000000 | -3.5677509 | 0.0000000 | 0.0000000 | 0.0000000 | 0.0000000 | 0.0000000 | -1.0976330 | 0.0000000 | 0.0000000 | 0.0000000 | 0.0000000 | -0.0670246 | -0.2196893 | -0.9895840 | -0.9154397 | 0.0000000 | -1.0676928 | 0.0000000 | 0.0000000 | 49 | 4 | 295.5666 | 244.71331 |
| 2.1330462 | -0.2777187 | -1.4212903 | -2.8338655 | -27.2877822 | 0.0000000 | -3.8823637 | 0.0000000 | 0.0000000 | 0.0000000 | 0.0000000 | 0.0000000 | -1.0335569 | 0.0000000 | 0.0000000 | 0.0000000 | 0.0000000 | 0.0000000 | 0.0000000 | 0.0000000 | -2.4601129 | 0.0000000 | -1.2990524 | 0.0000000 | 0.0000000 | 49 | 5 | 214.4071 | 163.82841 |
| 3.5015521 | 0.0000000 | -2.8900759 | -5.1389169 | -21.2322606 | 0.0000000 | -2.8737356 | 0.0000000 | 0.0000000 | 0.0000000 | 0.0000000 | 0.0000000 | -3.5718228 | 0.0000000 | 0.0000000 | 0.0000000 | 0.0000000 | 0.0000000 | -0.9855249 | -0.8099899 | -1.6378053 | 0.0000000 | -2.2594177 | 0.0000000 | 0.0000000 | 49 | 6 | 144.3234 | 93.96014 |
| 0.0022336 | -1.8100560 | -0.1249397 | -0.8201926 | -7.7110339 | 0.0000000 | -0.2966832 | 0.0000000 | -0.0526487 | 0.0000000 | 0.0000000 | 0.0000000 | -0.1775269 | 0.0000000 | 0.0000000 | 0.0000000 | 0.0000000 | -0.0433175 | -0.3635596 | -0.0971984 | -0.5269599 | 0.0000000 | -0.1261366 | -0.4615243 | 0.0000000 | 50 | 1 | 494.4275 | 440.08363 |
| 0.5629019 | -0.9368239 | -0.6822771 | -1.0699575 | -15.6686266 | 0.0000000 | -0.7317640 | 0.0000000 | 0.0000000 | 0.0000000 | 0.0000000 | 0.0000000 | -0.7165772 | 0.0000000 | 0.0000000 | 0.0000000 | -0.0422454 | -0.4080218 | -0.1207048 | 0.0000000 | -1.1347998 | 0.0000000 | -0.5507803 | 0.0000000 | 0.0000000 | 50 | 2 | 504.3451 | 451.43127 |
| 0.7263616 | -0.4510940 | -2.3385176 | -0.2174349 | -15.2791964 | 0.0000000 | -1.5874975 | 0.0000000 | 0.0000000 | 0.0000000 | 0.0000000 | 0.0000000 | -1.4234525 | 0.0000000 | 0.0000000 | 0.0000000 | -0.1382330 | 0.0000000 | -0.3194418 | -0.1848288 | -1.0786621 | 0.0000000 | -0.4939577 | 0.0000000 | 0.0000000 | 50 | 3 | 400.0995 | 348.22111 |
| 2.3759474 | 0.0000000 | -1.6761439 | -2.6223850 | -25.2912813 | 0.0000000 | -2.5014955 | 0.0000000 | 0.0000000 | 0.0000000 | 0.0000000 | 0.0000000 | -1.6071400 | 0.0000000 | 0.0000000 | 0.0000000 | -0.0500351 | 0.0000000 | 0.0000000 | -0.9363337 | -1.3907858 | 0.0000000 | -2.2484303 | 0.0000000 | 0.0000000 | 50 | 4 | 249.8470 | 198.90686 |
| 3.8160864 | 0.0000000 | -3.1223593 | -1.7183036 | -27.8724007 | 0.0000000 | -2.3997702 | 0.0000000 | 0.0000000 | -0.1290157 | -0.2144534 | 0.0000000 | -2.1189788 | 0.0000000 | 0.0000000 | 0.0000000 | 0.0000000 | 0.0000000 | 0.0000000 | 0.0000000 | -3.3451122 | 0.0000000 | -2.3560953 | 0.0000000 | 0.0000000 | 50 | 5 | 196.6372 | 146.06974 |
| 4.6048918 | 0.0000000 | -3.1642717 | -6.8220462 | -15.1573294 | 0.0000000 | -3.4996171 | 0.0000000 | 0.0000000 | 0.0000000 | 0.0000000 | 0.0000000 | -3.5565279 | 0.0000000 | 0.0000000 | 0.0000000 | 0.0000000 | 0.0000000 | 0.0000000 | -2.7246937 | -1.1010940 | 0.0000000 | -2.9951608 | 0.0000000 | 0.0000000 | 50 | 6 | 150.7902 | 100.44074 |
