# Supplementary material for: Tropical Land‐Use Change Disrupts Zeta‐Diversity Across Taxa
Source: Glob Chang Biol. 2025 May 14;31(5):e70245. doi: 10.1111/gcb.70245 (PMC12076182; doi:10.1111/gcb.70245)
Supplement: Supplementary file 1 — Data S1. [file GCB-31-e70245-s001.zip › gcb70245-sup-0001-Appendix1.zip!/SM-5-DB_pasture.html]

table output


MS-GDM Coefficients for dung beetle communities in transformed habitats from 50 iterations (Binomial family (link=log) for each order of zeta and each predictor, using a random sample of 1000 site combinations for each MS-GDM. Here are the 50 replicates for each predictor, AIC, and deviance.

| (Intercept) | Elevation1 | Elevation2 | Elevation3 | Canopy.cover1 | Canopy.cover2 | Canopy.cover3 | Tree.density1 | Tree.density2 | Tree.density3 | Fragmentation1 | Fragmentation2 | Fragmentation3 | Forest.cover1 | Forest.cover2 | Forest.cover3 | Precipitation1 | Precipitation2 | Precipitation3 | Disturbance1 | Disturbance2 | Disturbance3 | distance1 | distance2 | distance3 | Rep | Group | AIC | Residual\_Deviance |
| --- | --- | --- | --- | --- | --- | --- | --- | --- | --- | --- | --- | --- | --- | --- | --- | --- | --- | --- | --- | --- | --- | --- | --- | --- | --- | --- | --- | --- |
| 0.0000000 | 0.0000000 | -1.7451860 | -0.1389066 | -0.1507626 | 0.0000000 | 0.0000000 | -0.0680219 | 0.0000000 | 0.0000000 | -0.0770315 | 0.0000000 | 0.0000000 | -0.4415964 | -0.6181796 | 0.0000000 | 0.0000000 | -0.1475463 | -0.0889961 | 0.0000000 | 0.0000000 | 0.0000000 | 0.0000000 | -1.1845198 | 0.000000e+00 | 1 | 1 | 712.05751 | 660.5389024 |
| 0.6278688 | -0.1161500 | -2.4434996 | 0.0000000 | 0.0000000 | 0.0000000 | 0.0000000 | -0.4055827 | 0.0000000 | 0.0000000 | -0.4878952 | 0.0000000 | -0.1765512 | -0.9049285 | -0.7642049 | 0.0000000 | 0.0000000 | 0.0000000 | 0.0000000 | 0.0000000 | 0.0000000 | 0.0000000 | -0.9940464 | -0.7505410 | 0.000000e+00 | 1 | 2 | 416.01038 | 365.5374216 |
| 1.7739563 | -1.4603177 | -3.7144359 | -0.2819771 | -0.4931913 | -0.1542961 | 0.0000000 | -0.3588819 | 0.0000000 | 0.0000000 | -0.0663374 | 0.0000000 | 0.0000000 | -2.9662260 | -0.2470020 | 0.0000000 | 0.0000000 | -0.7140965 | -1.2351907 | 0.0000000 | 0.0000000 | 0.0000000 | -1.0118332 | -0.3271692 | 0.000000e+00 | 1 | 3 | 224.82098 | 174.6707561 |
| 3.4190697 | -2.9802805 | -2.4106881 | -0.2673329 | 0.0000000 | 0.0000000 | 0.0000000 | 0.0000000 | 0.0000000 | 0.0000000 | -2.9159870 | 0.0000000 | -0.0542691 | -0.2257153 | -3.8695784 | 0.0000000 | 0.0000000 | -2.2562028 | 0.0000000 | 0.0000000 | 0.0000000 | 0.0000000 | -1.8967352 | 0.0000000 | 0.000000e+00 | 1 | 4 | 118.57956 | 68.5427632 |
| 912.5159874 | 0.0000000 | 0.0000000 | -4867.6322997 | -501.8302400 | 0.0000000 | 0.0000000 | 0.0000000 | 0.0000000 | 0.0000000 | 0.0000000 | 0.0000000 | 0.0000000 | -2053.2697074 | 0.0000000 | 0.0000000 | 0.0000000 | -630.6433507 | 0.0000000 | -568.2792511 | 0.0000000 | 0.0000000 | -256.6821519 | 0.0000000 | 0.000000e+00 | 1 | 5 | 50.01003 | 0.0100333 |
| 47.5727476 | -57.2937508 | 0.0000000 | -27.4658327 | 0.0000000 | 0.0000000 | -26.3303462 | -30.4894694 | 0.0000000 | -27.6619235 | 0.0000000 | 0.0000000 | 0.0000000 | 0.0000000 | 0.0000000 | 0.0000000 | 0.0000000 | 0.0000000 | 0.0000000 | -21.4848275 | 0.0000000 | -474.0759539 | 0.0000000 | 0.0000000 | -2.687779e+05 | 1 | 6 | 51.86001 | 1.8526491 |
| 0.0000000 | 0.0000000 | -1.7451860 | -0.1389066 | -0.1507626 | 0.0000000 | 0.0000000 | -0.0680219 | 0.0000000 | 0.0000000 | -0.0770315 | 0.0000000 | 0.0000000 | -0.4415964 | -0.6181796 | 0.0000000 | 0.0000000 | -0.1475463 | -0.0889961 | 0.0000000 | 0.0000000 | 0.0000000 | 0.0000000 | -1.1845198 | 0.000000e+00 | 2 | 1 | 712.05751 | 660.5389024 |
| 0.3688978 | -0.3681695 | -2.0496909 | -0.2887742 | -0.1999959 | 0.0000000 | 0.0000000 | 0.0000000 | 0.0000000 | 0.0000000 | -0.2080065 | 0.0000000 | 0.0000000 | -0.6215143 | -0.6268589 | 0.0000000 | 0.0000000 | -0.4012870 | -0.3396620 | 0.0000000 | 0.0000000 | 0.0000000 | -0.6995436 | -0.8479182 | 0.000000e+00 | 2 | 2 | 426.06070 | 375.5941113 |
| 1.1240311 | -1.0375081 | -2.3040642 | -2.0415472 | -0.6643169 | 0.0000000 | 0.0000000 | -0.0831773 | -0.0501212 | 0.0000000 | 0.0000000 | 0.0000000 | -0.5520332 | -1.4121203 | 0.0000000 | 0.0000000 | -0.2102188 | 0.0000000 | 0.0000000 | 0.0000000 | 0.0000000 | 0.0000000 | -1.5947481 | -1.7117176 | 0.000000e+00 | 2 | 3 | 214.55841 | 164.4185752 |
| 1.3073064 | -1.5443835 | -1.3180335 | -0.1641271 | 0.0000000 | 0.0000000 | 0.0000000 | -0.9639266 | 0.0000000 | 0.0000000 | 0.0000000 | 0.0000000 | 0.0000000 | -5.5881357 | 0.0000000 | 0.0000000 | 0.0000000 | 0.0000000 | 0.0000000 | 0.0000000 | -2.5650619 | 0.0000000 | -0.9067204 | -2.6939724 | 0.000000e+00 | 2 | 4 | 115.14132 | 65.0824381 |
| 5.2459419 | -0.0638883 | -1.1291349 | -4.9090695 | 0.0000000 | -0.4681669 | -0.1528240 | 0.0000000 | 0.0000000 | 0.0000000 | -6.2485403 | 0.0000000 | 0.0000000 | -0.3855527 | 0.0000000 | 0.0000000 | 0.0000000 | 0.0000000 | 0.0000000 | 0.0000000 | -9.5995633 | 0.0000000 | -0.0969072 | -6.0189034 | -1.220863e+05 | 2 | 5 | 75.35001 | 25.3500071 |
| 1.2929118 | -42.0209007 | 0.0000000 | 0.0000000 | 0.0000000 | 0.0000000 | 0.0000000 | -4.0028963 | 0.0000000 | 0.0000000 | 0.0000000 | 0.0000000 | -0.9558781 | -16.0324970 | 0.0000000 | 0.0000000 | 0.0000000 | 0.0000000 | 0.0000000 | 0.0000000 | 0.0000000 | 0.0000000 | 0.0000000 | 0.0000000 | 0.000000e+00 | 2 | 6 | 54.42555 | 4.4181944 |
| 0.0000000 | 0.0000000 | -1.7451860 | -0.1389066 | -0.1507626 | 0.0000000 | 0.0000000 | -0.0680219 | 0.0000000 | 0.0000000 | -0.0770315 | 0.0000000 | 0.0000000 | -0.4415964 | -0.6181796 | 0.0000000 | 0.0000000 | -0.1475463 | -0.0889961 | 0.0000000 | 0.0000000 | 0.0000000 | 0.0000000 | -1.1845198 | 0.000000e+00 | 3 | 1 | 712.05751 | 660.5389024 |
| 0.5811889 | -0.5437222 | -1.1314381 | -0.8390059 | -0.3248848 | 0.0000000 | 0.0000000 | -0.0118870 | 0.0000000 | 0.0000000 | -0.0000005 | -0.0000024 | -0.0000018 | -0.4606836 | -0.7644337 | 0.0000000 | 0.0000000 | -0.0000547 | -0.0731371 | -0.0000105 | -0.0000256 | -0.0000216 | -1.9900444 | -0.0000326 | 0.000000e+00 | 3 | 2 | 404.51894 | 353.9434325 |
| 1.3519278 | -2.0172606 | -3.4162219 | -2.4201701 | -0.2715466 | 0.0000000 | 0.0000000 | 0.0000000 | 0.0000000 | 0.0000000 | -0.5213045 | 0.0000000 | 0.0000000 | -4.7656921 | 0.0000000 | 0.0000000 | 0.0000000 | -1.7952531 | -1.3786460 | 0.0000000 | 0.0000000 | -11.7201884 | -0.0915045 | -0.9470944 | 0.000000e+00 | 3 | 3 | 174.89923 | 124.8643263 |
| 380.6291635 | -34.3579666 | -129.9394959 | 0.0000000 | -83.5196011 | 0.0000000 | 0.0000000 | -24.7140875 | -54.9161676 | -17.6493276 | -121.4019139 | 0.0000000 | -3.4035943 | -10.1926504 | -88.7455199 | -7.9741817 | -0.0000266 | -6.7248275 | -16.8089969 | -249.7542106 | -139.7936618 | -0.0016294 | -7.7549804 | -218.0396388 | -3.981328e+02 | 3 | 4 | 50.00104 | 0.0010364 |
| 0.8569984 | -6.7833850 | 0.0000000 | -3.1607869 | -1.2871745 | 0.0000000 | 0.0000000 | -1.6896115 | 0.0000000 | 0.0000000 | -1.5346335 | 0.0000000 | -0.4382996 | -3.3286948 | 0.0000000 | 0.0000000 | 0.0000000 | -0.0553332 | -1.4538628 | 0.0000000 | 0.0000000 | -7.3124498 | 0.0000000 | -0.7190685 | -1.041183e+00 | 3 | 5 | 69.74658 | 19.7318630 |
| 0.0000000 | 0.0000000 | -1.7451860 | -0.1389066 | -0.1507626 | 0.0000000 | 0.0000000 | -0.0680219 | 0.0000000 | 0.0000000 | -0.0770315 | 0.0000000 | 0.0000000 | -0.4415964 | -0.6181796 | 0.0000000 | 0.0000000 | -0.1475463 | -0.0889961 | 0.0000000 | 0.0000000 | 0.0000000 | 0.0000000 | -1.1845198 | 0.000000e+00 | 4 | 1 | 712.05751 | 660.5389024 |
| 0.3602866 | -1.0621629 | -0.6716458 | -1.0114451 | 0.0000000 | 0.0000000 | 0.0000000 | 0.0000000 | 0.0000000 | 0.0000000 | 0.0000000 | 0.0000000 | -0.0618396 | -1.0149405 | -0.7970598 | 0.0000000 | 0.0000000 | -1.0229454 | 0.0000000 | 0.0000000 | 0.0000000 | 0.0000000 | -0.6703351 | -1.0470285 | 0.000000e+00 | 4 | 2 | 412.77414 | 362.2992757 |
| 0.5602805 | -1.2042398 | -0.9456276 | -0.5800564 | 0.0000000 | 0.0000000 | 0.0000000 | 0.0000000 | 0.0000000 | 0.0000000 | -0.2538872 | 0.0000000 | 0.0000000 | -2.6813092 | -0.9932507 | 0.0000000 | 0.0000000 | -0.4930277 | -0.7948917 | 0.0000000 | -0.2137844 | 0.0000000 | -0.8030291 | -2.2399015 | 0.000000e+00 | 4 | 3 | 206.41233 | 156.2875041 |
| 1.1058057 | -3.4587997 | -1.2282402 | -1.2230911 | 0.0000000 | 0.0000000 | 0.0000000 | 0.0000000 | 0.0000000 | 0.0000000 | 0.0000000 | 0.0000000 | 0.0000000 | -3.6912455 | 0.0000000 | 0.0000000 | 0.0000000 | -1.6953826 | -1.6679557 | 0.0000000 | -2.5609391 | -2.1006812 | 0.0000000 | -1.3577902 | -1.534401e+00 | 4 | 4 | 110.26564 | 60.2214780 |
| 5.7940830 | -2.2758950 | -2.0727694 | -3.5682004 | 0.0000000 | 0.0000000 | 0.0000000 | 0.0000000 | 0.0000000 | 0.0000000 | 0.0000000 | 0.0000000 | -0.0800049 | -4.7266677 | 0.0000000 | 0.0000000 | -3.7002747 | -0.4193077 | -1.0109735 | 0.0000000 | 0.0000000 | -17.3057756 | -2.8961033 | -5.1925669 | 0.000000e+00 | 4 | 5 | 83.97464 | 33.9525560 |
| 0.9651390 | -6.4985379 | 0.0000000 | -20.3753528 | -1.6238710 | 0.0000000 | 0.0000000 | 0.0000000 | 0.0000000 | 0.0000000 | 0.0000000 | 0.0000000 | 0.0000000 | 0.0000000 | 0.0000000 | 0.0000000 | 0.0000000 | -4.4666801 | 0.0000000 | 0.0000000 | 0.0000000 | 0.0000000 | 0.0000000 | 0.0000000 | 0.000000e+00 | 4 | 6 | 61.48401 | 11.4840142 |
| 0.0000000 | 0.0000000 | -1.7451860 | -0.1389066 | -0.1507626 | 0.0000000 | 0.0000000 | -0.0680219 | 0.0000000 | 0.0000000 | -0.0770315 | 0.0000000 | 0.0000000 | -0.4415964 | -0.6181796 | 0.0000000 | 0.0000000 | -0.1475463 | -0.0889961 | 0.0000000 | 0.0000000 | 0.0000000 | 0.0000000 | -1.1845198 | 0.000000e+00 | 5 | 1 | 712.05751 | 660.5389024 |
| 0.8459862 | -0.4626112 | -1.9596925 | 0.0000000 | -0.1951105 | 0.0000000 | 0.0000000 | -0.4260279 | 0.0000000 | 0.0000000 | -0.1231984 | 0.0000000 | 0.0000000 | -1.0495540 | -0.9412398 | 0.0000000 | 0.0000000 | 0.0000000 | -0.1635360 | 0.0000000 | 0.0000000 | 0.0000000 | -1.2706664 | -0.5605753 | 0.000000e+00 | 5 | 2 | 427.89186 | 377.4561200 |
| 1.8523738 | -1.8645193 | -1.0131792 | -2.6038020 | 0.0000000 | 0.0000000 | 0.0000000 | -0.7122368 | 0.0000000 | 0.0000000 | -1.1835073 | 0.0000000 | 0.0000000 | -0.9439670 | 0.0000000 | 0.0000000 | 0.0000000 | -0.0208450 | 0.0000000 | 0.0000000 | 0.0000000 | 0.0000000 | -2.9805552 | -0.5471349 | 0.000000e+00 | 5 | 3 | 231.99276 | 181.8327593 |
| 0.2365264 | -2.1329935 | -2.5185668 | -2.8009208 | 0.0000000 | 0.0000000 | 0.0000000 | 0.0000000 | 0.0000000 | 0.0000000 | -0.2938287 | 0.0000000 | 0.0000000 | 0.0000000 | 0.0000000 | 0.0000000 | 0.0000000 | 0.0000000 | 0.0000000 | 0.0000000 | 0.0000000 | -2.0779768 | -3.8829870 | 0.0000000 | 0.000000e+00 | 5 | 4 | 79.87367 | 29.8442289 |
| 6.3672622 | -13.1689133 | 0.0000000 | -8.7238692 | 0.0000000 | -2.0662088 | 0.0000000 | 0.0000000 | 0.0000000 | 0.0000000 | -5.9792126 | 0.0000000 | 0.0000000 | 0.0000000 | 0.0000000 | -0.1758007 | 0.0000000 | -3.4528246 | 0.0000000 | 0.0000000 | -7.7891050 | 0.0000000 | -1.5840162 | 0.0000000 | 0.000000e+00 | 5 | 5 | 59.08994 | 9.0825784 |
| 41.3171847 | 0.0000000 | -35.1968438 | 0.0000000 | 0.0000000 | 0.0000000 | -4.5754761 | -11.2531688 | 0.0000000 | -9.4646996 | 0.0000000 | 0.0000000 | -2.6341447 | -3.1382582 | 0.0000000 | 0.0000000 | 0.0000000 | 0.0000000 | -10.3736644 | 0.0000000 | 0.0000000 | -5.3954101 | -29.4579057 | 0.0000000 | 0.000000e+00 | 5 | 6 | 50.00003 | 0.0000284 |
| 0.0000000 | 0.0000000 | -1.7451860 | -0.1389066 | -0.1507626 | 0.0000000 | 0.0000000 | -0.0680219 | 0.0000000 | 0.0000000 | -0.0770315 | 0.0000000 | 0.0000000 | -0.4415964 | -0.6181796 | 0.0000000 | 0.0000000 | -0.1475463 | -0.0889961 | 0.0000000 | 0.0000000 | 0.0000000 | 0.0000000 | -1.1845198 | 0.000000e+00 | 6 | 1 | 712.05751 | 660.5389024 |
| 0.1269571 | -0.3521834 | -2.0988223 | -0.2823031 | -0.1240990 | 0.0000000 | 0.0000000 | -0.1494757 | 0.0000000 | 0.0000000 | 0.0000000 | 0.0000000 | 0.0000000 | -1.1975489 | -0.3032383 | 0.0000000 | 0.0000000 | 0.0000000 | 0.0000000 | 0.0000000 | 0.0000000 | 0.0000000 | -0.4192537 | -1.5409189 | 0.000000e+00 | 6 | 2 | 433.35431 | 382.8495528 |
| 1.1638986 | -3.1619895 | -0.3610006 | -1.4994087 | -0.2104232 | 0.0000000 | 0.0000000 | 0.0000000 | 0.0000000 | 0.0000000 | -0.7611639 | 0.0000000 | 0.0000000 | 0.0000000 | -1.0456559 | 0.0000000 | 0.0000000 | -1.3290757 | 0.0000000 | 0.0000000 | 0.0000000 | -1.5511356 | -2.0870718 | 0.0000000 | 0.000000e+00 | 6 | 3 | 220.89644 | 170.7673897 |
| 3.0724847 | -2.1381784 | 0.0000000 | -0.3711864 | -1.7653593 | 0.0000000 | 0.0000000 | 0.0000000 | 0.0000000 | 0.0000000 | -0.2111786 | 0.0000000 | 0.0000000 | -4.8275524 | 0.0000000 | -1.2774888 | -1.3260815 | -1.3659163 | 0.0000000 | -2.5973541 | -1.9176931 | 0.0000000 | -0.7684242 | -2.5382049 | 0.000000e+00 | 6 | 4 | 96.89745 | 46.8827306 |
| 1.7790735 | -10.4352527 | 0.0000000 | -4.3945206 | 0.0000000 | 0.0000000 | 0.0000000 | 0.0000000 | 0.0000000 | 0.0000000 | 0.0000000 | 0.0000000 | 0.0000000 | -1.4165259 | 0.0000000 | 0.0000000 | 0.0000000 | 0.0000000 | 0.0000000 | 0.0000000 | 0.0000000 | -3.7303347 | -3.4003738 | -1.4520426 | -5.054750e-02 | 6 | 5 | 88.55275 | 38.5306718 |
| 0.0000000 | 0.0000000 | -1.7451860 | -0.1389066 | -0.1507626 | 0.0000000 | 0.0000000 | -0.0680219 | 0.0000000 | 0.0000000 | -0.0770315 | 0.0000000 | 0.0000000 | -0.4415964 | -0.6181796 | 0.0000000 | 0.0000000 | -0.1475463 | -0.0889961 | 0.0000000 | 0.0000000 | 0.0000000 | 0.0000000 | -1.1845198 | 0.000000e+00 | 7 | 1 | 712.05751 | 660.5389024 |
| 0.3813713 | -0.2372756 | -2.1711018 | -1.4545303 | -0.3893372 | 0.0000000 | 0.0000000 | 0.0000000 | 0.0000000 | 0.0000000 | -0.0006827 | 0.0000000 | 0.0000000 | -0.4735022 | -0.4177563 | 0.0000000 | 0.0000000 | -0.0980530 | 0.0000000 | 0.0000000 | 0.0000000 | 0.0000000 | -0.7389870 | -1.6347199 | 0.000000e+00 | 7 | 2 | 379.72881 | 329.3456081 |
| 0.7831839 | -0.6916489 | -0.3958122 | -3.3382715 | -0.3804543 | 0.0000000 | 0.0000000 | -0.2132485 | 0.0000000 | 0.0000000 | 0.0000000 | 0.0000000 | -0.2650604 | -1.0557506 | 0.0000000 | 0.0000000 | 0.0000000 | -1.7831815 | 0.0000000 | 0.0000000 | 0.0000000 | -0.8604377 | -1.5427278 | -0.4440483 | 0.000000e+00 | 7 | 3 | 241.07954 | 190.8875713 |
| 1.4042340 | -2.7093985 | 0.0000000 | -2.8569939 | 0.0000000 | 0.0000000 | 0.0000000 | -0.9770229 | 0.0000000 | 0.0000000 | -2.6805262 | 0.0000000 | 0.0000000 | -2.6203505 | 0.0000000 | 0.0000000 | 0.0000000 | -0.5722731 | 0.0000000 | 0.0000000 | 0.0000000 | -5.1578472 | -1.9098809 | -0.8766969 | 0.000000e+00 | 7 | 4 | 115.38372 | 65.3616397 |
| 1.4367355 | -8.6152190 | 0.0000000 | -3.7009573 | -1.2332273 | 0.0000000 | 0.0000000 | 0.0000000 | 0.0000000 | 0.0000000 | 0.0000000 | 0.0000000 | 0.0000000 | 0.0000000 | 0.0000000 | 0.0000000 | 0.0000000 | -2.2841774 | -1.1306558 | 0.0000000 | -3.2035665 | -3.9426784 | 0.0000000 | -0.8320531 | 0.000000e+00 | 7 | 5 | 75.15815 | 25.1507853 |
| 0.0000000 | 0.0000000 | -1.7451860 | -0.1389066 | -0.1507626 | 0.0000000 | 0.0000000 | -0.0680219 | 0.0000000 | 0.0000000 | -0.0770315 | 0.0000000 | 0.0000000 | -0.4415964 | -0.6181796 | 0.0000000 | 0.0000000 | -0.1475463 | -0.0889961 | 0.0000000 | 0.0000000 | 0.0000000 | 0.0000000 | -1.1845198 | 0.000000e+00 | 8 | 1 | 712.05751 | 660.5389024 |
| 0.3438261 | -1.0159341 | -0.9051716 | -0.3958937 | -0.2012068 | 0.0000000 | 0.0000000 | -0.3624496 | 0.0000000 | 0.0000000 | -0.0233635 | 0.0000000 | 0.0000000 | -2.6254832 | 0.0000000 | 0.0000000 | 0.0000000 | -0.2345184 | -0.7194853 | 0.0000000 | 0.0000000 | 0.0000000 | -0.7711690 | -0.9896662 | 0.000000e+00 | 8 | 2 | 375.07306 | 324.6682708 |
| 0.6463320 | -6.5398400 | 0.0000000 | -2.5117859 | 0.0000000 | 0.0000000 | 0.0000000 | -0.2716966 | 0.0000000 | 0.0000000 | -0.4923510 | 0.0000000 | 0.0000000 | -2.6559854 | 0.0000000 | 0.0000000 | 0.0000000 | 0.0000000 | -1.2805227 | 0.0000000 | 0.0000000 | -0.4560201 | -0.7081349 | -1.8117843 | 0.000000e+00 | 8 | 3 | 198.34513 | 148.2494509 |
| 3.0540524 | -1.4272643 | -4.7729375 | 0.0000000 | 0.0000000 | 0.0000000 | -0.6327976 | 0.0000000 | 0.0000000 | 0.0000000 | -0.2487203 | 0.0000000 | -2.1802294 | -1.8547677 | 0.0000000 | 0.0000000 | -0.0050975 | 0.0000000 | 0.0000000 | 0.0000000 | 0.0000000 | -20.9508264 | -1.8770567 | -4.1513859 | 0.000000e+00 | 8 | 4 | 96.72926 | 46.6703816 |
| 0.0000000 | 0.0000000 | -1.7451860 | -0.1389066 | -0.1507626 | 0.0000000 | 0.0000000 | -0.0680219 | 0.0000000 | 0.0000000 | -0.0770315 | 0.0000000 | 0.0000000 | -0.4415964 | -0.6181796 | 0.0000000 | 0.0000000 | -0.1475463 | -0.0889961 | 0.0000000 | 0.0000000 | 0.0000000 | 0.0000000 | -1.1845198 | 0.000000e+00 | 9 | 1 | 712.05751 | 660.5389024 |
| 0.2074381 | -0.0895055 | -1.0802964 | -0.0443765 | -0.2373473 | 0.0000000 | 0.0000000 | -0.1625984 | 0.0000000 | 0.0000000 | -0.0393402 | 0.0000000 | -0.2058789 | -1.7226359 | -0.4443001 | 0.0000000 | 0.0000000 | -0.0316566 | -0.2897083 | 0.0000000 | 0.0000000 | 0.0000000 | -1.0631041 | -0.8812466 | 0.000000e+00 | 9 | 2 | 412.97524 | 362.4603971 |
| 0.6885469 | -1.3292139 | -0.4669076 | -2.2115177 | 0.0000000 | 0.0000000 | -0.1606054 | -0.0015431 | 0.0000000 | 0.0000000 | 0.0000000 | -0.0004425 | -0.0391148 | -2.4125638 | -0.0153868 | 0.0000000 | 0.0000000 | -0.5247989 | -1.3583934 | -0.0004586 | -0.0011446 | -0.0038066 | -1.5961743 | -1.1829177 | 0.000000e+00 | 9 | 3 | 222.21419 | 172.0890740 |
| 4.5647900 | -1.6911893 | -2.1123629 | -6.1594635 | -1.5793351 | 0.0000000 | -0.0924109 | -1.7113364 | 0.0000000 | 0.0000000 | 0.0000000 | 0.0000000 | 0.0000000 | -2.8813659 | 0.0000000 | 0.0000000 | 0.0000000 | -1.7399893 | -1.0756938 | 0.0000000 | 0.0000000 | 0.0000000 | -3.0912337 | 0.0000000 | -2.227088e+00 | 9 | 4 | 98.67539 | 48.6680328 |
| 0.0000000 | 0.0000000 | -1.7451860 | -0.1389066 | -0.1507626 | 0.0000000 | 0.0000000 | -0.0680219 | 0.0000000 | 0.0000000 | -0.0770315 | 0.0000000 | 0.0000000 | -0.4415964 | -0.6181796 | 0.0000000 | 0.0000000 | -0.1475463 | -0.0889961 | 0.0000000 | 0.0000000 | 0.0000000 | 0.0000000 | -1.1845198 | 0.000000e+00 | 10 | 1 | 712.05751 | 660.5389024 |
| 0.7259580 | 0.0000000 | -1.9500615 | 0.0000000 | -0.1054950 | 0.0000000 | 0.0000000 | -0.0983854 | 0.0000000 | 0.0000000 | -0.4398937 | 0.0000000 | 0.0000000 | -1.7753817 | -0.4147048 | 0.0000000 | 0.0000000 | -0.0427400 | -0.1520482 | 0.0000000 | 0.0000000 | 0.0000000 | -1.6737531 | -0.6794537 | 0.000000e+00 | 10 | 2 | 390.97175 | 340.5342717 |
| 0.0568137 | -3.1410263 | -0.0115833 | -3.6950361 | -0.3494971 | 0.0000000 | 0.0000000 | -0.5002050 | 0.0000000 | 0.0000000 | 0.0000000 | 0.0000000 | 0.0000000 | -0.4859881 | 0.0000000 | 0.0000000 | 0.0000000 | 0.0000000 | 0.0000000 | 0.0000000 | 0.0000000 | -0.4077184 | -1.0546739 | -1.8483022 | 0.000000e+00 | 10 | 3 | 202.16427 | 152.0278268 |
| 2.8329523 | -6.6861263 | 0.0000000 | -2.4155012 | -1.7391867 | 0.0000000 | 0.0000000 | -1.8444280 | 0.0000000 | 0.0000000 | -1.3992231 | 0.0000000 | 0.0000000 | -7.5549704 | 0.0000000 | 0.0000000 | 0.0000000 | -0.3661225 | -3.1427298 | 0.0000000 | 0.0000000 | -13.4729952 | 0.0000000 | -0.8405036 | 0.000000e+00 | 10 | 4 | 85.87086 | 35.8487791 |
| 5.0712770 | -28.1757675 | 0.0000000 | -3.3826659 | 0.0000000 | 0.0000000 | 0.0000000 | -3.0281534 | 0.0000000 | 0.0000000 | -0.0882728 | 0.0000000 | 0.0000000 | -7.2007096 | 0.0000000 | 0.0000000 | 0.0000000 | -1.5091913 | 0.0000000 | 0.0000000 | 0.0000000 | -52.4062774 | -1.6450559 | 0.0000000 | -1.014811e+01 | 10 | 5 | 67.55307 | 17.5383523 |
| 24.1842648 | -17.1289138 | 0.0000000 | -14.7076820 | 0.0000000 | 0.0000000 | 0.0000000 | 0.0000000 | 0.0000000 | 0.0000000 | -9.8043685 | 0.0000000 | 0.0000000 | -41.1054411 | -7.5993860 | 0.0000000 | 0.0000000 | -6.1055560 | 0.0000000 | 0.0000000 | 0.0000000 | 0.0000000 | -11.3249659 | -3.0023966 | 0.000000e+00 | 10 | 6 | 59.21757 | 9.2102102 |
| 0.0000000 | 0.0000000 | -1.7451860 | -0.1389066 | -0.1507626 | 0.0000000 | 0.0000000 | -0.0680219 | 0.0000000 | 0.0000000 | -0.0770315 | 0.0000000 | 0.0000000 | -0.4415964 | -0.6181796 | 0.0000000 | 0.0000000 | -0.1475463 | -0.0889961 | 0.0000000 | 0.0000000 | 0.0000000 | 0.0000000 | -1.1845198 | 0.000000e+00 | 11 | 1 | 712.05751 | 660.5389024 |
| 0.3566307 | -0.2249492 | -2.4978864 | -0.0750160 | 0.0000000 | 0.0000000 | 0.0000000 | 0.0000000 | 0.0000000 | 0.0000000 | -0.5203561 | 0.0000000 | 0.0000000 | -1.3907675 | 0.0000000 | 0.0000000 | 0.0000000 | 0.0000000 | -0.7132457 | 0.0000000 | 0.0000000 | 0.0000000 | -1.1362217 | -0.6214728 | 0.000000e+00 | 11 | 2 | 379.04664 | 328.6255231 |
| 0.0374224 | -1.4186031 | 0.0000000 | -1.7447351 | -0.4049376 | 0.0000000 | 0.0000000 | -0.2744149 | 0.0000000 | 0.0000000 | 0.0000000 | 0.0000000 | 0.0000000 | -4.4453668 | 0.0000000 | 0.0000000 | 0.0000000 | -1.2814999 | 0.0000000 | 0.0000000 | 0.0000000 | -0.7350034 | -1.3116499 | -0.2491412 | 0.000000e+00 | 11 | 3 | 184.38537 | 134.2257966 |
| 1.8548104 | -4.3061044 | 0.0000000 | -5.0376381 | 0.0000000 | 0.0000000 | 0.0000000 | -1.8979356 | 0.0000000 | 0.0000000 | 0.0000000 | 0.0000000 | 0.0000000 | -5.2345963 | -0.8555120 | 0.0000000 | 0.0000000 | -3.7656481 | -0.1550414 | 0.0000000 | 0.0000000 | 0.0000000 | 0.0000000 | 0.0000000 | -3.048764e+00 | 11 | 4 | 108.01706 | 57.9876223 |
| 0.0000000 | -13.0929908 | 0.0000000 | -6.3354933 | 0.0000000 | 0.0000000 | 0.0000000 | 0.0000000 | 0.0000000 | 0.0000000 | 0.0000000 | 0.0000000 | 0.0000000 | -52.5416719 | 0.0000000 | 0.0000000 | 0.0000000 | -0.1507944 | -2.4300795 | 0.0000000 | 0.0000000 | 0.0000000 | -1.1646992 | 0.0000000 | 0.000000e+00 | 11 | 5 | 60.35069 | 10.3506854 |
| 0.0000000 | 0.0000000 | -1.7451860 | -0.1389066 | -0.1507626 | 0.0000000 | 0.0000000 | -0.0680219 | 0.0000000 | 0.0000000 | -0.0770315 | 0.0000000 | 0.0000000 | -0.4415964 | -0.6181796 | 0.0000000 | 0.0000000 | -0.1475463 | -0.0889961 | 0.0000000 | 0.0000000 | 0.0000000 | 0.0000000 | -1.1845198 | 0.000000e+00 | 12 | 1 | 712.05751 | 660.5389024 |
| 0.4159814 | -0.8605534 | -1.3023253 | -0.6011359 | 0.0000000 | 0.0000000 | 0.0000000 | 0.0000000 | 0.0000000 | 0.0000000 | -0.2811521 | 0.0000000 | 0.0000000 | -0.4244033 | -0.8313495 | 0.0000000 | 0.0000000 | -0.3077487 | -0.4636436 | 0.0000000 | 0.0000000 | 0.0000000 | -0.7798237 | -1.2314038 | 0.000000e+00 | 12 | 2 | 415.67726 | 365.1708800 |
| 0.8278974 | -2.7843562 | -0.2783936 | -0.7938754 | -0.2457108 | 0.0000000 | 0.0000000 | 0.0000000 | 0.0000000 | 0.0000000 | 0.0000000 | 0.0000000 | 0.0000000 | -2.9648334 | 0.0000000 | 0.0000000 | 0.0000000 | -0.0534791 | 0.0000000 | 0.0000000 | 0.0000000 | 0.0000000 | -3.2290055 | -0.1560012 | -1.547401e-01 | 12 | 3 | 216.44566 | 166.3300760 |
| 0.0000000 | -3.7324505 | -1.3452993 | -1.0750143 | 0.0000000 | 0.0000000 | 0.0000000 | -1.3412911 | 0.0000000 | 0.0000000 | 0.0000000 | 0.0000000 | 0.0000000 | -0.9988472 | 0.0000000 | 0.0000000 | 0.0000000 | -0.2509712 | 0.0000000 | 0.0000000 | 0.0000000 | -4.5714280 | -1.7142718 | -0.0821998 | 0.000000e+00 | 12 | 4 | 102.20706 | 52.1408219 |
| 3.2277843 | -13.8655796 | 0.0000000 | -1.3975111 | 0.0000000 | 0.0000000 | 0.0000000 | -2.9798451 | 0.0000000 | 0.0000000 | 0.0000000 | 0.0000000 | 0.0000000 | -7.8125340 | 0.0000000 | 0.0000000 | 0.0000000 | -1.1155880 | 0.0000000 | 0.0000000 | -1.8994205 | -4.7056274 | 0.0000000 | -2.7444046 | 0.000000e+00 | 12 | 5 | 76.13635 | 26.1142716 |
| 0.0000000 | 0.0000000 | -1.7451860 | -0.1389066 | -0.1507626 | 0.0000000 | 0.0000000 | -0.0680219 | 0.0000000 | 0.0000000 | -0.0770315 | 0.0000000 | 0.0000000 | -0.4415964 | -0.6181796 | 0.0000000 | 0.0000000 | -0.1475463 | -0.0889961 | 0.0000000 | 0.0000000 | 0.0000000 | 0.0000000 | -1.1845198 | 0.000000e+00 | 13 | 1 | 712.05751 | 660.5389024 |
| 0.1451860 | -0.2003693 | -2.0488690 | 0.0000000 | -0.1439275 | 0.0000000 | 0.0000000 | -0.0161467 | 0.0000000 | 0.0000000 | 0.0000000 | 0.0000000 | -0.2062217 | -1.5491092 | -0.1580787 | 0.0000000 | 0.0000000 | -0.8169931 | 0.0000000 | 0.0000000 | 0.0000000 | 0.0000000 | -0.2455597 | -1.4273762 | 0.000000e+00 | 13 | 2 | 405.67818 | 355.2198338 |
| 0.1927735 | -2.2433906 | 0.0000000 | -2.6077864 | -0.0877956 | 0.0000000 | 0.0000000 | -0.6704771 | 0.0000000 | 0.0000000 | 0.0000000 | 0.0000000 | -0.0586347 | -1.7551471 | 0.0000000 | 0.0000000 | 0.0000000 | -0.2805394 | 0.0000000 | 0.0000000 | 0.0000000 | -2.4777100 | -1.9025633 | -0.5301074 | 0.000000e+00 | 13 | 3 | 196.20760 | 146.0830055 |
| 0.0000000 | -0.5124500 | -4.0388177 | -1.4598649 | -0.6608593 | 0.0000000 | 0.0000000 | 0.0000000 | 0.0000000 | 0.0000000 | -0.8395945 | 0.0000000 | 0.0000000 | -1.0063235 | 0.0000000 | 0.0000000 | 0.0000000 | -0.5584317 | 0.0000000 | 0.0000000 | 0.0000000 | -2.0910669 | 0.0000000 | -1.7344661 | -6.008248e+00 | 13 | 4 | 104.40044 | 54.3710007 |
| 0.0000000 | -29.4812254 | 0.0000000 | 0.0000000 | 0.0000000 | 0.0000000 | 0.0000000 | 0.0000000 | 0.0000000 | -1.2955088 | -2.3232989 | 0.0000000 | 0.0000000 | -37.9914675 | 0.0000000 | 0.0000000 | 0.0000000 | 0.0000000 | -0.1393260 | 0.0000000 | 0.0000000 | 0.0000000 | -0.6568079 | 0.0000000 | 0.000000e+00 | 13 | 5 | 60.67692 | 10.6769150 |
| 6.9979043 | -75.3899068 | 0.0000000 | -6.9207111 | 0.0000000 | 0.0000000 | 0.0000000 | -2.3596615 | 0.0000000 | 0.0000000 | 0.0000000 | 0.0000000 | -1.9407695 | -3.3630697 | 0.0000000 | 0.0000000 | 0.0000000 | 0.0000000 | 0.0000000 | 0.0000000 | 0.0000000 | -5.7822772 | 0.0000000 | -6.3627974 | 0.000000e+00 | 13 | 6 | 60.89096 | 10.8836013 |
| 0.0000000 | 0.0000000 | -1.7451860 | -0.1389066 | -0.1507626 | 0.0000000 | 0.0000000 | -0.0680219 | 0.0000000 | 0.0000000 | -0.0770315 | 0.0000000 | 0.0000000 | -0.4415964 | -0.6181796 | 0.0000000 | 0.0000000 | -0.1475463 | -0.0889961 | 0.0000000 | 0.0000000 | 0.0000000 | 0.0000000 | -1.1845198 | 0.000000e+00 | 14 | 1 | 712.05751 | 660.5389024 |
| 0.4106041 | -0.2329565 | -1.7532643 | -0.5426896 | -0.1391053 | 0.0000000 | 0.0000000 | -0.0091390 | 0.0000000 | 0.0000000 | 0.0000000 | 0.0000000 | 0.0000000 | -0.6722370 | -0.6429314 | 0.0000000 | 0.0000000 | -0.4697834 | -0.6635586 | 0.0000000 | 0.0000000 | 0.0000000 | -0.7341879 | -0.8030908 | 0.000000e+00 | 14 | 2 | 433.33430 | 382.8285097 |
| 1.3822029 | -3.3627291 | -1.1897097 | -0.4713858 | -0.0000005 | 0.0000000 | 0.0000000 | -0.3913093 | 0.0000000 | 0.0000000 | -1.2750900 | -0.0000003 | 0.0000000 | -2.1031636 | -0.9184093 | 0.0000000 | 0.0000000 | -0.8019348 | 0.0000000 | 0.0000000 | -0.0000004 | -2.3897656 | -1.3173850 | -0.7601532 | 0.000000e+00 | 14 | 3 | 205.30375 | 155.2007097 |
| 0.0000000 | -2.5858782 | -0.0892118 | -14.2040796 | 0.0000000 | 0.0000000 | 0.0000000 | 0.0000000 | 0.0000000 | 0.0000000 | 0.0000000 | 0.0000000 | 0.0000000 | -0.9093128 | -0.2795863 | 0.0000000 | 0.0000000 | -0.4769574 | -1.4861532 | 0.0000000 | 0.0000000 | 0.0000000 | -0.8141863 | -2.0501983 | 0.000000e+00 | 14 | 4 | 103.38520 | 53.3704777 |
| 1.4853009 | -9.0644667 | 0.0000000 | -28.5058781 | -2.6778221 | 0.0000000 | 0.0000000 | -3.0380349 | 0.0000000 | 0.0000000 | 0.0000000 | 0.0000000 | 0.0000000 | 0.0000000 | 0.0000000 | 0.0000000 | 0.0000000 | 0.0000000 | 0.0000000 | 0.0000000 | 0.0000000 | 0.0000000 | 0.0000000 | 0.0000000 | -2.084062e+01 | 14 | 5 | 57.68785 | 7.6731352 |
| 4.3559439 | -41.1212074 | 0.0000000 | -43.5287759 | -3.0646915 | 0.0000000 | 0.0000000 | 0.0000000 | 0.0000000 | 0.0000000 | -1.7505717 | 0.0000000 | 0.0000000 | 0.0000000 | 0.0000000 | 0.0000000 | -4.2068733 | 0.0000000 | 0.0000000 | 0.0000000 | 0.0000000 | 0.0000000 | 0.0000000 | 0.0000000 | 0.000000e+00 | 14 | 6 | 58.89917 | 8.8991716 |
| 0.0000000 | 0.0000000 | -1.7451860 | -0.1389066 | -0.1507626 | 0.0000000 | 0.0000000 | -0.0680219 | 0.0000000 | 0.0000000 | -0.0770315 | 0.0000000 | 0.0000000 | -0.4415964 | -0.6181796 | 0.0000000 | 0.0000000 | -0.1475463 | -0.0889961 | 0.0000000 | 0.0000000 | 0.0000000 | 0.0000000 | -1.1845198 | 0.000000e+00 | 15 | 1 | 712.05751 | 660.5389024 |
| 0.1406731 | -0.6352265 | -0.9322688 | -0.3286516 | -0.0486798 | 0.0000000 | 0.0000000 | 0.0000000 | 0.0000000 | 0.0000000 | -0.2260608 | 0.0000000 | 0.0000000 | -0.6701473 | -1.3940293 | 0.0000000 | 0.0000000 | -0.3960265 | 0.0000000 | 0.0000000 | 0.0000000 | 0.0000000 | -1.0267468 | -0.2852562 | 0.000000e+00 | 15 | 2 | 432.63495 | 382.2264099 |
| 2.5554085 | -2.1007740 | -3.3727720 | -0.4263375 | -1.5798577 | 0.0000000 | 0.0000000 | -0.3629099 | 0.0000000 | 0.0000000 | 0.0000000 | 0.0000000 | 0.0000000 | 0.0000000 | -2.9521232 | 0.0000000 | 0.0000000 | -0.2860626 | 0.0000000 | 0.0000000 | 0.0000000 | 0.0000000 | -1.7643247 | -0.7431401 | 0.000000e+00 | 15 | 3 | 173.44630 | 123.3391398 |
| 3.4847200 | -1.0250530 | 0.0000000 | -7.7565640 | -1.3333881 | -0.9949343 | 0.0000000 | -1.1041519 | 0.0000000 | 0.0000000 | 0.0000000 | -0.3412165 | 0.0000000 | 0.0000000 | -0.4414794 | 0.0000000 | 0.0000000 | 0.0000000 | -0.5447892 | 0.0000000 | 0.0000000 | -25.1196972 | -3.0358311 | -2.4647623 | 0.000000e+00 | 15 | 4 | 78.11761 | 28.0808141 |
| 11.8471764 | -20.1457233 | 0.0000000 | 0.0000000 | -16.6034867 | 0.0000000 | 0.0000000 | 0.0000000 | 0.0000000 | 0.0000000 | -0.5119146 | 0.0000000 | -50.6598384 | -30.3564706 | 0.0000000 | 0.0000000 | 0.0000000 | 0.0000000 | 0.0000000 | 0.0000000 | 0.0000000 | 0.0000000 | 0.0000000 | 0.0000000 | -4.899253e+02 | 15 | 5 | 55.29127 | 5.2839103 |
| 6.0033231 | -24.7255925 | 0.0000000 | -9.9646472 | 0.0000000 | 0.0000000 | 0.0000000 | 0.0000000 | 0.0000000 | 0.0000000 | -0.2695829 | 0.0000000 | 0.0000000 | -0.7109086 | 0.0000000 | 0.0000000 | -4.7456169 | 0.0000000 | 0.0000000 | 0.0000000 | 0.0000000 | -0.3419758 | 0.0000000 | -17.6283218 | -5.967387e+03 | 15 | 6 | 52.57891 | 2.5715542 |
| 0.0000000 | 0.0000000 | -1.7451860 | -0.1389066 | -0.1507626 | 0.0000000 | 0.0000000 | -0.0680219 | 0.0000000 | 0.0000000 | -0.0770315 | 0.0000000 | 0.0000000 | -0.4415964 | -0.6181796 | 0.0000000 | 0.0000000 | -0.1475463 | -0.0889961 | 0.0000000 | 0.0000000 | 0.0000000 | 0.0000000 | -1.1845198 | 0.000000e+00 | 16 | 1 | 712.05751 | 660.5389024 |
| 0.2356259 | -0.5958671 | -1.2571759 | 0.0000000 | -0.2665489 | 0.0000000 | 0.0000000 | -0.1980775 | 0.0000000 | 0.0000000 | 0.0000000 | 0.0000000 | 0.0000000 | -1.7117459 | -0.3661272 | 0.0000000 | 0.0000000 | 0.0000000 | -0.3701996 | 0.0000000 | 0.0000000 | 0.0000000 | -0.6852079 | -1.1828535 | 0.000000e+00 | 16 | 2 | 436.55876 | 386.1532259 |
| 0.5217273 | -0.7867111 | -1.6709134 | 0.0000000 | 0.0000000 | 0.0000000 | 0.0000000 | 0.0000000 | 0.0000000 | 0.0000000 | 0.0000000 | 0.0000000 | 0.0000000 | -2.6157362 | -0.2788262 | 0.0000000 | 0.0000000 | 0.0000000 | -0.3970650 | 0.0000000 | 0.0000000 | 0.0000000 | -2.7077701 | -0.3726187 | 0.000000e+00 | 16 | 3 | 198.31508 | 148.1567259 |
| 2.5763313 | -1.7788314 | -1.9491896 | -1.2117471 | 0.0000000 | 0.0000000 | 0.0000000 | -0.5371517 | 0.0000000 | 0.0000000 | 0.0000000 | 0.0000000 | 0.0000000 | -3.5346553 | 0.0000000 | 0.0000000 | 0.0000000 | 0.0000000 | 0.0000000 | 0.0000000 | 0.0000000 | -4.8840221 | -4.8520408 | -0.0597696 | -1.112884e+01 | 16 | 4 | 101.11239 | 51.0776818 |
| 0.0000000 | -3.4126916 | 0.0000000 | -3.8280885 | 0.0000000 | 0.0000000 | 0.0000000 | -2.5183597 | 0.0000000 | 0.0000000 | -0.9519572 | 0.0000000 | 0.0000000 | -4.5760942 | 0.0000000 | 0.0000000 | -0.3824783 | 0.0000000 | 0.0000000 | -5.6144577 | 0.0000000 | 0.0000000 | -0.1775529 | 0.0000000 | -9.747206e+02 | 16 | 5 | 59.02195 | 9.0072316 |
| 15.3743555 | -25.6235868 | 0.0000000 | -5.7922697 | -3.8783611 | 0.0000000 | 0.0000000 | 0.0000000 | 0.0000000 | -17.7135536 | -9.9882590 | 0.0000000 | -3.4801897 | 0.0000000 | 0.0000000 | 0.0000000 | 0.0000000 | 0.0000000 | 0.0000000 | 0.0000000 | -8.1783167 | -3.2054193 | 0.0000000 | -10.0256718 | -5.653159e+03 | 16 | 6 | 52.72658 | 2.7192176 |
| 0.0000000 | 0.0000000 | -1.7451860 | -0.1389066 | -0.1507626 | 0.0000000 | 0.0000000 | -0.0680219 | 0.0000000 | 0.0000000 | -0.0770315 | 0.0000000 | 0.0000000 | -0.4415964 | -0.6181796 | 0.0000000 | 0.0000000 | -0.1475463 | -0.0889961 | 0.0000000 | 0.0000000 | 0.0000000 | 0.0000000 | -1.1845198 | 0.000000e+00 | 17 | 1 | 712.05751 | 660.5389024 |
| 0.3815052 | -0.2753757 | -1.2199685 | 0.0000000 | -0.4962067 | 0.0000000 | 0.0000000 | -0.2666082 | 0.0000000 | -0.0714233 | -0.0722957 | 0.0000000 | 0.0000000 | -1.7786718 | 0.0000000 | 0.0000000 | 0.0000000 | -0.0736803 | 0.0000000 | 0.0000000 | 0.0000000 | 0.0000000 | -1.6411101 | 0.0000000 | 0.000000e+00 | 17 | 2 | 433.05198 | 382.4890238 |
| 2.6209583 | -0.8411750 | -1.9841007 | -0.1519669 | -0.5573875 | 0.0000000 | 0.0000000 | -0.9006105 | 0.0000000 | 0.0000000 | 0.0000000 | 0.0000000 | 0.0000000 | -1.7131998 | -1.5816196 | 0.0000000 | 0.0000000 | -0.4476377 | -0.3845497 | 0.0000000 | 0.0000000 | 0.0000000 | -2.9779614 | 0.0000000 | 0.000000e+00 | 17 | 3 | 205.92819 | 155.7317923 |
| 0.0000000 | -4.1242326 | 0.0000000 | -2.6070215 | 0.0000000 | 0.0000000 | 0.0000000 | -1.1900244 | 0.0000000 | 0.0000000 | -1.3202140 | 0.0000000 | 0.0000000 | -3.4499508 | 0.0000000 | 0.0000000 | 0.0000000 | -1.1127361 | 0.0000000 | 0.0000000 | 0.0000000 | -2.7619532 | -0.5873185 | -0.2580526 | -2.611105e+00 | 17 | 4 | 98.38777 | 48.3509666 |
| 1.4535802 | -6.4972445 | 0.0000000 | 0.0000000 | 0.0000000 | 0.0000000 | 0.0000000 | -1.9982124 | 0.0000000 | -3.0867554 | 0.0000000 | 0.0000000 | 0.0000000 | -15.0068187 | 0.0000000 | 0.0000000 | 0.0000000 | 0.0000000 | 0.0000000 | 0.0000000 | -4.9327838 | -14.2972091 | 0.0000000 | 0.0000000 | -2.402877e+01 | 17 | 5 | 61.80176 | 11.7944026 |
| 0.0000000 | 0.0000000 | -1.7451860 | -0.1389066 | -0.1507626 | 0.0000000 | 0.0000000 | -0.0680219 | 0.0000000 | 0.0000000 | -0.0770315 | 0.0000000 | 0.0000000 | -0.4415964 | -0.6181796 | 0.0000000 | 0.0000000 | -0.1475463 | -0.0889961 | 0.0000000 | 0.0000000 | 0.0000000 | 0.0000000 | -1.1845198 | 0.000000e+00 | 18 | 1 | 712.05751 | 660.5389024 |
| 0.0716940 | -0.2589842 | -1.7819185 | -0.3312865 | -0.1900572 | 0.0000000 | 0.0000000 | -0.0000444 | 0.0000000 | 0.0000000 | 0.0000000 | -0.0004974 | 0.0000000 | -1.4808708 | -0.2515779 | 0.0000000 | 0.0000000 | -0.1273864 | -0.0001644 | -0.0013720 | -0.0042656 | -0.0037002 | -0.7331804 | -0.9082486 | 0.000000e+00 | 18 | 2 | 428.34720 | 377.8910192 |
| 0.4722192 | -2.0957038 | -1.0830515 | -1.8342328 | -0.1207809 | 0.0000000 | 0.0000000 | 0.0000000 | 0.0000000 | 0.0000000 | 0.0000000 | 0.0000000 | -0.3626662 | -3.0174539 | 0.0000000 | 0.0000000 | 0.0000000 | -2.1804824 | 0.0000000 | 0.0000000 | 0.0000000 | 0.0000000 | -0.0361755 | -1.8495551 | 0.000000e+00 | 18 | 3 | 213.43388 | 163.3048013 |
| 2.9463873 | -2.6030527 | -2.5577409 | -1.1493232 | -0.0683172 | 0.0000000 | 0.0000000 | 0.0000000 | 0.0000000 | 0.0000000 | -2.6308417 | 0.0000000 | 0.0000000 | -2.2735505 | 0.0000000 | 0.0000000 | 0.0000000 | 0.0000000 | -1.9368274 | 0.0000000 | 0.0000000 | 0.0000000 | -2.4472024 | -1.8247878 | 0.000000e+00 | 18 | 4 | 124.22386 | 74.1723396 |
| 7.7861368 | -1.4316661 | -4.0466295 | -0.7545682 | -0.3405464 | 0.0000000 | -1.8678481 | -0.6518528 | 0.0000000 | 0.0000000 | 0.0000000 | 0.0000000 | 0.0000000 | -4.3742737 | 0.0000000 | 0.0000000 | 0.0000000 | -0.8087138 | 0.0000000 | 0.0000000 | 0.0000000 | -3.5187401 | -7.7813447 | 0.0000000 | -5.744657e+00 | 18 | 5 | 93.93805 | 43.9380515 |
| 208.2596611 | -2.1620255 | 0.0000000 | -46.0997197 | -19.2680606 | 0.0000000 | -37.6284868 | -3.9230887 | 0.0000000 | 0.0000000 | -4.2219303 | 0.0000000 | 0.0000000 | -57.9634446 | 0.0000000 | 0.0000000 | -40.0018963 | 0.0000000 | -97.4546888 | 0.0000000 | 0.0000000 | -1116.6598289 | -84.2494819 | -151.2177388 | 0.000000e+00 | 18 | 6 | 50.00736 | 0.0000006 |
| 0.0000000 | 0.0000000 | -1.7451860 | -0.1389066 | -0.1507626 | 0.0000000 | 0.0000000 | -0.0680219 | 0.0000000 | 0.0000000 | -0.0770315 | 0.0000000 | 0.0000000 | -0.4415964 | -0.6181796 | 0.0000000 | 0.0000000 | -0.1475463 | -0.0889961 | 0.0000000 | 0.0000000 | 0.0000000 | 0.0000000 | -1.1845198 | 0.000000e+00 | 19 | 1 | 712.05751 | 660.5389024 |
| 0.2184598 | -0.3287568 | -1.8512369 | -0.0101485 | -0.3163477 | 0.0000000 | 0.0000000 | -0.0000006 | -0.0000001 | 0.0000000 | -0.4994176 | 0.0000000 | 0.0000000 | -1.4196086 | -0.5396530 | 0.0000000 | 0.0000000 | -0.1968064 | 0.0000000 | -0.0000001 | -0.0000002 | -0.0256105 | -0.3099422 | -1.7825706 | -1.000000e-07 | 19 | 2 | 384.52014 | 334.0856131 |
| 0.6000935 | -5.1381032 | 0.0000000 | -1.6136046 | -0.4061731 | 0.0000000 | 0.0000000 | -0.1375931 | 0.0000000 | 0.0000000 | -0.9563975 | 0.0000000 | -0.0002762 | -4.7430022 | 0.0000000 | 0.0000000 | 0.0000000 | -0.1881074 | 0.0000000 | 0.0000000 | 0.0000000 | -3.8048161 | -0.5483994 | -1.9907126 | 0.000000e+00 | 19 | 3 | 189.05805 | 138.9355676 |
| 2.2218144 | -2.4777377 | -0.8902556 | -2.0894186 | 0.0000000 | 0.0000000 | 0.0000000 | 0.0000000 | 0.0000000 | 0.0000000 | 0.0000000 | 0.0000000 | 0.0000000 | -3.1905517 | 0.0000000 | 0.0000000 | 0.0000000 | -0.3700326 | 0.0000000 | 0.0000000 | 0.0000000 | -0.9225665 | -4.8300223 | 0.0000000 | 0.000000e+00 | 19 | 4 | 127.63274 | 77.5815069 |
| 2.2229042 | -13.1054770 | 0.0000000 | -2.5167427 | 0.0000000 | 0.0000000 | 0.0000000 | 0.0000000 | 0.0000000 | 0.0000000 | -2.0327450 | 0.0000000 | 0.0000000 | -1.2947198 | 0.0000000 | 0.0000000 | 0.0000000 | 0.0000000 | 0.0000000 | 0.0000000 | 0.0000000 | -5.8694959 | -4.3644663 | 0.0000000 | -2.711450e+00 | 19 | 5 | 76.40370 | 26.3889835 |
| 0.0000000 | 0.0000000 | -1.7451860 | -0.1389066 | -0.1507626 | 0.0000000 | 0.0000000 | -0.0680219 | 0.0000000 | 0.0000000 | -0.0770315 | 0.0000000 | 0.0000000 | -0.4415964 | -0.6181796 | 0.0000000 | 0.0000000 | -0.1475463 | -0.0889961 | 0.0000000 | 0.0000000 | 0.0000000 | 0.0000000 | -1.1845198 | 0.000000e+00 | 20 | 1 | 712.05751 | 660.5389024 |
| 0.3027889 | 0.0000000 | -3.9431893 | 0.0000000 | -0.1915531 | 0.0000000 | 0.0000000 | 0.0000000 | 0.0000000 | 0.0000000 | 0.0000000 | -0.0416002 | -0.3754059 | -1.1327000 | 0.0000000 | 0.0000000 | 0.0000000 | -0.1842850 | -0.4189967 | 0.0000000 | 0.0000000 | 0.0000000 | -0.2982458 | -1.3097634 | 0.000000e+00 | 20 | 2 | 366.26452 | 315.9020958 |
| 1.3075955 | -3.7931414 | 0.0000000 | -1.7609786 | 0.0000000 | 0.0000000 | 0.0000000 | 0.0000000 | 0.0000000 | 0.0000000 | -1.5257441 | 0.0000000 | 0.0000000 | -3.8583608 | 0.0000000 | 0.0000000 | 0.0000000 | -1.4013011 | 0.0000000 | 0.0000000 | -1.1641960 | -8.8270200 | -0.1809531 | -1.4247661 | 0.000000e+00 | 20 | 3 | 254.76223 | 204.6308448 |
| 2.8626845 | -6.8122787 | 0.0000000 | -6.7437313 | -1.6484032 | 0.0000000 | 0.0000000 | 0.0000000 | 0.0000000 | 0.0000000 | -1.2811965 | 0.0000000 | 0.0000000 | -5.6439496 | 0.0000000 | 0.0000000 | 0.0000000 | -3.3160926 | 0.0000000 | 0.0000000 | 0.0000000 | -0.7731663 | -0.7692502 | 0.0000000 | -7.647785e+00 | 20 | 4 | 87.07448 | 37.0229612 |
| 215.3901810 | -746.6748437 | 0.0000000 | -7.3604128 | 0.0000000 | 0.0000000 | 0.0000000 | -103.0580421 | 0.0000000 | 0.0000000 | 0.0000000 | 0.0000000 | -232.5102834 | -489.9644815 | 0.0000000 | 0.0000000 | -97.4453571 | 0.0000000 | -18.0248547 | 0.0000000 | 0.0000000 | -198.7282493 | 0.0000000 | -116.2237342 | -1.933630e+03 | 20 | 5 | 50.00736 | 0.0000029 |
| 0.0000000 | 0.0000000 | -1.7451860 | -0.1389066 | -0.1507626 | 0.0000000 | 0.0000000 | -0.0680219 | 0.0000000 | 0.0000000 | -0.0770315 | 0.0000000 | 0.0000000 | -0.4415964 | -0.6181796 | 0.0000000 | 0.0000000 | -0.1475463 | -0.0889961 | 0.0000000 | 0.0000000 | 0.0000000 | 0.0000000 | -1.1845198 | 0.000000e+00 | 21 | 1 | 712.05751 | 660.5389024 |
| 0.4335359 | -0.4419488 | -1.3611398 | 0.0000000 | -0.4982067 | 0.0000000 | 0.0000000 | -0.0746084 | 0.0000000 | 0.0000000 | -0.2961951 | 0.0000000 | 0.0000000 | -1.7248218 | -0.3540885 | 0.0000000 | 0.0000000 | 0.0000000 | -0.2541203 | 0.0000000 | 0.0000000 | 0.0000000 | -1.5017707 | 0.0000000 | 0.000000e+00 | 21 | 2 | 415.86766 | 365.3491777 |
| 1.3238287 | 0.0000000 | -4.5080443 | 0.0000000 | 0.0000000 | 0.0000000 | 0.0000000 | 0.0000000 | 0.0000000 | 0.0000000 | -0.0475246 | 0.0000000 | 0.0000000 | -1.1815165 | -0.5785794 | 0.0000000 | -0.3432511 | -0.2998418 | 0.0000000 | 0.0000000 | 0.0000000 | 0.0000000 | -2.4210189 | -0.3824016 | 0.000000e+00 | 21 | 3 | 218.20697 | 168.0818516 |
| 0.2107338 | -8.8769901 | 0.0000000 | -0.6014529 | 0.0000000 | 0.0000000 | 0.0000000 | 0.0000000 | 0.0000000 | 0.0000000 | 0.0000000 | 0.0000000 | 0.0000000 | -5.5080485 | 0.0000000 | 0.0000000 | 0.0000000 | 0.0000000 | 0.0000000 | 0.0000000 | 0.0000000 | -4.4106839 | -2.3728689 | -0.7755044 | -2.208101e+01 | 21 | 4 | 82.13508 | 32.1162905 |
| 858.6105565 | -2467.1414377 | 0.0000000 | -246.3503464 | -258.7055486 | 0.0000000 | -14.7718940 | -259.6107144 | 0.0000000 | 0.0000000 | 0.0000000 | 0.0000000 | 0.0000000 | -435.7280317 | 0.0000000 | 0.0000000 | 0.0000000 | 0.0000000 | -273.5802352 | 0.0000000 | 0.0000000 | -150.4089316 | -145.7026879 | -147.2876112 | 0.000000e+00 | 21 | 6 | 50.01473 | 0.0000111 |
| 0.0000000 | 0.0000000 | -1.7451860 | -0.1389066 | -0.1507626 | 0.0000000 | 0.0000000 | -0.0680219 | 0.0000000 | 0.0000000 | -0.0770315 | 0.0000000 | 0.0000000 | -0.4415964 | -0.6181796 | 0.0000000 | 0.0000000 | -0.1475463 | -0.0889961 | 0.0000000 | 0.0000000 | 0.0000000 | 0.0000000 | -1.1845198 | 0.000000e+00 | 22 | 1 | 712.05751 | 660.5389024 |
| 0.5685328 | -0.0701730 | -1.7749185 | -0.2139185 | -0.1681937 | 0.0000000 | 0.0000000 | -0.2137215 | 0.0000000 | 0.0000000 | 0.0000000 | -0.0003536 | -0.0011989 | -0.3727806 | -0.6373044 | 0.0000000 | 0.0000000 | -0.0021243 | -0.0830883 | -0.0008400 | -0.0013996 | -0.2578160 | -1.4437862 | -0.6323528 | 0.000000e+00 | 22 | 2 | 455.32924 | 404.8298619 |
| 1.3989799 | -2.0686922 | -0.9183874 | -0.8888160 | -0.4579855 | 0.0000000 | 0.0000000 | -0.8327509 | 0.0000000 | 0.0000000 | 0.0000000 | 0.0000000 | 0.0000000 | -5.2728306 | 0.0000000 | 0.0000000 | 0.0000000 | -0.2278961 | -0.3055056 | 0.0000000 | 0.0000000 | -4.1213265 | -2.0014638 | -0.3127896 | 0.000000e+00 | 22 | 3 | 233.67989 | 183.5525376 |
| 4.5106538 | 0.0000000 | -9.6312935 | -1.9577183 | 0.0000000 | 0.0000000 | 0.0000000 | 0.0000000 | 0.0000000 | 0.0000000 | -4.0002195 | 0.0000000 | 0.0000000 | 0.0000000 | 0.0000000 | 0.0000000 | -1.2597746 | -3.2593910 | 0.0000000 | 0.0000000 | 0.0000000 | -4.6002280 | -0.4981461 | -2.3450948 | -4.154801e+00 | 22 | 4 | 71.84109 | 21.8263702 |
| 33.8534547 | -17.9939584 | -14.3942059 | -14.2439182 | -11.9172168 | 0.0000000 | 0.0000000 | 0.0000000 | 0.0000000 | 0.0000000 | -2.3462057 | -1.3101629 | 0.0000000 | -0.2735335 | 0.0000000 | 0.0000000 | 0.0000000 | -7.5694340 | 0.0000000 | 0.0000000 | 0.0000000 | -1.5390158 | -16.2014826 | 0.0000000 | -1.301022e+04 | 22 | 5 | 60.96596 | 10.9659608 |
| 1.9583840 | -15.1029667 | 0.0000000 | -7.7413716 | 0.0000000 | 0.0000000 | 0.0000000 | -6.7843026 | 0.0000000 | 0.0000000 | 0.0000000 | 0.0000000 | 0.0000000 | -1.9507630 | 0.0000000 | 0.0000000 | 0.0000000 | 0.0000000 | 0.0000000 | 0.0000000 | 0.0000000 | -6.0390417 | 0.0000000 | 0.0000000 | 0.000000e+00 | 22 | 6 | 56.72643 | 6.7117149 |
| 0.0000000 | 0.0000000 | -1.7451860 | -0.1389066 | -0.1507626 | 0.0000000 | 0.0000000 | -0.0680219 | 0.0000000 | 0.0000000 | -0.0770315 | 0.0000000 | 0.0000000 | -0.4415964 | -0.6181796 | 0.0000000 | 0.0000000 | -0.1475463 | -0.0889961 | 0.0000000 | 0.0000000 | 0.0000000 | 0.0000000 | -1.1845198 | 0.000000e+00 | 23 | 1 | 712.05751 | 660.5389024 |
| 0.1638254 | -0.9258222 | -1.5491592 | -1.3366715 | -0.1704907 | 0.0000000 | 0.0000000 | -0.0114106 | 0.0000000 | 0.0000000 | 0.0000000 | 0.0000000 | -0.5936871 | -1.6030194 | -0.0012259 | 0.0000000 | 0.0000000 | -0.6652530 | -0.6801703 | -0.0007221 | 0.0000000 | -0.0004509 | -0.5077367 | -0.4656820 | 0.000000e+00 | 23 | 2 | 390.44171 | 340.0595893 |
| 2.6681698 | -1.7219743 | -3.1805754 | -3.1667883 | 0.0000000 | 0.0000000 | 0.0000000 | -1.1714819 | 0.0000000 | -0.6383578 | -2.5941007 | 0.0000000 | -2.2643798 | 0.0000000 | 0.0000000 | 0.0000000 | 0.0000000 | -0.9817102 | -1.2290400 | 0.0000000 | -0.5469030 | -1.1617845 | 0.0000000 | -2.4230525 | 0.000000e+00 | 23 | 3 | 165.38190 | 115.3037461 |
| 5.1954939 | -5.3799665 | 0.0000000 | -5.1447139 | 0.0000000 | 0.0000000 | 0.0000000 | -2.2733543 | 0.0000000 | 0.0000000 | -2.6072505 | 0.0000000 | 0.0000000 | 0.0000000 | -2.0333971 | 0.0000000 | 0.0000000 | -0.6269236 | -0.9752212 | 0.0000000 | 0.0000000 | 0.0000000 | -3.6715018 | 0.0000000 | 0.000000e+00 | 23 | 4 | 121.33748 | 71.3006848 |
| 9.5162588 | -9.0195973 | -8.1933034 | -2.4972345 | -0.0101215 | 0.0000000 | 0.0000000 | 0.0000000 | 0.0000000 | 0.0000000 | 0.0000000 | 0.0000000 | 0.0000000 | -8.0039374 | 0.0000000 | 0.0000000 | 0.0000000 | -7.3593546 | 0.0000000 | 0.0000000 | 0.0000000 | -7.1383967 | -2.0937351 | -4.6059607 | 0.000000e+00 | 23 | 5 | 70.48446 | 20.4771041 |
| 373.3243397 | -3121.8967873 | 0.0000000 | 0.0000000 | 0.0000000 | 0.0000000 | 0.0000000 | -8.1706635 | 0.0000000 | 0.0000000 | 0.0000000 | 0.0000000 | -112.1062496 | -258.0813823 | 0.0000000 | 0.0000000 | 0.0000000 | 0.0000000 | 0.0000000 | -0.2568294 | 0.0000000 | -0.0102317 | -243.5434585 | -66.9984868 | -6.928700e-03 | 23 | 6 | 50.00893 | 0.0089254 |
| 0.0000000 | 0.0000000 | -1.7451860 | -0.1389066 | -0.1507626 | 0.0000000 | 0.0000000 | -0.0680219 | 0.0000000 | 0.0000000 | -0.0770315 | 0.0000000 | 0.0000000 | -0.4415964 | -0.6181796 | 0.0000000 | 0.0000000 | -0.1475463 | -0.0889961 | 0.0000000 | 0.0000000 | 0.0000000 | 0.0000000 | -1.1845198 | 0.000000e+00 | 24 | 1 | 712.05751 | 660.5389024 |
| 0.2119399 | -0.2959301 | -2.4462289 | 0.0000000 | -0.1758293 | 0.0000000 | 0.0000000 | -0.1472364 | 0.0000000 | -0.0518197 | -0.0923218 | 0.0000000 | 0.0000000 | -1.5015971 | 0.0000000 | 0.0000000 | 0.0000000 | 0.0000000 | -0.5079754 | 0.0000000 | 0.0000000 | -0.1042853 | -0.7667295 | -0.4545793 | 0.000000e+00 | 24 | 2 | 451.82927 | 401.3752179 |
| 1.1764525 | -0.9461069 | -1.0822742 | -1.1772465 | 0.0000000 | 0.0000000 | 0.0000000 | 0.0000000 | 0.0000000 | 0.0000000 | -0.6532452 | 0.0000000 | -0.7668589 | -1.0768936 | -1.0705952 | 0.0000000 | 0.0000000 | -0.2355829 | 0.0000000 | 0.0000000 | 0.0000000 | 0.0000000 | -2.2808956 | -1.2695024 | 0.000000e+00 | 24 | 3 | 215.78030 | 165.6205625 |
| 2.8845339 | -0.8716734 | -7.7253022 | -0.0532880 | -1.7362437 | 0.0000000 | 0.0000000 | -1.2116162 | 0.0000000 | 0.0000000 | 0.0000000 | 0.0000000 | 0.0000000 | -1.9069330 | 0.0000000 | 0.0000000 | 0.0000000 | 0.0000000 | 0.0000000 | 0.0000000 | 0.0000000 | -2.1271058 | -0.3187558 | -2.8969260 | 0.000000e+00 | 24 | 4 | 89.45518 | 39.4036638 |
| 8.8381715 | -3.9647069 | 0.0000000 | -12.6677774 | -1.5012534 | 0.0000000 | 0.0000000 | 0.0000000 | 0.0000000 | 0.0000000 | -0.5830915 | 0.0000000 | 0.0000000 | 0.0000000 | 0.0000000 | 0.0000000 | -7.9164520 | 0.0000000 | 0.0000000 | 0.0000000 | 0.0000000 | 0.0000000 | -8.1415793 | 0.0000000 | -2.065254e+04 | 24 | 5 | 73.96683 | 23.9668342 |
| 0.0000000 | 0.0000000 | -1.7451860 | -0.1389066 | -0.1507626 | 0.0000000 | 0.0000000 | -0.0680219 | 0.0000000 | 0.0000000 | -0.0770315 | 0.0000000 | 0.0000000 | -0.4415964 | -0.6181796 | 0.0000000 | 0.0000000 | -0.1475463 | -0.0889961 | 0.0000000 | 0.0000000 | 0.0000000 | 0.0000000 | -1.1845198 | 0.000000e+00 | 25 | 1 | 712.05751 | 660.5389024 |
| 0.6810271 | -0.0012546 | -2.6336121 | -0.0015025 | -0.3230677 | -0.0001463 | 0.0000000 | -0.3192382 | -0.0005315 | -0.3259300 | -0.3318208 | -0.0002602 | -0.0000081 | -0.2250313 | -0.3952825 | -0.0003974 | -0.0002836 | -0.0034817 | -0.2939341 | -0.0002281 | -0.0010193 | -0.2217935 | -1.1401485 | -0.5998094 | 0.000000e+00 | 25 | 2 | 406.86547 | 356.4903962 |
| 2.1720191 | -0.9106758 | -2.7559125 | -1.0540514 | -1.0247175 | 0.0000000 | 0.0000000 | -0.7467568 | 0.0000000 | 0.0000000 | -0.4722549 | 0.0000000 | 0.0000000 | -2.3533227 | 0.0000000 | 0.0000000 | 0.0000000 | -0.1430896 | -0.9433686 | 0.0000000 | -1.2591829 | 0.0000000 | -0.6645154 | -1.8529113 | 0.000000e+00 | 25 | 3 | 218.02218 | 167.8667909 |
| 3.6800477 | 0.0000000 | -5.7068180 | -2.1429236 | 0.0000000 | 0.0000000 | 0.0000000 | 0.0000000 | 0.0000000 | 0.0000000 | 0.0000000 | 0.0000000 | 0.0000000 | -0.5964934 | -0.9448594 | 0.0000000 | -3.9018587 | -1.9513174 | 0.0000000 | 0.0000000 | 0.0000000 | -2.2280526 | -3.6447268 | 0.0000000 | -1.870898e+00 | 25 | 4 | 79.71835 | 29.7036296 |
| 9.4698127 | -19.3505472 | 0.0000000 | -2.4231570 | 0.0000000 | 0.0000000 | 0.0000000 | 0.0000000 | 0.0000000 | 0.0000000 | 0.0000000 | -1.0588568 | -0.5909736 | -25.4618873 | -1.8041081 | 0.0000000 | 0.0000000 | -2.3425711 | 0.0000000 | 0.0000000 | 0.0000000 | 0.0000000 | -8.8617290 | 0.0000000 | -5.026745e+00 | 25 | 5 | 64.17802 | 14.1706639 |
| 73.7355946 | -92.5353222 | -13.1243297 | -125.6106015 | 0.0000000 | 0.0000000 | -58.2570133 | -58.6114267 | 0.0000000 | -2.1622234 | -1.8650780 | 0.0000000 | 0.0000000 | 0.0000000 | 0.0000000 | -3.8216637 | 0.0000000 | 0.0000000 | -69.5745331 | 0.0000000 | 0.0000000 | 0.0000000 | 0.0000000 | 0.0000000 | -3.411029e+02 | 25 | 6 | 50.00736 | 0.0000003 |
| 0.0000000 | 0.0000000 | -1.7451860 | -0.1389066 | -0.1507626 | 0.0000000 | 0.0000000 | -0.0680219 | 0.0000000 | 0.0000000 | -0.0770315 | 0.0000000 | 0.0000000 | -0.4415964 | -0.6181796 | 0.0000000 | 0.0000000 | -0.1475463 | -0.0889961 | 0.0000000 | 0.0000000 | 0.0000000 | 0.0000000 | -1.1845198 | 0.000000e+00 | 26 | 1 | 712.05751 | 660.5389024 |
| 0.3439026 | -0.2822124 | -1.9733528 | -0.5123910 | -0.3174527 | 0.0000000 | 0.0000000 | 0.0000000 | 0.0000000 | 0.0000000 | -0.2921193 | 0.0000000 | 0.0000000 | -1.5914016 | 0.0000000 | 0.0000000 | 0.0000000 | 0.0000000 | 0.0000000 | 0.0000000 | 0.0000000 | 0.0000000 | -0.8543483 | -1.3326025 | 0.000000e+00 | 26 | 2 | 420.52887 | 370.0433242 |
| 0.0000000 | -1.6825306 | -0.9636819 | -1.0517636 | -0.5307774 | 0.0000000 | 0.0000000 | 0.0000000 | 0.0000000 | 0.0000000 | 0.0000000 | 0.0000000 | 0.0000000 | -4.4569619 | 0.0000000 | 0.0000000 | 0.0000000 | -0.2736250 | 0.0000000 | 0.0000000 | 0.0000000 | -1.3632826 | -0.9625061 | -1.4271787 | 0.000000e+00 | 26 | 3 | 203.00580 | 152.8834774 |
| 4.1376052 | 0.0000000 | -1.4575976 | -6.4913756 | -0.7091672 | 0.0000000 | 0.0000000 | 0.0000000 | 0.0000000 | 0.0000000 | 0.0000000 | 0.0000000 | -0.0008713 | 0.0000000 | 0.0000000 | 0.0000000 | -0.0002128 | 0.0000000 | 0.0000000 | 0.0000000 | 0.0000000 | -0.7916563 | -8.0925953 | 0.0000000 | -1.585391e+00 | 26 | 4 | 83.42315 | 33.3937102 |
| 5.7337476 | -30.4569426 | 0.0000000 | -6.8908715 | 0.0000000 | 0.0000000 | 0.0000000 | -0.1382029 | 0.0000000 | 0.0000000 | 0.0000000 | 0.0000000 | 0.0000000 | -1.2033847 | 0.0000000 | 0.0000000 | 0.0000000 | 0.0000000 | 0.0000000 | 0.0000000 | 0.0000000 | -19.0514686 | -6.6556164 | 0.0000000 | -1.825487e+02 | 26 | 5 | 71.31165 | 21.2969306 |
| 0.0000000 | 0.0000000 | -1.7451860 | -0.1389066 | -0.1507626 | 0.0000000 | 0.0000000 | -0.0680219 | 0.0000000 | 0.0000000 | -0.0770315 | 0.0000000 | 0.0000000 | -0.4415964 | -0.6181796 | 0.0000000 | 0.0000000 | -0.1475463 | -0.0889961 | 0.0000000 | 0.0000000 | 0.0000000 | 0.0000000 | -1.1845198 | 0.000000e+00 | 27 | 1 | 712.05751 | 660.5389024 |
| 0.4154389 | -0.4683916 | -2.4143467 | -0.0028041 | -0.5504298 | 0.0000000 | 0.0000000 | -0.3652103 | -0.0004123 | 0.0000000 | 0.0000000 | -0.0012515 | -0.0061992 | -0.6864690 | -0.7579823 | 0.0000000 | -0.0004372 | -0.0040781 | -0.0000721 | -0.3399238 | -0.0015563 | -0.0928873 | -0.1828229 | -1.1968566 | 0.000000e+00 | 27 | 2 | 391.15082 | 340.6350809 |
| 1.1499498 | -1.1491748 | -1.4623360 | 0.0000000 | -0.4756336 | 0.0000000 | 0.0000000 | -0.1940226 | 0.0000000 | 0.0000000 | 0.0000000 | 0.0000000 | 0.0000000 | -2.7301845 | 0.0000000 | 0.0000000 | 0.0000000 | -0.3900601 | -1.1622675 | 0.0000000 | 0.0000000 | 0.0000000 | -1.5310941 | -2.1489864 | 0.000000e+00 | 27 | 3 | 194.26514 | 144.1694631 |
| 3.1064830 | -21.4958368 | 0.0000000 | -8.9925803 | -0.1157705 | 0.0000000 | 0.0000000 | 0.0000000 | 0.0000000 | 0.0000000 | -4.5859514 | 0.0000000 | 0.0000000 | 0.0000000 | -3.6926660 | 0.0000000 | 0.0000000 | -2.3263147 | 0.0000000 | 0.0000000 | 0.0000000 | 0.0000000 | 0.0000000 | -0.1801972 | -3.736137e-01 | 27 | 4 | 93.85404 | 43.8098835 |
| 18.1617734 | 0.0000000 | -13.6850046 | -0.6933946 | 0.0000000 | 0.0000000 | 0.0000000 | -2.9556774 | 0.0000000 | 0.0000000 | -16.0221308 | 0.0000000 | -8.1506500 | -2.3346052 | 0.0000000 | 0.0000000 | -1.8973304 | -3.8746371 | 0.0000000 | 0.0000000 | -7.9224564 | -2.5780171 | 0.0000000 | -7.6616645 | -1.054190e+05 | 27 | 5 | 57.16378 | 7.1564181 |
| 0.0000000 | 0.0000000 | -1.7451860 | -0.1389066 | -0.1507626 | 0.0000000 | 0.0000000 | -0.0680219 | 0.0000000 | 0.0000000 | -0.0770315 | 0.0000000 | 0.0000000 | -0.4415964 | -0.6181796 | 0.0000000 | 0.0000000 | -0.1475463 | -0.0889961 | 0.0000000 | 0.0000000 | 0.0000000 | 0.0000000 | -1.1845198 | 0.000000e+00 | 28 | 1 | 712.05751 | 660.5389024 |
| 0.8031210 | -0.9260443 | -1.3807148 | 0.0000000 | -0.2895679 | 0.0000000 | 0.0000000 | -0.3524261 | 0.0000000 | 0.0000000 | -0.9913418 | 0.0000000 | 0.0000000 | -0.7672883 | -0.9333235 | 0.0000000 | 0.0000000 | 0.0000000 | 0.0000000 | 0.0000000 | 0.0000000 | 0.0000000 | -1.8330822 | 0.0000000 | 0.000000e+00 | 28 | 2 | 331.28997 | 280.7995818 |
| 0.8984972 | -0.0417491 | -3.3684306 | -0.9091170 | -0.4960432 | 0.0000000 | 0.0000000 | 0.0000000 | 0.0000000 | 0.0000000 | 0.0000000 | 0.0000000 | 0.0000000 | -1.0173859 | 0.0000000 | 0.0000000 | 0.0000000 | 0.0000000 | -0.3132694 | 0.0000000 | 0.0000000 | 0.0000000 | -1.6126950 | -1.5673199 | 0.000000e+00 | 28 | 3 | 220.48929 | 170.3516274 |
| 4.3922903 | -1.9377713 | -1.6905122 | -1.9473252 | -0.2889461 | -0.5190754 | -0.6502506 | 0.0000000 | 0.0000000 | 0.0000000 | 0.0000000 | 0.0000000 | 0.0000000 | 0.0000000 | -2.1529923 | 0.0000000 | -0.0000001 | 0.0000000 | -0.6464474 | 0.0000000 | 0.0000000 | 0.0000000 | -6.1377535 | -0.4554200 | 0.000000e+00 | 28 | 4 | 106.61082 | 56.6034621 |
| 9.9196156 | 0.0000000 | 0.0000000 | 0.0000000 | 0.0000000 | 0.0000000 | 0.0000000 | -2.2125512 | 0.0000000 | -3.9546096 | -14.5604423 | 0.0000000 | -3.6312226 | -10.3199961 | 0.0000000 | 0.0000000 | 0.0000000 | -5.9943197 | 0.0000000 | 0.0000000 | -3.3878927 | 0.0000000 | 0.0000000 | -7.8652185 | -8.163616e+04 | 28 | 5 | 59.14187 | 9.1271470 |
| 0.0000000 | 0.0000000 | -1.7451860 | -0.1389066 | -0.1507626 | 0.0000000 | 0.0000000 | -0.0680219 | 0.0000000 | 0.0000000 | -0.0770315 | 0.0000000 | 0.0000000 | -0.4415964 | -0.6181796 | 0.0000000 | 0.0000000 | -0.1475463 | -0.0889961 | 0.0000000 | 0.0000000 | 0.0000000 | 0.0000000 | -1.1845198 | 0.000000e+00 | 29 | 1 | 712.05751 | 660.5389024 |
| 0.3549277 | -0.3840845 | -1.5801949 | 0.0000000 | -0.3892546 | 0.0000000 | 0.0000000 | -0.2082063 | 0.0000000 | 0.0000000 | 0.0000000 | 0.0000000 | 0.0000000 | -1.2816113 | -0.6057180 | 0.0000000 | 0.0000000 | -0.2679209 | 0.0000000 | 0.0000000 | 0.0000000 | 0.0000000 | -0.5032217 | -1.5075099 | 0.000000e+00 | 29 | 2 | 407.90646 | 357.4784597 |
| 1.3512290 | -1.3901264 | -1.5099000 | -0.0612813 | -0.8880479 | 0.0000000 | 0.0000000 | -0.1367838 | 0.0000000 | 0.0000000 | 0.0000000 | 0.0000000 | 0.0000000 | -3.8071311 | 0.0000000 | 0.0000000 | 0.0000000 | -1.5952902 | 0.0000000 | 0.0000000 | 0.0000000 | -3.2420624 | -0.3267850 | -2.0085422 | 0.000000e+00 | 29 | 3 | 237.61064 | 187.4557725 |
| 1.7615267 | -13.0026262 | 0.0000000 | -0.7145458 | 0.0000000 | 0.0000000 | 0.0000000 | 0.0000000 | 0.0000000 | 0.0000000 | -0.5274698 | 0.0000000 | 0.0000000 | -8.5471796 | 0.0000000 | 0.0000000 | 0.0000000 | -2.5196234 | 0.0000000 | 0.0000000 | 0.0000000 | -12.2010382 | 0.0000000 | -2.0528831 | -1.841552e+00 | 29 | 4 | 109.68825 | 59.6588095 |
| 0.6248863 | -12.6983733 | 0.0000000 | -4.8515708 | -0.0739382 | 0.0000000 | 0.0000000 | -1.5926470 | 0.0000000 | 0.0000000 | -1.0044699 | 0.0000000 | 0.0000000 | -6.1688717 | 0.0000000 | 0.0000000 | 0.0000000 | -0.3947208 | 0.0000000 | 0.0000000 | 0.0000000 | 0.0000000 | -1.3051843 | 0.0000000 | 0.000000e+00 | 29 | 5 | 71.60684 | 21.5847575 |
| 2.4120460 | -7.7794518 | 0.0000000 | 0.0000000 | 0.0000000 | 0.0000000 | 0.0000000 | 0.0000000 | 0.0000000 | -20.2905268 | -3.2871345 | 0.0000000 | -1.3562747 | -5.0842703 | 0.0000000 | 0.0000000 | 0.0000000 | 0.0000000 | 0.0000000 | 0.0000000 | -5.3734715 | -1.3579370 | 0.0000000 | 0.0000000 | -3.861586e+04 | 29 | 6 | 57.94353 | 7.9435252 |
| 0.0000000 | 0.0000000 | -1.7451860 | -0.1389066 | -0.1507626 | 0.0000000 | 0.0000000 | -0.0680219 | 0.0000000 | 0.0000000 | -0.0770315 | 0.0000000 | 0.0000000 | -0.4415964 | -0.6181796 | 0.0000000 | 0.0000000 | -0.1475463 | -0.0889961 | 0.0000000 | 0.0000000 | 0.0000000 | 0.0000000 | -1.1845198 | 0.000000e+00 | 30 | 1 | 712.05751 | 660.5389024 |
| 0.0000000 | 0.0000000 | -1.4578946 | 0.0000000 | -0.3516191 | 0.0000000 | 0.0000000 | -0.3153832 | 0.0000000 | 0.0000000 | -0.0844285 | 0.0000000 | 0.0000000 | -2.3375412 | 0.0000000 | 0.0000000 | 0.0000000 | 0.0000000 | -0.2641707 | 0.0000000 | 0.0000000 | 0.0000000 | -0.0230645 | -2.0771087 | 0.000000e+00 | 30 | 2 | 415.50592 | 365.0202556 |
| 0.3292790 | -0.9146301 | -2.2328550 | -1.1175790 | -0.6162524 | 0.0000000 | 0.0000000 | -0.0096470 | 0.0000000 | 0.0000000 | -0.0451937 | 0.0000000 | 0.0000000 | -1.7997097 | 0.0000000 | 0.0000000 | 0.0000000 | -0.6320942 | -0.3465697 | 0.0000000 | 0.0000000 | 0.0000000 | -0.4813319 | -2.0369594 | 0.000000e+00 | 30 | 3 | 196.57963 | 146.4545121 |
| 5.5586621 | -2.5003612 | 0.0000000 | -3.9750801 | -0.8530255 | 0.0000000 | 0.0000000 | -0.3915787 | 0.0000000 | 0.0000000 | 0.0000000 | -0.3744474 | -0.8764354 | -1.1291187 | 0.0000000 | 0.0000000 | 0.0000000 | -1.1709404 | 0.0000000 | 0.0000000 | -1.7494298 | -2.6811158 | -5.6083293 | 0.0000000 | -1.664670e+00 | 30 | 4 | 149.47673 | 99.4325673 |
| 5.2384291 | -6.7385173 | 0.0000000 | -2.4464507 | 0.0000000 | 0.0000000 | 0.0000000 | 0.0000000 | 0.0000000 | 0.0000000 | -0.1954535 | 0.0000000 | 0.0000000 | -5.8797110 | 0.0000000 | 0.0000000 | 0.0000000 | 0.0000000 | 0.0000000 | 0.0000000 | 0.0000000 | -1.5343105 | -9.5738532 | 0.0000000 | 0.000000e+00 | 30 | 5 | 62.40037 | 12.3856551 |
| 0.0000000 | -8.0742804 | 0.0000000 | -2.4464938 | 0.0000000 | 0.0000000 | 0.0000000 | 0.0000000 | 0.0000000 | 0.0000000 | -4.8693634 | 0.0000000 | 0.0000000 | -9.5078877 | 0.0000000 | 0.0000000 | 0.0000000 | 0.0000000 | 0.0000000 | 0.0000000 | -0.8293041 | 0.0000000 | 0.0000000 | 0.0000000 | -1.260965e+01 | 30 | 6 | 64.09190 | 14.0845426 |
| 0.0000000 | 0.0000000 | -1.7451860 | -0.1389066 | -0.1507626 | 0.0000000 | 0.0000000 | -0.0680219 | 0.0000000 | 0.0000000 | -0.0770315 | 0.0000000 | 0.0000000 | -0.4415964 | -0.6181796 | 0.0000000 | 0.0000000 | -0.1475463 | -0.0889961 | 0.0000000 | 0.0000000 | 0.0000000 | 0.0000000 | -1.1845198 | 0.000000e+00 | 31 | 1 | 712.05751 | 660.5389024 |
| 0.2983504 | -0.1176273 | -2.7651635 | 0.0000000 | 0.0000000 | 0.0000000 | 0.0000000 | 0.0000000 | 0.0000000 | 0.0000000 | -0.4394615 | 0.0000000 | 0.0000000 | -0.2683031 | -0.9697893 | 0.0000000 | 0.0000000 | -1.2664966 | 0.0000000 | 0.0000000 | 0.0000000 | 0.0000000 | -0.0025313 | -0.4811433 | 0.000000e+00 | 31 | 2 | 471.03912 | 420.5719102 |
| 0.9360332 | -1.1622258 | 0.0000000 | -5.4157451 | 0.0000000 | 0.0000000 | 0.0000000 | 0.0000000 | 0.0000000 | 0.0000000 | -0.7218940 | -0.8053159 | 0.0000000 | 0.0000000 | 0.0000000 | 0.0000000 | 0.0000000 | -1.8953194 | 0.0000000 | 0.0000000 | 0.0000000 | -3.2028657 | -0.7032580 | -2.1603235 | 0.000000e+00 | 31 | 3 | 215.75021 | 165.6262011 |
| 0.3892727 | -0.9220960 | 0.0000000 | -6.3430866 | -0.0946070 | 0.0000000 | 0.0000000 | 0.0000000 | 0.0000000 | 0.0000000 | 0.0000000 | 0.0000000 | -0.5419788 | -1.2799902 | 0.0000000 | 0.0000000 | 0.0000000 | -3.8506545 | 0.0000000 | 0.0000000 | 0.0000000 | -0.7151278 | -1.4710311 | 0.0000000 | 0.000000e+00 | 31 | 4 | 116.11048 | 66.0752892 |
| 0.1444292 | -7.8466050 | 0.0000000 | -4.7366012 | 0.0000000 | 0.0000000 | 0.0000000 | 0.0000000 | 0.0000000 | 0.0000000 | 0.0000000 | 0.0000000 | 0.0000000 | -5.4244708 | 0.0000000 | 0.0000000 | 0.0000000 | -1.9291010 | -3.5537140 | 0.0000000 | 0.0000000 | -0.9434871 | 0.0000000 | 0.0000000 | -3.816924e+00 | 31 | 5 | 71.62391 | 21.6091888 |
| 0.0000000 | 0.0000000 | -1.7451860 | -0.1389066 | -0.1507626 | 0.0000000 | 0.0000000 | -0.0680219 | 0.0000000 | 0.0000000 | -0.0770315 | 0.0000000 | 0.0000000 | -0.4415964 | -0.6181796 | 0.0000000 | 0.0000000 | -0.1475463 | -0.0889961 | 0.0000000 | 0.0000000 | 0.0000000 | 0.0000000 | -1.1845198 | 0.000000e+00 | 32 | 1 | 712.05751 | 660.5389024 |
| 0.4416588 | -0.5003346 | -1.3816070 | -0.7530086 | -0.1707876 | 0.0000000 | 0.0000000 | -0.0404171 | 0.0000000 | 0.0000000 | 0.0000000 | 0.0000000 | 0.0000000 | -0.5094404 | -0.9473170 | 0.0000000 | 0.0000000 | -0.6195231 | -0.4503543 | 0.0000000 | 0.0000000 | -0.2878723 | -0.5172134 | -1.0252709 | 0.000000e+00 | 32 | 2 | 424.76701 | 374.2790755 |
| 0.6865318 | -0.9453209 | -1.0969780 | -2.1768325 | -0.4516990 | 0.0000000 | 0.0000000 | 0.0000000 | 0.0000000 | 0.0000000 | 0.0000000 | 0.0000000 | 0.0000000 | -3.6571215 | 0.0000000 | 0.0000000 | 0.0000000 | -2.0887408 | 0.0000000 | 0.0000000 | 0.0000000 | -1.8965164 | -0.5678614 | -1.5733024 | 0.000000e+00 | 32 | 3 | 207.30078 | 157.2051044 |
| 1.6328460 | -9.4222347 | 0.0000000 | -1.0117266 | 0.0000000 | 0.0000000 | 0.0000000 | 0.0000000 | 0.0000000 | 0.0000000 | -1.4035420 | 0.0000000 | 0.0000000 | -5.0063372 | -3.8364003 | 0.0000000 | 0.0000000 | 0.0000000 | -0.3323139 | 0.0000000 | 0.0000000 | -0.9976572 | 0.0000000 | -3.0627060 | 0.000000e+00 | 32 | 4 | 90.45559 | 40.4187940 |
| 2.3330589 | -34.5054188 | 0.0000000 | -2.1097908 | -0.8979044 | 0.0000000 | 0.0000000 | 0.0000000 | 0.0000000 | 0.0000000 | 0.0000000 | 0.0000000 | 0.0000000 | 0.0000000 | 0.0000000 | 0.0000000 | 0.0000000 | 0.0000000 | 0.0000000 | 0.0000000 | 0.0000000 | -7.0690077 | -2.0894238 | -2.4806476 | 0.000000e+00 | 32 | 5 | 81.96366 | 31.9636578 |
| 0.0000000 | 0.0000000 | -1.7451860 | -0.1389066 | -0.1507626 | 0.0000000 | 0.0000000 | -0.0680219 | 0.0000000 | 0.0000000 | -0.0770315 | 0.0000000 | 0.0000000 | -0.4415964 | -0.6181796 | 0.0000000 | 0.0000000 | -0.1475463 | -0.0889961 | 0.0000000 | 0.0000000 | 0.0000000 | 0.0000000 | -1.1845198 | 0.000000e+00 | 33 | 1 | 712.05751 | 660.5389024 |
| 0.0000000 | -0.1151583 | -1.5940658 | -1.2095900 | -0.0578760 | 0.0000000 | 0.0000000 | 0.0000000 | 0.0000000 | 0.0000000 | 0.0000000 | 0.0000000 | -0.4272855 | -1.8198713 | 0.0000000 | 0.0000000 | 0.0000000 | -0.7408650 | -0.0141887 | 0.0000000 | 0.0000000 | 0.0000000 | -0.4852689 | -0.9718102 | 0.000000e+00 | 33 | 2 | 401.72212 | 351.2499979 |
| 1.0606573 | -2.0341859 | -0.2756240 | -1.9900592 | -0.3986029 | 0.0000000 | 0.0000000 | -0.1887059 | 0.0000000 | 0.0000000 | 0.0000000 | 0.0000000 | 0.0000000 | -2.3374605 | -0.1135099 | 0.0000000 | 0.0000000 | 0.0000000 | 0.0000000 | 0.0000000 | 0.0000000 | 0.0000000 | -3.4369226 | 0.0000000 | 0.000000e+00 | 33 | 3 | 197.51035 | 147.3868362 |
| 1.8763191 | -3.1716365 | 0.0000000 | -1.2318393 | -0.0750449 | 0.0000000 | 0.0000000 | -0.4267801 | 0.0000000 | 0.0000000 | 0.0000000 | 0.0000000 | 0.0000000 | -6.1661084 | 0.0000000 | 0.0000000 | 0.0000000 | -4.3522169 | -0.3235424 | 0.0000000 | 0.0000000 | -1.4221890 | -1.5522072 | -0.1697284 | -8.428768e-01 | 33 | 4 | 100.26494 | 50.2354977 |
| 2.3372539 | -9.7332380 | 0.0000000 | -47.0694089 | 0.0000000 | 0.0000000 | 0.0000000 | 0.0000000 | 0.0000000 | 0.0000000 | 0.0000000 | 0.0000000 | 0.0000000 | 0.0000000 | -2.3841766 | 0.0000000 | -1.7273174 | -3.3614287 | 0.0000000 | 0.0000000 | 0.0000000 | 0.0000000 | 0.0000000 | 0.0000000 | -5.245637e+04 | 33 | 5 | 63.67767 | 13.6776742 |
| 20.9882411 | 0.0000000 | -12.3821669 | -3.8482155 | 0.0000000 | 0.0000000 | 0.0000000 | -0.2003919 | -3.7258360 | 0.0000000 | 0.0000000 | 0.0000000 | 0.0000000 | -1.4341235 | 0.0000000 | 0.0000000 | -0.2379747 | -18.9607715 | 0.0000000 | -6.4635622 | 0.0000000 | 0.0000000 | -26.9485282 | 0.0000000 | 0.000000e+00 | 33 | 6 | 50.00736 | 0.0000000 |
| 0.0000000 | 0.0000000 | -1.7451860 | -0.1389066 | -0.1507626 | 0.0000000 | 0.0000000 | -0.0680219 | 0.0000000 | 0.0000000 | -0.0770315 | 0.0000000 | 0.0000000 | -0.4415964 | -0.6181796 | 0.0000000 | 0.0000000 | -0.1475463 | -0.0889961 | 0.0000000 | 0.0000000 | 0.0000000 | 0.0000000 | -1.1845198 | 0.000000e+00 | 34 | 1 | 712.05751 | 660.5389024 |
| 0.1475528 | -0.8532893 | -1.2066130 | -0.6445852 | -0.1669990 | 0.0000000 | 0.0000000 | 0.0000000 | 0.0000000 | 0.0000000 | -0.1753020 | 0.0000000 | 0.0000000 | -1.7226068 | 0.0000000 | 0.0000000 | 0.0000000 | -0.6190566 | 0.0000000 | 0.0000000 | 0.0000000 | -0.6660611 | -0.3176484 | -1.3978477 | 0.000000e+00 | 34 | 2 | 420.66752 | 370.1325555 |
| 0.7197946 | 0.0000000 | -3.5045691 | -3.4377122 | -0.4713606 | -0.1360826 | 0.0000000 | 0.0000000 | 0.0000000 | 0.0000000 | 0.0000000 | 0.0000000 | 0.0000000 | 0.0000000 | 0.0000000 | 0.0000000 | 0.0000000 | -0.7496055 | 0.0000000 | 0.0000000 | 0.0000000 | -2.3028411 | -1.1892916 | -1.0667169 | 0.000000e+00 | 34 | 3 | 208.45371 | 158.3042598 |
| 6.5881430 | -5.8412466 | -3.0413052 | -1.8717030 | 0.0000000 | 0.0000000 | 0.0000000 | -0.5595387 | 0.0000000 | 0.0000000 | -1.9224621 | 0.0000000 | 0.0000000 | 0.0000000 | -2.4679460 | -0.4955262 | 0.0000000 | 0.0000000 | 0.0000000 | 0.0000000 | 0.0000000 | -1.0788919 | -6.3915719 | 0.0000000 | -1.213792e+00 | 34 | 4 | 108.85830 | 58.8214964 |
| 3.2959364 | -24.9207591 | 0.0000000 | 0.0000000 | -1.0532529 | 0.0000000 | 0.0000000 | 0.0000000 | 0.0000000 | 0.0000000 | 0.0000000 | 0.0000000 | 0.0000000 | -16.3306998 | 0.0000000 | 0.0000000 | 0.0000000 | 0.0000000 | 0.0000000 | 0.0000000 | 0.0000000 | 0.0000000 | -3.1472121 | -2.3961076 | 0.000000e+00 | 34 | 5 | 68.63106 | 18.6310584 |
| 0.0000000 | 0.0000000 | -1.7451860 | -0.1389066 | -0.1507626 | 0.0000000 | 0.0000000 | -0.0680219 | 0.0000000 | 0.0000000 | -0.0770315 | 0.0000000 | 0.0000000 | -0.4415964 | -0.6181796 | 0.0000000 | 0.0000000 | -0.1475463 | -0.0889961 | 0.0000000 | 0.0000000 | 0.0000000 | 0.0000000 | -1.1845198 | 0.000000e+00 | 35 | 1 | 712.05751 | 660.5389024 |
| 0.6876048 | 0.0000000 | -1.7909903 | -0.7288828 | -0.0297971 | 0.0000000 | 0.0000000 | -0.0900210 | 0.0000000 | 0.0000000 | 0.0000000 | 0.0000000 | 0.0000000 | 0.0000000 | -1.2126938 | 0.0000000 | 0.0000000 | -0.2612301 | -0.0950726 | 0.0000000 | -0.0732056 | -0.4477392 | -1.5601833 | -0.6449377 | 0.000000e+00 | 35 | 2 | 395.74413 | 345.2823484 |
| 0.1319366 | -2.6274957 | 0.0000000 | -3.2595375 | -0.6420178 | 0.0000000 | 0.0000000 | 0.0000000 | 0.0000000 | 0.0000000 | 0.0000000 | 0.0000000 | 0.0000000 | -3.8255745 | 0.0000000 | 0.0000000 | 0.0000000 | -1.4549502 | 0.0000000 | 0.0000000 | 0.0000000 | -0.3131611 | 0.0000000 | -1.9816533 | 0.000000e+00 | 35 | 3 | 188.96310 | 138.8232633 |
| 2.2114319 | -2.7943996 | 0.0000000 | 0.0000000 | -0.6656094 | 0.0000000 | 0.0000000 | -1.3323897 | -0.9924119 | 0.0000000 | 0.0000000 | 0.0000000 | -0.9943389 | -24.7629465 | 0.0000000 | 0.0000000 | 0.0000000 | -1.8767239 | 0.0000000 | 0.0000000 | 0.0000000 | 0.0000000 | -1.6268121 | 0.0000000 | 0.000000e+00 | 35 | 4 | 98.25235 | 48.2155556 |
| 9.8943282 | -9.3704332 | 0.0000000 | -4.1530451 | -2.8939063 | 0.0000000 | 0.0000000 | 0.0000000 | 0.0000000 | 0.0000000 | 0.0000000 | 0.0000000 | -5.3618253 | -2.1109523 | 0.0000000 | 0.0000000 | 0.0000000 | 0.0000000 | 0.0000000 | 0.0000000 | 0.0000000 | -16.6494069 | -9.2773885 | -0.4183001 | -1.725924e+01 | 35 | 5 | 68.20231 | 18.2023086 |
| 0.0000000 | 0.0000000 | -1.7451860 | -0.1389066 | -0.1507626 | 0.0000000 | 0.0000000 | -0.0680219 | 0.0000000 | 0.0000000 | -0.0770315 | 0.0000000 | 0.0000000 | -0.4415964 | -0.6181796 | 0.0000000 | 0.0000000 | -0.1475463 | -0.0889961 | 0.0000000 | 0.0000000 | 0.0000000 | 0.0000000 | -1.1845198 | 0.000000e+00 | 36 | 1 | 712.05751 | 660.5389024 |
| 0.3890157 | -1.5163674 | -1.5916802 | -0.0499398 | -0.4559308 | 0.0000000 | -0.0219595 | 0.0000000 | 0.0000000 | 0.0000000 | 0.0000000 | 0.0000000 | 0.0000000 | -2.4350757 | 0.0000000 | 0.0000000 | 0.0000000 | 0.0000000 | -0.5439460 | 0.0000000 | 0.0000000 | -0.0975599 | -0.1602433 | -1.9264011 | 0.000000e+00 | 36 | 2 | 404.47535 | 354.0703024 |
| 1.1273869 | -1.7480026 | 0.0000000 | -0.5615264 | -0.6667259 | 0.0000000 | 0.0000000 | -0.8786937 | 0.0000000 | 0.0000000 | -0.1287025 | 0.0000000 | 0.0000000 | -1.3834280 | 0.0000000 | 0.0000000 | 0.0000000 | 0.0000000 | 0.0000000 | 0.0000000 | 0.0000000 | -3.7447925 | -2.8803425 | 0.0000000 | 0.000000e+00 | 36 | 3 | 232.41082 | 182.2783472 |
| 5.2461765 | -1.2974464 | -3.7187984 | -1.0254862 | -0.0426678 | 0.0000000 | 0.0000000 | -0.0917342 | 0.0000000 | 0.0000000 | -0.5626120 | 0.0000000 | 0.0000000 | -0.7435438 | -1.5900319 | 0.0000000 | 0.0000000 | -1.8798043 | 0.0000000 | 0.0000000 | 0.0000000 | 0.0000000 | -5.5458796 | 0.0000000 | -1.406950e+01 | 36 | 4 | 107.05098 | 57.0068211 |
| 1.1571582 | -21.3303829 | 0.0000000 | 0.0000000 | 0.0000000 | 0.0000000 | 0.0000000 | 0.0000000 | 0.0000000 | 0.0000000 | -6.2004422 | 0.0000000 | -0.3432013 | -10.3003243 | 0.0000000 | 0.0000000 | 0.0000000 | 0.0000000 | 0.0000000 | 0.0000000 | 0.0000000 | -2.0202303 | 0.0000000 | -1.9888554 | -3.252498e+04 | 36 | 6 | 53.43881 | 3.4314493 |
| 0.0000000 | 0.0000000 | -1.7451860 | -0.1389066 | -0.1507626 | 0.0000000 | 0.0000000 | -0.0680219 | 0.0000000 | 0.0000000 | -0.0770315 | 0.0000000 | 0.0000000 | -0.4415964 | -0.6181796 | 0.0000000 | 0.0000000 | -0.1475463 | -0.0889961 | 0.0000000 | 0.0000000 | 0.0000000 | 0.0000000 | -1.1845198 | 0.000000e+00 | 37 | 1 | 712.05751 | 660.5389024 |
| 0.3139316 | -0.6043187 | -1.6696591 | -0.1116113 | -0.2502385 | 0.0000000 | 0.0000000 | 0.0000000 | 0.0000000 | 0.0000000 | 0.0000000 | -0.0893824 | -0.0711191 | -2.1423624 | 0.0000000 | 0.0000000 | 0.0000000 | -0.3925010 | -0.2125654 | 0.0000000 | 0.0000000 | 0.0000000 | -0.6934734 | -1.0741818 | 0.000000e+00 | 37 | 2 | 400.71445 | 350.1570459 |
| 1.0345747 | -1.2824591 | -0.7876052 | -5.8381954 | 0.0000000 | 0.0000000 | 0.0000000 | -0.1908099 | 0.0000000 | 0.0000000 | -0.3046691 | 0.0000000 | 0.0000000 | -0.7544327 | 0.0000000 | 0.0000000 | 0.0000000 | -2.4366600 | -0.8557311 | 0.0000000 | 0.0000000 | -1.6517431 | -1.4233103 | 0.0000000 | 0.000000e+00 | 37 | 3 | 206.64000 | 156.5453136 |
| 3.8056462 | -0.3339987 | -2.2900142 | -1.2481833 | 0.0000000 | -1.0602941 | -0.4869365 | -0.4208205 | 0.0000000 | 0.0000000 | 0.0000000 | 0.0000000 | 0.0000000 | -2.7286118 | -0.0142866 | 0.0000000 | 0.0000000 | -1.8591588 | -0.3241423 | 0.0000000 | 0.0000000 | -0.0000026 | -4.2859803 | -0.0786929 | -2.056791e+00 | 37 | 4 | 116.83049 | 66.7716100 |
| 0.0000000 | -35.3367555 | 0.0000000 | -5.3794564 | 0.0000000 | 0.0000000 | 0.0000000 | -0.1575694 | 0.0000000 | 0.0000000 | -0.7571879 | 0.0000000 | 0.0000000 | -1.1387035 | 0.0000000 | 0.0000000 | 0.0000000 | 0.0000000 | 0.0000000 | 0.0000000 | 0.0000000 | -3.3421896 | 0.0000000 | -2.0988956 | -6.664648e-01 | 37 | 5 | 74.62936 | 24.6146392 |
| 219.7171763 | -938.6711970 | -9.0843660 | -245.8827005 | -115.0641204 | 0.0000000 | 0.0000000 | -11.2341258 | 0.0000000 | 0.0000000 | 0.0000000 | 0.0000000 | 0.0000000 | 0.0000000 | 0.0000000 | -0.0928566 | -3.6808206 | -67.4100654 | -66.5586915 | 0.0000000 | 0.0000000 | -0.0003151 | 0.0000000 | 0.0000000 | -1.367449e+03 | 37 | 6 | 50.00389 | 0.0038935 |
| 0.0000000 | 0.0000000 | -1.7451860 | -0.1389066 | -0.1507626 | 0.0000000 | 0.0000000 | -0.0680219 | 0.0000000 | 0.0000000 | -0.0770315 | 0.0000000 | 0.0000000 | -0.4415964 | -0.6181796 | 0.0000000 | 0.0000000 | -0.1475463 | -0.0889961 | 0.0000000 | 0.0000000 | 0.0000000 | 0.0000000 | -1.1845198 | 0.000000e+00 | 38 | 1 | 712.05751 | 660.5389024 |
| 0.1602266 | -1.0648554 | -0.4974579 | -1.1457967 | -0.2773295 | 0.0000000 | 0.0000000 | 0.0000000 | 0.0000000 | 0.0000000 | 0.0000000 | 0.0000000 | 0.0000000 | -1.4291186 | 0.0000000 | 0.0000000 | 0.0000000 | 0.0000000 | -0.3889070 | 0.0000000 | 0.0000000 | -0.4401888 | -1.5682519 | -0.3889635 | 0.000000e+00 | 38 | 2 | 419.36154 | 368.9299186 |
| 1.5618067 | -3.0785198 | -4.4392675 | -0.5673804 | 0.0000000 | 0.0000000 | 0.0000000 | 0.0000000 | 0.0000000 | 0.0000000 | 0.0000000 | 0.0000000 | 0.0000000 | 0.0000000 | -1.7373973 | 0.0000000 | 0.0000000 | 0.0000000 | -0.7550704 | 0.0000000 | 0.0000000 | 0.0000000 | -1.1446854 | -1.7028967 | 0.000000e+00 | 38 | 3 | 206.00527 | 155.9128675 |
| 0.6106507 | -5.7574577 | 0.0000000 | -2.8889553 | 0.0000000 | 0.0000000 | 0.0000000 | 0.0000000 | 0.0000000 | 0.0000000 | -2.3749664 | -0.0687791 | 0.0000000 | 0.0000000 | -3.5481345 | 0.0000000 | 0.0000000 | -0.3037394 | 0.0000000 | 0.0000000 | -0.2399015 | -3.2528747 | 0.0000000 | -1.6429459 | 0.000000e+00 | 38 | 4 | 108.74601 | 58.7165698 |
| 0.1100841 | -17.9577322 | 0.0000000 | 0.0000000 | 0.0000000 | 0.0000000 | 0.0000000 | 0.0000000 | 0.0000000 | 0.0000000 | -2.7199459 | 0.0000000 | 0.0000000 | -19.4226427 | 0.0000000 | 0.0000000 | 0.0000000 | 0.0000000 | 0.0000000 | 0.0000000 | 0.0000000 | 0.0000000 | -0.9677639 | 0.0000000 | -1.187140e-02 | 38 | 5 | 78.36202 | 28.3546645 |
| 0.0000000 | 0.0000000 | -1.7451860 | -0.1389066 | -0.1507626 | 0.0000000 | 0.0000000 | -0.0680219 | 0.0000000 | 0.0000000 | -0.0770315 | 0.0000000 | 0.0000000 | -0.4415964 | -0.6181796 | 0.0000000 | 0.0000000 | -0.1475463 | -0.0889961 | 0.0000000 | 0.0000000 | 0.0000000 | 0.0000000 | -1.1845198 | 0.000000e+00 | 39 | 1 | 712.05751 | 660.5389024 |
| 0.1990671 | -0.2912834 | -2.3748585 | -0.2304809 | -0.3288228 | 0.0000000 | 0.0000000 | -0.2767825 | 0.0000000 | 0.0000000 | -0.0457949 | 0.0000000 | 0.0000000 | -1.0269936 | 0.0000000 | 0.0000000 | -0.0098681 | 0.0000000 | -0.2642810 | 0.0000000 | 0.0000000 | 0.0000000 | -0.3608858 | -1.3630245 | 0.000000e+00 | 39 | 2 | 422.43753 | 371.8869101 |
| 2.2654511 | 0.0000000 | -3.8715188 | 0.0000000 | 0.0000000 | 0.0000000 | 0.0000000 | -0.0812114 | 0.0000000 | 0.0000000 | -1.1136903 | 0.0000000 | 0.0000000 | -2.1180414 | -0.2663376 | 0.0000000 | 0.0000000 | 0.0000000 | -0.5825881 | 0.0000000 | 0.0000000 | 0.0000000 | -3.5742260 | 0.0000000 | 0.000000e+00 | 39 | 3 | 209.59367 | 159.4487938 |
| 4.4002220 | -4.4719068 | 0.0000000 | 0.0000000 | -0.0629703 | 0.0000000 | 0.0000000 | -2.0660815 | 0.0000000 | 0.0000000 | -6.2379814 | 0.0000000 | 0.0000000 | -9.3143334 | 0.0000000 | 0.0000000 | 0.0000000 | -2.3397903 | 0.0000000 | 0.0000000 | -3.0978911 | 0.0000000 | 0.0000000 | -1.2346367 | -6.452347e+00 | 39 | 4 | 84.50789 | 34.4784527 |
| 3.2507953 | -3.9537983 | 0.0000000 | -5.4648475 | -0.1567782 | 0.0000000 | 0.0000000 | -0.0765020 | 0.0000000 | 0.0000000 | 0.0000000 | 0.0000000 | 0.0000000 | 0.0000000 | 0.0000000 | 0.0000000 | 0.0000000 | -4.4236293 | -0.4951776 | 0.0000000 | 0.0000000 | -8.2693389 | 0.0000000 | -12.4533155 | -3.483722e+04 | 39 | 5 | 57.50902 | 7.5016605 |
| 0.0000000 | 0.0000000 | -1.7451860 | -0.1389066 | -0.1507626 | 0.0000000 | 0.0000000 | -0.0680219 | 0.0000000 | 0.0000000 | -0.0770315 | 0.0000000 | 0.0000000 | -0.4415964 | -0.6181796 | 0.0000000 | 0.0000000 | -0.1475463 | -0.0889961 | 0.0000000 | 0.0000000 | 0.0000000 | 0.0000000 | -1.1845198 | 0.000000e+00 | 40 | 1 | 712.05751 | 660.5389024 |
| 0.0991577 | -0.0106002 | -2.3875283 | 0.0000000 | -0.2673219 | 0.0000000 | 0.0000000 | -0.2409184 | 0.0000000 | 0.0000000 | 0.0000000 | 0.0000000 | 0.0000000 | -1.2036707 | -0.4794588 | 0.0000000 | 0.0000000 | -0.2592409 | 0.0000000 | 0.0000000 | 0.0000000 | 0.0000000 | 0.0000000 | -1.9718576 | 0.000000e+00 | 40 | 2 | 396.81058 | 346.3702663 |
| 0.5119171 | -1.0352292 | -2.4112333 | -2.1270168 | 0.0000000 | 0.0000000 | 0.0000000 | 0.0000000 | 0.0000000 | 0.0000000 | -0.3814534 | 0.0000000 | 0.0000000 | 0.0000000 | 0.0000000 | 0.0000000 | 0.0000000 | -0.4382583 | 0.0000000 | 0.0000000 | 0.0000000 | -3.9147832 | -1.6152974 | -0.6782728 | 0.000000e+00 | 40 | 3 | 254.85727 | 204.6922102 |
| 4.8117558 | -5.1949367 | 0.0000000 | -3.0699170 | 0.0000000 | -0.8751549 | 0.0000000 | 0.0000000 | 0.0000000 | 0.0000000 | 0.0000000 | 0.0000000 | 0.0000000 | -1.9133192 | 0.0000000 | 0.0000000 | 0.0000000 | -2.5524039 | 0.0000000 | 0.0000000 | -2.1687002 | -4.5575722 | -4.6769338 | 0.0000000 | -5.848032e+00 | 40 | 4 | 100.77937 | 50.7572885 |
| 0.0000000 | 0.0000000 | -1.7451860 | -0.1389066 | -0.1507626 | 0.0000000 | 0.0000000 | -0.0680219 | 0.0000000 | 0.0000000 | -0.0770315 | 0.0000000 | 0.0000000 | -0.4415964 | -0.6181796 | 0.0000000 | 0.0000000 | -0.1475463 | -0.0889961 | 0.0000000 | 0.0000000 | 0.0000000 | 0.0000000 | -1.1845198 | 0.000000e+00 | 41 | 1 | 712.05751 | 660.5389024 |
| 0.1997544 | -0.0732089 | -1.1396450 | -0.8322522 | -0.2670112 | 0.0000000 | 0.0000000 | -0.2598054 | 0.0000000 | 0.0000000 | 0.0000000 | 0.0000000 | 0.0000000 | -1.2937250 | 0.0000000 | 0.0000000 | 0.0000000 | -0.1965601 | 0.0000000 | 0.0000000 | 0.0000000 | 0.0000000 | -1.3424971 | -0.5237008 | 0.000000e+00 | 41 | 2 | 421.91258 | 371.3759382 |
| 1.2523698 | -0.3110995 | -4.4475883 | 0.0000000 | -0.2966357 | 0.0000000 | 0.0000000 | -0.6835950 | 0.0000000 | 0.0000000 | -0.0956892 | 0.0000000 | -0.1357418 | -1.5544270 | -0.5307199 | 0.0000000 | 0.0000000 | 0.0000000 | -0.7177215 | 0.0000000 | 0.0000000 | 0.0000000 | -0.3330621 | -2.4353931 | 0.000000e+00 | 41 | 3 | 188.61701 | 138.4992497 |
| 2.7792255 | -3.4849052 | 0.0000000 | -1.2130704 | -0.8560193 | 0.0000000 | 0.0000000 | -1.7800869 | 0.0000000 | 0.0000000 | 0.0000000 | 0.0000000 | 0.0000000 | -4.7823068 | 0.0000000 | 0.0000000 | 0.0000000 | 0.0000000 | -0.5038536 | 0.0000000 | 0.0000000 | -4.4492010 | -3.1506375 | -0.9979338 | 0.000000e+00 | 41 | 4 | 103.63673 | 53.5852099 |
| 0.0000000 | -8.6611521 | 0.0000000 | -32.5275552 | -2.0825394 | 0.0000000 | 0.0000000 | 0.0000000 | 0.0000000 | 0.0000000 | 0.0000000 | 0.0000000 | 0.0000000 | 0.0000000 | -0.1434110 | 0.0000000 | -0.5992533 | -0.7407177 | 0.0000000 | 0.0000000 | 0.0000000 | 0.0000000 | 0.0000000 | 0.0000000 | 0.000000e+00 | 41 | 5 | 70.68048 | 20.6804792 |
| 2.4583849 | -22.3753328 | 0.0000000 | -3.5210126 | 0.0000000 | 0.0000000 | 0.0000000 | -3.0249516 | 0.0000000 | 0.0000000 | -6.7749303 | 0.0000000 | 0.0000000 | -0.5829124 | 0.0000000 | 0.0000000 | 0.0000000 | 0.0000000 | 0.0000000 | 0.0000000 | 0.0000000 | -8.2770765 | 0.0000000 | 0.0000000 | -2.495469e+04 | 41 | 6 | 58.70858 | 8.7085790 |
| 0.0000000 | 0.0000000 | -1.7451860 | -0.1389066 | -0.1507626 | 0.0000000 | 0.0000000 | -0.0680219 | 0.0000000 | 0.0000000 | -0.0770315 | 0.0000000 | 0.0000000 | -0.4415964 | -0.6181796 | 0.0000000 | 0.0000000 | -0.1475463 | -0.0889961 | 0.0000000 | 0.0000000 | 0.0000000 | 0.0000000 | -1.1845198 | 0.000000e+00 | 42 | 1 | 712.05751 | 660.5389024 |
| 0.5395343 | 0.0000000 | -3.3282845 | -1.7837887 | -0.0109525 | 0.0000000 | 0.0000000 | -0.2898310 | 0.0000000 | -0.5819201 | -0.1723783 | 0.0000000 | -0.4576856 | 0.0000000 | 0.0000000 | 0.0000000 | 0.0000000 | -0.2835871 | 0.0000000 | 0.0000000 | 0.0000000 | 0.0000000 | -0.7967096 | -0.8587971 | 0.000000e+00 | 42 | 2 | 368.25756 | 317.7804912 |
| 0.0000000 | -2.2237365 | 0.0000000 | -1.6602816 | -0.3022968 | 0.0000000 | 0.0000000 | -0.4975490 | 0.0000000 | 0.0000000 | -0.0789699 | 0.0000000 | 0.0000000 | -3.8053758 | 0.0000000 | 0.0000000 | 0.0000000 | -1.5724439 | -0.1635914 | 0.0000000 | 0.0000000 | -3.5779806 | 0.0000000 | -1.2891589 | -1.274929e-01 | 42 | 3 | 196.76602 | 146.6041001 |
| 1.8197771 | -12.4546195 | 0.0000000 | 0.0000000 | -1.3236762 | 0.0000000 | 0.0000000 | -0.3105277 | 0.0000000 | 0.0000000 | 0.0000000 | 0.0000000 | -3.4235580 | -15.2688315 | 0.0000000 | 0.0000000 | 0.0000000 | 0.0000000 | 0.0000000 | 0.0000000 | 0.0000000 | -2.2574998 | -1.2158800 | -2.0848840 | 0.000000e+00 | 42 | 4 | 84.12291 | 34.0861106 |
| 76.7877939 | -87.2808883 | 0.0000000 | 0.0000000 | -1.4129299 | -2.4988756 | 0.0000000 | -5.5966452 | -0.2357167 | 0.0000000 | 0.0000000 | -0.0000193 | -22.3685295 | -15.9836001 | -226.9829712 | 0.0000000 | -1.9658692 | -0.3226636 | -2.6086778 | -179.4439217 | 0.0000000 | 0.0000000 | 0.0000000 | 0.0000000 | -3.099435e+02 | 42 | 5 | 50.00164 | 0.0016365 |
| 63.4975122 | -43.4677244 | 0.0000000 | 0.0000000 | 0.0000000 | -5.4702337 | -0.1101739 | -54.2550541 | 0.0000000 | 0.0000000 | -12.5073566 | -7.4536436 | -1.4064253 | -7.0885311 | -0.3127145 | -3.5037608 | 0.0000000 | 0.0000000 | -16.7870342 | -24.1347928 | -75.0695530 | 0.0000000 | 0.0000000 | 0.0000000 | -1.029879e+02 | 42 | 6 | 50.01472 | 0.0000001 |
| 0.0000000 | 0.0000000 | -1.7451860 | -0.1389066 | -0.1507626 | 0.0000000 | 0.0000000 | -0.0680219 | 0.0000000 | 0.0000000 | -0.0770315 | 0.0000000 | 0.0000000 | -0.4415964 | -0.6181796 | 0.0000000 | 0.0000000 | -0.1475463 | -0.0889961 | 0.0000000 | 0.0000000 | 0.0000000 | 0.0000000 | -1.1845198 | 0.000000e+00 | 43 | 1 | 712.05751 | 660.5389024 |
| 0.7368477 | -0.2990636 | -0.9504725 | -0.8819693 | -0.1182258 | 0.0000000 | 0.0000000 | -0.2466538 | 0.0000000 | 0.0000000 | -1.1977664 | 0.0000000 | 0.0000000 | -0.4083691 | -0.7812079 | 0.0000000 | 0.0000000 | 0.0000000 | 0.0000000 | 0.0000000 | 0.0000000 | 0.0000000 | -1.9563459 | 0.0000000 | 0.000000e+00 | 43 | 2 | 430.47491 | 379.9825250 |
| 0.7646608 | -0.8996705 | -2.2687824 | 0.0000000 | 0.0000000 | 0.0000000 | 0.0000000 | -0.2873921 | 0.0000000 | 0.0000000 | 0.0000000 | 0.0000000 | 0.0000000 | -1.6287001 | 0.0000000 | 0.0000000 | 0.0000000 | -0.6265355 | -0.7487512 | 0.0000000 | 0.0000000 | -0.3908128 | -2.2976083 | 0.0000000 | 0.000000e+00 | 43 | 3 | 225.85010 | 175.7047798 |
| 1.2349786 | 0.0000000 | -5.1400067 | -0.7348281 | 0.0000000 | 0.0000000 | 0.0000000 | -0.6119971 | 0.0000000 | 0.0000000 | -0.6146174 | 0.0000000 | 0.0000000 | -2.1604784 | 0.0000000 | 0.0000000 | 0.0000000 | -2.1576590 | 0.0000000 | 0.0000000 | 0.0000000 | -3.9002545 | -0.8083569 | -0.3174335 | 0.000000e+00 | 43 | 4 | 113.34127 | 63.3044689 |
| 583.3498333 | -2266.3507095 | 0.0000000 | -40.6677869 | -119.4508832 | 0.0000000 | 0.0000000 | -187.1494206 | -1.7918453 | 0.0000000 | -462.8059972 | 0.0000000 | -214.3200230 | -270.0768983 | 0.0000000 | 0.0000000 | -64.4861602 | 0.0000000 | 0.0000000 | 0.0000000 | 0.0000000 | -106.9723113 | 0.0000000 | 0.0000000 | -7.969698e+03 | 43 | 6 | 50.00738 | 0.0000191 |
| 0.0000000 | 0.0000000 | -1.7451860 | -0.1389066 | -0.1507626 | 0.0000000 | 0.0000000 | -0.0680219 | 0.0000000 | 0.0000000 | -0.0770315 | 0.0000000 | 0.0000000 | -0.4415964 | -0.6181796 | 0.0000000 | 0.0000000 | -0.1475463 | -0.0889961 | 0.0000000 | 0.0000000 | 0.0000000 | 0.0000000 | -1.1845198 | 0.000000e+00 | 44 | 1 | 712.05751 | 660.5389024 |
| 0.3049771 | -0.3505279 | -2.6927063 | -0.7275934 | 0.0000000 | 0.0000000 | 0.0000000 | 0.0000000 | 0.0000000 | 0.0000000 | -0.1622718 | 0.0000000 | -0.4563406 | -1.2794029 | 0.0000000 | 0.0000000 | 0.0000000 | -0.3068180 | -0.4843019 | 0.0000000 | 0.0000000 | 0.0000000 | -0.2040818 | -1.5545833 | 0.000000e+00 | 44 | 2 | 410.19851 | 359.8090381 |
| 0.5507055 | -1.8648975 | -0.8276327 | -2.7340309 | -1.0144468 | 0.0000000 | 0.0000000 | 0.0000000 | 0.0000000 | 0.0000000 | -0.4147777 | 0.0000000 | 0.0000000 | -1.7229867 | -0.7342270 | 0.0000000 | 0.0000000 | -1.9705798 | 0.0000000 | 0.0000000 | 0.0000000 | 0.0000000 | 0.0000000 | -0.5986366 | 0.000000e+00 | 44 | 3 | 238.64073 | 188.5181810 |
| 4.6321090 | -6.0171978 | -1.7749399 | 0.0000000 | -0.7661430 | -0.8059661 | 0.0000000 | -1.5436226 | 0.0000000 | 0.0000000 | 0.0000000 | -0.2214178 | 0.0000000 | 0.0000000 | -4.5134588 | 0.0000000 | 0.0000000 | 0.0000000 | -2.0494171 | 0.0000000 | 0.0000000 | 0.0000000 | -3.3128826 | 0.0000000 | -3.657948e+00 | 44 | 4 | 83.18896 | 33.1668839 |
| 11.3205936 | -10.9991112 | 0.0000000 | 0.0000000 | -3.9031080 | 0.0000000 | 0.0000000 | -0.5280654 | 0.0000000 | 0.0000000 | -1.8570398 | 0.0000000 | 0.0000000 | -14.4557713 | 0.0000000 | 0.0000000 | 0.0000000 | 0.0000000 | -1.2241429 | 0.0000000 | -12.7725469 | -41.5725896 | 0.0000000 | -4.3167879 | -1.785507e+01 | 44 | 6 | 65.59574 | 15.5883793 |
| 0.0000000 | 0.0000000 | -1.7451860 | -0.1389066 | -0.1507626 | 0.0000000 | 0.0000000 | -0.0680219 | 0.0000000 | 0.0000000 | -0.0770315 | 0.0000000 | 0.0000000 | -0.4415964 | -0.6181796 | 0.0000000 | 0.0000000 | -0.1475463 | -0.0889961 | 0.0000000 | 0.0000000 | 0.0000000 | 0.0000000 | -1.1845198 | 0.000000e+00 | 45 | 1 | 712.05751 | 660.5389024 |
| 0.6623532 | -0.4811466 | -1.5282646 | 0.0000000 | -0.3443774 | 0.0000000 | 0.0000000 | -0.2477470 | 0.0000000 | 0.0000000 | -0.1277561 | 0.0000000 | 0.0000000 | -1.1521868 | -0.7769056 | 0.0000000 | 0.0000000 | -0.4289096 | 0.0000000 | 0.0000000 | 0.0000000 | 0.0000000 | -1.1525178 | -0.7569759 | 0.000000e+00 | 45 | 2 | 394.65661 | 344.2045130 |
| 1.1599636 | -1.6783448 | -2.3206931 | -0.4036816 | -0.7172097 | 0.0000000 | 0.0000000 | -1.2644461 | 0.0000000 | 0.0000000 | 0.0000000 | 0.0000000 | 0.0000000 | -6.5033249 | 0.0000000 | 0.0000000 | 0.0000000 | -1.0118772 | 0.0000000 | 0.0000000 | 0.0000000 | 0.0000000 | 0.0000000 | -1.2164045 | -1.744272e+00 | 45 | 3 | 192.84028 | 142.7036891 |
| 5.4856163 | -4.2352509 | 0.0000000 | -3.4409036 | -2.6046780 | 0.0000000 | 0.0000000 | -2.1118480 | 0.0000000 | 0.0000000 | 0.0000000 | 0.0000000 | -4.0399622 | -6.8079058 | 0.0000000 | 0.0000000 | 0.0000000 | -2.8063583 | -2.1368798 | 0.0000000 | 0.0000000 | 0.0000000 | -1.2919402 | -1.0281661 | 0.000000e+00 | 45 | 4 | 93.83135 | 43.8239945 |
| 0.8606081 | -8.7927870 | 0.0000000 | -13.3533268 | -2.2432258 | 0.0000000 | 0.0000000 | 0.0000000 | 0.0000000 | 0.0000000 | -0.3928075 | 0.0000000 | 0.0000000 | 0.0000000 | 0.0000000 | 0.0000000 | 0.0000000 | -1.8467024 | 0.0000000 | 0.0000000 | 0.0000000 | 0.0000000 | 0.0000000 | 0.0000000 | 0.000000e+00 | 45 | 5 | 90.15921 | 40.1518488 |
| 0.0000000 | 0.0000000 | -1.7451860 | -0.1389066 | -0.1507626 | 0.0000000 | 0.0000000 | -0.0680219 | 0.0000000 | 0.0000000 | -0.0770315 | 0.0000000 | 0.0000000 | -0.4415964 | -0.6181796 | 0.0000000 | 0.0000000 | -0.1475463 | -0.0889961 | 0.0000000 | 0.0000000 | 0.0000000 | 0.0000000 | -1.1845198 | 0.000000e+00 | 46 | 1 | 712.05751 | 660.5389024 |
| 0.6759383 | 0.0000000 | -2.4016221 | 0.0000000 | -0.1486389 | 0.0000000 | 0.0000000 | -0.2274565 | 0.0000000 | 0.0000000 | 0.0000000 | 0.0000000 | -0.0565563 | -1.9606494 | -0.2953771 | 0.0000000 | 0.0000000 | -0.6410767 | -0.5183921 | 0.0000000 | 0.0000000 | 0.0000000 | -0.6754799 | -0.7065167 | 0.000000e+00 | 46 | 2 | 419.64730 | 369.1747903 |
| 1.1665046 | -0.9877640 | -3.2546908 | -0.2175704 | -0.1136016 | 0.0000000 | 0.0000000 | 0.0000000 | 0.0000000 | 0.0000000 | -0.9604994 | 0.0000000 | 0.0000000 | -2.1620569 | 0.0000000 | 0.0000000 | -0.1811459 | 0.0000000 | -1.0072930 | 0.0000000 | 0.0000000 | 0.0000000 | -0.4924586 | -2.9743130 | 0.000000e+00 | 46 | 3 | 208.07222 | 157.9817491 |
| 10.4334267 | -0.8546603 | -2.9716346 | -3.0548660 | -2.7946205 | 0.0000000 | 0.0000000 | 0.0000000 | 0.0000000 | 0.0000000 | 0.0000000 | 0.0000000 | 0.0000000 | -4.9254261 | -2.3397209 | 0.0000000 | 0.0000000 | -2.0638848 | -0.7878223 | 0.0000000 | 0.0000000 | 0.0000000 | -9.3085989 | 0.0000000 | 0.000000e+00 | 46 | 4 | 78.70883 | 28.6793946 |
| 20.4835454 | 0.0000000 | -19.8390407 | -5.5250693 | 0.0000000 | 0.0000000 | 0.0000000 | -0.9402384 | 0.0000000 | 0.0000000 | -2.2244177 | 0.0000000 | 0.0000000 | 0.0000000 | 0.0000000 | 0.0000000 | -0.6478634 | -2.0959239 | 0.0000000 | 0.0000000 | 0.0000000 | -11.4812828 | -15.2353298 | 0.0000000 | -2.176992e+04 | 46 | 5 | 58.49366 | 8.4789441 |
| 0.0000000 | 0.0000000 | -1.7451860 | -0.1389066 | -0.1507626 | 0.0000000 | 0.0000000 | -0.0680219 | 0.0000000 | 0.0000000 | -0.0770315 | 0.0000000 | 0.0000000 | -0.4415964 | -0.6181796 | 0.0000000 | 0.0000000 | -0.1475463 | -0.0889961 | 0.0000000 | 0.0000000 | 0.0000000 | 0.0000000 | -1.1845198 | 0.000000e+00 | 47 | 1 | 712.05751 | 660.5389024 |
| 0.0006396 | -0.0074367 | -1.8479086 | -0.4087434 | -0.1157374 | 0.0000000 | 0.0000000 | -0.0301817 | 0.0000000 | 0.0000000 | 0.0000000 | 0.0000000 | 0.0000000 | -1.3701235 | 0.0000000 | 0.0000000 | 0.0000000 | -0.3514109 | -0.1577756 | 0.0000000 | 0.0000000 | 0.0000000 | -0.8627762 | -0.8868389 | 0.000000e+00 | 47 | 2 | 417.24472 | 366.7585023 |
| 1.1503188 | -2.6895486 | -1.8545976 | 0.0000000 | 0.0000000 | 0.0000000 | 0.0000000 | 0.0000000 | 0.0000000 | 0.0000000 | -1.5570854 | 0.0000000 | 0.0000000 | -1.3013742 | -1.8860532 | 0.0000000 | 0.0000000 | 0.0000000 | -0.9438153 | 0.0000000 | 0.0000000 | 0.0000000 | -1.3848770 | -1.7136272 | 0.000000e+00 | 47 | 3 | 154.24687 | 104.1766239 |
| 2.5252768 | -1.2838458 | -2.7732410 | -2.8631246 | 0.0000000 | 0.0000000 | 0.0000000 | 0.0000000 | 0.0000000 | 0.0000000 | -1.5860329 | 0.0000000 | 0.0000000 | -2.8942656 | 0.0000000 | 0.0000000 | 0.0000000 | -3.8134687 | 0.0000000 | 0.0000000 | -3.3087631 | -0.3734771 | 0.0000000 | -0.5484450 | 0.000000e+00 | 47 | 4 | 119.21218 | 69.1459403 |
| 1.2512832 | -2.4984730 | -2.2062934 | -0.5518187 | -1.4133063 | 0.0000000 | 0.0000000 | 0.0000000 | 0.0000000 | 0.0000000 | 0.0000000 | 0.0000000 | 0.0000000 | -5.2447501 | 0.0000000 | 0.0000000 | -2.5927539 | 0.0000000 | 0.0000000 | 0.0000000 | 0.0000000 | -0.8472142 | 0.0000000 | -3.7008738 | -1.644567e+05 | 47 | 5 | 69.42804 | 19.4206771 |
| 0.0000000 | 0.0000000 | -1.7451860 | -0.1389066 | -0.1507626 | 0.0000000 | 0.0000000 | -0.0680219 | 0.0000000 | 0.0000000 | -0.0770315 | 0.0000000 | 0.0000000 | -0.4415964 | -0.6181796 | 0.0000000 | 0.0000000 | -0.1475463 | -0.0889961 | 0.0000000 | 0.0000000 | 0.0000000 | 0.0000000 | -1.1845198 | 0.000000e+00 | 48 | 1 | 712.05751 | 660.5389024 |
| 0.0000000 | -0.3151318 | -0.9334845 | -0.4917052 | -0.1577567 | 0.0000000 | 0.0000000 | -0.3242250 | 0.0000000 | 0.0000000 | 0.0000000 | 0.0000000 | 0.0000000 | -2.6216746 | 0.0000000 | 0.0000000 | 0.0000000 | -0.5657840 | -0.6770961 | 0.0000000 | 0.0000000 | 0.0000000 | -0.2060851 | -1.1021740 | 0.000000e+00 | 48 | 2 | 411.84215 | 361.4022040 |
| 1.8754222 | -4.0260816 | 0.0000000 | -2.0499826 | 0.0000000 | -0.1189190 | 0.0000000 | 0.0000000 | 0.0000000 | 0.0000000 | -0.6029544 | 0.0000000 | 0.0000000 | -0.8875192 | -0.2126698 | -0.0845318 | 0.0000000 | 0.0000000 | -1.8556227 | 0.0000000 | 0.0000000 | 0.0000000 | -3.6289295 | 0.0000000 | 0.000000e+00 | 48 | 3 | 197.00283 | 146.8670522 |
| 3.1156833 | 0.0000000 | -5.6781760 | -1.0948069 | 0.0000000 | -0.8769789 | 0.0000000 | 0.0000000 | 0.0000000 | 0.0000000 | 0.0000000 | 0.0000000 | 0.0000000 | -3.0772657 | 0.0000000 | 0.0000000 | 0.0000000 | 0.0000000 | 0.0000000 | 0.0000000 | 0.0000000 | -3.0565313 | -2.0174784 | -4.5554398 | 0.000000e+00 | 48 | 4 | 102.20348 | 52.1519608 |
| 0.0000000 | 0.0000000 | -1.7451860 | -0.1389066 | -0.1507626 | 0.0000000 | 0.0000000 | -0.0680219 | 0.0000000 | 0.0000000 | -0.0770315 | 0.0000000 | 0.0000000 | -0.4415964 | -0.6181796 | 0.0000000 | 0.0000000 | -0.1475463 | -0.0889961 | 0.0000000 | 0.0000000 | 0.0000000 | 0.0000000 | -1.1845198 | 0.000000e+00 | 49 | 1 | 712.05751 | 660.5389024 |
| 0.1586024 | 0.0000000 | -2.8422909 | -0.3844149 | 0.0000000 | 0.0000000 | 0.0000000 | -0.0263368 | 0.0000000 | 0.0000000 | -0.0589073 | 0.0000000 | 0.0000000 | -1.8401331 | -0.0945184 | 0.0000000 | 0.0000000 | -1.2485251 | -0.2027859 | 0.0000000 | 0.0000000 | 0.0000000 | -0.0135681 | -0.7080201 | 0.000000e+00 | 49 | 2 | 420.28976 | 369.8725523 |
| 1.6045543 | -1.0218066 | -3.3264012 | -0.3722865 | -0.6027263 | 0.0000000 | 0.0000000 | 0.0000000 | 0.0000000 | 0.0000000 | 0.0000000 | 0.0000000 | 0.0000000 | -1.2437996 | -0.6564146 | 0.0000000 | 0.0000000 | 0.0000000 | -0.9941298 | 0.0000000 | 0.0000000 | 0.0000000 | -1.3608689 | -1.9077176 | 0.000000e+00 | 49 | 3 | 237.64867 | 187.5824318 |
| 0.5522432 | -1.3417258 | -0.2237646 | -3.5810754 | -0.0341713 | 0.0000000 | 0.0000000 | -0.8251944 | 0.0000000 | 0.0000000 | 0.0000000 | 0.0000000 | 0.0000000 | -1.2532052 | 0.0000000 | 0.0000000 | 0.0000000 | -2.8016937 | 0.0000000 | 0.0000000 | -4.2627951 | -4.0476771 | -0.0887334 | 0.0000000 | 0.000000e+00 | 49 | 4 | 98.06829 | 48.0314927 |
| 14.3519283 | 0.0000000 | -14.1806129 | -3.1240872 | -2.9897395 | 0.0000000 | 0.0000000 | 0.0000000 | 0.0000000 | -6.4276788 | 0.0000000 | 0.0000000 | 0.0000000 | 0.0000000 | 0.0000000 | 0.0000000 | -4.6412594 | 0.0000000 | 0.0000000 | 0.0000000 | 0.0000000 | -6.8542786 | -8.3025964 | -3.0482868 | 0.000000e+00 | 49 | 5 | 65.10570 | 15.0983413 |
| 19.4801683 | -20.4798834 | 0.0000000 | -4.2436931 | -14.2085808 | 0.0000000 | -1.1417677 | -24.1718005 | 0.0000000 | 0.0000000 | -10.1788049 | 0.0000000 | 0.0000000 | -9.3704222 | -0.4186963 | 0.0000000 | 0.0000000 | -1.1473531 | -14.6441406 | -9.3353688 | 0.0000000 | 0.0000000 | 0.0000000 | -0.3329272 | -2.492600e+00 | 49 | 6 | 50.00736 | 0.0000000 |
| 0.0000000 | 0.0000000 | -1.7451860 | -0.1389066 | -0.1507626 | 0.0000000 | 0.0000000 | -0.0680219 | 0.0000000 | 0.0000000 | -0.0770315 | 0.0000000 | 0.0000000 | -0.4415964 | -0.6181796 | 0.0000000 | 0.0000000 | -0.1475463 | -0.0889961 | 0.0000000 | 0.0000000 | 0.0000000 | 0.0000000 | -1.1845198 | 0.000000e+00 | 50 | 1 | 712.05751 | 660.5389024 |
| 0.5246830 | -0.3393570 | -1.4957001 | -0.0081722 | -0.2812779 | 0.0000000 | 0.0000000 | -0.1826874 | 0.0000000 | 0.0000000 | 0.0000000 | 0.0000000 | 0.0000000 | -1.6017339 | -0.5919418 | 0.0000000 | 0.0000000 | -0.2428412 | 0.0000000 | 0.0000000 | 0.0000000 | 0.0000000 | -1.1003718 | -0.9349493 | 0.000000e+00 | 50 | 2 | 398.72121 | 348.1713578 |
| 1.3261147 | -2.4697504 | -0.6939601 | -3.4962988 | -0.7576194 | 0.0000000 | 0.0000000 | -0.0371405 | 0.0000000 | 0.0000000 | 0.0000000 | 0.0000000 | -1.3992806 | 0.0000000 | 0.0000000 | 0.0000000 | 0.0000000 | 0.0000000 | -1.3194010 | 0.0000000 | 0.0000000 | -1.0661652 | -1.7026308 | -1.6247242 | 0.000000e+00 | 50 | 3 | 189.59344 | 139.4891983 |
| 1.7221946 | -14.8325673 | 0.0000000 | 0.0000000 | -0.6248016 | 0.0000000 | 0.0000000 | 0.0000000 | 0.0000000 | 0.0000000 | 0.0000000 | 0.0000000 | -0.0732659 | -7.4059788 | 0.0000000 | 0.0000000 | 0.0000000 | 0.0000000 | 0.0000000 | 0.0000000 | 0.0000000 | 0.0000000 | -3.0203934 | 0.0000000 | -5.273756e+00 | 50 | 4 | 116.71090 | 66.6814560 |
| 0.0000000 | -13.4734699 | 0.0000000 | 0.0000000 | -0.5340847 | 0.0000000 | 0.0000000 | 0.0000000 | 0.0000000 | 0.0000000 | 0.0000000 | 0.0000000 | 0.0000000 | -54.9713252 | 0.0000000 | 0.0000000 | 0.0000000 | -2.2491161 | 0.0000000 | 0.0000000 | 0.0000000 | 0.0000000 | -0.6374131 | 0.0000000 | 0.000000e+00 | 50 | 5 | 60.57915 | 10.5791468 |
