# Supplementary material for: Tropical Land‐Use Change Disrupts Zeta‐Diversity Across Taxa
Source: Glob Chang Biol. 2025 May 14;31(5):e70245. doi: 10.1111/gcb.70245 (PMC12076182; doi:10.1111/gcb.70245)
Supplement: Supplementary file 1 — Data S1. [file GCB-31-e70245-s001.zip › gcb70245-sup-0001-Appendix1.zip!/SM-6-MS_Orchid_forest.html]

table output


MS-GDM Coefficients for orchid communities in natural habitats from 50 iterations (Binomial family (link=log) for each order of zeta and each predictor, using a random sample of 1000 site combinations for each MS-GDM. Here are the 50 replicates for each predictor, AIC, and deviance.

| (Intercept) | Elevation1 | Elevation2 | Elevation3 | Canopy.cover1 | Canopy.cover2 | Canopy.cover3 | Tree.density1 | Tree.density2 | Tree.density3 | Fragmentation1 | Fragmentation2 | Fragmentation3 | Forest.cover1 | Forest.cover2 | Forest.cover3 | Precipitation1 | Precipitation2 | Precipitation3 | Disturbance1 | Disturbance2 | Disturbance3 | distance1 | distance2 | distance3 | Rep | Group | AIC | Residual\_Deviance |
| --- | --- | --- | --- | --- | --- | --- | --- | --- | --- | --- | --- | --- | --- | --- | --- | --- | --- | --- | --- | --- | --- | --- | --- | --- | --- | --- | --- | --- |
| 0.0000000 | -2.1160163 | -1.6219091 | -0.4175285 | -0.5003901 | -0.7817445 | -0.3676415 | 0.0000000 | -1.7048838 | -0.2197804 | -0.8469415 | 0.0000000 | -3.3837119 | 0.0000000 | 0.000000 | 0.0000000 | -0.9555855 | -0.5636922 | 0.0000000 | -0.3285607 | -1.2171728 | 0.0000000 | -1.1343690 | 0.0000000 | -1.422999e-01 | 1 | 1 | 229.39423 | 178.2193820 |
| 0.0000000 | -1.5102074 | -2.4685872 | -2.1942863 | 0.0000000 | 0.0000000 | -0.7423133 | -1.2044038 | -1.6841238 | 0.0000000 | 0.0000000 | -0.5656756 | 0.0000000 | 0.0000000 | 0.000000 | 0.0000000 | -2.1991147 | 0.0000000 | -0.6029397 | 0.0000000 | -1.3245238 | -0.3010311 | -0.9478639 | 0.0000000 | -1.174425e+00 | 2 | 1 | 215.02860 | 163.8032435 |
| 0.0000000 | -1.0818369 | -2.2772493 | 0.0000000 | 0.0000000 | -0.9565068 | -0.4686012 | 0.0000000 | -2.3812070 | -0.2040249 | -0.5950625 | 0.0000000 | -0.3317533 | 0.0000000 | 0.000000 | 0.0000000 | -1.7212341 | -0.0754055 | -0.3878923 | -0.0612439 | -1.3879660 | -0.2953540 | -1.1018625 | 0.0000000 | 0.000000e+00 | 3 | 1 | 211.14013 | 160.1283836 |
| 0.0000000 | -2.5978893 | -1.4922638 | -0.0820374 | -0.3337385 | -0.3268886 | -1.7344943 | 0.0000000 | -1.4115965 | -0.8330687 | -0.5316440 | -1.3918242 | -5.7270349 | 0.0000000 | 0.000000 | 0.0000000 | -1.1746941 | 0.0000000 | -0.1540891 | 0.0000000 | -1.0312661 | -0.1031723 | -1.3331749 | 0.0000000 | 0.000000e+00 | 4 | 1 | 208.84722 | 157.6898212 |
| 0.0000000 | -0.6160833 | -2.5262065 | -0.1442050 | -0.3323199 | 0.0000000 | -1.1034559 | -3.6343224 | -0.9940775 | -0.4920222 | -0.5655359 | 0.0000000 | -3.0652918 | 0.0000000 | 0.000000 | 0.0000000 | -1.3220717 | 0.0000000 | -0.3345598 | -0.3163259 | -0.8419207 | 0.0000000 | -1.4462655 | 0.0000000 | 0.000000e+00 | 5 | 1 | 213.94614 | 162.7392291 |
| 0.0000000 | 0.0000000 | -3.4987467 | -0.7947293 | -0.8223687 | -0.7851575 | -2.0915772 | -1.9959859 | -1.1394041 | -1.4221545 | -0.3976894 | -0.1957470 | -0.7222634 | 0.0000000 | 0.000000 | 0.0000000 | -1.0098712 | 0.0000000 | 0.0000000 | 0.0000000 | -1.3683859 | 0.0000000 | -0.9161262 | 0.0000000 | -6.587343e-01 | 6 | 1 | 203.32259 | 152.2525897 |
| 0.0000000 | -2.6187693 | -2.2320488 | 0.0000000 | 0.0000000 | -0.6919111 | -0.5398616 | -0.9281687 | -0.9789097 | -0.1280938 | 0.0000000 | -1.7476862 | -1.5555850 | -0.0795673 | 0.000000 | 0.0000000 | -1.2392308 | 0.0000000 | 0.0000000 | -0.0101629 | -0.9275748 | -0.4246242 | -1.6129304 | 0.0000000 | 0.000000e+00 | 7 | 1 | 218.13086 | 167.0533536 |
| 0.0000000 | -0.3514266 | -1.6257326 | -1.4465140 | 0.0000000 | -0.3110195 | -1.0492515 | -1.2842404 | -1.3936360 | 0.0000000 | -0.0117254 | 0.0000000 | -1.5447581 | 0.0000000 | 0.000000 | 0.0000000 | -1.1720188 | -0.4387515 | 0.0000000 | -0.0688441 | -1.9699158 | -0.5097294 | -1.4596398 | 0.0000000 | 0.000000e+00 | 8 | 1 | 212.50920 | 161.4514565 |
| 0.0000000 | -1.8035531 | -3.4266554 | -0.0016128 | 0.0000000 | -0.8033836 | -0.3920825 | -0.5272762 | -2.0786190 | 0.0000000 | -0.2959668 | 0.0000000 | -1.8832179 | 0.0000000 | 0.000000 | 0.0000000 | -1.2242386 | 0.0000000 | -0.1551454 | 0.0000000 | -0.8779998 | -0.4531390 | -1.2396431 | 0.0000000 | -1.004702e+00 | 9 | 1 | 204.67798 | 153.6372708 |
| 0.0000000 | -0.4332576 | -3.1748252 | 0.0000000 | -0.5113357 | -0.4033628 | -1.1382805 | -1.2192054 | -2.0611788 | 0.0000000 | -0.0524469 | -1.6146402 | 0.0000000 | -0.0756641 | 0.000000 | 0.0000000 | -0.5190004 | -0.1688270 | 0.0000000 | 0.0000000 | -0.2651588 | 0.0000000 | -1.2412613 | 0.0000000 | -9.031414e-01 | 10 | 1 | 244.21218 | 193.0968201 |
| 0.0000000 | -2.2615803 | 0.0000000 | 0.0000000 | -1.1327541 | 0.0000000 | -1.9783922 | -1.6733127 | -2.9473605 | -3.7017840 | -0.5991010 | 0.0000000 | -6.2217565 | 0.0000000 | 0.000000 | 0.0000000 | -3.1526755 | -0.8771974 | 0.0000000 | 0.0000000 | -2.0198729 | -0.2882429 | -2.1351391 | 0.0000000 | 0.000000e+00 | 1 | 2 | 68.19889 | 18.1510627 |
| 0.0000000 | -6.1649225 | -4.2379471 | 0.0000000 | -1.0752223 | -2.2593090 | -4.0388167 | -0.1668755 | -0.4569102 | -2.2329496 | -0.1612198 | 0.0000000 | -1.6529307 | -2.4038653 | 0.000000 | 0.0000000 | 0.0000000 | 0.0000000 | 0.0000000 | 0.0000000 | 0.0000000 | 0.0000000 | -2.2354322 | 0.0000000 | 0.000000e+00 | 2 | 2 | 81.49510 | 31.4061962 |
| 0.3963302 | -6.0403582 | 0.0000000 | -10.7883777 | -23.7571670 | -0.9657764 | -2.3366669 | 0.0000000 | 0.0000000 | 0.0000000 | 0.0000000 | 0.0000000 | 0.0000000 | 0.0000000 | 0.000000 | 0.0000000 | 0.0000000 | -2.4842235 | -1.0279815 | 0.0000000 | -2.4088310 | -3.5922301 | -2.1682182 | 0.0000000 | 0.000000e+00 | 3 | 2 | 65.64097 | 15.5849942 |
| 0.0000000 | 0.0000000 | -1.7506395 | -6.3054447 | 0.0000000 | -6.8812938 | -1.2798995 | 0.0000000 | -2.4308541 | 0.0000000 | 0.0000000 | 0.0000000 | 0.0000000 | 0.0000000 | 0.000000 | 0.0000000 | -1.7611809 | -1.2089256 | 0.0000000 | -2.2189328 | -0.7807841 | 0.0000000 | -1.3684658 | 0.0000000 | -1.169563e+05 | 4 | 2 | 67.86633 | 17.7906048 |
| 0.0000000 | 0.0000000 | -3.8389501 | 0.0000000 | 0.0000000 | 0.0000000 | -5.1736345 | -1.1311013 | 0.0000000 | 0.0000000 | -1.7250618 | -0.9615539 | -5.8080650 | 0.0000000 | -3.706171 | 0.0000000 | -0.4714936 | 0.0000000 | -1.5094053 | -3.7996309 | -1.1347577 | 0.0000000 | -1.4178040 | 0.0000000 | -5.576770e+01 | 5 | 2 | 56.77378 | 6.7259148 |
| 0.0000000 | -6.1040671 | -1.4652214 | -12.8513494 | -30.0868323 | 0.0000000 | -0.5109131 | -0.1962139 | -0.1745088 | 0.0000000 | -0.0493355 | -3.4893751 | 0.0000000 | 0.0000000 | 0.000000 | -2.5087820 | -2.8839218 | 0.0000000 | 0.0000000 | -2.6489368 | -2.6592744 | 0.0000000 | -1.6130672 | 0.0000000 | 0.000000e+00 | 6 | 2 | 62.76664 | 12.7232939 |
| 0.0000000 | 0.0000000 | -2.7689864 | 0.0000000 | -11.8194897 | 0.0000000 | -2.1048611 | 0.0000000 | -0.8844865 | -2.2735672 | 0.0000000 | 0.0000000 | -5.5509264 | 0.0000000 | 0.000000 | 0.0000000 | -0.4305181 | 0.0000000 | 0.0000000 | -0.4620238 | -1.3026289 | -2.6093156 | -2.3175283 | 0.0000000 | -8.489428e-01 | 7 | 2 | 71.45011 | 21.3462101 |
| 0.0000000 | -1.0148951 | -3.0140792 | -0.6374442 | -26.0875216 | 0.0000000 | -3.1132788 | -0.0427956 | -3.4379357 | 0.0000000 | -0.4796994 | 0.0000000 | -0.5462559 | 0.0000000 | 0.000000 | 0.0000000 | -2.1869001 | 0.0000000 | -2.7898490 | 0.0000000 | -0.9765124 | 0.0000000 | -0.5206270 | 0.0000000 | -1.417740e+00 | 8 | 2 | 70.99063 | 20.8855171 |
| 0.0000000 | -1.0071209 | -4.3900600 | -3.6192064 | 0.0000000 | -0.8259772 | -3.4362809 | -2.7332721 | 0.0000000 | -3.3967419 | 0.0000000 | 0.0000000 | -18.0804388 | 0.0000000 | 0.000000 | 0.0000000 | -1.4281531 | 0.0000000 | -0.5223121 | 0.0000000 | -2.8227644 | 0.0000000 | -0.3401471 | -0.6187734 | 0.000000e+00 | 9 | 2 | 73.05573 | 22.9162929 |
| 0.0000000 | -5.5847332 | 0.0000000 | 0.0000000 | -1.0025443 | -3.5698251 | -1.3715686 | -5.7080287 | -1.2507038 | -0.4816534 | -1.5135882 | 0.0000000 | 0.0000000 | 0.0000000 | 0.000000 | 0.0000000 | -4.8945721 | 0.0000000 | 0.0000000 | 0.0000000 | -0.6082025 | 0.0000000 | -3.0791894 | 0.0000000 | 0.000000e+00 | 10 | 2 | 66.46353 | 16.4056209 |
| 22.2285262 | -54.3690652 | -42.7601765 | -23.1304967 | 0.0000000 | -21.2740956 | -72.9033425 | -62.5865465 | 0.0000000 | -86.0268495 | 0.0000000 | 0.0000000 | -14.5702245 | 0.0000000 | 0.000000 | 0.0000000 | -137.0832323 | 0.0000000 | 0.0000000 | -7.4153117 | 0.0000000 | 0.0000000 | 0.0000000 | 0.0000000 | -4.793576e+01 | 1 | 3 | 50.00643 | 0.0000002 |
| 29.5523913 | -856.9314608 | 0.0000000 | -536.9820923 | 0.0000000 | 0.0000000 | -17.6861619 | 0.0000000 | -0.6503953 | -17.8093356 | -0.3568842 | -6.8921407 | -617.0038535 | -0.8307676 | 0.000000 | -0.1622415 | 0.0000000 | 0.0000000 | -1.0185053 | -24.6874525 | -5.6498961 | -0.2467433 | 0.0000000 | -35.9923516 | 0.000000e+00 | 2 | 3 | 50.00707 | 0.0000008 |
| 45.8088885 | -351.3736110 | -0.1443086 | -287.5265525 | -16.6746364 | -17.9047148 | -10.2057057 | -120.0148980 | 0.0000000 | 0.0000000 | 0.0000000 | -11.3825632 | -1077.8754393 | -27.3331714 | -48.627290 | 0.0000000 | -50.1430410 | 0.0000000 | 0.0000000 | -185.3372208 | -4.6867737 | 0.0000000 | 0.0000000 | -57.3428820 | 0.000000e+00 | 5 | 3 | 50.00707 | 0.0000007 |
| 56.3876195 | -385.4477422 | -12.2078470 | 0.0000000 | 0.0000000 | -256.3187468 | -133.4643372 | -175.3483264 | 0.0000000 | 0.0000000 | -16.2737841 | 0.0000000 | -11.4226774 | 0.0000000 | 0.000000 | -7.5581725 | -133.3318911 | 0.0000000 | 0.0000000 | -3.5827729 | 0.0000000 | 0.0000000 | 0.0000000 | 0.0000000 | -3.096686e+02 | 6 | 3 | 50.00707 | 0.0000005 |
| 63.6404566 | -97.9307572 | 0.0000000 | 0.0000000 | -684.4540888 | -0.1572790 | 0.0000000 | -190.7478710 | -5.1854710 | 0.0000000 | -2.6032784 | 0.0000000 | -2490.9452440 | -2822.5713565 | 0.000000 | -50.3963517 | -7.0301267 | 0.0000000 | -0.0217811 | -300.9160833 | 0.0000000 | -21.2623377 | 0.0000000 | -78.4975444 | 0.000000e+00 | 7 | 3 | 50.00692 | 0.0000080 |
| 105.9191524 | -237.4955797 | 0.0000000 | -732.6550976 | 0.0000000 | 0.0000000 | 0.0000000 | -3331.4434119 | 0.0000000 | 0.0000000 | -236.1962219 | -73.7849068 | -588.7737359 | 0.0000000 | 0.000000 | 0.0000000 | 0.0000000 | 0.0000000 | -284.1901451 | 0.0000000 | 0.0000000 | 0.0000000 | 0.0000000 | -160.5375593 | 0.000000e+00 | 8 | 3 | 50.00693 | 0.0000117 |
| 68.4293514 | -187.1485493 | 0.0000000 | -107.4218898 | 0.0000000 | 0.0000000 | 0.0000000 | -272.1015779 | -126.0448602 | 0.0000000 | -2.2563974 | 0.0000000 | 0.0000000 | 0.0000000 | 0.000000 | -8.5795378 | -182.2294989 | 0.0000000 | 0.0000000 | 0.0000000 | -65.9106090 | -10.0311097 | 0.0000000 | 0.0000000 | -2.930256e+02 | 9 | 3 | 50.00634 | 0.0000004 |
