# Supplementary material for: Tropical Land‐Use Change Disrupts Zeta‐Diversity Across Taxa
Source: Glob Chang Biol. 2025 May 14;31(5):e70245. doi: 10.1111/gcb.70245 (PMC12076182; doi:10.1111/gcb.70245)
Supplement: Supplementary file 1 — Data S1. [file GCB-31-e70245-s001.zip › gcb70245-sup-0001-Appendix1.zip!/SM-7-MS_Orchid_pastures.html]

table output


MS-GDM Coefficients for orchid communities in transformed habitats from 50 iterations (Binomial family (link=log) for each order of zeta and each predictor, using a random sample of 1000 site combinations for each MS-GDM. Here are the 50 replicates for each predictor, AIC, and deviance.

| (Intercept) | Elevation1 | Elevation2 | Elevation3 | Canopy.cover1 | Canopy.cover2 | Canopy.cover3 | Tree.density1 | Tree.density2 | Tree.density3 | Fragmentation1 | Fragmentation2 | Fragmentation3 | Forest.cover1 | Forest.cover2 | Forest.cover3 | Precipitation1 | Precipitation2 | Precipitation3 | Disturbance1 | Disturbance2 | Disturbance3 | distance1 | distance2 | distance3 | Rep | Group | AIC | Residual\_Deviance |
| --- | --- | --- | --- | --- | --- | --- | --- | --- | --- | --- | --- | --- | --- | --- | --- | --- | --- | --- | --- | --- | --- | --- | --- | --- | --- | --- | --- | --- |
| 0.0000000 | -1.4858397 | -3.4021625 | 0.0000000 | -0.3622659 | 0.000000 | -0.2075655 | -0.3815435 | -1.2616693 | 0.0000000 | -0.2545716 | -0.8325255 | 0.000000 | 0.0000000 | 0.0000000 | -2.0915120 | -0.4524391 | -0.4063305 | 0.0000000 | 0.0000000 | -0.1817396 | 0.0000000 | -0.7607695 | 0.0000000 | 0.000000 | 1 | 1 | 228.54149 | 178.302898 |
| 0.0000000 | -1.4858397 | -3.4021625 | 0.0000000 | -0.3622659 | 0.000000 | -0.2075655 | -0.3815435 | -1.2616693 | 0.0000000 | -0.2545716 | -0.8325255 | 0.000000 | 0.0000000 | 0.0000000 | -2.0915120 | -0.4524391 | -0.4063305 | 0.0000000 | 0.0000000 | -0.1817396 | 0.0000000 | -0.7607695 | 0.0000000 | 0.000000 | 2 | 1 | 228.54149 | 178.302898 |
| 0.0000000 | -1.4858397 | -3.4021625 | 0.0000000 | -0.3622659 | 0.000000 | -0.2075655 | -0.3815435 | -1.2616693 | 0.0000000 | -0.2545716 | -0.8325255 | 0.000000 | 0.0000000 | 0.0000000 | -2.0915120 | -0.4524391 | -0.4063305 | 0.0000000 | 0.0000000 | -0.1817396 | 0.0000000 | -0.7607695 | 0.0000000 | 0.000000 | 3 | 1 | 228.54149 | 178.302898 |
| 0.0000000 | -1.4858397 | -3.4021625 | 0.0000000 | -0.3622659 | 0.000000 | -0.2075655 | -0.3815435 | -1.2616693 | 0.0000000 | -0.2545716 | -0.8325255 | 0.000000 | 0.0000000 | 0.0000000 | -2.0915120 | -0.4524391 | -0.4063305 | 0.0000000 | 0.0000000 | -0.1817396 | 0.0000000 | -0.7607695 | 0.0000000 | 0.000000 | 4 | 1 | 228.54149 | 178.302898 |
| 0.0000000 | -1.4858397 | -3.4021625 | 0.0000000 | -0.3622659 | 0.000000 | -0.2075655 | -0.3815435 | -1.2616693 | 0.0000000 | -0.2545716 | -0.8325255 | 0.000000 | 0.0000000 | 0.0000000 | -2.0915120 | -0.4524391 | -0.4063305 | 0.0000000 | 0.0000000 | -0.1817396 | 0.0000000 | -0.7607695 | 0.0000000 | 0.000000 | 5 | 1 | 228.54149 | 178.302898 |
| 0.0000000 | -1.4858397 | -3.4021625 | 0.0000000 | -0.3622659 | 0.000000 | -0.2075655 | -0.3815435 | -1.2616693 | 0.0000000 | -0.2545716 | -0.8325255 | 0.000000 | 0.0000000 | 0.0000000 | -2.0915120 | -0.4524391 | -0.4063305 | 0.0000000 | 0.0000000 | -0.1817396 | 0.0000000 | -0.7607695 | 0.0000000 | 0.000000 | 6 | 1 | 228.54149 | 178.302898 |
| 0.0000000 | -1.4858397 | -3.4021625 | 0.0000000 | -0.3622659 | 0.000000 | -0.2075655 | -0.3815435 | -1.2616693 | 0.0000000 | -0.2545716 | -0.8325255 | 0.000000 | 0.0000000 | 0.0000000 | -2.0915120 | -0.4524391 | -0.4063305 | 0.0000000 | 0.0000000 | -0.1817396 | 0.0000000 | -0.7607695 | 0.0000000 | 0.000000 | 7 | 1 | 228.54149 | 178.302898 |
| 0.0000000 | -1.4858397 | -3.4021625 | 0.0000000 | -0.3622659 | 0.000000 | -0.2075655 | -0.3815435 | -1.2616693 | 0.0000000 | -0.2545716 | -0.8325255 | 0.000000 | 0.0000000 | 0.0000000 | -2.0915120 | -0.4524391 | -0.4063305 | 0.0000000 | 0.0000000 | -0.1817396 | 0.0000000 | -0.7607695 | 0.0000000 | 0.000000 | 8 | 1 | 228.54149 | 178.302898 |
| 0.0000000 | -1.4858397 | -3.4021625 | 0.0000000 | -0.3622659 | 0.000000 | -0.2075655 | -0.3815435 | -1.2616693 | 0.0000000 | -0.2545716 | -0.8325255 | 0.000000 | 0.0000000 | 0.0000000 | -2.0915120 | -0.4524391 | -0.4063305 | 0.0000000 | 0.0000000 | -0.1817396 | 0.0000000 | -0.7607695 | 0.0000000 | 0.000000 | 9 | 1 | 228.54149 | 178.302898 |
| 0.0000000 | -1.4858397 | -3.4021625 | 0.0000000 | -0.3622659 | 0.000000 | -0.2075655 | -0.3815435 | -1.2616693 | 0.0000000 | -0.2545716 | -0.8325255 | 0.000000 | 0.0000000 | 0.0000000 | -2.0915120 | -0.4524391 | -0.4063305 | 0.0000000 | 0.0000000 | -0.1817396 | 0.0000000 | -0.7607695 | 0.0000000 | 0.000000 | 10 | 1 | 228.54149 | 178.302898 |
| 0.6027209 | -27.3608416 | 0.0000000 | -1.2304469 | -0.9615423 | 0.000000 | -0.0080291 | -0.4612881 | -3.5402377 | 0.0000000 | 0.0000000 | 0.0000000 | 0.000000 | 0.0000000 | 0.0000000 | -4.8806689 | 0.0000000 | 0.0000000 | -3.1047302 | -1.1570053 | 0.0000000 | 0.0000000 | 0.0000000 | -1.3639490 | -66044.297007 | 1 | 2 | 81.91198 | 31.906184 |
| 0.1647518 | -0.7593380 | -1.2728899 | -0.8210473 | -2.1291607 | 0.000000 | 0.0000000 | -0.6866965 | 0.0000000 | -12.1956088 | 0.0000000 | -1.2037327 | 0.000000 | 0.0000000 | 0.0000000 | -42.8592510 | 0.0000000 | 0.0000000 | -4.1239070 | -0.5223577 | 0.0000000 | 0.0000000 | 0.0000000 | 0.0000000 | 0.000000 | 2 | 2 | 71.89698 | 21.882468 |
| 0.0000000 | -0.5970173 | -0.4379183 | 0.0000000 | -2.5680244 | 0.000000 | 0.0000000 | -3.3796233 | 0.0000000 | -10.7272089 | 0.0000000 | 0.0000000 | 0.000000 | 0.0000000 | 0.0000000 | -3.2990569 | 0.0000000 | -0.1197931 | -0.9327914 | -0.2216907 | 0.0000000 | 0.0000000 | 0.0000000 | -0.8454046 | 0.000000 | 3 | 2 | 91.16452 | 41.156853 |
| 3.0519044 | -42.1569611 | -7.7514458 | -7.9891238 | -0.8881886 | 0.000000 | 0.0000000 | 0.0000000 | 0.0000000 | 0.0000000 | 0.0000000 | 0.0000000 | 0.000000 | -5.0012497 | 0.0000000 | -43.6614295 | 0.0000000 | 0.0000000 | 0.0000000 | 0.0000000 | 0.0000000 | -116.6698624 | -1.0532893 | 0.0000000 | -18108.553464 | 5 | 2 | 61.80063 | 11.787496 |
| 0.0000000 | -48.3065149 | -4.2729111 | -4.1897378 | -14.2294355 | 0.000000 | -195.0678412 | -0.2213107 | 0.0000000 | 0.0000000 | 0.0000000 | 0.0000000 | 0.000000 | 0.0000000 | -1.5678243 | -7.1922374 | 0.0000000 | 0.0000000 | 0.0000000 | 0.0000000 | 0.0000000 | -0.3515366 | -0.5902589 | 0.0000000 | 0.000000 | 6 | 2 | 60.63384 | 10.619479 |
| 0.9713911 | -55.3597505 | 0.0000000 | -2.9277152 | -1.1879410 | 0.000000 | 0.0000000 | 0.0000000 | 0.0000000 | 0.0000000 | 0.0000000 | 0.0000000 | 0.000000 | 0.0000000 | 0.0000000 | -90.5484514 | 0.0000000 | 0.0000000 | -3.0881283 | -2.1726431 | 0.0000000 | 0.0000000 | -0.0600953 | 0.0000000 | -7131.133679 | 7 | 2 | 76.09741 | 26.097408 |
| 0.0000000 | -0.2303232 | -0.9003691 | 0.0000000 | -1.2142376 | 0.000000 | 0.0000000 | -1.6152067 | 0.0000000 | -10.1559733 | -5.9600419 | -2.7222918 | 0.000000 | 0.0000000 | 0.0000000 | 0.0000000 | 0.0000000 | -0.5828403 | 0.0000000 | 0.0000000 | 0.0000000 | 0.0000000 | 0.0000000 | 0.0000000 | 0.000000 | 8 | 2 | 122.94112 | 72.934014 |
| 8.6039171 | -43.3004608 | -4.6879170 | -4.1035185 | -0.7221488 | 0.000000 | -11.0364746 | -5.4802732 | -5.3006895 | -0.7717544 | 0.0000000 | 0.0000000 | 0.000000 | 0.0000000 | 0.0000000 | -9.5354063 | 0.0000000 | -2.8338594 | -5.6387532 | -1.3387677 | 0.0000000 | 0.0000000 | 0.0000000 | -8.3618554 | 0.000000 | 9 | 2 | 63.54199 | 13.534921 |
| 0.0000000 | -0.2479392 | -4.4859441 | 0.0000000 | -2.1054872 | 0.000000 | 0.0000000 | -0.9685270 | -0.7115333 | 0.0000000 | -4.2695748 | -1.0860819 | 0.000000 | 0.0000000 | 0.0000000 | -3.2471444 | -0.4087417 | 0.0000000 | 0.0000000 | -0.1895801 | 0.0000000 | 0.0000000 | 0.0000000 | 0.0000000 | 0.000000 | 10 | 2 | 105.36095 | 55.360951 |
| 0.2984869 | 0.0000000 | 0.0000000 | -2.0448866 | -2.4240973 | -0.549429 | -0.0355236 | -18.7082912 | 0.0000000 | 0.0000000 | -19.8484544 | 0.0000000 | -2.969704 | -1.0939412 | -0.2656075 | -5.0227610 | -1.4740605 | -8.8919395 | 0.0000000 | 0.0000000 | 0.0000000 | -1.7954391 | -12.5238136 | 0.0000000 | -6.702911 | 1 | 3 | 50.00724 | 0.000000 |
| 2.5109152 | -3.1956156 | 0.0000000 | 0.0000000 | -2.6585908 | -7.384802 | -12.2215936 | -8.9286669 | -0.0000597 | -0.0000612 | -32.5547929 | -0.0666659 | -34.894874 | -0.1536351 | 0.0000000 | -0.0000217 | -0.0009990 | -0.0000008 | -6.2800572 | -20.7180233 | 0.0000000 | 0.0000000 | -0.2280281 | -0.1054528 | -175.840724 | 5 | 3 | 50.00151 | 0.001511 |
| 0.0000000 | 0.0000000 | 0.0000000 | -49.8635741 | -3.1373985 | 0.000000 | -0.5844768 | -0.1663822 | 0.0000000 | -20.0141157 | -0.6854162 | 0.0000000 | -16.257419 | 0.0000000 | 0.0000000 | 0.0000000 | 0.0000000 | 0.0000000 | 0.0000000 | 0.0000000 | 0.0000000 | 0.0000000 | -0.1464437 | 0.0000000 | 0.000000 | 7 | 3 | 60.89418 | 10.894185 |
